# Importance of dipole moments and ambient polarity for the conformation of Xaa–Pro moieties – a combined experimental and theoretical study†
†Electronic supplementary information (ESI) available: Synthesis and analytical details of all compounds, details on the computational analysis. See DOI: 10.1039/c5sc02211h


**DOI:** 10.1039/c5sc02211h

**Published:** 2015-08-12

**Authors:** Christiane Siebler, Boris Maryasin, Michael Kuemin, Roman S. Erdmann, Carla Rigling, Claudio Grünenfelder, Christian Ochsenfeld, Helma Wennemers

**Affiliations:** a Laboratory of Organic Chemistry , D-CHAB , ETH Zürich , Vladimir Prelog Weg 3 , CH-8093 Zürich , Switzerland . Email: Wennemers@org.chem.ethz.ch; b Chair of Theoretical Chemistry , Department of Chemistry , University of Munich (LMU) , Butenandtstr. 7 , D-81377 Munich , Germany; c Center of Integrated Protein Science (CIPSM) at the Department of Chemistry , University of Munich (LMU) , Butenandtstr. 5-13 , D-81377 Munich , Germany

## Abstract

NMR spectroscopic studies with a series of proline derivatives revealed that the polarity of the environment has a significant effect on the *trans* : *cis* isomer ratio of Xaa–Pro bonds. Computational studies showed that this effect is due to differences in the overall dipole moments of *trans* and *cis* conformers. Comparisons between the conformational properties of amide and ester derivatives revealed an intricate balance between polarity effects and n → π* interactions of adjacent carbonyl groups. The findings have important implications for protein folding and signaling as well as the performance of proline-based stereoselective catalysts.

## Abstract 2

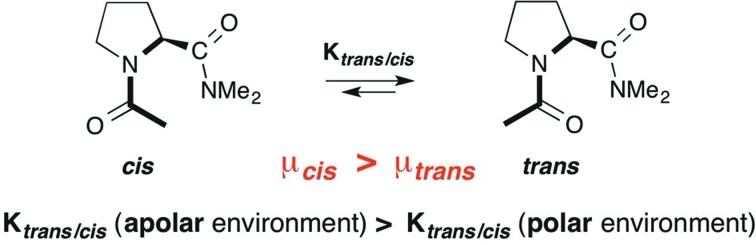
The *trans : cis* isomer ratio of Xaa–Pro bonds is significantly affected by the polarity of the environment. Computational and NMR spectroscopic studies revealed an intricate balance between polarity effects and interactions of carbonyl groups.

## Introduction

**Fig. 1:**
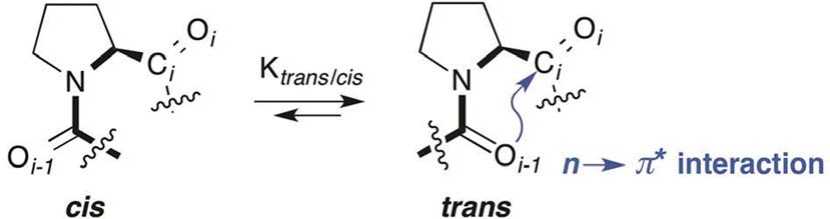
*Trans* and *cis* conformers of Xaa–Pro bonds.

Proline, the only cyclic proteinogenic amino acid, is often critically involved in protein folding and signaling.^1^ Prominent examples are collagen and proline-rich protein domains with two or more adjacent proline residues.^2^,^3^ Key to this unique role of proline is the isomerization of tertiary Xaa–Pro amide bonds between *cis* and *trans* conformers (Fig. 1).^1^–^4^ These moieties are present in water exposed domains of proteins as well as hydrophobic environments within membrane proteins.^1^,^5^,^6^ Furthermore, peptides bearing Xaa–Pro moieties have become popular as metal-free catalysts for a range of different reactions, including stereoselective C–C bond formations and acyl transfer reactions.^7^,^8^ The majority of these peptidic catalysts perform best in organic solvents. Understanding the factors that influence the *trans*–*cis* equilibrium of Xaa–Pro bonds in both aqueous and hydrophobic environment is therefore important.

The *trans* conformer is favored over the *cis* conformer in Xaa–Pro bonds by an interaction between the adjacent carbonyl groups (Fig. 1). This n → π* interaction involves the donation of electron density from the oxygen O_*i*–1_ lone pair (n) into the π* orbital of the adjacent carbonyl group (C_*i*_ = O_*i*_).^9^,^10^ In addition, steric effects can further favor or disfavor the *trans* over the *cis* conformer.^11^

To tune the conformational and functional properties of peptides and proteins, numerous proline derivatives with electron withdrawing, sterically demanding, or H-bond donating substituents, *e.g.* at C(4), have been developed that favor or disfavor the *trans* over the *cis* conformer compared to unsubstituted proline residues.^12^–^21^

**Fig. 2:**
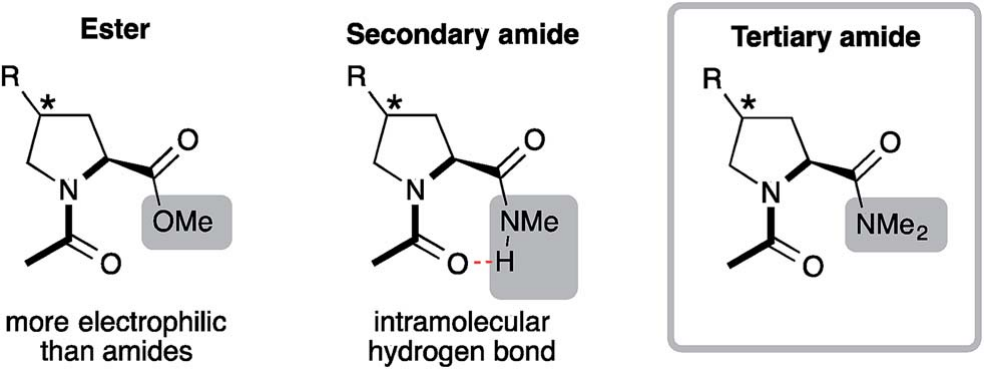
Acetylated model compounds bearing an ester (Ac-Xaa-OMe), secondary amide (Ac-Xaa-NHMe) and tertiary amide (Ac-Xaa-NMe_2_).

Acetylated methyl esters (Ac-Xaa-OMe) of proline and proline derivatives are commonly used model compounds to analyze the factors that determine the *trans* : *cis* conformer ratio (Fig. 2, left).^13^–^18^ They are preferred models compared to secondary amides Ac-Xaa-NHMe (Fig. 2, middle) that favor the *trans* conformer by donating an intramolecular H-bond and thereby obscure weaker interactions.^22^ Yet, esters are more electrophilic than amides and are therefore also not ideal models for analyzing the factors that affect the *trans* : *cis* amide equilibrium of Xaa–Pro bonds. Tertiary amides on the other hand cannot engage in an intramolecular H-bond and have a comparable electrophilicity as secondary amides (Fig. 2, right). They reflect in particular segments of peptides and proteins with two adjacent proline residues, which are common in collagen and proline-rich protein domains.^2^,^3^ We therefore envisioned acetylated dimethyl amides Ac-Xaa-NMe_2_ as appropriate models to allow insight into the factors that influence the *trans* : *cis* conformer ratio of Xaa–Pro bonds.

Herein we examined the conformational properties of dimethyl amide proline derivatives in aqueous and organic media by NMR spectroscopic and computational studies. We show that the *trans* : *cis* ratio of Xaa–Pro bonds is significantly affected by the polarity of the environment. Comparisons between different proline derivatives revealed that the polarity effects are in a fine balance with n → π* interactions. Furthermore, we demonstrate how the solvent affects the conformation and the catalytic performance of a tripeptidic catalyst.

## Results and discussion

### Conformational properties of proline derivatives Ac-Pro-OMe and Ac-Pro-NMe_2_ in polar and apolar solvents

#### NMR spectroscopic studies

**Fig. 3:**
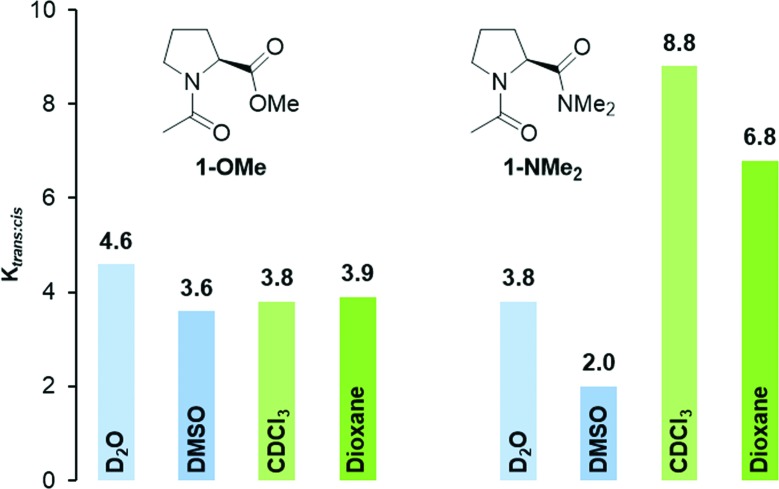
*K*_*trans*:*cis*_ values of Ac-Pro-OMe (**1-OMe**) and Ac-Pro-NMe_2_ (**1-NMe_2_**) determined by NMR-spectroscopy of 80 mM solutions in D_2_O and CDCl_3_, DMSO-d_6_, dioxane-d_8_.

We started by analyzing the conformational properties of the acetylated methyl ester and dimethyl amide of proline, Ac-Pro-OMe (**1-OMe**) and Ac-Pro-NMe_2_ (**1-NMe_2_**), by ^1^H NMR spectroscopy. Spectra of **1-OMe** and **1-NMe_2_** were recorded in the polar solvents D_2_O and DMSO-d_6_ as well as CDCl_3_ and dioxane-d_8_ as representatives of less polar solvents. All spectra showed two sets of signals corresponding to minor *cis* and major *trans* conformers due to their slow interconversion.^23^ In agreement with previous studies, *trans* : *cis* ratios of 4.6 and 3.8 were observed for **1-OMe** and **1-NMe_2_** in D_2_O, respectively (Fig. 3, light blue).^9^ Similarly the *trans* : *cis* conformer ratio of **1-OMe** is also in DMSO-d_6_ (*K*_*trans*/*cis*_ = 3.6) higher compared to that of **1-NMe_2_** (*K*_*trans*/*cis*_ = 2.0, Fig. 3, blue). The lower *trans* : *cis* ratios of the dimethyl amide in polar solvents are indicative of weaker n → π* interactions between the adjacent carbonyls and in agreement with the lower electrophilicity of amide compared to ester moieties.

Surprisingly, in the less polar solvents CDCl_3_ and dioxane-d_8_ the opposite trend was observed. In both solvents, the *trans* : *cis* conformer ratio of the amide **1-NMe_2_** is significantly higher compared to that of the ester **1-OMe** (Fig. 3, green and light green). For example, in CDCl_3_ the equilibrium constant *K*_*trans*/*cis*_ of **1-NMe_2_** is more than twice as high as that of **1-OMe** (*K*_*trans*/*cis*_ = 8.8 and 3.8, respectively).

These findings can neither be explained by interactions between adjacent carbonyl groups nor steric effects and show that an additional factor is contributing to the *trans* : *cis* conformer ratio of Xaa–Pro bonds. They are also unexpected with regard to the conformational properties of oligoprolines with more than six residues that adopt PPII helices with *all-trans* amide bonds in water and PPI helices with *all-cis* amide bonds in less polar solvents (*e.g.*^i^PrOH).^24^,^25^ These conformational preferences are due to hydration of the amides in PPII helices where they are oriented perpendicular to the axis and therefore solvent exposed, whereas the amides in PPI helices are aligned along the axis and not solvent exposed.^25^ The preference of the *trans*-conformer of **1-NMe_2_** in apolar solvents observed herein suggests that the helicity of oligoprolines precludes conformation-directing effects that are important within small peptides and segments of proteins without defined secondary structures.

Since the polarity of the solvent led to the observed differences between the prolyl ester and amide, we suspected that differences in the polarity of the *trans* and *cis* conformers are key for the observed equilibrium constants *K*_*trans*/*cis*_. To evaluate this hypothesis, we determined the overall dipole moments of the *trans* and *cis* conformers of the ester **1-OMe** and the amide **1-NMe_2_** by quantum chemical calculations.

#### Computational studies

**Fig. 4:**
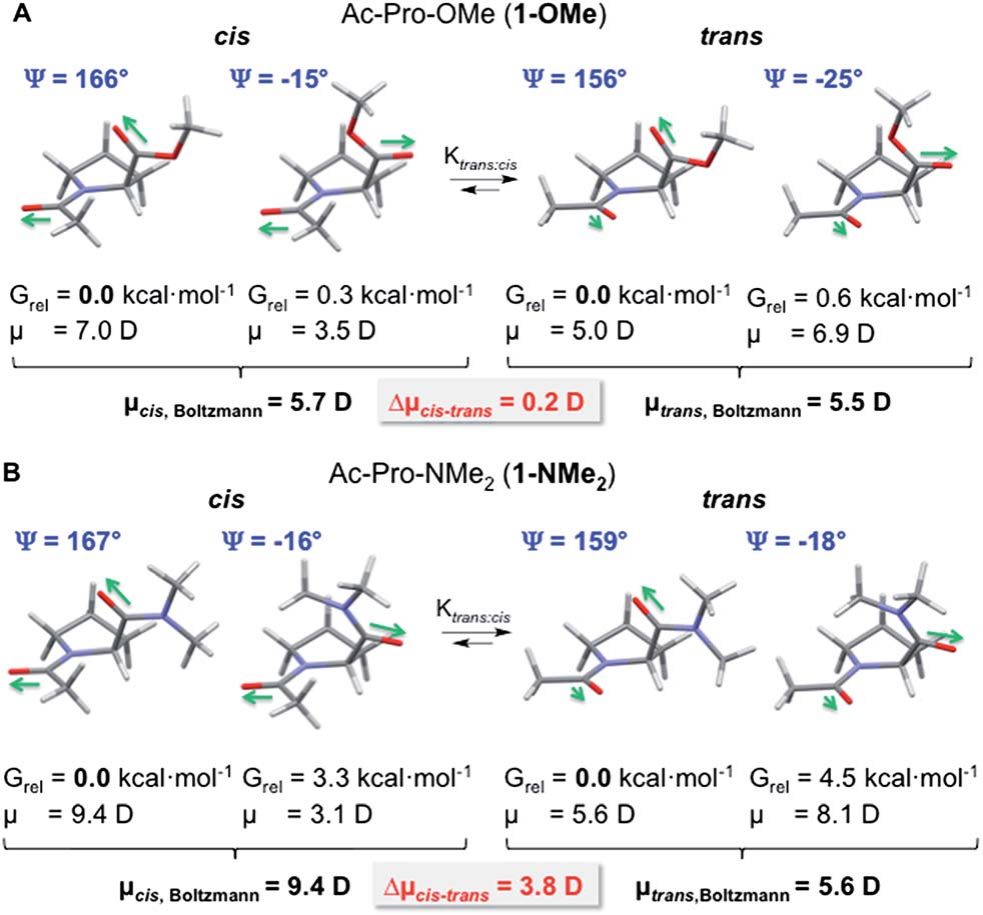
Lowest energy structures and their relative Gibbs free energies at 298 K (*G*_rel_) and Boltzmann-averaged dipole moments *μ* [D] of *trans* and *cis* conformers of Ac-Pro-OMe (**1-OMe**) and Ac-Pro-NMe_2_ (**1-NMe_2_**) calculated with CHCl_3_ as solvent at the PBE0-D3-COSMO/def2-TZVP level of theory. For clarity, only the values of the C(4)-*endo* conformers are listed. The values hardly change when also the C(4)-*exo* conformers are taken into account, see the ESI.†

We started by determining the geometries of the lowest energy conformations of the *cis* and *trans* isomers at the PBE0-D3/def2-TZVP level of theory using the Turbomole program package (versions 6.3.1 and 6.6).^26^,^27^ Thermal corrections for obtaining the Gibbs free energies at 298.15 K were calculated for all minima from unscaled vibrational frequencies obtained at the same level and were combined with single point energies calculated at the RI-MP2(ref. 28)/def2-QZVP//PBE0-D3/def2-TZVP level to yield Gibbs free energies at 298.15 K. Solvation effects were considered by the implicit solvation model COSMO.^29^ We employed this continuum solvation model rather than explicit solvent molecules that would require difficult sampling over the conformational space of solvent molecule arrangements and, in particular, would make the definition of the dipole moment difficult, since it is not well defined how many of the solvent molecules would need to be included in the dipole moment calculation. For both proline derivatives C(4)-*endo* puckered pyrrolidine rings were predicted to be energetically slightly more favored (by <1 kcal mol^–1^) compared to C(4)-*exo* puckers in case of the *cis* and the *trans* conformers. This is in good agreement with crystal structures and previously calculated structures of proline and proline derivatives.^30^,^31^ In the lowest energy structures of the *trans* conformers of both **1-OMe** and **1-NMe_2_** (Fig. 4, *G*_rel_ = 0.0 kcal mol^–1^) the *Ψ*-angles are ∼155° and the typical indicators of n → π* interactions between the adjacent carbonyl groups are present:^9^,^10^,^32^ The O_*i*–1_···C_*i*_ distances are within the van-der-Waals radii of the interaction partners (<3.2 Å), the Bürgi–Dunitz trajectory angles O_*i*–1_···C_*i*_–O_*i*_ (*Θ*_BD_) are around 95°, and C_*i*_ is not planar but pyramidalized (see ESI for details†). The values are less pronounced in case of the amide compared to the ester (*e.g.*, O_*i*–1_···C_*i*_ 3.10 Å **1-NMe_2_***versus* 3.02 Å **1-OMe**). This is indicative of weaker n → π* interactions as expected for the less electrophilic amide.

For the *trans* and *cis* conformers of the ester **1-OMe**, additional low energy structures (*G*_rel_ < 1 kcal mol^–1^) with *Ψ*-angles of approximately –20° were found (Fig. 4A). Conformations with similar *Ψ*-angles are also in case of the dimethyl amide **1-NMe_2_** the next lowest energy structures. Yet, they have significantly higher energies (*G*_rel_ > 3 kcal mol^–1^) due to steric repulsion between the dimethyl amide moiety and the pyrrolidine ring (Fig. 4B).

Rotation around the *Ψ*-angle affects the relative orientation of the carbonyl moieties significantly and was expected to affect the overall dipole moments of the *cis* and *trans* conformers. We therefore systematically changed the *Ψ*-angle within the identified lowest energy structures by steps of 30° and performed a constrained geometry optimization of each of these conformers to ensure that the conformations with *Ψ* = ∼160° and –20° are the global and local energy minimum structures. The dipole moments of these global and local energy minima structures were calculated on the same level of theory (PBE0-D3/def2-TZVP) as the Gibbs free energies. Overall dipole moments were then calculated as Boltzmann-averaged values over all available *cis* and *trans* conformers, respectively, of **1-OMe** and **1-NMe_2_** based on their Gibbs free energies (Fig. 4, see the ESI for details†).

The overall dipole moments of the *trans* and *cis* conformers of ester **1-OMe** are almost identical (Δ*μ*_*cis*–*trans*_ = 0.2 D) since polarity differences are leveled out by the almost equal population of conformers with *Ψ*-angles of 160° and –20°. In contrast, the *cis* conformer of amide **1-NMe_2_** has a significantly higher dipole moment compared to the respective *trans* conformer (Δ*μ*_*cis*–*trans*_ = 3.8 D). This is due to the higher population of the conformers with *Ψ*-angles around 160° within which the adjacent carbonyl moieties point into similar directions in the *cis* conformer but into different directions in the *trans* conformer (Fig. 4B).

These differences in the dipole moments corroborate the experimental findings: the less polar *trans* conformer is favored over the *cis* conformer significantly more in apolar than polar solvents. This is reflected in the observed higher *trans* : *cis* conformer ratio of the amide **1-NMe_2_** in apolar compared to polar solvents (*e.g.*, *K*_*trans*/*cis*_ = 8.8 in CDCl_3_ and *K*_*trans*/*cis*_ = 3.8 in D_2_O, Fig. 3). Since the n → π* interaction between the adjacent amide groups is weak the *trans* : *cis* conformer ratios of amide **1-NMe_2_** is mainly controlled by the polarity of the environment. The situation is different in case of the ester **1-OMe**. Here, the polarity of the environment has a minor effect and the strength of the n → π* interaction between the adjacent carbonyl groups controls *K*_*trans*/*cis*_.‡
‡It is noteworthy that the *trans* : *cis* ratios of the esters are generally higher in D_2_O than CDCl_3_. This could be due to a better solvation and thereby stabilization of the increased charge separation induced by the n → π* interaction between the adjacent carbonyl groups within the *trans* conformer.
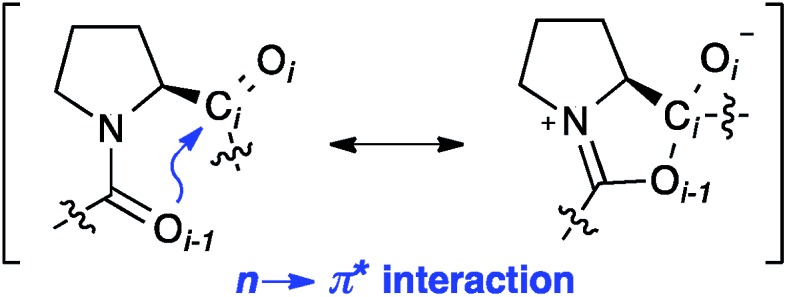




### Conformational properties of proline derivatives bearing electron withdrawing substituents

**Fig. 5:**
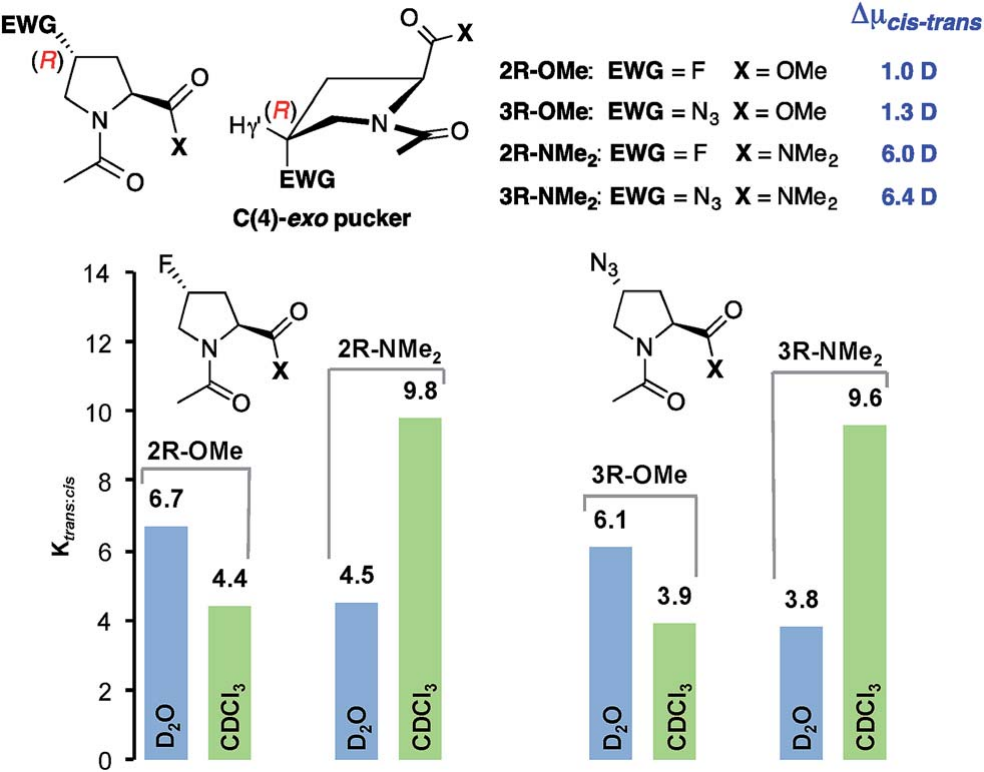
*K*_*trans*:*cis*_ values of (4*R*)-configured Ac-Xaa-OMe and Ac-Xaa-NMe_2_ of Flp and Azp derivatives determined by NMR-spectroscopy of 80 mM solutions in D_2_O (blue) and CDCl_3_ (green) and difference in the dipole moments (Δ*μ*_*cis*–*trans*_) of *trans* and *cis* conformers calculated with CHCl_3_ as solvent at the PBE0-D3-COSMO/def2-TZVP level of theory.

**Fig. 6:**
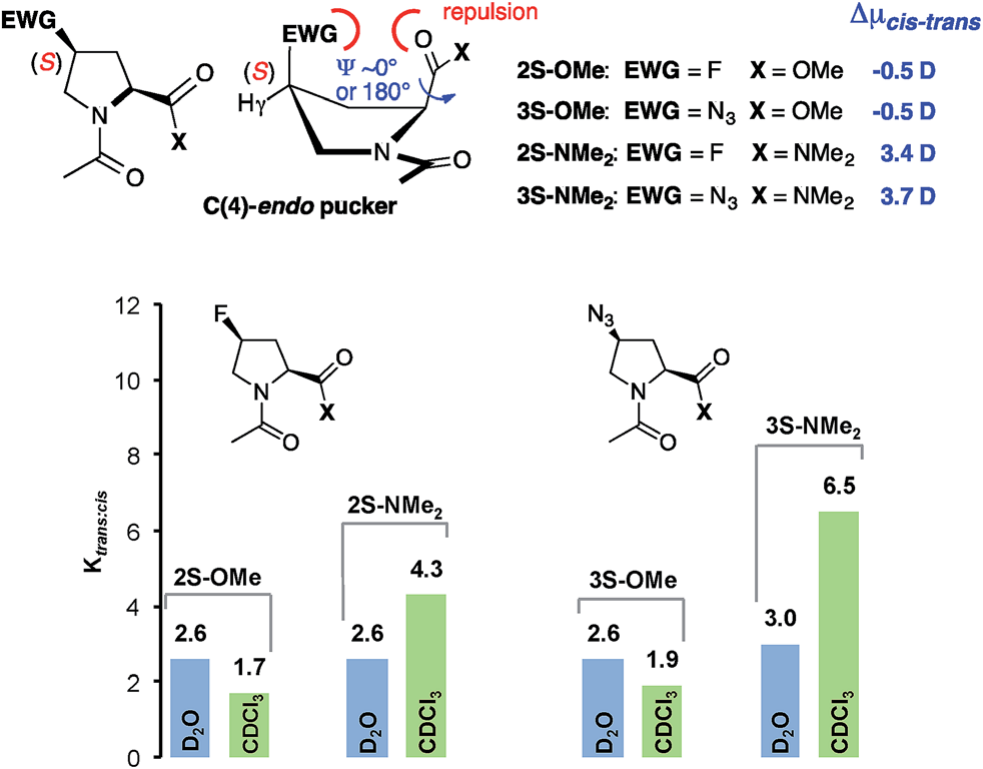
*K*_*trans*:*cis*_ values of (4*S*)-configured Ac-Xaa-OMe and Ac-Xaa-NMe_2_ of Flp and Azp derivatives determined by NMR-spectroscopy of 80 mM solutions in D_2_O (blue) and CDCl_3_ (green) and difference in the dipole moments (Δ*μ*_*cis*–*trans*_) of *trans* and *cis* conformers calculated with CHCl_3_ as solvent at the PBE0-D3-COSMO/def2-TZVP level of theory.

To probe the generality of these findings, we analyzed the solvent dependence of the *trans* : *cis* conformer ratios of (4*S*)- and (4*R*)-configured fluoroproline (Flp) and azidoproline (Azp) methyl esters (Ac-Xaa-OMe) and dimethyl amides (Ac-Xaa-NMe_2_). Fluoroprolines are among the most often used proline derivatives for tuning the *trans* : *cis* conformer ratio within peptides and proteins since they can be incorporated by chemical synthesis and protein expression into peptides and proteins.^12^,^16^ Azidoprolines are attractive since they allow for further derivatisation by, *e.g.*, “click chemistry”.^14^,^17^,^33^ The electron-withdrawing fluoro and azido substituents are known to control the ring puckering of these derivatives by a stereoelectronic gauche effect.^16^,^17^ This leads in case of (4*R*)Flp and (4*R*)Azp to a preference of C(4)-*exo* puckering whereas (4*S*)Flp and (4*S*)Azp adopt C(4)-*endo* puckers preferentially (Fig. 5 and 6, top middle). Thus, these derivatives allow for probing whether the polarity effect is affected by the ring pucker and substituents at C(4).

Calculations of the difference in the dipole moments (Δ*μ*_*cis*–*trans*_) of the *cis* and *trans* conformers with the lowest energies of all four proline derivatives by the methods described above showed a similar trend as for the unsubstituted prolines **1-OMe** and **1-NMe_2_** (Fig. 5 and 6, see the ESI for the lowest energy structures†): regardless of the absolute configuration at C(4) and the ring pucker, the overall dipole moments of the *trans* and *cis* conformers of the methyl esters (**2*R***/**2*S***/**3*R***/**3*S*–OMe**) are almost identical (Δ*μ*_*cis*–*trans*_ = ±1 D) whereas those of the *trans* conformers of the amides (**2*R***/**2*S***/**3*R***/**3*S*–NMe_2_**) are significantly higher compared to those of the respective *cis* conformers (Δ*μ*_*cis*–*trans*_ = 2.5–6.3 D).

Thus, a similar trend is expected for the *trans* : *cis* conformer ratios in polar *versus* less polar solvents for these substituted proline derivatives as for **1-OMe** and **1-NMe_2_**. Indeed, in D_2_O the *trans* : *cis* conformer ratios of the (4*R*)-configured dimethyl amides **2*R*-NMe_2_** and **3*R*-NMe_2_** are lower than those of the methyl esters Ac-(4*R*)Flp-OMe **2*R*-OMe** and Ac-(4*R*)Azp-OMe **3*R*-OMe** (Fig. 5, blue).^16^,^17^ This reflects the lower electrophilicity of the amide compared to the ester and the thereby weakened n → π* interaction. In CDCl_3_ this trend is reversed and the *trans* : *cis* conformer ratios of the amides are significantly higher than those of the esters (Fig. 5, green), which underscores that the polarity controls their conformational properties.

In the diastereoisomeric (4*S*)-configured esters Ac-(4*S*)Flp-OMe **2*S*–OMe** and Ac-(4*S*)Azp-OMe **3*S*–OMe** the *trans* conformer is generally less favored (*K*_*trans*/*cis*_ = 2.6) since a transannular electronic repulsion between the electron-rich F or N_3_ substituent and the carbonyl oxygen of the methyl ester or amide moiety places the adjacent carbonyl groups in an unfavorable position for an n → π* interaction (Fig. 6, top middle).^18^ Thus, the contribution of the n → π* interaction is less compared to that in unsubstituted proline.^16^,^17^ As a result, the esters and respective dimethyl amides have comparable *trans* : *cis* amide ratios in D_2_O (Fig. 6, blue). In CDCl_3_ the *trans* : *cis* conformer ratios of the amides are higher by a factor of 2–3 compared to those of the respective esters (Fig. 6, green). Thus, the *trans* : *cis* ratio is also within these substituted proline-amide derivatives predominantly controlled by the polarity of the solvent and the difference in the dipole moments of the *trans* and *cis* conformers.§
§For a detailed listing of the calculated dipole moments and the structures, see the ESI.†



### *Trans* : *cis* isomer ratio of short peptides with the Pro–Pro motive

**Fig. 7:**
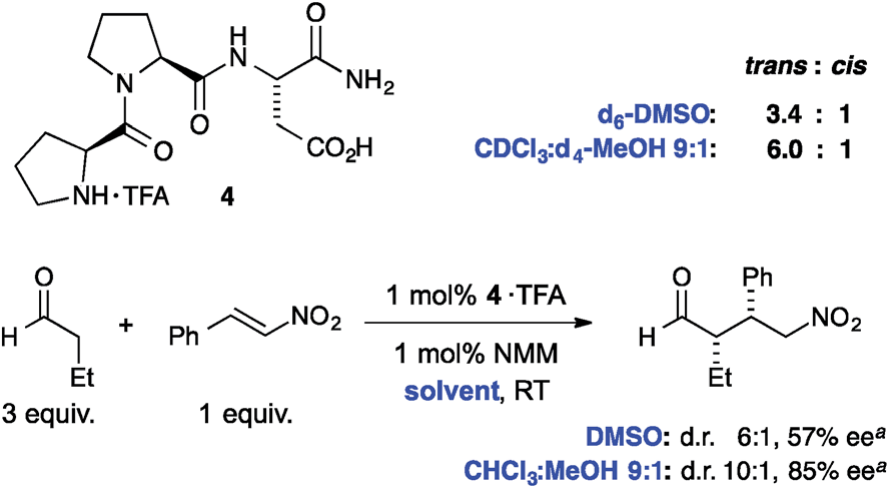
*Trans* : *cis* ratios of the tripeptidic catalyst H-Pro-Pro-Asp-NH_2_ (**4**) and stereoselectivities of 1,4-addition reactions between butanal and nitrostyrene. ^a^Data taken from ref. 35.

Finally we probed whether the observed polarity effects also occur in short-chain peptides. Towards this goal we examined the *trans* : *cis* isomer ratio of the tripeptide H-Pro-Pro-Asp-NH_2_ (**4**) that is a known catalyst for aldol and conjugate addition reactions between aldehydes and nitroolefins (Fig. 7).^34^–^36^ Reassuringly, NMR spectroscopic analyses of the trifluoroacetic acid (TFA) salt of **4** in DMSO-d_6_ showed a *trans* : *cis* ratio of 3.4 : 1, whereas the *trans* : *cis* ratio is 6.0 : 1 in a mixture of CDCl_3_ : MeOH-d_4_ 9 : 1 (the peptide is not soluble in pure CDCl_3_). Thus, the *trans* isomer is also within this tripeptide more favored in apolar compared to polar environments, which shows how important the choice of the solvent is for the conformational properties of short chain peptides with the Pro–Pro motive.

Interestingly, the diastereoselectivity (d.r.) and enantioselectivity (ee) of this peptidic catalyst in C–C bond forming reactions correlates with the *trans* : *cis* amide ratios and are significantly higher in a mixture of CHCl_3_ : MeOH 9 : 1 than DMSO (Fig. 7, bottom). Whereas the polarity of the solvent is likely also affecting other factors, *e.g.*, differences in the interaction strength of a putative imminium–nitronate intermediate with the carboxylic acid moiety of the catalyst,^36^ the data indicates that the *trans* : *cis* conformer ratio correlates with the stereochemical outcome of the reaction.

## Conclusions

In conclusion, we have shown how important the polarity of the environment is for the *trans* : *cis* conformer equilibrium of proline derivatives that are connected by tertiary amide groups. Polar solvents favor the *cis* conformer, apolar environments the less polar *trans* conformer. The results also show that the *trans* : *cis* ratio of Xaa–Pro bonds is controlled by a fine balance of n → π* interactions between adjacent amides and the polarity of the environment. Since both are comparatively weak interactions, subtle structural or environmental changes can affect their contributions significantly. The results are particularly relevant for peptides and proteins containing Pro–Pro moieties, which are common motives in, *e.g.*, collagen and catalytically active peptides. The presented findings provide a guide for influencing the *trans* : *cis* conformer ratio *via* the environment and thereby control the conformational and folding properties of peptides and proteins.

## Supplementary Material

Supplementary information
