# Supplementary material for: Importance of dipole moments and ambient polarity for the conformation of Xaa–Pro moieties – a combined experimental and theoretical study
Source: Chem Sci. 2015 Aug 12;6(12):6725–30. doi: 10.1039/c5sc02211h (PMC6090429; doi:10.1039/c5sc02211h)
Supplement: Supplementary file 1 [file SC-006-C5SC02211H-s001.pdf]

Electronic Supplementary Material (ESI) for Chemical Science.
This journal is © The Royal Society of Chemistry 2015

Importance of Dipole Moments and Ambient Polarity for the Conformation of Xaa-Pro Moieties – A Combined Experimental and Theoretical Study

Christiane Siebler,[a] Boris Maryasin,[b] Michael Kümin,[a] Roman S. Erdmann,[a] Carla Rigling,[a] Claudio Grünenfelder,[a] Christian Ochsenfeld*[b] and Helma Wennemers*[a]

[a] Laboratory of Organic Chemistry, D-CHAB, ETH Zurich,
Vladimir-Prelog-Weg 3
8093 Zurich, Switzerland
Email: helma.wennemers@org.chem.ethz.ch

[b] Chair of Theoretical Chemistry, Department of Chemistry, University of Munich (LMU), Butenandtstr. 7, D-81377 Munich, and Center of Integrated Protein Science (CIPSM) at the Department of Chemistry, University of Munich (LMU), Butenandtstr. 5-13, D-81377 Munich
Email: christian.ochsenfeld@cup.uni-muenchen.de

Table of contents

1. General aspects 3
2. Synthesis and Analytical Data of Proline Derivatives 4
   2.1. Ac-Pro-OMe (**1-OMe**) 4
   2.2. Ac-Pro-NMe_2 (**1-NMe$_2$**) 6
   2.3. Ac-(4*R*)Flp-OMe (**2R-OMe**) 10
   2.4. Ac-(4*R*)Flp-NMe_2 (**2R-NMe$_2$**) 12
   2.5. Ac-(4*R*)Azp-NMe_2 (**3R-NMe$_2$**) 16
   2.6. Ac-(4*S*)Flp-OMe (**2S-OMe**) 18
   2.7. Ac-(4*S*)Flp-NMe_2 (**2S-NMe$_2$**) 19
   2.8. Ac-(4*S*)Azp-NMe_2 (**3S-NMe$_2$**) 23
   2.9. H-Pro-Pro-Asp-NH_2 ·TFA (**4**) 25
3. Quantum Chemical Calculations of Methylesters and Dimethylamides of Proline Derivatives 27
   3.1. Gibbs free energies and dipole moments of Ac-Pro-OMe (**1-OMe**) and Ac-Pro-NMe_2 (**1-NMe$_2$**) 27
   3.2. Indicators for the n → π* Interaction in Ac-Pro-OMe (**1-OMe**) and Ac-Pro-OMe (**1-NMe$_2$**) 28
   3.3. Gibbs free energies and dipole moments of Ac-(4*R*)Flp-OMe (**2R-OMe**) and Ac-(4*R*)Flp-NMe_2 (**2R-NMe$_2$**) 29
   3.4. Gibbs free energies and dipole moments of Ac-(4*R*)Azp-OMe (**3R-OMe**) and Ac-(4*R*)Azp-NMe_2 (**3R-NMe$_2$**) 30
   3.5. Gibbs free energies and dipole moments of Ac-(4*S*)Flp-OMe (**2S-OMe**) and Ac-(4*S*)Flp-NMe_2 (**2S-NMe$_2$**) 35
   3.6. Gibbs free energies and dipole moments of Ac-(4*S*)Azp-OMe (**3S-OMe**) and Ac-(4*S*)Azp-NMe_2 (**3S-NMe$_2$**) 36
4. Cartesian Coordinates 41
5. Torsion Potential of Methylesters and Dimethylamides of Proline Derivatives 69
6. NMR Spectra 77
7. References 99

1. General aspects

Materials and reagents were of the highest commercially available grade and used without further purification. For solid phase peptide syntheses Rink Amide-ChemMatrix resin from Biotage was used. Reactions were monitored by thin layer chromatography using Merck silica gel 60 F254 plates. Compounds were visualized by UV and ninhydrin. Flash chromatography was performed using Merck silica gel 60, particle size 40 - 63 µm and Fluka silica gel 60 Å, 230-400 mesh particle size. ^{1}H and ^{13}C NMR spectra were recorded on Bruker DPX 400, Bruker AV 400 MHz and Bruker AVIII 600 MHz spectrometers. Chemical shifts are reported in ppm using TMS or the residual solvent peak as a reference. A Bruker Esquire 3000plus and a Bruker Amazon Speed instrument were used for electrospray ionization (ESI) mass spectrometry measurements. A Bruker microflex instrument was used for Maldi-MS analysis with α-Cyano-4-hydroxycinnamic acid (CHCA) as MALDI-MS Matrix. Analytical HPLC was performed using a LiChrospher 100 RP-18e 5 µm (250 mm x 4 mm) column from Merck. Preparative HPLC was carried out on a LiChrospher RP-18e 5 µm (250 mm x 10 mm) column from Merck. A Chirascan Plus (Applied Biophysics Ltd, Leatherhead, UK) was used for CD measurements. The solutions were measured in a quartz cell with a pathlength of 1.0 mm (Hellma 110-QS). For automated peptide synthesis, a Syro I Peptide Synthesizer (MultiSynTech GmbH, Witten, Germany) was employed.

2. Synthesis and Analytical Data of Proline Derivatives

2.1. Ac-Pro-OMe (1-OMe)

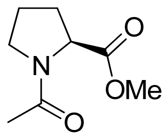

The compound was purchased from Bachem. The analytical data are in agreement with published data.[1]

^{1}H NMR (400 MHz, Deuterium Oxide); *trans* conformer δ / ppm = 4.46 (dd, *J* = 8.6, 4.3 Hz, 1H; Hα), , 3.77 (s, 3H; OMe), 3.73 – 3.60 (m, 2H; Hδ), 2.37 – 2.25 (m, 1H; Hβ), 2.13 (s, 3H; Ac), 2.09 – 1.96 (m, 2H; Hβ, Hγ). Isolated signals of the *cis* conformer δ / ppm = 4.72 (dd, *J* = 8.8, 2.7 Hz, 1H; Hα, overlapping with water), 3.82 (s, 3H; OMe), 3.60 – 3.44 (m, 1H; Hδ).

^{13}C NMR (101 MHz, Deuterium Oxide) δ / ppm = 175.0 (carbonyl), 173.0 (carbonyl), 59.0 (Cα), 52.9 (OMe), 48.4 (Cδ), 29.2 (Cβ), 24.2 (Cγ), 21.2 (Ac). Isolated signals of the *cis* conformer δ / ppm = 174.6, 60.7, 53.2, 46.6, 30.6, 22.3.

$K_{trans/cis}$ (400 MHz, Deuterium Oxide) = 4.6

^{1}H NMR (400 MHz, DMSO-d_6); *trans* conformer δ / ppm = 4.25 (dd, *J* = 8.7, 4.2 Hz, 1H; Hα), 3.60 (s, 3H; OMe), 3.58 – 3.46 (2 x dψt, *J* = 9.9, 6.8 Hz, 2H; Hδ), 2.20 – 2.10 (m, *J* = 12.2, 8.6, 7.4 Hz, 2H; Hβ), 1.97 (s, 3H; Ac), 1.95 – 1.86 (m, *J* = 7.1, 6.4 Hz, 2H; Hγ), 1.86 – 1.78 (m, *J* = 11.9, 4.4 Hz, 1H; Hβ). Isolated signals of the *cis* conformer δ / ppm = 4.60 (dd, *J* = 8.7, 2.6 Hz, 1H; Hα), 3.69 (s, 3H; OMe), 3.36 (dd, *J* = 8.5, 5.6 Hz, 2H; Hδ), 2.26 – 2.19 (m, *J* = 8.7, 3.8, 1.8 Hz, 1H; Hβ), 2.04 (dddd, *J* = 12.9, 6.8, 3.5, 2.6 Hz, 1H ; Hβ), 1.84 (s, 3H; Ac), 1.77 – 1.67 (m, 2H; Hγ).

^{13}C NMR (101 MHz, DMSO-d6); *trans* conformer δ / ppm = 172.55 (carbonyl), 168.32 (carbonyl), 58.01 (Cα), 51.70 (OMe), 47.22 (Cδ), 29.01 (Cβ), 24.40 (Cγ), 22.03 (Ac).

Isolated signals of the *cis* conformer δ / ppm = 172.77 (carbonyl), 168.48 (carbonyl), 59.21 (Cα), 52.33 (OMe), 45.77 (Cδ), 30.76(Cβ), 22.37 (Cγ).

$K_{trans/cis}$ (400 MHz, DMSO-d6) = 3.6

^{1}H NMR (400 MHz, Chloroform-d); *trans* conformer δ / ppm = 4.48 (dd, *J* = 8.7, 3.8 Hz, 1H; Hα), 3.72 (s, 3H; OMe), 3.69 – 3.45 (m, 2H; Hδ), 2.35 – 1.86 (m, 4H; Hβ, Hγ), 2.09 (s, 3H; Ac). Isolated signals of the *cis* conformer δ / ppm = 4.37 (dd, *J* = 8.6, 2.8 Hz, 1H; Hα), 3.76 (s, 3H; OMe), 1.97 (s, 3H; Ac).

^{13}C NMR (101 MHz, $CDCl_3$); *trans* conformer δ / ppm = 173.0 (carbonyl), 169.6 (carbonyl), 58.6 (Cα), 52.3 (OMe), 47.8 (Cδ), 29.6 (Cβ), 24.9 (Cγ), 22.4 (Ac). Isolated signals of the *cis* conformer δ / ppm = 60.3 (Cα), 52.7 (OMe), 46.4 (Cδ), 31.6 (Cβ), 22.9 (Cγ), 22.4 (Ac).

$K_{trans/cis}$ (400 MHz, Chloroform-d) = 3.9

^{1}**H NMR** (400 MHz, 1,4-Dioxane-d8); *trans* conformer δ / ppm = 4.33 (dd, *J* = 8.6, 3.8 Hz, 1H; Hα), 3.62 (s, 3H; OMe), 3.61 – 3.40 (m, 2H; Hδ), 2.27 – 1.77 (m, 4H; Hβ, Hγ), 1.95 (s, 3H; Ac). Isolated signals of the *cis* conformer δ / ppm = 4.39 (dd, *J* = 8.6, 2.7 Hz, 1H; Hα), 3.70 (s, 3H; OMe), 1.84 (s, 3H; Ac).

^{13}**C NMR** (101 MHz, 1,4-Dioxane-d8); *trans* conformer δ / ppm = 173.4 (carbonyl), 168.7 (carbonyl), 59.0 (Cα), 52.0 (OMe), 48.0 (Cδ), 30.0 (Cβ), 25.5 (Cα), 22.0 (Ac). Isolated signals of the *cis* conformer δ / ppm = 60.6 (Cα), 52.5 (OMe), 46.6 (Cδ), 32.0 (Cβ), 23.4 (Cγ), 22.0 (Ac).

$\mathbf{K_{trans/cis}}$ (400 MHz, 1,4-Dioxane-d8) = 3.9

2.2. Ac-Pro-NMe_2 (1-NMe_2)

Boc-Pro-NMe_2

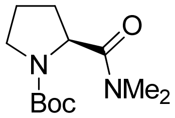

Boc-Pro-OH (500 mg, 2.32 mmol, 1.0 eq) was dissolved in CH_2Cl_2 (9.3 ml) and EDC·HCl (490 mg, 2.56 mmol, 1.1 eq), *i*Pr_2NEt (514 µL, 3.02 mmol, 1.3 eq) and $HNMe_2$·HCl (243 mg, 3.02 mmol, 1.3 eq) were added. The solution was stirred overnight and diluted with CH_2Cl_2 (50 ml) and washed with HCl (1M, 3 x 15 ml) and sat. $NaHCO_3$ (3 x 15 ml). The organic layer was dried over $MgSO_4$ and concentrated under reduced pressure. The residue was subjected to flash chromatography on silica (5% MeOH in CH_2Cl_2) to obtain the title compound (337 mg, 60%) as a colorless oil.

TLC R*f* = 0.36 (silica, 5% MeOH in CH_2Cl_2, ninhydrin).

(two isomers are visible in the ^{1}H NMR- and ^{13}C NMR-spectra in a ratio of ≈ 1.2:1 in $CDCl_3$).

^{1}H NMR (400 MHz, $CDCl_3$) δ/ppm = 4.68 (dd, *J* = 8.3 Hz, 3.2 Hz, 1H; Hα), 3.66-3.54 (m, 1H; Hδ), 3.54-3.37 (m, 1H; Hδ), 3.10 (s, 3H; NMe_2), 2.97 (s, 3H; NMe_2), 2.23-1.98 (m, 2H; Hβ), 1.92-1.79 (m, 2 H; Hβ), 1.47 (s, 9H). Isolated signals of the minor conformer: 4.55 (dd, *J* = 8.2 Hz, 4.3 Hz, 1H), 3.07 (s, 3H), 2.98 (s, 3H), 1.41 (s, 9H).

^{13}C NMR (101 MHz, $CDCl_3$) δ/ppm = 172.7 (amide), 154.9 (Boc), 79.8 (Boc), 56.7 (Cα), 47.2 (Cδ), 37.3 (NMe_2), 36.3 (NMe_2), 29.9 (Cβ), 28.9 (Boc), 24.6 (Cγ). Isolated signals of the minor conformer: 174.1 (amide), 154.7 (Boc), 79.8 (Boc), 56.8 (Cα), 47.0 (Cδ), 37.3 (NMe_2), 36.3 (NMe_2), 30.7 (Cβ), 28.7 (Boc), 24.0 (Cγ).

ESI-MS: *m/z* calcd for $C_{12}H_{22}N_2O_3$: 242.2; found: 265.3 [M+Na]+ (100%).

H-Pro-NMe₂·TFA

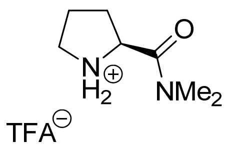

Boc-Pro-NMe$_2$ (337 mg, 1.39 mmol, 1.0 eq) was stirred in a 1:1 mixture of TFA and CH_2Cl_2 (7 ml) for 3 hours. The solution was concentrated under reduced pressure to obtain the title compound (356 mg, quant.) as slightly yellowish oil.

TLC R_f = 0.41 (silica, MeCN:H_2O 4:1, ninhydrin).

^{1}H NMR (400 MHz, $CDCl_3$) δ/ppm = 10.41 (s, 1H, NH_2^+), 7.66 (s, 1H; NH_2^+), 4.92-4.81 (m, 1H; Hα), 3.62-3.44 (m, 2H; Hδ), 3.09 (s, 3H; NMe$_2$), 3.05 (s, 3H; NMe$_2$), 2.63-2.51 (m, 1H; Hβ), 2.25-2.14 (m, 1H; Hβ), 2.13-2.03 (m, 1H; Hγ), 2.02-1.92 (m, 1H; Hγ).

^{13}C NMR (101 MHz, $CDCl_3$) δ/ppm = 168.6 (amide), 58.7 (Cα), 47.5 (Cδ), 37.1 (NMe$_2$), 36.7 (NMe$_2$), 29.8 (Cβ), 25.2 (Cγ).

ESI-MS: *m/z* calcd for $C_7H_{14}N_2O$: 142.1; found: 143.1 [M+H]+ (100%).

Ac-Pro-NMe₂ (1-NMe₂)

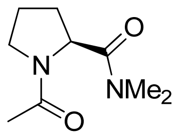

H-Pro-NMe_2·TFA (356 mg, 1.39 mmol, 1.0 eq) was dissolved in 3.8 ml of CH_2Cl_2 and Ac_2O (263 µL, 2.78 mmol, 2.0 eq) and NEt_3 (391 µL, 2.78 mmol, 2.0 eq) were added. The solution was stirred overnight, diluted with CH_2Cl_2 (15 ml), and washed with 1M HCl (3x 5 ml) and sat. $NaHCO_3$ (3 x 5 ml). The organic layer was dried under reduced pressure and the residue was subjected to flash chromatography (silica, 5% MeOH in CH_2Cl_2) to obtain the title compound (150 mg, 59%) as colorless oil.

The analytical data are in agreement with published data.[2]

TLC R*f* = 0.22 (silica, 5% MeOH in CH_2Cl_2, TDM).

^{1}H NMR (400 MHz, Deuterium Oxide); *trans* conformer δ / ppm = 4.85 – 4.73 (Hα, overlapping with DHO), 3.64 (t, *J* = 6.9 Hz, 2H; Hδ), 3.12 (s, 3H; NMe), 2.92 (s, 3H; NMe), 2.32 (dddd, *J* = 12.6, 8.8, 7.9, 7.2 Hz, 1H; Hβ), 2.02 – 1.94 (m, 2H; Hγ), 2.10 (s, Ac), 1.89 – 1.81 (m, 1H; Hβ). Isolated signals of the *cis* conformer δ / ppm = 4.99 (dd, *J* = 8.9, 3.3 Hz, 1H; Hα), 3.53 (ddd, *J* = 8.0, 5.5, 2.0 Hz, 2H; Hδ), 3.14 (s, 3H; NMe), 2.96 (s, 3H; NMe), 2.42 (dddd, *J* = 12.7, 9.8, 8.8, 7.1 Hz, 1H; Hβ), 1.90 (s, 3H; Ac).
^{13}C NMR (101 MHz, Deuterium Oxide); *trans* conformer δ / ppm = 173.3 (carbonyl), 172.4 (carbonyl), 57.3 (Cα), 48.8 (Cδ), 36.9 (NMe), 35.7 (NMe), 28.7 (Cβ), 24.1 (Cγ), 21.2 (Ac). Isolated signals of the *cis* conformer δ / ppm = 59.2 (Cα), 47.2 (Cδ), 36.8 (NMe), 35.9 (NMe), 30.3 (Cβ), 22.4 (Cγ), 21.0 (Ac).
$K_{trans/cis}$ (400 MHz, Deuterium Oxide) = 3.8

^{1}H NMR (400 MHz, DMSO-d6); *trans* conformer δ / ppm = 4.69 (dd, *J* = 8.6, 3.7 Hz, 1H; Hα), 3.54 – 3.45 (m, 2H; Hδ), 3.01 (s, 3H; NMe), 2.79 (s, 3H; NMe), 2.10 (dψtd, *J* = 12.3, 8.6, 7.6 Hz, 1H; Hβ), 1.93 (s, 3H; Ac), 1.95 – 1.83 (m, *J* = 16.1, 6.9 Hz, 2H; Hγ), 1.75 – 1.67 (m, 1H; Hβ). Isolated signals of the *cis* conformer δ / ppm = 4.86 (dd, *J* = 8.7, 3.0 Hz, 1H; Hα), 3.03 (s, 3H; NMe), 2.84 (s, 3H; NMe), 2.25 (dddd, *J* = 12.3, 9.5, 8.7, 7.4 Hz, 1H; Hβ), 1.88 – 1.81 (m, 1H; Hβ), 1.79 – 1.70 (m, 2H; Hγ), 1.73 (s, 3H; Ac).
^{13}C NMR (101 MHz, DMSO-d6); *trans* conformer δ / ppm = 171.18 (carbonyl), 167.70 (carbonyl), 55.78 (Cα), 47.49 (Cδ), 36.48 (NMe), 35.18 (NMe), 28.60 (Cβ), 24.16 (Cγ), 22.20 (Ac). Isolated signals of the *cis* conformer δ / ppm = 171.10 (carbonyl), 168.33 (carbonyl), 57.61 (Cα), 46.19 (Cδ), 36.34 (NMe), 35.40 (NMe), 30.38 (Cβ), 22.29 (Cγ), 21.96 (Ac).
$K_{trans/cis}$ (400 MHz, DMSO-d, Hα) = 2.0

^{1}H NMR (400 MHz, $CDCl_3$) δ/ppm = 4.86 (dd, *J* = 8.2 Hz, 3.6 Hz, 1H; Hα), 3.73 (ddd, *J* = 9.5 Hz, 8.0 Hz, 5.0 Hz, 1H; Hδ), 3.53 (dt, *J* = 9.7 Hz, 7.2 Hz, 1H; Hδ), 3.14 (s, 3H; NMe_2), 2.97 (s, 3H; NMe_2), 2.26-2.10 (m, 2H; Hβ), 2.10 (s, 3H; Ac), 2.02-1.81 (m, 2H; Hγ). Isolated signals of the minor conformer: 4.63 (dd, *J* = 8.6 Hz, 2.6 Hz, 1H; Hα), 3.10 (s, 3H; NMe_2), 3.01 (s, 3H; NMe_2), 1.91 (s, 3H; Ac).
^{13}C NMR (101 MHz, $CDCl_3$) δ/ppm = 172.3, (carbonyl), 172.1, (carbonyl), 57.0 (Cα), 48.6, (Cδ), 37.7 (NMe_2), 36.7 (NMe_2), 29.1 (Cβ), 24.8 (Cγ), 21.4 (Ac).
$K_{trans/cis}$ (400 MHz, Chloroform-d) = 8.8

^{1}H NMR (400 MHz, 1,4-Dioxane-d8); *trans* conformer δ / ppm = 4.80 (dd, *J* = 8.2, 3.4 Hz, 1H; Hα), 3.64 (ddd, *J* = 9.5, 7.9, 4.8 Hz, 1H; Hδ), 3.48 (ddd, *J* = 9.5, 7.2 Hz, 1H; Hδ), 3.09 (s, 3H; NMe), 2.86 (s, 3H; NMe), 2.20 – 1.99 (m, 2H), 1.97 (s, 3H; Ac), 1.94 – 1.77 (m, 2H).
Isolated signals of the *cis* conformer δ / ppm = 4.66 (dd, J = 8.6, 2.8 Hz, 1H; Ha), 2.31 – 2.20 (m, 1H),3.05 (s, 3H; NMe), 2.92 (s, 3H; NMe), 1.76 (s, 3H; Ac).
^{13}C NMR (101 MHz, 1,4-Dioxane-d8); *trans* conformer δ / ppm =172.3 (carbonyl), 169.6 (carbonyl), 56.7 (Cα), 48.4 (Cδ), 37.0 (NMe), 35.8 (NMe), 29.7 (Cβ), 25.3 (Cγ), 21.8 (Ac).
$K_{trans/cis}$ (400 MHz, 1,4-Dioxane-d8) = 6.8

HRMS (ESI): *m/z* calcd for $C_9H_{16}N_2O_2$: 207.1104 $[M+Na]^+$; found: 207.1107 $[M+Na]^+$.

2.3. Ac-(4*R*)Flp-OMe (2R-OMe)

Boc-(4*R*)Flp-OMe

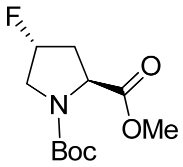

Boc-(4*R*)Flp-OH (415 mg, 1.78 mmol) was suspended in toluene (11.86 mL) and MeOH (3.5 ml) and set under an Ar atmosphere. Trimethylsilyldiazomethane (264.2 mg, 1.3 equiv., 1.16 ml) was added, while the reaction mixture turned yellow and gas formation was observed. The reaction mixture was stirred for 10 min and quenched with AcOH in MeOH. The ester Boc-(4*R*)Flp-OMe was purified by flash chromatography on silica (CH_2Cl_2 → 3% MeOH → 5% MeOH in CH_2Cl_2). The title compound (394 mg, 90%) was isolated in as colorless solid.

TLC R*f* = 0.61 (5% MeOH, ninhydrin).

^{1}H NMR (500 MHz, Chloroform-d) δ / ppm = 5.33 – 5.10 (m, 1H; Hγ), 4.40 (dd, *J* = 9.3, 7.5 Hz, 1H, Hα), 3.91 (ddd, *J* = 22.5, 13.1, 2.4 Hz, 1H; Hδ), 3.74 (s, 3H; OMe), 3.66 (dd, *J* = 13.0, 3.4 Hz, 1H; Hδ), 2.67 – 2.49 (m, 1H; Hβ), 2.20 – 2.00 (m, 1H; Hβ), 1.42 (s, 9H; Boc). Isolated signals of the *minor* conformer δ/ppm= 4.47 (ψt, *J* = 8.3 Hz, 1H; Hα), 3.81 (ddd, *J* = 22.5, 12.9, 2.2 Hz, 1H; Hδ), 3.76 (s, 3H; OMe), 3.58 (dd, *J* = 13.0, 3.4 Hz, 1H; Hδ), 1.46 (s, 9H; Boc).

TFA H-(4*R*)Flp-OMe

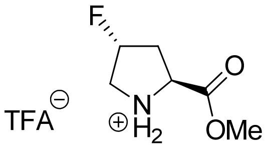

Boc-(4*R*)Flp-OMe (394 mg) was dissolved in TFA:CH_2Cl_2 (1:1, 4 ml) and stirred for 3 hours. The mixture was concentrated under reduced pressure and the residue was suspended in little CH_2Cl_2. Upon addition of Et_2O a the title compound (416 mg, quant.) precipitated as colourless solid that was filtered off.

^{1}H NMR (500 MHz, Deuterium Oxide) δ / ppm = 5.64 – 5.40 (m, 1H; Hγ), 4.78 – 4.70 (m, 1H, Hα), 3.84 (s, 3H; OMe), 3.81 – 3.57 (m, 2H; Hδ), 2.89 – 2.72 (m, 1H; Hβ), 2.53 – 2.26 (m, 1H; Hβ).

Ac-(4*R*)Flp-OMe (2R-OMe)

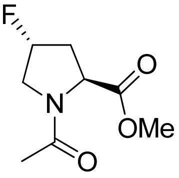

H-(4*R*)Flp-OMe·TFA (394 mg, 1.59 mmol, 1.0 equiv.) was suspended in of CH_2Cl_2 (3.19 ml) and NEt_3 (551 µl, mmol, 2.50 equiv.) was added. After cooling with an ice bath Ac_2O (301 µl, 3.18 mmol, 2.00 equiv.) was added and the reaction mixture was stirred overnight. The reaction mixture was diluted with CH_2Cl_2 (100 ml) and washed with HCl (1M, 3 x 100 ml) and saturated $NaHCO_3$ (3 x 100 ml). The organic layer was dried over $MgSO_4$ and concentrated under reduced pressure. After purification with flash chromatography on silica the title compound (274 mg, 91%) was obtained as colorless oil.

The analytical data are in agreement with published data.[1]

^{1}H NMR (400 MHz, Deuterium Oxide); *trans* conformer δ / ppm = 5.55 – 5.35 (dm, *J* = 51.7 Hz, 1H; Hγ), 4.59 (dd, *J* = 10.0, 7.8 Hz, 1H; Hα), 4.09 – 3.84 (m, 2H; Hδ), 3.79 (s, 3H; OMe), 2.78 – 2.66 (m, 1H; Hβ), 2.35 – 2.13 (m, 4H; Hβ, Ac). Isolated signals of the *cis* conformer δ / ppm = 5.49 – 5.29 (dm, *J* = 52.2 Hz, 1H; Hγ), 4.93 (ψt, J = 8.4 Hz, 1H; Hα), 4.20 – 4.09 (m, 1H; Hδ), 3.84 (s, 3H; OMe), 3.64 – 3.45 (m, 1H; Hδ), 2.94 – 2.79 (m, 1H; Hβ), 2.54 – 2.35 (m, 1H; Hβ), 2.04 (s, 3H; Ac).
^{13}C NMR (101 MHz, Deuterium Oxide); *trans* conformer δ / ppm = 174.3 (carbonyl), 173.3 (carbonyl), 92.8 (d, *J* = 175.2 Hz; Cγ), 57.6 (Cα), 54.5 (d, *J* = 22.3 Hz; Cδ), 53.1 (OMe), 35.6 (d, *J* = 22.1 Hz; Cβ), 21.3 (Ac). Isolated signals of the *cis* conformer δ / ppm = 91.5 (d, *J* = 174.6 Hz; Cγ), 58.5 (Cα), 53.4 (OMe), 52.8 (d, *J* = 22.8 Hz; Cδ), 37.1 (d, *J* = 22.2 Hz; Cβ), 20.8 (Ac).
$K_{trans/cis}$ (400 MHz, Deuterium Oxide) = 6.7

^{1}H NMR (400 MHz, Chloroform-d); *trans* conformer δ / ppm = 5.40 – 5.19 (dm, *J* = 51.6 Hz 1H; Hγ), 4.62 – 4.52 (ψt, *J* = 8.5 Hz, 1H; Hα), 3.89 – 3.76 (m, 2H; Hδ), 3.75 (s, 3H; OMe), 2.67 – 2.53 (m, 1H; Hβ), 2.20 – 2.02 (m, 4H, Hβ, Ac). Isolated signals of the *cis* conformer δ / ppm = 5.32 – 5.13 (m, *J* = 52.5 Hz, 1H; Hγ), 4.23 (dddd, *J* = 21.4, 13.8, 2.6, 1.0 Hz, 1H; Hδ), 3.62 – 3.43 (m, 1H; Hδ), 2.81 – 2.68 (m, 1H; Hβ), 2.37 – 2.20 (m, 1H; Hβ), 1.99 (s, 3H; Ac).
^{13}C NMR (101 MHz, Chloroform-d); *trans* conformer δ / ppm = 172.6 (carbonyl), 169.5 (carbonyl), 92.0 (d, *J* = 180.0 Hz; Cγ), 57.4 (Cα), 54.3 (d, *J* = 22.9 Hz, Cδ), 52.6 (OMe), 36.24 (d, *J* = 22.4 Hz; Cβ), 22.4 (Ac). Isolated signals of the *cis* conformer δ / ppm = 170.0 (carbonyl), 90.4 (d, *J* = 178.9 Hz ; Cγ), 58.4 (Cα), 53.0 (OMe), 38.4 (d, *J* = 23.0 Hz; Cβ), 21.8 (Ac).
$K_{trans/cis}$ (400 MHz, Chloroform-d) = 4.4

HRMS (ESI): *m/z* calcd for $C_8H_{12}FNO_3+H^+$: 190.0874 $[M+H]^+$; found: 190.0872 $[M+H]^+$.

2.4. Ac-(4*R*)Flp-NMe$_2$ (2R-NMe$_2$)

Boc-(4*R*)Flp-NMe$_2$

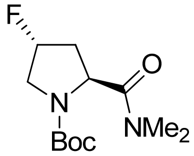

Boc-(4*R*)Flp-OH (250 mg, 1.07 mmol, 1.0 eq) was suspended in CH_2Cl_2 (4.3 ml) and *i*Pr_2NEt (459 µl, 2.68 mmol, 2.5 eq) was added whereupon the suspension turned into a solution. Next EDC·HCl (288 mg, 1.50 mmol, 1.4 eq) and $HNMe_2$·HCl (131 mg, 1.61 mmol, 1.5 eq) were added and the solution was stirred overnight. The solution was diluted with of CH_2Cl_2 (50 ml) and washed with 1M HCl (3 x 20 ml), dried over $MgSO_4$ and concentrated under reduced pressure. The oily residue was subjected to column chromatography on silica (7% MeOH in CH_2Cl_2) to obtain the title compound (173 mg, 62%) as a colourless oil.

TLC R_f = 0.38 (silica, 7% MeOH in CH_2Cl_2, ninhydrin).

(two isomers are visible in the ^{1}H NMR- and ^{13}C NMR-spectra in a ratio of ≈ 1:1 in $CDCl_3$).

^{1}H NMR (400 MHz, $CDCl_3$) δ/ppm = 5.35-5.16 (m, 1H; Hγ), 4.88 (t, *J* = 7.8 Hz, 1H; Hα), 3.92 (ddd, *J* = 22.6 Hz, 13.1 Hz, 2.1 Hz, 1H; Hδ), 3.66 (dt, *J* = 12.9 Hz, 4.0 Hz, 1H; Hδ), 3.17 (s, 3H; NMe_2), 3.01 (s, 3H; NMe_2), 2.54-2.39 (m, 1H; Hβ), 2.25-2.05 (m, 1H; Hβ), 1.47 (s, 9H; Boc). Isolated signal of the other conformer: 4.78 (dd, *J* = 8.4 Hz, 7.7 Hz, 1H; Hα), 3.85 (dd, *J* = 22.4 Hz, 13.0 Hz, 1H; Hδ), 3.75 (ddd, *J* = 12.9 Hz, 6.3 Hz, 3.4 Hz, 1H; Hδ), 3.11 (s, 3H; NMe_2), 2.99 (s, 3H; NMe_2), 1.43 (s, 9H; Boc).
^{19}F NMR (376 MHz, $CDCl_3$) δ/ppm = -176.2 (s), -177.0 (s) (both conformers).
^{13}C NMR (101 MHz, $CDCl_3$) δ/ppm = 172.6 (amide), 154.7 (Boc), 93.3 (d, *J* = 81.3 Hz; Cγ), 80.5 (Boc), 54.7 (Cα), 53.9 (d, *J* = 22.7 Hz; Cδ), 37.9 (NMe_2), 37.7 (d, *J* = 6.5 Hz; Cβ), 37.5 (NMe_2), 28.8 (Boc). Isolated signals the other conformer: 172.5 (amide), 91.6 (d, *J* = 80.7 Hz; Cγ), 80.5 (Boc), 54.7 (Cα), 53.6 (d, *J* = 21.8 Hz; Cδ), 37.1 (NMe_2), 36.9 (NMe_2), 36.5 (d, *J* = 5.6 Hz; Cβ), 28.7 (Boc).

ESI-MS: *m/z* calcd for $C_{12}H_{21}FN_2O_3$: 260.2; found: 283.3 $[M+Na]^+$ (100%).

H-(4*R*)Flp-NMe$_2$·HCl

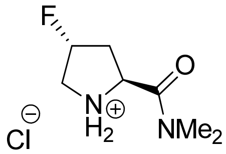

Boc-(4*R*)Flp-NMe$_2$ (173 mg, 665 µmol, 1.0 eq) was dissolved in HCl (1.7 ml, 4M in dioxane) and stirred for 3 hours. The solution was concentrated under reduced pressure to obtain the title compound (180 mg, quant.) as slightly yellowish oil.

TLC R*f* = 0.43 (silica, MeCN:H_2O 4:1, ninhydrin).

^{1}H NMR (400 MHz, D_2O) δ/ppm = 5.49 (dt, *J* = 50.8 Hz, 3.3 Hz, 1H; Cγ), 4.93 (dd, *J* = 10.2 Hz, 8.0 Hz, 1H; Cα), 3.72 (ddd, *J* = 19.1 Hz, 13.7 Hz, 2.2 Hz, 1H; Cδ), 3.55 (ddd, *J* = 37.7 Hz, 13.7 Hz, 3.0 Hz, 1H; Cδ), 3.01 (s, 3H; NMe$_2$), 2.91 (s, 3H; NMe$_2$), 2.94-2.81 (m, 1H; Hβ), 2.16 (dddd, *J* = 15.0 Hz, 14.4 Hz, 10.3 Hz, 3.8 Hz, 1H).

^{13}C NMR (101 MHz, D_2O) δ/ppm = 168.4 (amide), 93.3 (d, *J* = 175.7 Hz; Cγ), 57.8 (Cα), 52.5 (d, *J* = 23.0 Hz; Cδ), 37.0 (NMe$_2$), 36.3 (NMe$_2$), 36.0 (d, *J* = 22.0 Hz; Cβ).

ESI-MS: *m/z* calcd for $C_7H_{14}ClFN_2O$: 196.1; found: 161.1 $[M-Cl]^+$ (100%).

Ac-(4*R*)Flp-NMe$_2$ (2R-NMe$_2$)

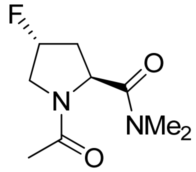

H-(4*R*)Flp-NMe$_2$·HCl (180 mg, 914 µmol, 1.0 eq) was suspended in CH_2Cl_2 (3 ml) and NEt_3 (380 µl, 2.74 mmol, 3.0 eq) was added. After cooling with an ice bath Ac_2O (242 µl, 2.56 mmol, 2.8 eq) was added and the reaction mixture was stirred overnight. The reaction mixture was concentrated under reduced pressure, diluted with dioxane: water 1:1 and concentrated again, to remove residual Ac_2O. AcOH was removed by coevaporation with toluene. The residue was purified by ion exchange chromoatography over Amberlite IR-120H ion exchange resin to remove NEt_3. The title compound (105 mg, 57 %) was obtained as colourless oil.

TLC R_f = 0.40 (10% MeOH in CH_2Cl_2, TDM or Vanillin).

HRMS (ESI): *m/z* calcd for $C_9H_{15}FN_2O_2+H^+$: 203.1190 $[M+H]^+$; found: 203.1189 $[M+H]^+$.

^{1}H NMR (400 MHz, Deuterium Oxide); *trans* conformer δ / ppm = 5.55 – 5.36 (dm, *J* = 52.0 Hz, 1H; Hγ), 5.08 – 4.97 (dψd, *J* = 9.3, 1.5 Hz, 1H; Hα), 4.05 (ddd, J = 21.6, 13.0, 2.3 Hz, 1H; Hδ), 3.87 (ddd, J = 38.4, 13.1, 3.1 Hz, 1H; Hδ), 3.18 (s, 3H; NMe), 2.97 (s, 3H; NMe), 2.81 – 2.68 (m, 1H; Hβ), 2.14 (s, 3H; Ac), 2.18 – 1.96 (m, *J* = 14.8, 9.7, 3.8, 0.7 Hz, 1H; Hβ). Isolated signals of the *cis* conformer δ / ppm = 5.50 – 5.29 (dm, *J* = 51.9 Hz, 1H; Hγ), 5.26 (ψt, *J* = 8.4 Hz, 1H; Hα), 4.20 – 4.09 (m, 1H; Hδ), 3.56 (dddd, *J* = 38.1, 13.9, 3.2, 0.7 Hz, 1H; Hδ), 3.20 (s, 3H; NMe), 3.01 (s, 3H; NMe), 2.33 – 2.20 (m, 1H; Hβ), 1.95 (s, 3H; Ac).

^{13}C NMR (101 MHz, Deuterium Oxide); *trans* conformer δ / ppm = 172.7 (carbonyl), 172.6 (carbonyl), 92.9 (d, *J* = 174.8 Hz, Cγ), 55.4 (Cα), 54.8 (d, *J* = 22.1 Hz; Cδ), 37.0 (NMe), 35.9 (NMe), 35.1 (d, *J* = 22.0 Hz; Cβ), 21.4 (Ac). Isolated signals of the *cis* conformer δ / ppm = 57.0 (Cα), 53.3 (d, *J* = 22.4 Hz; Cδ), 36.7 (d, *J* = 22.5 Hz; Cβ), 36.8 (NMe), 36.1 (NMe), 20.7 (Ac).

$K_{trans/cis}$ (400 MHz, Deuterium Oxide) = 4.5

^{1}H NMR (600 MHz, Chloroform-d); *trans* conformer δ / ppm = 5.40 – 5.27 (dm, *J* = 53.0 Hz, 1H; Hγ), 4.98 (t, *J* = 7.9 Hz, 1H; Hα), 3.88 (ddd, *J* = 34.7, 12.1, 3.4 Hz, 1H; Hδ), 3.81 (dddd, *J* = 21.4, 12.2, 2.1, 1.1 Hz, 1H; Hδ), 3.18 (s, 3H; NMe), 2.96 (s, 3H; NMe), 2.45 (dddt, *J* = 21.0, 14.3, 7.9, 1.8 Hz, 1H; Hβ), 2.27 – 2.14 (m, 1H; Hβ), 2.08 (s, 3H; Ac). Isolated signals of the *cis* conformer δ / ppm = 5.28 – 5.17 (m, 1H; Hγ), 4.85 (t, *J* = 8.1 Hz, 1H; Hα), 4.23 (ddd, *J* = 21.8, 14.0, 2.1 Hz, 1H; Hδ), 3.60 (ddd, *J* = 37.1, 13.8, 3.4 Hz, 1H; Hδ), 3.11 (s, 3H; NMe), 3.01 (s, 3H; NMe), 2.74 – 2.64 (m, 1H; Hβ), 1.91 (s, 3H; Ac).

^{13}C NMR (151 MHz, Chloroform-d); *trans* conformer δ / ppm = 171.9 (carbonyl), 169.4 (carbonyl), 92.6 (d, *J* = 178.9 Hz; Cγ), 54.6 (d, *J* = 22.6 Hz; Cδ), 54.4 (Cα), 37.6 (NMe), 36.3 (d, *J* = 21.9 Hz; Cβ), 36.2 (NMe) 22.49 (Ac). Isolated signals of the *cis* conformer δ / ppm = 171.1 (carbonyl), 169.9 (carbonyl), 90.8 (d, *J* = 177.9 Hz; Cγ), 56.34 (Cα), 53.2 (d, *J* = 22.5 Hz; Hδ), 38.2 (d, *J* = 22.7 Hz; Cβ), 36.9 (NMe), 21.9 (Ac).

Noesy (600 MHz, Chloroform-d) cross peak 4.89 ppm (Hα minor) and 1.91 ppm (Ac minor) → *cis* conformer.

$\mathbf{K_{trans/cis}}$ (600 MHz, Chloroform-d) = 9.8

2.5. Ac-(4*R*)Azp-NMe$_2$ (3R-NMe$_2$)

Ac-(4*R*)Azp-OH

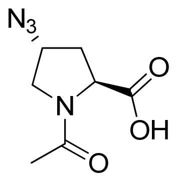

300 mg (1.41 mmol) Ac-(4*R*)Azp-OMe (300 mg, 1.41 mmol) were dissolved in THF (7 ml) and diluted with MeOH (7 ml). NaOH (85 mg, 2.1 mmol in 1.4 ml H_2O) was added to the solution. The reaction mixture was stirred at room temperatur for 2 h. The mixture was neutralized with HCl (1 M) and the solvent volume was reduced under reduced pressure. The remaining solution was diluted with CH_2Cl_2 and washed with HCl (1M, 30 ml). The aqueous layer was washed with CH_2Cl_2 (4 times), the combined organic layers were dried over Na_2SO_4 and concentrated under reduced pressure. The title compound (281 mg, quant.) was obtained as colorless solid.

Two isomers are visible in the ^{1}H NMR- and ^{13}C NMR-spectra in a ratio of 1:4 in D_2O.

^{1}H-NMR (400 MHz, Deuterium Oxide): δ / ppm (*major*) = 4.39-4.32 (m, 2H; Hα, Hγ), 3.75 (dd, *J* = 11.7 Hz, *J* = 4.9 Hz, 1H; Hδ), 3.63-3.56 (m, 1H; Hδ), 2.39 (dddd, *J* = 13.3 Hz, 8.1 Hz, 3.4 Hz, 1.5 Hz, 1H; Hβ), 2.32 (ddd, *J* = 13.7 Hz, 8.3 Hz, 5.4 Hz, 1H; Hβ), 2.00 (s, 3H; Ac). Isolated signals of the *minor* conformer δ / ppm = 4.63 (dd, *J* = 8.3 Hz, 6.9 Hz, 1H; Hα), 4.33 (m, 1H; Hγ), 3.58-3.52 (m, 1H; Hδ), 3.41 (dd, *J* = 12.6 Hz, 5.4 Hz, 1H; Hδ), 2.19 (dddd, *J* = 13.7 Hz, 8.5 Hz, 4.4 Hz, 1.5 Hz, 1H; Hβ), 2.19 (ddd, *J* = 13.8 Hz, 6.7 Hz, 5.6 Hz, 1H; Hβ), 1.89 (s, 3H; Ac).

^{13}C-NMR (101 MHz, D2O+0.07 M NaOD, 25°C): δ / ppm (both conformers) = 179.1, 179.0, 174.0, 172.9, 61.9, 60.4, 60.2, 58.9, 53.9, 51.7, 37.0, 35.0, 21.9, 21.1.

MS (ESI, neg): *m/z* (%) = 395.3 [2M-H]$^-$ (100), 197.2 [M-H]$^-$ (25), calculated for $C_7H_{10}N_4O_3$.

Ac-(4*R*)Azp-NMe$_2$ (3R-NMe$_2$)

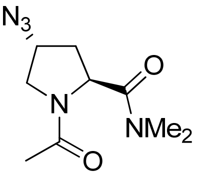

Ac-(4*S*)Azp-OH (30 mg, 0.152 mmol) was dissolved in CH_2Cl_2 (500 µl) and pentafluorophenol (29 mg, 0.158 mmol) and EDC (43 mg, 0.225 mmol) were added. The reaction mixture was stirred at room temperature for 1 h, diluted with CH_2Cl_2, washed with HCl (0.5M) and the aqueous phase was extracted with CH_2Cl_2. The combined organic layers were dried over Na_2SO_4 and concentrated under reduced pressure. The oily pentafluorophenylesters residue (55 mg, 0.151 mmol, 99%) was dissolved in dry CH_2Cl_2 (1 ml). NEt3 (213 µl, 1.53 mmol) and dimethylamide hydrochloride (114 mg, 1.40 mmol) were added and the reaction mixture was stirred at room temperature for 1h. The mixture was diluted with CH_2Cl_2 and washed with HCl (1M). The combined organic layers were dried over Na_2SO_4 and concentrated under reduced pressure. The title compound (25 mg, 78%) was purified by flash chromatography on silica (4% - 6% MeOH in CH_2Cl_2) and isolated as colorless oil.

^{1}H NMR (400 MHz, Deuterium Oxide); *trans* conformer δ / ppm = 4.80 (ψt, *J* = 8.1 Hz, 1H; Hα), 4.33 (m, 1H; Hγ), 3.73 (dd, *J* = 11.8, 4.8 Hz, 1H; Hδ), 3.63 (dt, *J* = 11.8, 2 Hz, 1H; Hδ), 3.03 (s, 3H; NMe), 2.82 (s, 3H; NMe), 2.39 (dddd, *J* = 13.5, 8.0, 3.0, 1.4 Hz, 1H; Hβ), 1.99 (m, 1H; Hβ), 1.99 (s, 3H; Ac). Isolated signals of the *cis* conformer δ / ppm = 5.03 (dd, *J* = 7.7, 1H; Hα), 4.25 (m, 1H; Hγ), 3.67 (dt, *J* = 13.0, 2.0 Hz, 1H; Hδ), 3.48 (dd, *J* = 12.6, 5.1 Hz, 1H; Hδ), 3.04 (s, 3H; NMe), 2.86 (s, 3H; NMe), 2.53 (dddd, *J* = 13.6, 8.2, 3.6, 1.6 Hz, 1H; Hβ), 2.12 (ddd, *J* = 13.7, 7.2, 5.7 Hz, 1H; Hβ), 1.79 (s, 3H; Ac).
^{13}C NMR (101 MHz, Deuterium Oxide); *trans* conformer: δ / ppm = 173.0, 172.8, 60.3, 56.0, 53.8, 37.4, 36.3, 34.3, 21.7. *cis* conformer 173.8, 172.7, 58.7, 57.7, 52.0, 37.2, 36.4, 35.9, 21.0.
$K_{trans/cis}$ (400 MHz, Deuterium Oxide) = 3.8

^{1}H NMR (400 MHz, Chloroform-d); *trans* conformer δ / ppm = 4.93 (dd, *J* = 7.7, 6.1 Hz, 1H; Hα), 4.45 (qd, *J* = 5.6, 3.8 Hz, 1H; Hγ), 3.94 (dd, *J* = 10.7, 5.8 Hz, 1H; Hδ), 3.50 (ddd, *J* = 10.8, 3.9, 0.7 Hz, 1H ; Hδ), 3.18 (s, 3H; NMe), 2.97 (s, 3H; NMe), 2.28 – 2.15 (m, 2H, Hβ), 2.08 (s, 3H; Ac). Isolated signals of the *cis* conformer δ / ppm = 4.76 (dd, *J* = 8.2, 6.1 Hz, 1H; Hα), 4.24 (m, *J* = 5.2 Hz, 1H; Hγ), 3.90 – 3.83 (m, 1H; Hδ), 3.77 (dd, *J* = 12.4, 5.5 Hz, 1H; Hδ), 3.11 (s, 3H; NMe), 3.02 (s, 3H; NMe), 2.42 (dddd, *J* = 13.0, 8.3, 4.6, 1.5 Hz, 1H; Hβ), 1.90 (s, 3H; Ac).
^{13}C NMR (101 MHz, Chloroform-d); *trans* conformer δ / ppm = 171.6, 169.2, 77.2, 60.0, 54.6, 52.9, 37.5, 36.2, 34.9, 22.4.
$K_{trans/cis}$ (400 MHz, Chloroform-d) = 9.6
MS (ESI): *m/z* (%) = 248.0 $[M+Na]^+$(100), 473.1 $[2M+Na]^+$(60) calcd. for $C_9H_{15}N_5O_2$.

2.6. Ac-(4*S*)Flp-OMe (2S-OMe)

Ac-(4*S*)Flp-OMe (2S-OMe)

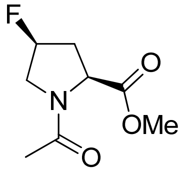

Boc-(4*S*)Flp-OH (250 mg, 1.07 mmol) dissolved in MeOH (25 ml) and cooled in an ice bath before acetyl chloride (25 ml) was added slowly. The reaction mixture was stirred at room temperature overnight. The solution was concentrated under reduced pressure and the residue was dissolved in CH_2Cl_2 (38 ml). DMAP (1.13 g, 9.25 mmol, 8.6 equiv.) and acetyl chloride (224 µl, 3.1 mmol, 2.9 equiv.) were added and the reaction mixture was stirred at room temperature overnight.
The solution was quenched with MeOH and concentrated under reduced pressure. The residue was dissolved in citric acid (20 w-%, 200 ml) and extracted with CH_2Cl_2 (3 x 100 ml). The combined organic layers were dried over $MgSO_4$, concentrated under reduced pressure and purified by flash chromatography on silica (5% MeOH in CH_2Cl_2). The title compound (106 mg, 52%) was obtained as colourless oil.

The analytical data are in agreement with published data.[1]

TLC R*f* = 0.41 (silica, 5% MeOH in CH_2Cl_2, vanillin).

^{1}H NMR (600 MHz, Deuterium Oxide); *trans* conformer δ / ppm = 5.51 – 5.37 (dm, *J* = 52.0 Hz, 1H ; Hγ), 4.76 (dd, *J* = 8.8, 2.9 Hz, 1H ; Hα), 4.04 – 3.86 (m, 2H ; Hδ), 3.79 (br.s, 3H; OMe), 2.76 – 2.47 (m, 2H; Hβ), 2.16 (s, 3H ; Ac). Isolated signals of the *cis* conformer δ / ppm = 5.47 – 5.34 (dm, *J* = 52.4 Hz, 1H; Hγ), 4.95 – 4.90 (d, *J* = 9.3 Hz, 1H; Hα), 3.82 (br. s, 3H; OMe), 2.08 (s, 3H; Ac).

^{13}C NMR (151 MHz, Deuterium Oxide); *trans* conformer δ / ppm = 173.8 (carbonyl), 173.4 (carbonyl), 93.4 (d, *J* = 173.0 Hz; Cγ), 57.4 (Cα), 54.5 (d, *J* = 23.1 Hz; Cδ), 53.1 (OMe), 35.5 (d, *J* = 21.1 Hz; Cβ), 21.3 (Ac).

Noesy (600 MHz, Deuterium Oxide) cross peak δ / ppm = 2.09 Ac (minor) and 4.9 (Hα minor)→ *cis* conformer.

$K_{trans/cis}$ (600 MHz, Deuterium Oxide) = 2.6

^{1}H NMR (600 MHz, Chloroform-d); *trans* conformer δ / ppm = 5.29 (dtt, *J* = 52.5, 4.2, 1.1 Hz, 1H; Hγ), 4.81 – 4.72 (d, *J* = 9.8 Hz, 1H; Hα), 3.94 – 3.71 (m, 5H-m; Hδ, OMe), 2.56 – 2.24 (m, 2H; Hβ), 2.11 (s, 3H; Ac). Isolated signals of the *cis* conformer δ / ppm = 5.29 – 5.17 (dm, *J* = 52.4 Hz, 1H; Hγ), 4.50 (d, *J* = 9.4 Hz, 1H; Hα), 2.75 – 2.67 (m, 1H; Hβ), 2.05 (s, 3H; Ac).

^{13}C NMR (151 MHz, Chloroform-d); *trans* conformer δ / ppm = 171.3 (carbonyl), 169.6 (carbonyl), 92.3 (d, *J* = 179.0 Hz; Cγ), 57.1 (Cα), 54.3 (d, *J* = 24.7 Hz; Cδ), 52.6 (OMe), 36.3 (d, *J* = 21.7 Hz; Cβ), 22.4 (Ac). Isolated signals of the *cis* conformer δ / ppm = 171.5 (carbonyl), 170.0 (carbonyl), 90.9 (d, *J* = 175.8 Hz; Cγ), 58.8 (Cα), 53.2 (d, *J* = 24.2 Hz; Cδ), 53.0 (OMe), 38.3 (d, *J* = 21.8 Hz; Cβ), 22.2 (Ac).

Noesy (600 MHz, Chloroform-d) cross peak δ / ppm = 2.1 (Ac minor) and 4.5 (Hα minor) → *cis* conformer.

$K_{trans/cis}$ (400 MHz, Chloroform-d) = 1.7

HRMS (ESI): *m/z* calcd for $C_8H_{12}FNO_3+H^+$: 190.0874 $[M+H]^+$; found: 190.0876 $[M+H]^+$.

2.7. Ac-(4*S*)Flp-NMe₂ (2S-NMe₂)

Boc-(4*S*)Flp-NMe₂

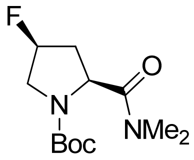

Boc-(4*S*)Flp-OH (250 mg, 1.07 mmol, 1.0 eq) was converted to the title compound according the protocol for its (4*R*)-configured diastereoisomer. The title compound (195 mg, 70 %) was obtaines as colourless oil.

TLC R*f* = 0.39 (silica, 7% MeOH in CH_2Cl_2, ninhydrin).

(two isomers are visible in the 1H NMR- and 13C NMR-spectra in a ratio of ≈ 2:3 in $CDCl_3$).

^{1}H NMR (400 MHz, $CDCl_3$) δ/ppm = 5.31-5.13 (m, 1H; Hγ), 4.76 (dd, *J* = 9.6 Hz, 2.1 Hz, 1H; Hα), 3.96-3.71 (m, 2H; Hδ), 3.05 (s, 3H; NMe_2), 2.99 (s, 3H; NMe_2), 2.60-2.37 (m, 1H; Hβ), 2.31-2.16 (m, 1H; Hβ), 1.48 (s, 9H; Boc). Isolated signals of the minor conformer: 4.63 (dd, *J* = 9.5 Hz, 2.9 Hz, 1H; Hα), 3.00 (s, 3H; NMe_2), 1.42 (s, 9H; Boc).

^{13}C NMR (101 MHz, $CDCl_3$) δ/ppm = 172.6 (amide), 154.7 (Boc), 93.3 (d, *J* = 81.3 Hz; Cγ), 80.5 (Boc), 54.7 (Cα), 53.9 (d, *J* = 22.7 Hz; Cδ), 37.9 (NMe_2), 37.7 (d, *J* = 6.5 Hz; Cβ), 37.5 (NMe_2),28.8 (Boc). Isolated signals the other conformer: 172.5 (amide), 91.6 (d, *J* = 80.7 Hz; Cγ), 80.5 (Boc), 54.7 (Cα), 53.6 (d, *J* = 21.8 Hz; Cδ), 37.1 (NMe_2), 36.9 (NMe_2), 36.5 (d, *J* = 5.6 Hz; Cβ), 28.7 (Boc).

ESI-MS: *m/z* calcd for $C_{12}H_{21}FN_2O_3$: 260.2; found: 283.3 $[M+Na]^+$ (100%).

H-(4*S*)Flp-NMe$_2$·HCl

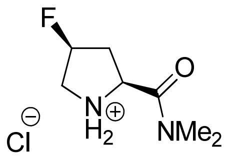

Boc-(4*S*)Flp-NMe$_2$ (195 mg, 749 µmol, 1.0 eq) was dissolved in 4M HCl in dioxane and stirred for 2.5 hours. The mixture was concentrated under reduced pressure and the residue was suspended in little CH_2Cl_2 and dissolved in as little MeOH as possible. Upon addition of Et_2O the tile compound precipitated as colorless solid (147 mg, quant.) and filtered off.

TLC R*f* = 0.20 (silica, 10% MeOH in CH_2Cl_2, ninhydrin).

^{1}H NMR (400 MHz, $CDCl_3$) δ/ppm = 5.40 (dt, *J* = 50.9 Hz, 3.7 Hz, 1H; Hγ), 4.80 (dd, *J* = 11.1 Hz, 3.9 Hz, 1H; Hα), 3.81 (ddd, *J* = 17.7 Hz, 13.6 Hz, 2.3 Hz, 1H; Hδ), 3.46 (ddd, *J* = 37.6 Hz, 13.6 Hz, 3.2 Hz, 1H; Hδ), 2.96 (s, 1H; NMe$_2$), 2.89 (s, 1H; NMe$_2$), 2.78 (dddd, *J* = 40.7 Hz, 15.5 Hz, 11.1 Hz, 4.4 Hz, 1H; Hβ), 2.44-2.30 (m, 1H; Hβ).
^{19}F NMR (376 MHz, D_2O) δ/ppm = -175.0 (ddddd, *J* = 50.9 Hz, 40.9 Hz, 37.7 Hz, 23.3 Hz, 17.8 Hz).
^{13}C NMR (101 MHz, D_2O) δ/ppm = 168.7 (amide), 92.2 (d, *J* = 175.6 Hz; Cγ), 57.8 (Cα), 52.6 (d, *J* = 23.0 Hz; Cδ), 36.9 (NMe$_2$), 36.2 (NMe$_2$), 36.0 (d, *J* = 22.1 Hz; Cβ).

ESI-MS: *m/z* calcd for $C_7H_{14}ClFN_2O$: 196.1; found: 161.1 $[M-Cl]^+$ (100%).

Ac-(4*S*)Flp-NMe$_2$ (2S-NMe$_2$)

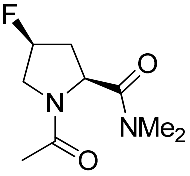

H-(4*S*)Flp-NMe$_2$·HCl (120 mg, 610 µmol, 1.0 eq) was acetylated according the protocol for the preparation of its (4*R*)-configured diastereoisomer to obtain the title compound (66.6 mg, 54%) as colourless solid.

TLC R_f = 0.42 (10% MeOH in CH_2Cl_2, TDM or Vanillin).

^{1}H NMR (400 MHz, Deuterium Oxide); *trans* conformer δ / ppm = 5.42 (dψtψt, *J* = 52.7, 4.1, 1.0 Hz, 1H; Hγ), 5.00 (d, *J* = 10.4 Hz, 1H; Hα), 4.08 – 3.88 (m, 2H; Hδ), 3.12 (s, 3H; NMe), 2.96 (s, 3H; NMe), 2.66 (dddd, *J* = 44.1, 14.8, 10.4, 4.1 Hz, 1H; Hβ), 2.35 (ddtd, *J* = 20.9, 15.2, 1.8, 1.1 Hz, 1H; Hβ), 2.14 (s, 3H; Ac). Isolated signals of the *cis* conformer δ / ppm = 5.47 – 5.29 (dm, *J* = 52.5 Hz, 1H; Hγ), 5.14 (d, *J* = 10.0 Hz, 1H; Hα), 3.88 – 3.71 (m, 2H; Hδ), 3.14 (s, 3H; NMe), 2.99 (s, 3H; NMe), 2.81 (ddd, *J* = 15.3, 10.1, 4.0 Hz, 1H; Hβ), 2.51 (ddddd, *J* = 19.6, 15.1, 2.1, 1.0 Hz, 1H; Hβ), 1.97 (s, 3H; Ac).
^{13}C NMR (101 MHz, Deuterium Oxide); *trans* conformer δ / ppm = 172.9 (carbonyl), 172.0 (carbonyl), 93.1 (d, *J* = 174.7 Hz; Cγ), 56.6 (Cα), 54.9 (d, *J* = 23.4 Hz; Cδ), 36.7 (NMe), 35.8 (NMe), 35.4 (d, *J* = 21.4 Hz; Cβ), 21.3 (Ac). Isolated signals of the *cis* conformer δ / ppm = 173.5 (carbonyl), 172.1 (carbonyl), 91.8 (d, *J* = 173.1 Hz; Cγ), 58.4 (Cα), 53.8 (d, *J* = 23.6 Hz; Cδ), 37.1 (d, *J* = 21.4 Hz; Cβ), 36.8 (NMe), 35.9 (NMe), 21.1 (Ac).
$K_{trans/cis}$ (400 MHz, Deuterium Oxide, Hγ, Ac) = 2.6

^{1}H NMR (400 MHz, Chloroform-d); *trans* conformer δ / ppm = 5.29 (dtt, *J* = 53.9, 5.4, 2.6 Hz, 1H, Hγ), 4.96 (dd, *J* = 9.5, 2.9 Hz, 1H; Hα), 4.05 – 3.80 (m, 2H; Hδ), 3.10 – 2.93 (m, 6H; NMe, NMe), 2.46 (dddd, *J* = 31.9, 14.8, 9.5, 5.5 Hz, 1H; Hβ), 2.32 – 2.19 (m, 1H; Hβ), 2.10 (s, 3H; Ac). Isolated signals of the *cis* conformer δ / ppm = 5.33 – 5.13 (dm, *J* = 53.4 Hz, 1H; Hγ), 4.69 (d, *J* = 9.9 Hz, 1H; Hα), 2.64 (dddd, *J* = 9.6, 4.7 Hz, 1H; Hβ), 1.95 (s, 3H; Ac).
^{13}C NMR (101 MHz, Chloroform-d); *trans* conformer δ / ppm = 170.1 (carbonyl), 169.5 (carbonyl), 91.5 (d, *J* = 182.0 Hz; Cγ), 55.6 (Cα), 54.2 (d, *J* = 26.1 Hz; Hδ), 37.1 (NMe), 36.2(NMe), 35.7 (d, *J* = 21.6 Hz; Cβ), 22.4 (Ac). Isolated signals of the *cis* conformer δ / ppm = 90.4 (d, *J* = 178.9 Hz; Cγ), 58.1 (Cα), 38.0 (d, *J* = 22.2 Hz; Cβ), 22.1 (Ac).
^{19}F NMR (565 MHz, Chloroform-d) δ/ppm = -170.96 (s), -172.54 (s).

^{1}**H{19F} NMR** (600 MHz, Chloroform-d) δ / ppm = 5.29 (dq, *J* = 7.7 Hz, 2.5 Hz, 1H; Hγ), 4.95 (dd, *J* = 9.5 Hz, 2.5 Hz, 1H; Hα), 3.95 (d, *J* = 11.6 Hz, 1H; Hδ), 3.89 (dd, *J* = 11.9 Hz, 5.4 Hz, 1H; Hδ), 3.07 (s, 3H; NMe_2), 2.97 (s, 3H; NMe_2), 2.46 (ddd, *J* = 14.8 Hz, 9.6 Hz, 5.6 Hz, 1H; Hβ), 2.24 (d, *J* = 14.3 Hz, 1H; Hβ), 2.09 (s, 3H; Ac).
$\mathbf{K_{trans/cis}}$ (400 MHz, Chloroform-d) = 4.3

HRMS (ESI): *m/z* calcd for $C_9H_{15}FN_2O_2+H^+$: 203.1190 $[M+H]^+$; found: 203.1189 $[M+H]^+$.

2.8. Ac-(4*S*)Azp-NMe$_2$ (3S-NMe$_2$)

Ac-(4*S*)Azp-OH

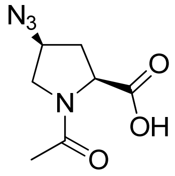

Ac-(4*S*)Azp-OMe (300 mg, 1.41 mmol) were dissolved in THF (7 ml) and diluted with MeOH (7 ml). NaOH (85 mg, 2.1 mmol in 1.4 ml H_2O) were added and the reaction mixture was stirred at room temperature for 3 h. The mixture was diluted with CH_2Cl_2 and washed with HCl (1 M, 30 ml) and brine (30 ml). The combined organic layers were dried over Na_2SO_4 and concentrated under reduced pressure. The title compound (1.28 mmol , 91 %) was obtained as colorless oil.

Two isomers are visible in the ^{1}H NMR- and ^{13}C NMR-spectra in a ratio of 1:2.1 in D_2O.

^{1}H-NMR (400 MHz, Deuterium Oxide): δ / ppm (*major*) = 4.49 (dd, *J* = 9.7 Hz, 2.3 Hz 1H; Hα), 4.37 (m, 1H; Hγ), 3.74 (dd, *J* = 11.8 Hz, 5.3 Hz, 1H; Hδ), 3.58-3.52 (m, 1H; Hδ), 2.50-2.35 (m, 1H; Hβ), 2.19 (ddd, *J* = 14.2 Hz, 3.7 Hz, 2.4 Hz, 1H; Hβ), 1.20 (s, 3H; Ac). Isolated signals of the *minor* conformer δ / ppm = 4.63 (dd, *J* = 8.9 Hz, 1.6 Hz, 1H; Hα), 4.33 (m, 1H; Hγ), 3.58-3.52 (m, 1H; Hδ), 3.41 (bd, *J* = 13.2 Hz, 1H; Hδ), 2.52-2.35 (m, 2H; Hβ), 1.93 (s, 3H; Ac).

^{13}C-NMR (101 MHz, $CDCl_3$+CD_3OD): δ / ppm (*major*) = 172.3, 170.5, 59.1, 57.3, 52.5, 34.1, 22.0. Isolated signals of the *minor* conformer δ / ppm = 172.6, 170.5, 58.6, 58.0, 51.2, 36.4, 21.7.

MS (ESI, neg): m/z (%) = 395.2 [2M-H]$^-$ (100), 197.3 [M-H]$^-$ (55), calculated for $C_7H_{10}N_4O_3$.

Ac-(4*S*)Azp-NMe$_2$ (3S-NMe$_2$)

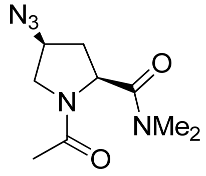

Ac-(4*S*)Azp-OH (30 mg, 0.15 mmol) was dissolved in CH_2Cl_2 (500 µl) and pentafluorophenol (29 mg, 0.16 mmol) and EDC (43 mg, 0.225 mmol) were added to the solution. The reaction mixture was stirred for 1h at room temperature, diluted with CH_2Cl_2 and washed with HCl (0.5M). The aqueous phase was extracted with CH_2Cl_2, dried over Na_2SO_4 and concentrated under reduced pressure to obtain the pentafluorphenylester as viscous oil (55 mg, 0.151 mmol, 99 %). The pentafluorphenylester (51 mg, 0.14 mmol) was dissoleved in dry CH_2Cl_2 and treated with NEt_3 (213 µl, 1.53 mmol) and dimethylamide hydrochloride (114 mg, 1.40 mmol). The reaction mixture was stirred for 1h at room temperature, diluted with CH_2Cl_2 and washed with HCl (1M). The organic phase was dried over Na_2SO_4 and the solvent was removed under reduced pressure. The title compound (25 mg, 78%) was purified by flash chromatography on silica (4% → 6% MeOH in CH_2Cl_2) and obtained as colourless oil.

^{1}H NMR (400 MHz, Deuterium Oxide); *trans* conformer δ / ppm = 4.77 (ψt, *J* = 9.6, 4.5 Hz, 1H; Hα), 4.31 (m, 1H; Hγ), 3.84 (dd, *J* = 11.5, 6.0 Hz, 1H; Hδ), 3.52 (dd, *J* = 11.5, 3.9 Hz, 1H; Hδ), 2.97 (s, 3H; NMe), 2.82 (s, 3H; NMe), 2.58 (ddd, *J* = 13.9, 9.5, 6.1 Hz, 1H; Hβ), 1.91 (dt, *J* = 13.4, 4.0 Hz, 1H; Hβ), 1.99 (s, 3H; Ac). Isolated signals of the *cis* conformer δ / ppm = 4.94 (dd, *J* = 9.5, 2.3 Hz, 1H; Hα), 4.31 (m, 1H; Hγ), 3.69 (dd, *J* = 13.1, 5.9 Hz, 1H; Hδ), 3.44 (bd, *J* = 13.1 Hz, 1H; Hδ), 3.00 (s, 3H; NMe), 2.87 (s, 3H; NMe), 2.66 (ddd, *J* = 14.1, 9.5, 5.9, 1H; Hβ), 2.11 (dm, *J* = 14.3 Hz, 1H; Hβ), 1.80 (s, 3H; Ac).
^{13}C NMR (101 MHz, Deuterium Oxide); *trans* conformer: δ / ppm = 170.6, 168.9, 58.9, 54.7, 52.1, 37.1, 36.0, 34.1, 22.3.
$K_{trans/cis}$ (400 MHz, Deuterium Oxide) = 3.0

^{1}H NMR (400 MHz, Chloroform-d); *trans* conformer δ / ppm = 4.81 (dd, *J* = 8.3, 7.2 Hz, 1H; Hα), 4.18 – 4.05 (m, 1H; Hγ), 3.88 (dd, *J* = 10.3, 7.1 Hz, 1H; Hδ), 3.57 (dd, *J* = 10.3, 8.1 Hz, 1H; Hδ), 3.10 (s, 3H; NMe), 2.96 (s, 3H; NMe), 2.55 (dddd, *J* = 12.9, 8.1, 7.2, 0.8 Hz, 1H; Hβ), 2.07 (s, 3H; Ac), 2.03 – 1.92 (m, 1H; Hβ). Isolated signals of the *cis* conformer δ / ppm = 4.62 (dd, *J* = 8.9, 4.9 Hz, 1H; Hα), 3.06 (s, 3H; NMe), 3.01 (s, 3H; NMe), 2.74 – 2.64 (m, 1H; Hβ), 1.88 (s, 3H; Ac).
^{13}C NMR (101 MHz, Chloroform-d); *trans* conformer δ / ppm = 170.8, 169.0, 58.7, 54.8, 52.2, 37.2, 36.2, 34.3, 22.4.
$K_{trans/cis}$ (400 MHz, Chloroform-d) = 6.5
MS (ESI): m/z (%) = 248.0 $[M+Na]^+$, 473.1 $[2M+Na]^+$ calcd. for $C_9H_{15}N_5O_2$.

2.9. H-Pro-Pro-Asp-NH_2 ·TFA (4)

H-Pro-Pro-Asp-NH_2 ·TFA (4)

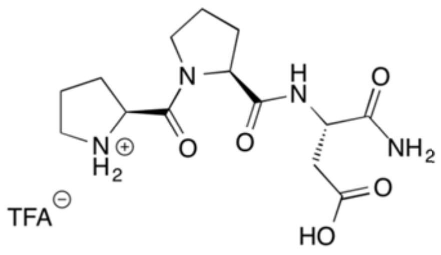

H-Pro-Pro-Asp-NH_2 ·TFA (**4**) was purchased from Bachem and used without further purification. The analytical data is in agreement with published data.[3]

^{1}H NMR (600 MHz, $CDCl_3$:CD_3OH 9:1); *trans* conformer δ/ppm = 8.00 (d, *J* = 8.3 Hz, 1H; D3-HN), 7.11 (s, 1H; NH_2), 6.45 (s, 1H; NH_2), 4.67-4.61 (m, 2H, Hα), 4.49 (dd, *J* = 8.2, 5.3 Hz, 1H; Hα), 3.71-3.67 (m, 1H; Hδ), 3.58-3.54 (m, 1H; Hδ), 3.41-3.38 (m, 2H; Hδ), 2.94 (dd, *J* = 17.0, 5.6 Hz, 1H; E3-Hβ), 2.75 (dd, *J* = 17.1, 5.6 Hz, 1H; E3-Hβ), 2.52-2.46 (m, 1H; Hβ), 2.26-2.22 (m, 1H, Hβ), 2.15-2.01 (m, 6H, Hβ/Hγ). Isolated signals of the *cis* conformer δ/ppm 8.69 (d, *J* = 8.1 Hz, 1H, D3-HN), 7.49 (s, 1H; NH_2), 6.40 (d, *J* = 0.2 Hz, 1H; NH_2), 4.80 (ddd, *J* = 9.2, 8.2, 4.7 Hz, 1H; Hα), 4.46 (dd, *J* = 8.5, 3.1 Hz, 1H; Hα), 4.24 (dd, *J* = 8.5, 6.9 Hz, 1H; Hα), 3.74-3.71 (m, 1H; Hδ), 3.54-3.52 (m, 1H; Hδ), 3.45-3.42 (m, 2H, Hδ), 2.87 (dd, *J* = 16.0, 4.7 Hz, 1H; E3-Hβ), 2.39-2.33 (m, 1H; Hβ), 1.99-1.90 (m, 3H; Hβ/Hγ).

^{13}C NMR (151 MHz, $CDCl_3$:CD_3OH 9:1); *trans* conformer δ/ppm 174.00 (CO), 173.90 (CO), 171.71 (CO), 168.50 (CO), 61.24 (Cα), 58.93 (Cα), 49.70 (Cα), 47.60 (Cδ), 46.64 (Cδ), 35.58 (E3-Cβ), 28.87 (Cβ), 28.84 (Cβ), 25.18 (Cγ), 24.54 (Cγ). *cis* conformer 173.94 (CO), 173.74 (CO), 171.93 (CO), 167.91 (CO), 60.13 (Cα), 58.89 (Cα), 50.41 (Cα), 47.90 (Cδ), 46.98 (Cδ), 36.80 (E3-Cβ), 32.11 (Cβ), 29.24 (Cβ), 24.51 (Cγ), 22.46 (Cγ).

$K_{trans/cis}$ (600 MHz, $CDCl_3$:CD_3OH 9:1) = 6.0

^{1}H NMR (600 MHz, DMSO-d_6); *trans* conformer δ/ppm = 8.11 (d, *J* = 8.0 Hz, 1H; E3-HN), 7.12 (s, 1H; NH_2), 7.10 (s, 1H; NH_2), 4.48 (t, *J* = 6.7 Hz, 1H; Hα), 4.44-4.40 (m, 1H; Hα), 4.36 (dd, *J* = 8.5, 5.1 Hz, 1H; Hα), 3.65-3.61 (m, 1H; Hδ), 3.49-3.44 (m, 1H; Hδ), 3.27-3.22 (m, 1H; Hδ), 3.19-3.15 (m, 1H; Hδ), 2.65 (dd, *J* = 16.4, 6.2 Hz, 1H; E3-Hβ), 2.53-2.50 (overlapp, 1H; E3-Hβ), 2.43-2.36 (m, 1H; Hβ), 2.15-2.09 (m, 1H; Hβ), 1.98-1.85 (m, 6H; Hβ/Hγ). Isolated signals of the *cis* conformer δ/ppm = 8.57 (d, *J* = 8.0 Hz, 1H; E3-HN), 7.40 (s, 1H; NH_2), 7.15 (s, 1H; NH_2), 4.53 (td, *J* = 8.2, 5.4 Hz, 1H; Hα), 3.94 (t, *J* = 8.2 Hz, 1H, Hα), 3.51 (td, *J* = 12.5, 6.5 Hz, 1H; Hδ), 2.73 (dd, *J* = 16.4, 5.4 Hz, 1H; E3-Hβ), 2.57 (dd, *J* = 16.4, 8.5 Hz, 1H; E3-Hβ), 2.25 (dq, *J* = 12.6, 8.5 Hz, 1H; Hβ), 2.07-2.03 (m, 1H; Hβ), 1.84-1.74 (m, 3H; Hβ/Hγ).

^{13}**C NMR** (151 MHz; DMSO-d_6): *trans* conformer δ/ppm = 172.06 (CO), 171.75 (CO), 170.54 (CO), 166.90 (CO), 59.87 (Cα), 58.20 (Cα), 49.20 (Cα), 46.72 (Cδ), 45.73 (Cδ), 35.88 (E3-Cβ), 28.78 (Cβ), 27.79 (Cβ), 24.34, 23.39. *cis* conformer δ/ppm = 171.84 (CO), 171.79 (CO), 170.49 (CO), 166.52 (CO), 58.74 (Cα), 58.35 (Cα), 49.35 (Cα), 47.28 (Cδ), 45.38 (Cδ), 36.02 (E3-Cβ), 31.48 (Cβ), 27.70 (Cβ), 23.40 (Cγ), 21.85 (Cγ).

$\mathbf{K_{trans/cis}}$ (600 MHz, DMSO-d_6) = 3.4

3. Quantum Chemical Calculations of Methylesters and Dimethylamides of Proline Derivatives

All geometries were optimized at the PBE0-D3[4,5]/def2-TZVP[6] level of theory. The thermal corrections to the Gibbs free energy at 298.15 K were calculated for all minima from unscaled vibrational frequencies obtained at the same level. The dipole moments were obtained at the same level of theory. The thermal corrections to the Gibbs free energy were combined with single point energies calculated at the RI-MP2/def2-QZVP//PBE0-D3/def2-TZVP level to yield Gibbs free energies (G_{298}) at 298.15 K. The implicit solvation model COSMO[7] was exploited for solvent effects consideration. The dipole moments of all systems were calculated as Boltzmann-averaged values over all available conformers, relying on the Gibbs free energies. All quantum mechanical calculations were performed with the Turbomole program package (versions 6.3.1 and 6.6)[8].

3.1. Gibbs free energies and dipole moments of Ac-Pro-OMe (1-OMe) and Ac-Pro-NMe$_2$ (1-NMe$_2$)

Table 1 Relative free energies ΔG, dipole moments μ [D] and Boltzmann distribution averaged dipole moments of *trans, cis, endo* and *exo* conformers of Ac-Pro-OMe (**1-OMe**) and Ac-Pro-NMe$_2$ (**1-NMe$_2$**). Calculated with $CHCl_3$ as solvent at the PBE0-D3-COSMO/def2-TZVP level of theory.

	Ac-Pro-OMe (**1-OMe**)				Ac-Pro-NMe$_2$ (**1-NMe$_2$**)			
	endo		*exo*		*endo*		*exo*	
trans								
**Ψ (N_i-C_i^{α}-C_i-OCH_3/NMe_2)**	**156°**	**-25°**	**144°**	**-36°**	**159°**	**-18°**	**133°**	**-40°**
ΔG [kJ/mol] (RI-MP2-COSMO/def2-QZVP//PBE0-D3-COSMO/def2-TZVP)	0.0	2.6	1.5	3.3	0.0	18.9	3.2	12.8
μ [D] PBE0-D3-COSMO/def2-TZVP	5.02	6.87	5.02	6.95	5.57	8.12	4.64	8.56
μ_{endo} (RI-MP2)"BOLTZMANN"	**5.50**				**5.57**			
μ_{exo} (RI-MP2)"BOLTZMANN"			5.64				4.72	
μ_{ALL} (RI-MP2) "BOLTZMANN"	5.55				5.38			
cis								
**Ψ (N_i-C_i^{α}-C_i-OCH_3/NMe_2)**	**166°**	**-16°**	**157°**	**-31°**	**167°**	**-16°**	**161°**	**-39°**
ΔG [kJ/mol] (RI-MP2-COSMO/def2-QZVP//PBE0-D3-COSMO/def2-TZVP)	0.0	1.4	3.1	4.5	0.0	13.9	3.1	8.1
μ [D] PBE0-D3-COSMO/def2-TZVP	7.01	3.45	6.96	3.83	9.44	3.10	9.45	4.21
μ_{endo} (RI-MP2)"BOLTZMANN"	**5.72**				**9.41**			
μ_{exo} (RI-MP2)"BOLTZMANN"			5.82				8.84	
μ_{ALL} RI-MP2)"BOLTZMANN"	5.74				9.27			
**Δ $\mu_{cis\text{-}trans}$ [D]**								
endo (RI-MP2)"BOLTZMANN"	**0.22**				**3.84**			
exo (RI-MP2)"BOLTZMANN"			0.18				4.12	
ALL (RI-MP2) "BOLTZMANN"	0.19				3.89			

3.2. Indicators for the n → π* Interaction in Ac-Pro-OMe (1-OMe) and Ac-Pro-OMe (1-NMe$_2$)

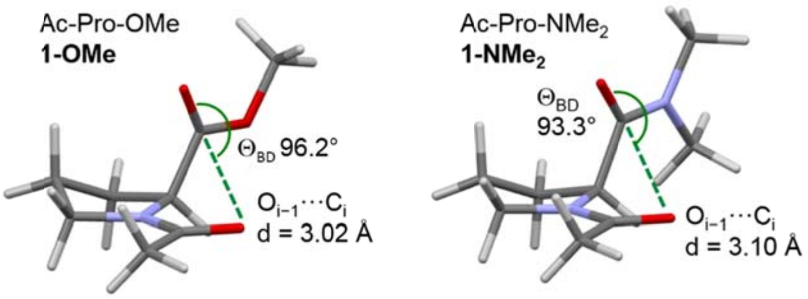

Figure 1 Distances and angles within Ac-Pro-OMe (**1-OMe**) and Ac-Pro-NMe$_2$ (**1-NMe$_2$**). Pyramidalization at **C**O$_2$Me = 0.020 Å, at **C**ONMe$_2$ = 0.017 Å.

3.3. Gibbs free energies and dipole moments of Ac-(4*R*)Flp-OMe (2R-OMe) and Ac-(4*R*)Flp-NMe_2 (2R-NMe_2)

Table 2 Relative free energies ΔG, dipole moments μ [D] and Boltzmann distribution averaged dipole moments of *trans, cis, endo* and *exo* conformers of Ac-(4*R*)Flp-OMe (**2R-OMe**) and Ac-(4*R*)Flp-NMe_2 (**2R-NMe_2**). Calculated with $CHCl_3$ as solvent at the PBE0-D3-COSMO/def2-TZVP level of theory.

	Ac-(4*R*)Flp-OMe (**2R-OMe**)				Ac-(4*R*)Flp -NMe_2 (**2R-NMe_2**)			
	exo		*endo*		*exo*		*endo*	
trans								
**Ψ (N_i-C_i^{α}-C_i-OCH_3/NMe_2)**	**143°**	**-36°**	**153°**	**-29°**	**131°**	**-40°**	**131°**	**-20°**
ΔG [kJ/mol] (RI-MP2-COSMO/def2-QZVP//PBE0 -D3-COSMO/def2-TZVP)	0.0	2.5	8.0	10.4	0.0	12.2	8.0	27.8
μ [D] PBE0-D3-COSMO/def2-TZVP	4.26	7.30	2.42	5.04	2.83	9.22	2.42	6.84
μ_{exo} (RI-MP2)"BOLTZMANN"	**5.07**				**2.88**			
μ_{ALL} (RI-MP2) "BOLTZMANN"		5.00				2.86		
cis								
**Ψ (N_i-C_i^{α}-C_i-OCH_3/NMe_2)**	**156°**	**-32°**	**165°**	**-22°**	**160°**	**-39°**	**165°**	**-17°**
ΔG [kJ/mol] (RI-MP2-COSMO/def2-QZVP//PBE0-D3-COSMO/def2-TZVP)	0.0	1.5	7.2	8.2	0.0	8.1	7.4	24.1
μ [D] PBE0-D3-COSMO/def2-TZVP	6.67	4.75	7.13	3.47	8.96	5.67	9.57	3.35
μ_{exo} (RI-MP2)"BOLTZMANN"	**6.00**				**8.84**			
μ_{ALL} RI-MP2)"BOLTZMANN"		5.98				8.88		
**Δ $\mu_{cis\text{-}trans}$ [D]**								
exo (RI-MP2)"BOLTZMANN"	**0.93**				**5.96**			
ALL (RI-MP2) "BOLTZMANN"		**0.98**				**6.01**		

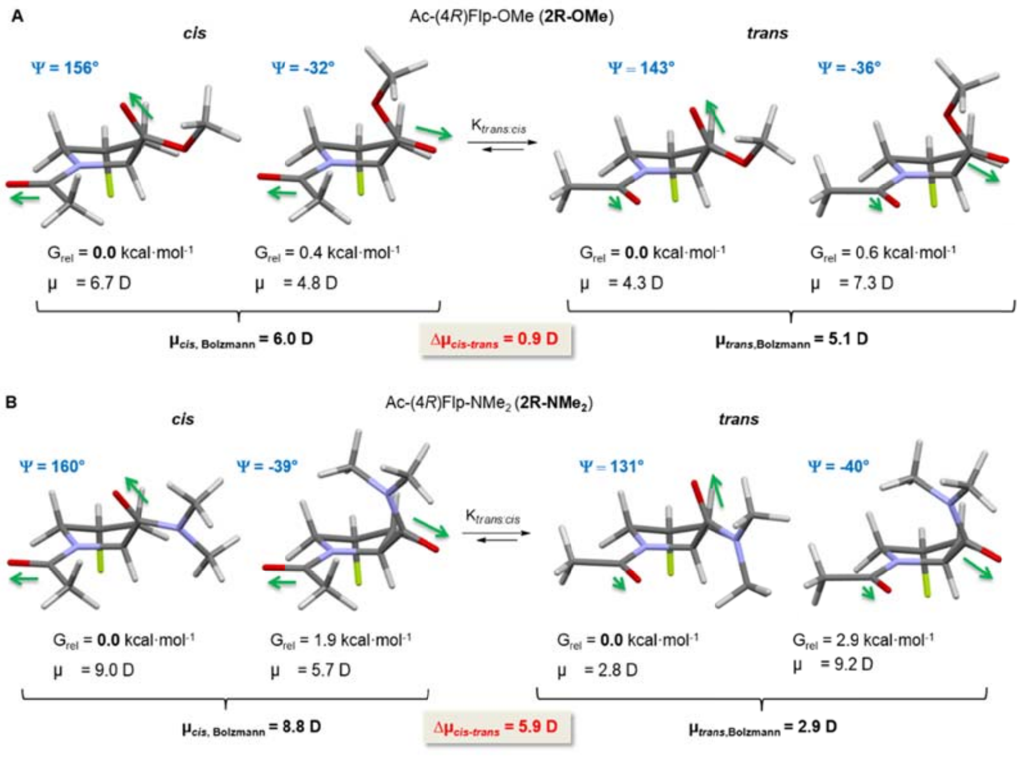

Figure 2 Lowest energy structures and Boltzmann distribution averaged dipole moments μ [D] of *trans* and *cis* conformers of Ac-(4*R*)Flp-OMe (**2R-OMe**) and Ac-(4*R*)Flp-NMe_2 (**2R-NMe_2**) calculated with $CHCl_3$ as solvent at the PBE0-D3-COSMO/def2-TZVP level of theory.

3.4. Gibbs free energies and dipole moments of Ac-(4*R*)Azp-OMe (3R-OMe) and Ac-(4*R*)Azp-NMe$_2$ (3R-NMe$_2$)

Table 3 Relative free energies ΔG, dipole moments μ [D] and Boltzmann distribution averaged dipole moments of *exo* puckered *trans and cis* conformers of Ac-(4*R*)Azp-OMe (**3R-OMe**) considering the C^{δ}-C^{γ}-N-N tosion. Calculated with $CHCl_3$ as solvent at the PBE0-D3-COSMO/def2-TZVP level of theory.

	Ac-(4*R*)Azp-OMe (**3R-OMe**) *exo*					
trans						
**Ψ (N_i-C_i^{α}-C_i-OCH_3)**	**143.8°**	**143.5°**	**145.1°**	**-34.9°**	**-35.2°**	**-34.3°**
ΔG [kJ/mol] (RI-MP2-COSMO/def2-QZVP//PBE0 -D3-COSMO/def2-TZVP)	0. 0	0.8	2.1	2.7	3.1	5.2
μ [D] PBE0-D3-COSMO/def2-TZVP	4.11	4.17	4.63	7.46	7.17	7.67
C^{δ}-C^{γ}-N-N tosion	-178.0°	65.9°	-31.6°	-178.3°	65.8°	-26.3°
μ_{ALL} (RI-MP2) "BOLTZMANN"				**5.05**		
cis						
**Ψ (N_i-C_i^{α}-C_i-OCH_3)**	**156.9°**	**-31.3°**	**156.7°**	**-31.1°**	**166.0°**	**-30.5°**
ΔG [kJ/mol] (RI-MP2-COSMO/def2-QZVP//PBE0 -D3-COSMO/def2-TZVP)	0.0	1.1	0.4	1.6	1.9	2.2
μ [D] PBE0-D3-COSMO/def2-TZVP	7.06	5.17	6.34	4.73	7.14	5.35
C^{δ}-C^{γ}-N-N torsion	66.1°	66.1°	-179.9°	-179.8°	-68.2°	-36.5°
μ_{ALL} (RI-MP2) "BOLTZMANN"				**6.11**		
Δ $\mu_{cis\text{-}trans}$ [D]				**1.06**		

Table 4 Relative free energies, dipole moments μ [D] and Boltzmann distribution averaged dipole moments of *exo* puckered *trans and cis* conformers of Ac-(4*R*)Azp-NMe$_2$ (**3R-NMe$_2$**) considering the C$^{\delta}$-C$^{\gamma}$-N-N tosion. Calculated with $CHCl_3$ as solvent at the PBE0-D3-COSMO/def2-TZVP level of theory.

			Ac-(4*R*)Azp-NMe$_2$ (**3R-NMe$_2$**) *exo*			
trans						
**Ψ (N$_i$-C$_i^{\alpha}$-C$_i$-NMe$_2$)**	**131.2°**	**131.3°**	**131.4°**	**-39.0°**	**-39.3°**	**-39.0°**
ΔG [kJ/mol] (RI-MP2-COSMO/def2-QZVP//PBE0 -D3-COSMO/def2-TZVP)	0.0	0.3	1.0	13.4	14.1	16.2
μ [D] PBE0-D3-COSMO/def2-TZVP	2.53	2.23	2.71	9.44	9.11	9.64
C$^{\delta}$-C$^{\gamma}$-N-N torsion	-73.5°	-176.4°	65.2°	-178.1°	66.0°	-26.1°
μ$_{ALL}$ (RI-MP2) "BOLTZMANN"				2.50		
cis						
**Ψ (N$_i$-C$_i^{\alpha}$-C$_i$-NMe$_2$)**	**159.8°**	**160.4°**	**165.6°**	**-38.2°**	**-37.9°**	**-37.9°**
ΔG [kJ/mol] (RI-MP2-COSMO/def2-QZVP//PBE0 -D3-COSMO/def2-TZVP)	0.0	1.0	2.7	7.6	8.0	10.5
μ [D] PBE0-D3-COSMO/def2-TZVP	9.23	8.45	9.16	6.04	5.84	6.39
C$^{\delta}$-C$^{\gamma}$-N-N tosion	65.6°	-178.2°	-74.2°	66.4°	-179.7°	-29.4°
μ$_{ALL}$ (RI-MP2) "BOLTZMANN"				8.82		
Δ μ$_{cis\text{-}trans}$ [D]				**6.32**		

Table 5 Relative free energies ΔG and dipole moments μ [D] averaged dipole moments of *endo* puckered *trans and cis* conformers of Ac-(4*R*)Azp-OMe (**3R-OMe**) considering the C$^{\delta}$-C$^{\gamma}$-N-N tosion. Calculated with $CHCl_3$ as solvent at the PBE0-D3-COSMO/def2-TZVP level of theory.

			Ac-(4*R*)Azp-OMe (**3R-OMe**) *endo*			
trans						
**Ψ (N$_i$-C$_i^{\alpha}$-C$_i$-OCH$_3$)**	**152.4°**	**152.0°**	**152.5°**	**-27.9°**	**-28.3°**	**-28°**
ΔG [kJ/mol] (RI-MP2-COSMO/def2-QZVP//PBE0 -D3-COSMO/def2-TZVP)	4.7	5.0	6.8	6.8	7.6	9.1
μ [D] PBE0-D3-COSMO/def2-TZVP	1.99	2.14	1.93	4.75	4.47	4.84
C$^{\delta}$-C$^{\gamma}$-N-N tosion	159.7	93.3	-62.4	160.6	92.6	-62.6
cis						
**Ψ (N$_i$-C$_i^{\alpha}$-C$_i$-OCH$_3$)**	**163.8°**	**164.3°**	**-20.7°**	**-19.8°**	**164.5°**	**-20.0°**
ΔG [kJ/mol] (RI-MP2-COSMO/def2-QZVP//PBE0 -D3-COSMO/def2-TZVP)	3.5	3.7	5.0	5.0	5.7	6.9
μ [D] PBE0-D3-COSMO/def2-TZVP	7.39	6.99	3.59	3.27	7.27	3.85
C$^{\delta}$-C$^{\gamma}$-N-N torsion	88.9	159.9	89.1	160.1	-60.5	-60.7

Table 6 Relative free energies ΔG and dipole moments μ [D] of *endo* puckered *trans and cis* conformers of Ac-(4*R*)Azp-NMe_2 (**3R-NMe_2**) considering the C^{δ}-C^{γ}-N-N tosion. Calculated with $CHCl_3$ as solvent at the PBE0-D3-COSMO/def2-TZVP level of theory.

	Ac-(4*R*)Azp-NMe_2 (**3R-NMe_2**) *endo*					
trans						
**Ψ (N_i-C_i^{α}-C_i-NMe_2)**	**131.5°**	**132.6°**	**-17.1°**	**-16.8°**		
ΔG [kJ/mol] (RI-MP2-COSMO/def2-QZVP//PBE0 -D3-COSMO/def2-TZVP)	5.5	7.0	25.5	26.9		
μ [D] PBE0-D3-COSMO/def2-TZVP	2.78	2.36	6.26	6.73		
C^{δ}-C^{γ}-N-N torsion	157.8	-62.3	92.8	-63.3		
cis						
**Ψ (N_i-C_i^{α}-C_i-NMe_2)**	**165.5°**	**165.7°**	**165.7°**	**-14.1°**	**-14.5°**	**-14.2°**
ΔG [kJ/mol] (RI-MP2-COSMO/def2-QZVP//PBE0 -D3-COSMO/def2-TZVP)	4.3	4.4	6.4	20.0	20.4	22.8
μ [D] PBE0-D3-COSMO/def2-TZVP	9.86	9.43	9.63	3.23	3.26	3.88
C^{δ}-C^{γ}-N-N tosion	88.9	159.8	-60.5	162.9	89.2	-61.6

Table 7 Bolzmann averaged differences in dipole moments Δ $\mu_{cis\text{-}trans}$ [D] for the *exo* puckered conformations as well as over all *exo* and *endo* conformations.

**Δ $\mu_{cis\text{-}trans}$ [D]**		
	Ac-(4*R*)Azp-OMe (**3R-OMe**)	Ac-(4*R*)Azp-NMe_2 (**3R-NMe_2**)
exo (RI-MP2)"BOLTZMANN"	**1.06**	**6.32**
ALL (RI-MP2) "BOLTZMANN"	**1.34**	**6.44**

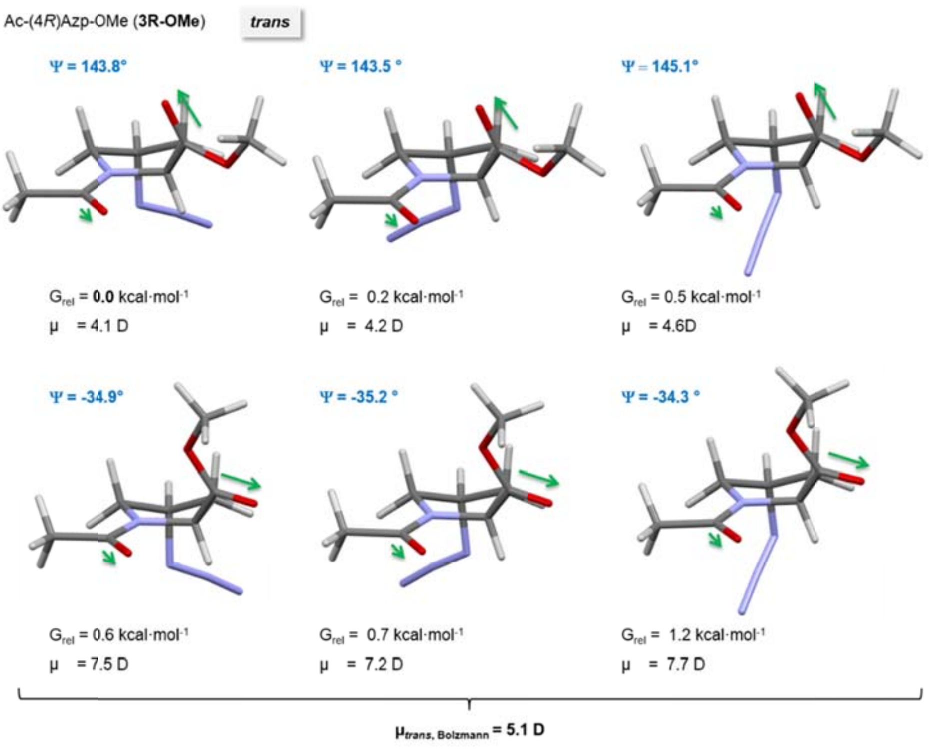

Figure 3 Lowest energy structures and Boltzmann distribution averaged dipole moments μ [D] of *trans* conformers of Ac-(4*R*)Azp-OMe (**3R-OMe**) calculated with $CHCl_3$ as solvent at the PBE0-D3-COSMO/def2-TZVP level of theory.

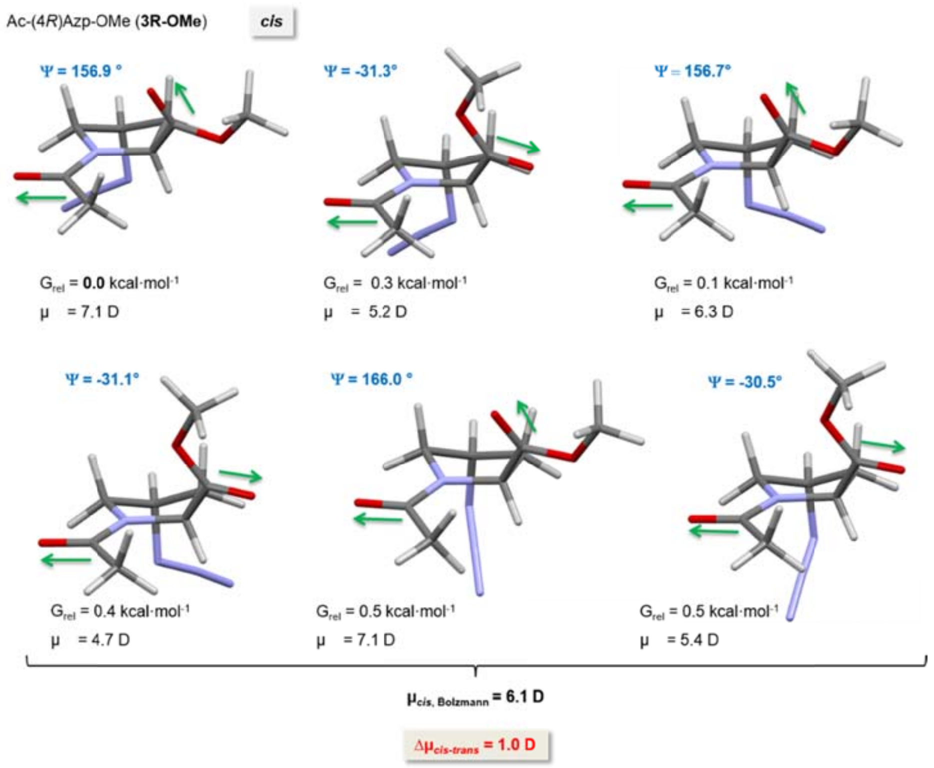

Figure 4 Lowest energy structures and Boltzmann distribution averaged dipole moments μ [D] of *cis* conformers of Ac-(4*R*)Azp-OMe (**3R-OMe**) calculated with $CHCl_3$ as solvent at the PBE0-D3-COSMO/def2-TZVP level of theory.

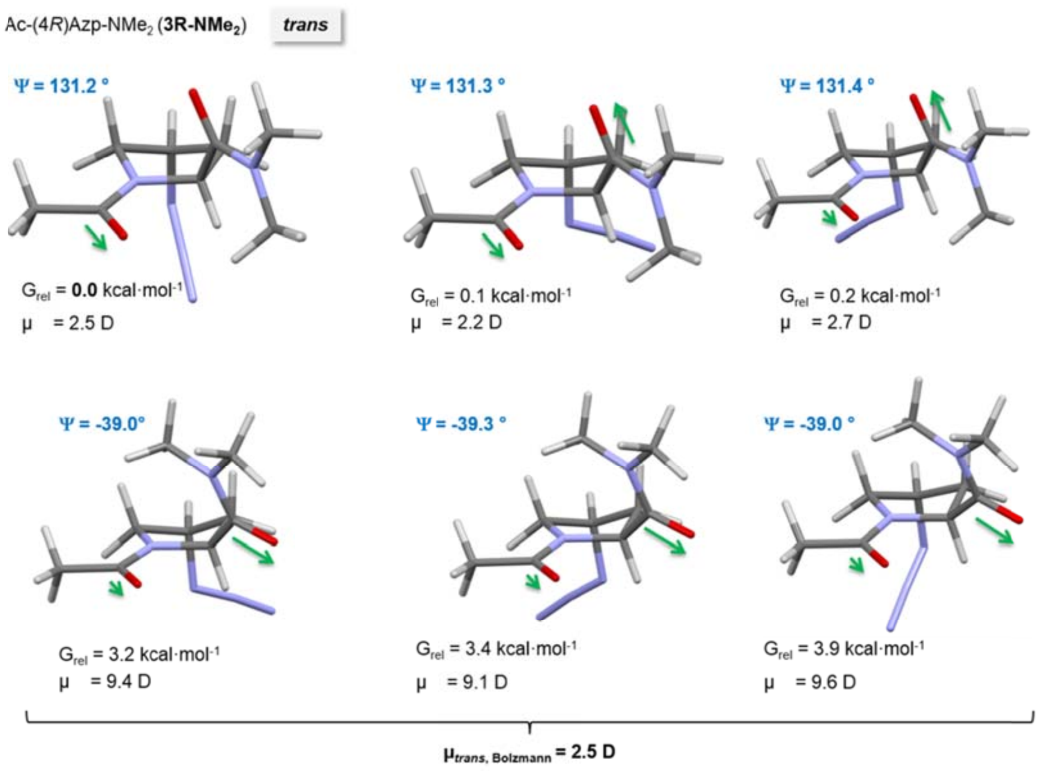

Figure 5 Lowest energy structures and Boltzmann distribution averaged dipole moments μ [D] of *trans* conformers of Ac-(4*R*)Azp-NMe$_2$ (**3R-NMe$_2$**) calculated with $CHCl_3$ as solvent at the PBE0-D3-COSMO/def2-TZVP level of theory.

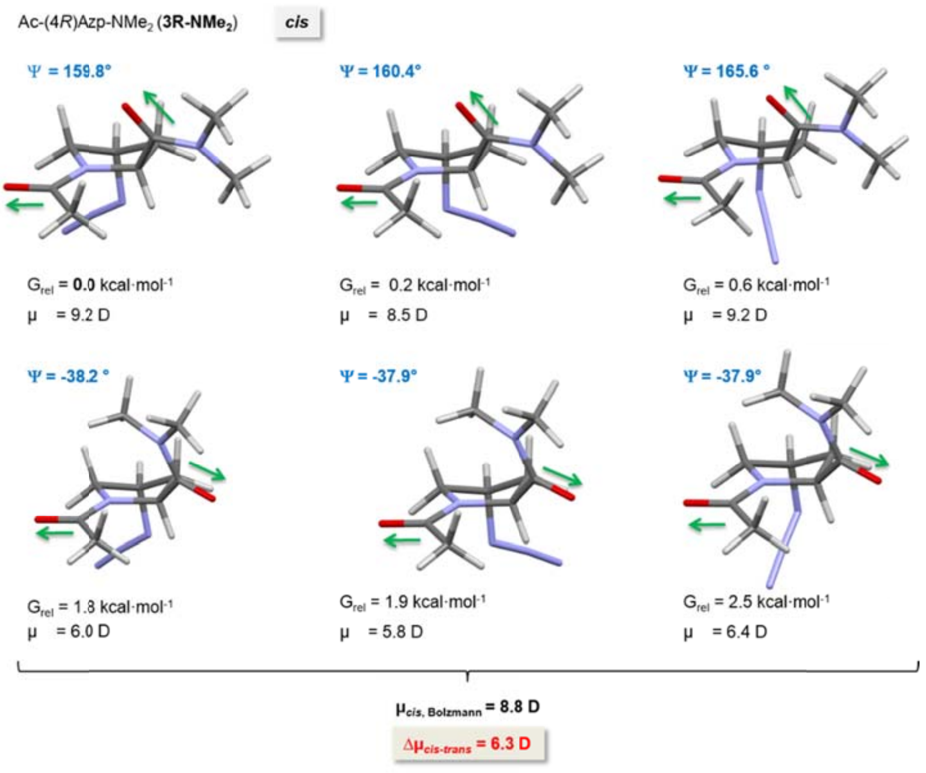

Figure 6 Lowest energy structures and Boltzmann distribution averaged dipole moments μ [D] of *cis* conformers of Ac-(4*R*)Azp-NMe$_2$ (**3R-NMe$_2$**) calculated with $CHCl_3$ as solvent at the PBE0-D3-COSMO/def2-TZVP level of theory.

3.5. Gibbs free energies and dipole moments of Ac-(4*S*)Flp-OMe (2S-OMe) and Ac-(4*S*)Flp-NMe_2 (2S-NMe_2)

Table 8 Relative free energies ΔG, dipole moments μ [D] and Boltzmann distribution averaged dipole moments of *trans, cis, endo* and *exo* conformers of Ac-(4*S*)Flp-OMe (**2S-OMe**) and Ac-(4*S*)Flp-NMe_2 (**2S-NMe_2**). Calculated with $CHCl_3$ as solvent at the PBE0-D3-COSMO/def2-TZVP level of theory.

	Ac-(4*S*)Flp-OMe (**2S-OMe**)				Ac-(4*S*)Flp -NMe_2 (**2S-NMe_2**)			
	endo		*exo*		*endo*		*exo*	
trans								
**Ψ (N_i-C_i^{α}-C_i-OCH_3/NMe_2)**	**173°**	**-3°**	**146°**	**-35°**	**171°**	**-10°**	**133°**	**-40°**
ΔG [kJ/mol] (RI-MP2-COSMO/def2-QZVP//PBE0 -D3-COSMO/def2-TZVP)	0.0	1.2	6.6	8.2	0.0	13.5	4.1	14.8
μ [D] PBE0-D3-COSMO/def2-TZVP	5.59	5.99	3.03	4.82	6.50	7.31	4.03	6.60
μ_{endo} (RI-MP2)"BOLTZMANN"	5.74				6.50			
μ_{ALL} (RI-MP2) "BOLTZMANN"		**5.61**				**6.11**		
cis								
**Ψ (N_i-C_i^{α}-C_i-OCH_3/NMe_2)**	**-176°**	**4°**	**160°**	**-31°**	**174°**	**-8°**	**163°**	**-39°**
ΔG [kJ/mol] (RI-MP2-COSMO/def2-QZVP//PBE0-D3-COSMO/def2-TZVP)	0.0	0.4	9.7	11.5	0.0	9.42	6.17	11.8
μ [D] PBE0-D3-COSMO/def2-TZVP	7.18	2.56	7.11	3.08	9.74	0.88	9.66	2.77
μ_{endo} (RI-MP2)"BOLTZMANN"	5.06				9.54			
μ_{ALL} (RI-MP2)"BOLTZMANN"		**5.07**				**9.50**		
**Δ $\mu_{cis\text{-}trans}$ [D]**								
endo (RI-MP2)"BOLTZMANN"	**-0.68**				**3.04**			
ALL (RI-MP2) "BOLTZMANN"		**-0.54**				**3.39**		

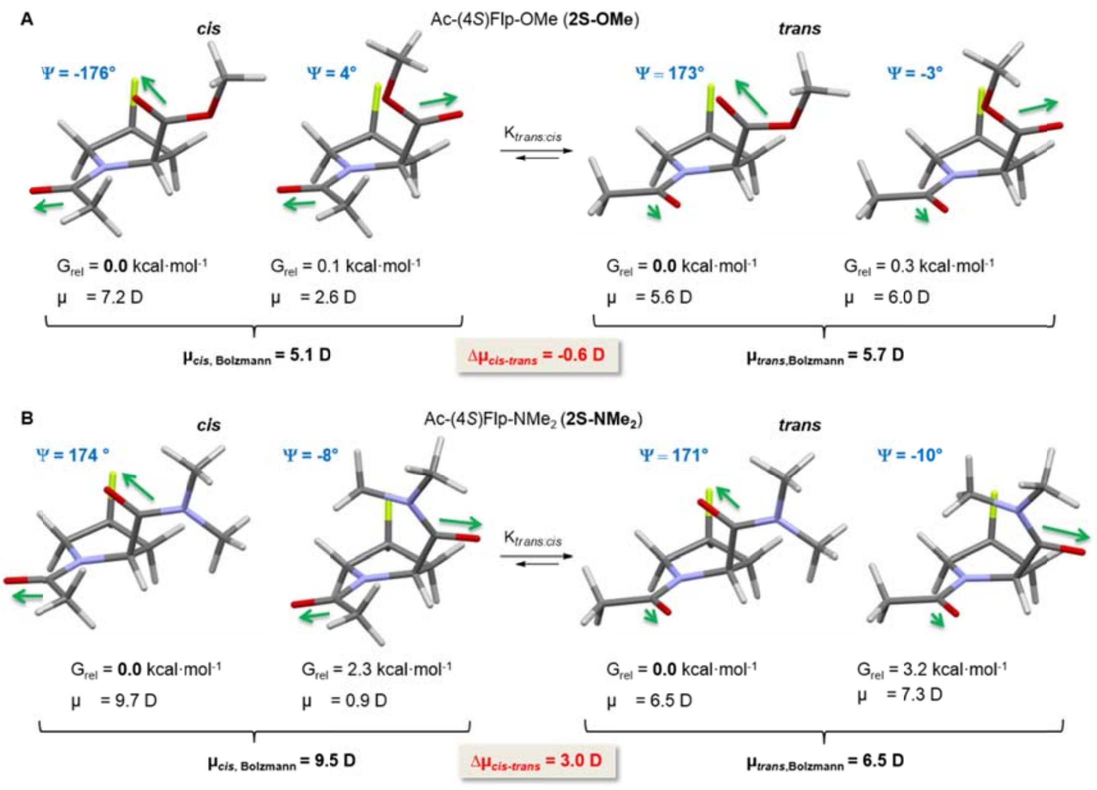

Figure 7 Lowest energy structures and Boltzmann distribution averaged dipole moments μ [D] of *trans* and *cis* conformers of Ac-(4*S*)Flp-OMe (**2S-OMe**) and Ac-(4*S*)Flp-NMe_2 (**2S-NMe_2**) calculated with $CHCl_3$ as solvent at the PBE0-D3-COSMO/def2-TZVP level of theory.

3.6. Gibbs free energies and dipole moments of Ac-(4*S*)Azp-OMe (3S-OMe) and Ac-(4*S*)Azp-NMe$_2$ (3S-NMe$_2$)

Table 9 Relative free energies ΔG, dipole moments μ [D] and Boltzmann distribution averaged dipole moments of *endo* puckered *trans and cis* conformers of Ac-(4*S*)Azp-OMe (**3S-OMe**) considering the C^{δ}-C^{γ}-N-N tosion. Calculated with $CHCl_3$ as solvent at the PBE0-D3-COSMO/def2-TZVP level of theory.

	Ac-(4*S*)Azp-OMe (**3S-OMe**) *endo*			
trans				
**Ψ (N_i-C_i^{α}-C_i-OCH_3)**	**-177.4°**	**11.0°**	**178.0°**	**-0.1°**
ΔG [kJ/mol] (RI-MP2-COSMO/def2-QZVP//PBE0 -D3-COSMO/def2-TZVP)	0.0	0.7	0.9	1.3
μ [D] PBE0-D3-COSMO/def2-TZVP	5.64	5.70	5.80	5.68
C^{δ}-C^{γ}-N-N tosion	-172.6	176.5	-51.9	-52.3
μ_{ALL} (RI-MP2) "BOLTZMANN"		**5.70**		
cis				
**Ψ (N_i-C_i^{α}-C_i-OCH_3)**	**15.1°**	**5.3°**	**-164.8°**	**-173.1°**
ΔG [kJ/mol] (RI-MP2-COSMO/def2-QZVP//PBE0 -D3-COSMO/def2-TZVP)	0.0	0.2	0.2	0.9
μ [D] PBE0-D3-COSMO/def2-TZVP	2.47	3.06	6.74	7.56
C^{δ}-C^{γ}-N-N torsion	176.7	-56.4	174.7	-56.0
μ_{ALL} (RI-MP2) "BOLTZMANN"		**4.73**		
Δ $\mu_{cis\text{-}trans}$ [D]		**-0.97**		

Table 10 Relative free energies ΔG, dipole moments μ [D] and Boltzmann distribution averaged dipole moments of *endo* puckered *trans and cis* conformers of Ac-(4*S*)Azp-NMe$_2$ (**3S-NMe$_2$**) considering the C^{δ}-C^{γ}-N-N tosion. Calculated with $CHCl_3$ as solvent at the PBE0-D3-COSMO/def2-TZVP level of theory.

	Ac-(4*S*)Azp-NMe$_2$ (**3S-NMe$_2$**) *endo*			
trans				
**Ψ (N_i-C_i^{α}-C_i-NMe_2)**	**171.3°**	**170.6°**	**-3.1°**	**-4.7°**
ΔG [kJ/mol] (RI-MP2-COSMO/def2-QZVP//PBE0 -D3-COSMO/def2-TZVP)	0.0	2.1	10.6	14.0
μ [D] PBE0-D3-COSMO/def2-TZVP	6.80	7.09	7.25	6.60
C^{δ}-C^{γ}-N-N torsion	149.1	-20.2	170.7	-67.6
μ_{ALL} (RI-MP2) "BOLTZMANN"		**6.89**		
cis				
**Ψ (N_i-C_i^{α}-C_i-NMe_2)**	**178.5°**	**174.3°**	**-1.9°**	**-3.5°**
ΔG [kJ/mol] (RI-MP2-COSMO/def2-QZVP//PBE0 -D3-COSMO/def2-TZVP)	0.0	1.4	8.6	8.8
μ [D] PBE0-D3-COSMO/def2-TZVP	9.36	10.22	0.28	0.69
C^{δ}-C^{γ}-N-N tosion	161.4	-25.1	174.5	-66.5
μ_{ALL} (RI-MP2) "BOLTZMANN"		**9.34**		
Δ $\mu_{cis\text{-}trans}$ [D]		**2.45**		

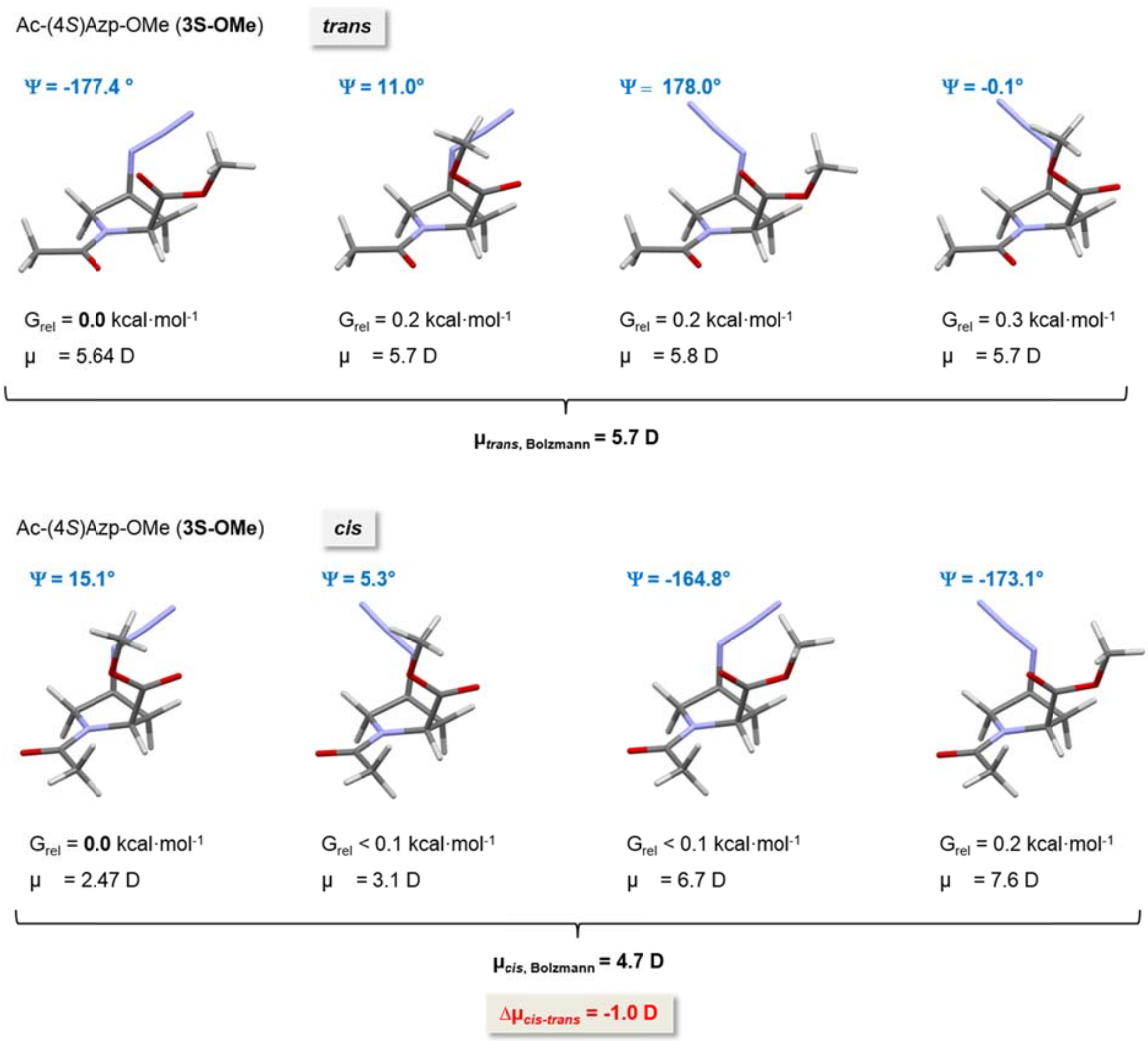

Figure 8 Lowest energy structures and Boltzmann distribution averaged dipole moments μ [D] of *trans* and *cis* conformers of Ac-(4*S*)Azp-OMe (**3S-OMe**) calculated with $CHCl_3$ as solvent at the PBE0-D3-COSMO/def2-TZVP level of theory.

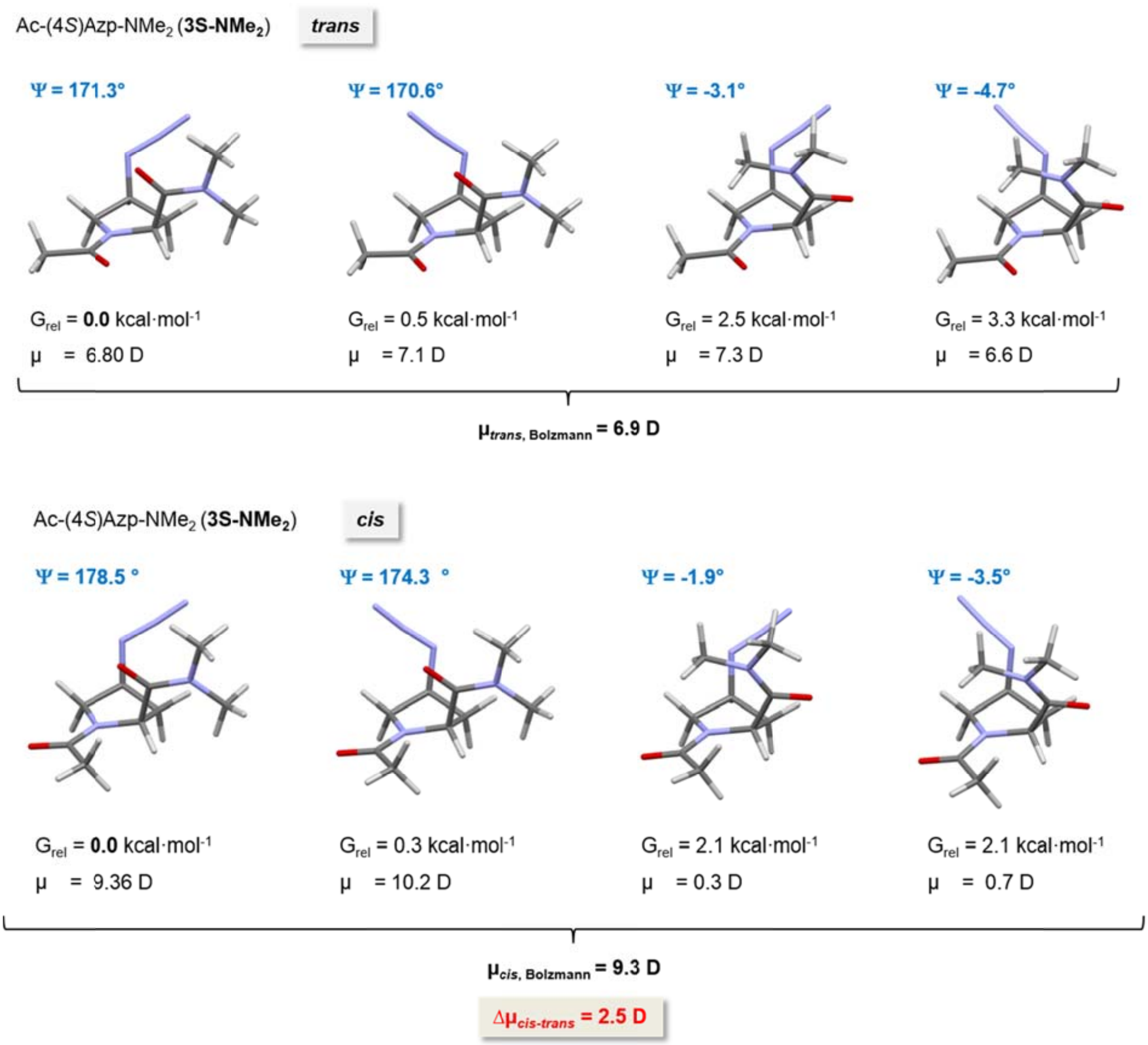

Figure 9 Lowest energy structures and Boltzmann distribution averaged dipole moments μ [D] of *trans* and *cis* conformers of Ac-(4*S*)Azp-NMe_2 (**3S-NMe_2**) calculated with $CHCl_3$ as solvent at the PBE0-D3-COSMO/def2-TZVP level of theory.

Table 11 Relative free energies ΔG and dipole moments μ [D] of *exo* puckered *trans and cis* conformers of Ac-(4*S*)Azp-OMe (**3S-OMe**) considering the C^{δ}-C^{γ}-N-N tosion. Calculated with $CHCl_3$ as solvent at the PBE0-D3-COSMO/def2-TZVP level of theory.

	Ac-(4*S*)Azp-OMe (**3S-OMe**) *exo*					
trans						
**Ψ (N_i-C_i^{α}-C_i-OCH_3)**	**145.2°**	**145.7°**	**-34.9°**	**-34.6°**	**144.8°**	**-35.4°**
ΔG [kJ/mol] (RI-MP2-COSMO/def2-QZVP//PBE0 -D3-COSMO/def2-TZVP)	4.2	5.0	5.9	5.9	6.0	7.8
μ [D] PBE0-D3-COSMO/def2-TZVP	2.53	2.55	4.65	4.40	3.04	4.43
C^{δ}-C^{γ}-N-N tosion	-162.9	-90.9	-162.6	-92.7	62.7	62.8
cis						
**Ψ (N_i-C_i^{α}-C_i-OCH_3)**	160.4	159.1	-30.7	-30.4	158.2	-31.7
ΔG [kJ/mol] (RI-MP2-COSMO/def2-QZVP//PBE0 -D3-COSMO/def2-TZVP)	7.5	7.5	8.7	9.3	10.2	11.2
μ [D] PBE0-D3-COSMO/def2-TZVP	7.35	6.94	3.03	3.41	7.33	3.10
C^{δ}-C^{γ}-N-N torsion	-87.6	-161.5	-161.4	-89.3	60.8	61.0

Table 12 Relative free energies ΔG and dipole moments μ [D] of *exo* puckered *trans and cis* conformers of Ac-(4*S*)Azp-NMe_2 (**3S-NMe$_2$**) considering the C^{δ}-C^{γ}-N-N tosion. Calculated with $CHCl_3$ as solvent at the PBE0-D3-COSMO/def2-TZVP level of theory.

	Ac-(4*S*)Azp-NMe_2 (**3S-NMe$_2$**) *exo*					
trans						
**Ψ (N_i-C_i^{α}-C_i-OCH_3)**	133.1	133.2	132.8	-39.6	-39.6	-40.0
ΔG [kJ/mol] (RI-MP2-COSMO/def2-QZVP//PBE0 -D3-COSMO/def2-TZVP)	1.3	2.5	4.3	12.2	12.8	14.9
μ [D] PBE0-D3-COSMO/def2-TZVP	3.72	3.83	4.41	6.54	6.29	6.11
C^{δ}-C^{γ}-N-N tosion	164.5	-89.9	63.5	-163.0	-92.7	63.4
cis						
**Ψ (N_i-C_i^{α}-C_i-OCH_3)**	162.5	163.2	162.3	-38.4	-38.4	-38.8
ΔG [kJ/mol] (RI-MP2-COSMO/def2-QZVP//PBE0 -D3-COSMO/def2-TZVP)	3.6	5.0	7.3	9.5	9.8	12.1
μ [D] PBE0-D3-COSMO/def2-TZVP	9.42	9.87	9.88	2.81	3.05	2.33
C^{δ}-C^{γ}-N-N torsion	-162.3	-86.5	61.2	-162.0	-90.6	61.9

Table 13 Bolzmann averaged differences in dipole moments Δ µcis-trans [D] for the *endo* puckered conformations as well as over all *exo* and *endo* conformations.

**Δ $\mu_{cis\text{-}trans}$ [D]**	Ac-(4*S*)Azp-OMe (**3S-OMe**)	Ac-(4*S*)Azp-NMe_2 (**3S-NMe_2**)
endo (RI-MP2)"BOLTZMANN"	**-0.97**	**2.45**
ALL (RI-MP2) "BOLTZMANN"	**-0.51**	**3.69**

4. Cartesian Coordinates

Ac-Pro-OMe (1-OMe) Ψ = 155.6°_endo trans

H	2.0607302	1.3300942	0.3688320
C	1.7805362	0.6089001	-0.3993135
C	1.4347298	-0.7668979	0.1845492
N	0.5196121	-1.3180008	-0.7882036
C	0.0606067	-0.3353729	-1.7631849
C	0.5168574	0.9881774	-1.1636983
H	2.6258654	0.4970199	-1.0821993
C	0.7723213	-0.6251433	1.5430980
H	2.3128860	-1.4028572	0.3019350
H	0.6804978	0.5262145	3.9185088
H	-1.0217199	-0.3829630	-1.8908948
H	0.5291364	-0.5143027	-2.7376822
H	0.6909116	1.7499936	-1.9237496
C	0.1287721	-2.6043333	-0.6923351
O	0.5455790	-3.3244465	0.2096341
C	-0.8361557	-3.0943278	-1.7350665
H	-0.9400579	-4.1724096	-1.6364962
H	-0.4993386	-2.8453613	-2.7435506
H	-1.8136189	-2.6270664	-1.5879777
O	-0.4076410	-0.4708690	1.7251300
O	1.6822518	-0.6329737	2.5131174
C	1.1813732	-0.4393644	3.8383103
H	2.0499796	-0.4733582	4.4911755
H	0.4790474	-1.2326742	4.0961176
H	-0.2418158	1.3598143	-0.4714685

Ac-Pro-OMe (1-OMe) Ψ = -25.0°_endo trans

H	2.0004508	1.3244397	0.3897856
C	1.7519859	0.6000671	-0.3858419
C	1.3711563	-0.7684567	0.1933536
N	0.4927761	-1.3203629	-0.8148996
C	0.0966040	-0.3455997	-1.8249055
C	0.5322745	0.9811654	-1.2174546
H	2.6286063	0.4764620	-1.0258362
C	0.7292469	-0.6257383	1.5615391
H	2.2418008	-1.4077254	0.3456607
H	-1.0510599	-1.2188249	3.4097600
H	-0.9775007	-0.3860367	-2.0117792
H	0.6158456	-0.5398422	-2.7702355
H	0.7516452	1.7322130	-1.9764338
C	0.0820898	-2.6008475	-0.7260478
O	0.4374900	-3.3135065	0.2074763
C	-0.8306248	-3.0929937	-1.8139729
H	-0.9511565	-4.1687598	-1.7091384
H	-0.4376097	-2.8593794	-2.8056292
H	-1.8099045	-2.6146445	-1.7255330
O	1.3726403	-0.5628626	2.5790769
O	-0.5928405	-0.5173113	1.5105207
C	-1.2515998	-0.3595027	2.7691018
H	-2.3126736	-0.2944145	2.5414287
H	-0.9103109	0.5490581	3.2666769
H	-0.2580931	1.3691193	-0.5707667

Ac-Pro-OMe (1-OMe) Ψ = 166.4°_endo cis

H	2.1343019	1.2820405	0.4162138
C	1.8159094	0.5724115	-0.3471414
C	1.4537804	-0.7986613	0.2421577
N	0.5523783	-1.3458240	-0.7435838
C	0.0453198	-0.3383471	-1.6701023
C	0.5388406	0.9735773	-1.0761841
H	2.6381024	0.4394472	-1.0539472
C	0.7938016	-0.6319717	1.6021891
H	2.3429501	-1.4178059	0.3746952
H	0.5734592	0.7028442	3.8736284
H	-1.0412556	-0.4016072	-1.7393563
H	0.4535812	-0.5164776	-2.6701496
H	0.7082289	1.7357939	-1.8365963
C	0.1753314	-2.6371827	-0.8462589
O	-0.5856294	-3.0026775	-1.7350937
C	0.7357002	-3.5956700	0.1700858
H	0.3405686	-4.5873865	-0.0367488
H	0.4522931	-3.3018594	1.1834254
H	1.8269515	-3.6268331	0.1220545
O	-0.3903227	-0.6690382	1.8119531
O	1.7125748	-0.4107771	2.5383119
C	1.2153804	-0.1785956	3.8599075
H	2.0924588	-0.0212310	4.4824063
H	0.6492316	-1.0428591	4.2086398
H	-0.1939617	1.3614068	-0.3644024

Ac-Pro-OMe (1-OMe) Ψ = -16.4°_endo cis

H	2.0453000	1.2685198	0.4764815
C	1.7697100	0.5645370	-0.3083780
C	1.4041684	-0.8174041	0.2510209
N	0.5593411	-1.3684481	-0.7840576
C	0.0699067	-0.3570827	-1.7160293
C	0.5208555	0.9558559	-1.0899431
H	2.6233238	0.4497542	-0.9802217
C	0.7442003	-0.6702785	1.6134700
H	2.2971179	-1.4226470	0.4157796
H	-0.9475691	-1.3497037	3.5208107
H	-1.0125167	-0.4359734	-1.8261619
H	0.5163851	-0.5173062	-2.7024291
H	0.7140161	1.7260045	-1.8365405
C	0.2147439	-2.6652462	-0.9205229
O	-0.5054399	-3.0332745	-1.8419565
C	0.7588781	-3.6268272	0.1017853
H	0.3742969	-4.6196798	-0.1189769
H	0.4583492	-3.3436499	1.1133444
H	1.8510410	-3.6505918	0.0743593
O	1.3785725	-0.4783352	2.6207244
O	-0.5789956	-0.7424605	1.5679669
C	-1.2569583	-0.5761626	2.8173303
H	-2.3161782	-0.6671847	2.5915070
H	-1.0368573	0.4047270	3.2395904
H	-0.2473249	1.3305524	-0.4090694

Ac-Pro-OMe (1-OMe) Ψ = 144.4°_exo trans

H	-0.2883141	2.5621203	0.2662366
C	-0.2835634	1.6024847	0.7815781
C	-1.6748619	1.0629580	1.0930392
C	-1.4229269	-0.4227291	1.2988949
N	-0.3619104	-0.7000423	0.3417434
C	0.3422001	0.4953347	-0.0777038
H	0.2882673	1.7100472	1.7071995
H	-2.1280446	1.5352050	1.9649540
H	-1.0860513	-0.6293112	2.3215029
H	-2.3035555	-1.0330051	1.0973835
C	-0.0153027	-1.8968330	-0.1695957
H	4.3608126	1.5099566	-1.4443634
H	0.1897290	0.6746618	-1.1450717
C	1.8299937	0.3818553	0.1842880
H	4.2178813	1.4890640	0.3395939
O	0.8858244	-1.9863752	-0.9984394
C	-0.7830725	-3.0925796	0.3197577
H	-0.7931612	-3.1366190	1.4109678
H	-0.3187794	-3.9914416	-0.0791664
H	-1.8210285	-3.0452062	-0.0199551
O	2.3119072	-0.1250385	1.1641328
O	2.5334812	0.9826859	-0.7694562
C	3.9513827	0.9804098	-0.5877292
H	4.3250442	-0.0436444	-0.5579511
H	-2.3391622	1.2104944	0.2373707

Ac-Pro-OMe (1-OMe) Ψ = -35.6°_exo trans

H	-0.2224995	2.5302047	0.3031958
C	-0.2624237	1.5676851	0.8110391
C	-1.6768976	1.0634425	1.0707423
C	-1.4726700	-0.4298005	1.2754368
N	-0.3814047	-0.7314569	0.3596476
C	0.3590046	0.4504563	-0.0390291
H	0.2808662	1.6554020	1.7559427
H	-2.1486684	1.5425792	1.9288625
H	-1.1833887	-0.6519839	2.3094533
H	-2.3609381	-1.0145035	1.0352567
C	-0.0474352	-1.9377806	-0.1381225
H	3.6095060	-1.0196224	2.5019563
H	0.2306338	0.6410682	-1.1077967
C	1.8510579	0.3565931	0.1916179

H	4.0665442	-0.9359286	0.7746329
O	0.8844141	-2.0542909	-0.9282298
C	-0.8664771	-3.1116693	0.3208229
H	-0.9239819	-3.1534004	1.4107066
H	-0.4103208	-4.0234293	-0.0577283
H	-1.8877397	-3.0375005	-0.0619589
O	2.6627002	0.9075796	-0.5083255
O	2.1539519	-0.3207630	1.2930481
C	3.5467413	-0.4312659	1.5897744
H	3.9848716	0.5560783	1.7410956
H	-2.3058240	1.2337348	0.1928732

Ac-Pro-OMe (1-OMe) Ψ = 157.2°_exo cis

H	-0.2096206	2.5438163	0.1537816
C	-0.2095175	1.6056416	0.7069099
C	-1.5974108	1.0807702	1.0491897
C	-1.3478245	-0.3972299	1.2933028
N	-0.3116558	-0.7136689	0.3193624
C	0.4110294	0.4621358	-0.1195538
H	0.3684681	1.7485287	1.6241723
H	-2.0342825	1.5822875	1.9129946
H	-0.9858900	-0.5822057	2.3108965
H	-2.2177738	-1.0304661	1.1244380
C	-0.1422601	-1.9705226	-0.1460845
H	4.3452715	1.9956997	-1.1769457
H	0.2654018	0.6440693	-1.1882143
C	1.8976262	0.3934812	0.1611629
H	4.1751750	1.6640560	0.5735029
O	-0.8090287	-2.9031108	0.2883132
C	0.9002770	-2.1782133	-1.2110592
H	0.9089204	-1.3813193	-1.9570168
H	0.7050184	-3.1306985	-1.6988291
H	1.8892528	-2.2202208	-0.7480428
O	2.4110105	-0.2637249	1.0283402
O	2.5622227	1.2056068	-0.6555456
C	3.9731906	1.2961110	-0.4329368
H	4.4393183	0.3186728	-0.5604125
H	-2.2723753	1.2086161	0.1986522

Ac-Pro-OMe (1-OMe) Ψ = -31.2°_exo cis

H	-0.0910348	2.5063865	0.2451409
C	-0.1501524	1.5492148	0.7611281
C	-1.5689295	1.0846242	1.0608845
C	-1.3985380	-0.4115313	1.2583485
N	-0.3587154	-0.7469109	0.2944498
C	0.4239862	0.4089210	-0.1011179
H	0.4189464	1.6262709	1.6916495
H	-1.9970131	1.5784532	1.9334202
H	-1.0682492	-0.6490240	2.2759151
H	-2.2952257	-0.9941079	1.0508576
C	-0.2328617	-1.9968061	-0.2010748
H	3.6622858	-1.1868676	2.3843352
H	0.2950354	0.6324432	-1.1636933
C	1.9151770	0.3043742	0.1368141
H	4.1631585	-0.9594789	0.6816401
O	-0.9429969	-2.9130615	0.1979976
C	0.8208280	-2.2163391	-1.2529209
H	0.8616859	-1.4088904	-1.9859823
H	0.6101935	-3.1568760	-1.7570640
H	1.8035355	-2.2924214	-0.7799327
O	2.7280095	0.8877692	-0.5354956
O	2.2156497	-0.4428741	1.1911491
C	3.6056825	-0.5343003	1.5170021
H	4.0054352	0.4525621	1.7522395
H	-2.2199910	1.2750777	0.2034696

Ac-Pro-NMe_2 (1-NMe_2) Ψ = 159.1°_endo trans

H	2.0634280	1.2935899	0.3359647
C	1.7456295	0.6027225	-0.4458192
C	1.4849951	-0.8118977	0.0840889
N	0.5433017	-1.3448388	-0.8739663
C	-0.0735443	-0.3176366	-1.7035719
C	0.4071037	0.9831989	-1.0714012
H	2.5304626	0.5619877	-1.2054548
C	0.8581747	-0.7715366	1.4825844
H	2.3785585	-1.4347919	0.0902368
H	-1.1616630	-0.4026196	-1.6946455
H	0.2683890	-0.4080939	-2.7408152
H	0.4909196	1.7910702	-1.7986225
C	0.2556474	-2.6594069	-0.8797295
O	0.8041236	-3.4347941	-0.1010194
C	-0.7653355	-3.1179167	-1.8836114
H	-0.7892590	-4.2052464	-1.8879708
H	-0.5385057	-2.7491128	-2.8862615
H	-1.7537443	-2.7398199	-1.6086636
O	-0.3589363	-0.7400310	1.6092019
H	-0.2914178	1.2916215	-0.2913816
N	1.7073612	-0.7214101	2.5306332
C	1.1851954	-0.7450468	3.8789270
H	1.5178317	-1.6495437	4.3977807
H	1.5393405	0.1256553	4.4378152
H	0.0992910	-0.7334328	3.8418424
C	3.1478287	-0.8006435	2.4163272
H	3.5004397	-1.8374327	2.4399046
H	3.5090836	-0.3243817	1.5076041
H	3.5919082	-0.2696829	3.2598850

Ac-Pro-NMe_2 (1-NMe_2) Ψ = -18.2°_endo trans

H	1.9961279	1.3007595	0.2478805
C	1.7803184	0.5542612	-0.5154003
C	1.2764693	-0.7605404	0.0922048
N	0.4333005	-1.3085818	-0.9530635
C	0.1977552	-0.3721031	-2.0469161
C	0.6703340	0.9591504	-1.4778776
H	2.7032002	0.3595767	-1.0664673
C	0.7118312	-0.5651725	1.5046653
H	2.1044252	-1.4473440	0.2770614
H	-0.8557125	-0.3564191	-2.3369434
H	0.7839523	-0.6554257	-2.9279278
H	1.0072975	1.6416100	-2.2581761
C	0.0663701	-2.6065175	-0.9305472
O	0.3490251	-3.3298262	0.0186221
C	-0.7201396	-3.1007729	-2.1128511
H	-0.8891989	-4.1688706	-1.9987252
H	-0.1909404	-2.9103027	-3.0493793
H	-1.6843647	-2.5884725	-2.1721630
O	1.5614745	-0.4322233	2.3820624
H	-0.1414835	1.4513149	-0.9355271
N	-0.6067328	-0.5331759	1.7832062
C	-1.0190230	-0.5048419	3.1727161
H	-1.4863014	0.4542043	3.4162941
H	-1.7464758	-1.3012629	3.3512843
H	-0.1539765	-0.6524392	3.8123830
C	-1.7062892	-0.5794843	0.8425574
H	-2.5460936	-0.0383056	1.2815115
H	-1.4650159	-0.0935149	-0.0959745
H	-2.0282774	-1.6070698	0.6468751

Ac-Pro-NMe_2 (1-NMe_2) Ψ = 167.0°_endo cis

H	2.1841386	1.2195129	0.3951378
C	1.8287713	0.5555350	-0.3931215
C	1.5288863	-0.8622795	0.1134540
N	0.5783185	-1.3487719	-0.8604614
C	-0.0310432	-0.2815026	-1.6446469
C	0.4940990	0.9884140	-0.9898192
H	2.5989248	0.5010690	-1.1666713
C	0.9177839	-0.8237226	1.5229216
H	2.4214372	-1.4889073	0.1095884
H	-1.1184159	-0.3645523	-1.6156019
H	0.2811554	-0.3654390	-2.6906689
H	0.5951216	1.8110411	-1.6977832
C	0.1984158	-2.6306755	-1.0233730
O	-0.6325502	-2.9412398	-1.8712691
C	0.8470810	-3.6552975	-0.1313334
H	0.4430820	-4.6339717	-0.3794632
H	0.6451964	-3.4378876	0.9198122
H	1.9309185	-3.6693342	-0.2712540
O	-0.2917883	-0.9342235	1.6683303
H	-0.1838547	1.3045019	-0.1938709
N	1.7640761	-0.6489347	2.5614566
C	1.2346141	-0.5426783	3.9033865
H	1.7490719	-1.2486789	4.5609432
H	1.3861654	0.4682260	4.2953061
H	0.1715835	-0.7662641	3.8904822

C	3.1903443	-0.4426627	2.4609909
H	3.5572296	-0.5982882	1.4515635
H	3.4518155	0.5761077	2.7664535
H	3.7104325	-1.1397744	3.1240095

Ac-Pro-NMe$_2$ (1-NMe$_2$) Ψ = -15.5°_endo cis

H	2.0348327	1.3028123	0.3762935
C	1.8303177	0.6011794	-0.4314281
C	1.3837606	-0.7683270	0.0976671
N	0.5730299	-1.2895675	-0.9851594
C	0.2531608	-0.2850619	-1.9946823
C	0.6904119	1.0230726	-1.3503834
H	2.7492367	0.4713723	-1.0075474
C	0.8000088	-0.6703597	1.5151788
H	2.2567560	-1.4047418	0.2559599
H	-0.8094461	-0.3182795	-2.2449931
H	0.8090513	-0.4957341	-2.9135840
H	0.9944367	1.7634597	-2.0901594
C	0.2309789	-2.5819988	-1.1679976
O	-0.4055299	-2.9316933	-2.1563959
C	0.6629589	-3.5585490	-0.1080409
H	0.2491714	-4.5351378	-0.3476080
H	0.3231837	-3.2525935	0.8839675
H	1.7531974	-3.6296090	-0.0729645
O	1.6335805	-0.5411906	2.4079770
H	-0.1263334	1.4572612	-0.7670511
N	-0.5183517	-0.7566337	1.7816682
C	-0.9352139	-0.8256745	3.1691430
H	-1.3405697	0.1347461	3.5022133
H	-1.7136937	-1.5854453	3.2693668
H	-0.0863222	-1.0864642	3.7948796
C	-1.6144698	-0.7387342	0.8327939
H	-1.3631389	-0.2058900	-0.0758794
H	-1.9456720	-1.7491607	0.5740983
H	-2.4522692	-0.2154964	1.2969293

Ac-Pro-NMe$_2$ (1-NMe$_2$) Ψ = 132.9°_exo trans

H	-0.5236813	2.6483360	0.4106993
C	-0.4108485	1.6752510	0.8888387
C	-1.7358857	1.0295470	1.2791497
C	-1.3690346	-0.4413934	1.3986012
N	-0.3876283	-0.6073916	0.3386360
C	0.2354447	0.6492868	-0.0497568
H	0.2192123	1.7926247	1.7733460
H	-2.1500406	1.4337764	2.2033930
H	-0.9223096	-0.6585665	2.3759306
H	-2.2217731	-1.1048476	1.2531350
C	-0.0358619	-1.7481507	-0.2772850
H	-0.0109686	0.8565463	-1.0907694
C	1.7455435	0.5844789	0.1779430
O	0.7894727	-1.7430119	-1.1892108
C	-0.6996707	-3.0117927	0.1957042
H	-0.6080849	-3.1257304	1.2779899
H	-0.2321726	-3.8579337	-0.3026251
H	-1.7653707	-2.9985137	-0.0475387
O	2.1605584	0.2072045	1.2683085
H	-2.4753643	1.1618259	0.4844717
N	2.5534443	1.0108517	-0.8105924
C	3.9861339	0.9780032	-0.6097085
H	4.4337116	1.8957777	-0.9968480
H	4.1968844	0.8916190	0.4530227
H	4.4333970	0.1235390	-1.1284940
C	2.0896901	1.2643602	-2.1630633
H	1.5962535	0.3830827	-2.5792666
H	1.4118924	2.1195157	-2.2099499
H	2.9558694	1.4969431	-2.7803868

Ac-Pro-NMe$_2$ (1-NMe$_2$)Ψ = -40.5°_exo trans

H	-0.4714844	2.6831732	0.3940103
C	-0.5053590	1.7212921	0.9037814
C	-1.9098723	1.1458047	1.0373987
C	-1.6560147	-0.3388220	1.2591521
N	-0.4456587	-0.5874935	0.4860657
C	0.2379918	0.6426162	0.1104029
H	-0.0509531	1.8505527	1.8900808
H	-2.4829601	1.5949309	1.8488070
H	-1.4930577	-0.5588009	2.3220679
H	-2.4800303	-0.9624380	0.9120712
C	-0.0350376	-1.7794281	0.0083371
H	0.1079698	0.8092146	-0.9624838
C	1.7530353	0.7125686	0.2875341
O	0.9533344	-1.8663447	-0.7122738
C	-0.8410913	-2.9839116	0.4122858
H	-1.0264023	-3.0037341	1.4883549
H	-0.2991273	-3.8789209	0.1154735
H	-1.8121618	-2.9746922	-0.0900453
O	2.3812330	1.3224934	-0.5715313
H	-2.4638359	1.2902357	0.1061804
N	2.3495807	0.1854742	1.3769447
C	3.7946540	0.1761860	1.4521158
H	4.1509437	-0.8501200	1.5812486
H	4.2062633	0.5889401	0.5355723
H	4.1380323	0.7728763	2.3025498
C	1.6856360	-0.4883346	2.4683375
H	0.6778113	-0.1168386	2.6243757
H	1.6461108	-1.5718404	2.3123033
H	2.2502022	-0.2971932	3.3832515

Ac-Pro-NMe$_2$ (1-NMe$_2$) Ψ = 160.9°_exo cis

H	-0.3624635	2.5133708	-0.1452800
C	-0.2649536	1.6448029	0.5058974
C	-1.5899637	1.1526247	1.0737499
C	-1.2857272	-0.2913123	1.4309064
N	-0.3916902	-0.7011220	0.3601187
C	0.2981910	0.4220302	-0.2446432
H	0.4125112	1.9108270	1.3220478
H	-1.9232011	1.7386289	1.9307924
H	-0.7818707	-0.3680134	2.4014434
H	-2.1598822	-0.9406115	1.4510013
C	-0.2666302	-1.9952220	0.0016129
H	0.0477340	0.4705692	-1.3069074
C	1.8138979	0.3430362	-0.0444926
O	-0.8704155	-2.8826529	0.5969148
C	0.6399086	-2.3060971	-1.1583983
H	0.5335837	-1.5936659	-1.9787795
H	0.4093057	-3.3078992	-1.5141964
H	1.6785471	-2.2799093	-0.8211286
O	2.2720597	-0.3487530	0.8543912
H	-2.3696720	1.1885241	0.3080699
N	2.5931911	1.0794730	-0.8654640
C	4.0265270	1.0821078	-0.6678929
H	4.3588119	2.0474691	-0.2724167
H	4.2963477	0.2973876	0.0331695
H	4.5279982	0.9090255	-1.6236032
C	2.1259567	2.0127936	-1.8647219
H	2.6201931	1.8098347	-2.8188069
H	1.0535983	1.9463574	-2.0173032
H	2.3661225	3.0391615	-1.5681481

Ac-Pro-NMe$_2$ (1-NMe$_2$) Ψ = -38.7°_exo cis

H	-0.1037185	2.4639398	0.2755852
C	-0.2217077	1.5036883	0.7759818
C	-1.6710789	1.1152901	1.0388161
C	-1.5994516	-0.3941369	1.2077252
N	-0.5228606	-0.7802277	0.3036321
C	0.2957238	0.3554167	-0.1009930
H	0.3371428	1.5474285	1.7147046
H	-2.0927517	1.6140967	1.9116309
H	-1.3591305	-0.6755487	2.2401250
H	-2.5155423	-0.9161697	0.9331206
C	-0.4564234	-2.0311913	-0.2016388
H	0.1072077	0.5930471	-1.1519579
C	1.8167865	0.2218996	-0.0356491
O	-1.2195491	-2.9107038	0.1845802
C	0.5981228	-2.3019417	-1.2397811
H	0.6899096	-1.4934070	-1.9669363
H	0.3449036	-3.2292036	-1.7488676
H	1.5721598	-2.4251514	-0.7588984
O	2.4589033	0.7542402	-0.9359285
H	-2.2893059	1.3626155	0.1718053
N	2.4097518	-0.4352886	0.9808034
C	3.8451516	-0.6248705	0.9529545
H	4.0764921	-1.6718568	1.1653254
H	4.2281097	-0.3580914	-0.0282150

H	4.3326075	-0.0013181	1.7085562
C	1.7459493	-0.9967163	2.1376867
H	2.3997077	-0.8698785	3.0028265
H	0.8117815	-0.4913266	2.3567281
H	1.5468687	-2.0658390	2.0121501

Ac-(4S)Flp-OMe (2S-OMe)and Ac-(4S)Flp-NMe_2 (2S-NMe_2)

Ac-(4S)Flp-NMe_2(2S-NMe_2)Ψ = 174.2° endo cis

H	2.1917306	1.1913437	0.4039410
C	1.8616290	0.5029291	-0.3723341
C	1.5213199	-0.9045446	0.1370818
N	0.5637724	-1.3628624	-0.8421393
C	-0.0524867	-0.2776669	-1.5886803
C	0.5761613	0.9760644	-1.0176445
H	2.6423576	0.4363262	-1.1329044
C	0.9196956	-0.9059835	1.5556415
H	2.4031029	-1.5476545	0.1261769
H	-1.1362839	-0.2658636	-1.4583427
H	0.1590348	-0.3798937	-2.6563613
H	0.7250069	1.7579025	-1.7631852
C	0.1721833	-2.6381974	-1.0443446
O	-0.6784803	-2.9047200	-1.8858617
C	0.8371912	-3.6993126	-0.2117586
H	0.4339868	-4.6670690	-0.5004183
H	0.6465281	-3.5292157	0.8497742
H	1.9190378	-3.7008692	-0.3670628
O	-0.2602972	-1.1736647	1.7198114
N	1.7542475	-0.6125048	2.5763233
C	1.2332539	-0.5275896	3.9231379
H	1.7135044	-1.2741217	4.5627073
H	1.4326577	0.4644906	4.3389130
H	0.1613534	-0.7034569	3.9061657
C	3.1655526	-0.3288123	2.4572615
H	3.5503596	-0.5680087	1.4704926
H	3.3701738	0.7273055	2.6628240
H	3.7174891	-0.9289324	3.1859078
F	-0.2540488	1.5132266	-0.0318770

Ac-(4S)Flp-OMe(2S-OMe)Ψ = 173.3° endo trans

H	2.1145785	1.2357955	0.3996237
C	1.8155293	0.5126725	-0.3574274
C	1.4046749	-0.8459499	0.2171769
N	0.4820353	-1.3528380	-0.7689670
C	-0.0034047	-0.3220331	-1.6745214
C	0.5894507	0.9605710	-1.1237879
H	2.6444726	0.3758153	-1.0544149
C	0.7520675	-0.7342310	1.5882349
H	2.2590911	-1.5162386	0.3275808
H	0.4266550	0.5354602	3.8799477
H	-1.0932445	-0.2594296	-1.6871096
H	0.3481143	-0.4941457	-2.6965085
H	0.7951326	1.7010939	-1.8973978
C	0.1470975	-2.6617026	-0.7941604
O	0.6314339	-3.4541666	0.0039562
C	-0.8398225	-3.0742191	-1.8493535
H	-0.9634226	-4.1537882	-1.8103568
H	-0.5023741	-2.7796157	-2.8458465
H	-1.8054959	-2.5936234	-1.6725838
O	-0.4110484	-0.9125941	1.8268915
O	1.6643515	-0.4042842	2.4998909
C	1.1805488	-0.2514888	3.8370688
H	2.0466043	0.0177240	4.4365089
H	0.7466935	-1.1870869	4.1912596
F	-0.3150341	1.5416096	-0.2335206

Ac-(4S)Flp-OMe(2S-OMe)Ψ = 4.4° endo cis

H	2.1627774	1.1843665	0.4755083
C	1.8508015	0.4758941	-0.2897166
C	1.3833238	-0.8647769	0.2841974
N	0.5204415	-1.3711006	-0.7585534
C	0.0541374	-0.3201814	-1.6531725
C	0.6421995	0.9464003	-1.0682519
H	2.6806985	0.3066338	-0.9782120
C	0.7223212	-0.6677731	1.6444472
H	2.2323040	-1.5278020	0.4628509
H	-0.6860436	-1.4694187	3.7238010
H	-1.0356587	-0.2720467	-1.6854470
H	0.4163762	-0.4950941	-2.6697035
H	0.8593806	1.7087701	-1.8163631
C	0.1697259	-2.6584704	-0.9669079
O	-0.5613704	-2.9621681	-1.9017536
C	0.7278257	-3.6821396	-0.0169698
H	0.3185485	-4.6547991	-0.2792092
H	0.4678275	-3.4461674	1.0166107
H	1.8177874	-3.7224845	-0.0886645
O	1.3191729	-0.1963996	2.5795432
O	-0.5334637	-1.0805599	1.6875035
C	-1.2027021	-0.9130182	2.9412372
H	-2.2062804	-1.3027843	2.7928366
H	-1.2371944	0.1430821	3.2099617
F	-0.2740527	1.5035481	-0.1685844

Ac-(4S)Flp-NMe_2 (2S-NMe_2)Ψ = 133.4°exo trans

H	-0.5765379	2.6399416	0.3112580
C	-0.4398754	1.6811262	0.8090732
C	-1.7419730	1.0197566	1.2164380
C	-1.3652287	-0.4443699	1.3739979
N	-0.3981612	-0.6148928	0.3127949
C	0.2270597	0.6391811	-0.0917773
H	0.1590615	1.8248410	1.7115340
H	-2.5209506	1.1463454	0.4596577
H	-0.9200406	-0.5973488	2.3644718
H	-2.2166267	-1.1139081	1.2581673
C	-0.0415134	-1.7626217	-0.2944078
H	0.0025644	0.8178282	-1.1421004
C	1.7337007	0.5871007	0.1718924
O	0.7866361	-1.7545023	-1.2007915
C	-0.7019501	-3.0235815	0.1864378
H	-0.5935130	-3.1380231	1.2672038
H	-0.2438565	-3.8705753	-0.3188166
H	-1.7711237	-3.0071820	-0.0404709
O	2.1208097	0.2232876	1.2762550
N	2.5594374	1.0041480	-0.8037698
C	3.9881908	0.9821564	-0.5729967
H	4.4355673	1.9069809	-0.9431338
H	4.1773813	0.8886450	0.4931185
H	4.4522132	0.1359710	-1.0900990
C	2.1212043	1.2543199	-2.1655495
H	1.6447971	0.3690424	-2.5929569
H	1.4372399	2.1036184	-2.2256667
H	2.9977429	1.4953461	-2.7644914
F	-2.2357214	1.5398243	2.4004985

Ac-(4S)Flp-NMe_2 (2S-NMe_2)Ψ = -38.5° exo cis

H	-0.5233619	2.7455763	0.3819020
C	-0.5236933	1.7761398	0.8764344
C	-1.9026681	1.1646765	1.0279380
C	-1.6330549	-0.3211974	1.1989382
N	-0.4379583	-0.5270579	0.4035319
C	0.2384239	0.7226033	0.0682109
H	-0.0917070	1.8985147	1.8731912
H	-2.5291135	1.3572632	0.1533476
H	-1.4678678	-0.5441700	2.2602632
H	-2.4398965	-0.9602941	0.8434946
C	-0.1478528	-1.7491221	-0.1033146
H	0.1232316	0.9302421	-0.9988869
C	1.7503894	0.8184268	0.2840371
O	-0.8130844	-2.7300967	0.2044164
C	1.0287428	-1.8450657	-1.0340847
H	1.0604817	-1.0277860	-1.7565219
H	0.9725953	-2.7971706	-1.5566926
H	1.9602173	-1.8165948	-0.4624977
O	2.3878185	1.4443944	-0.5562502
N	2.3298175	0.2499220	1.3585472
C	3.7736786	0.2769501	1.4736910
H	4.1349993	-0.7234724	1.7245459
H	4.2079151	0.5962615	0.5303111
H	4.0852540	0.9677569	2.2627980
C	1.6447634	-0.4068275	2.4517669
H	0.6361713	-0.0312598	2.5867341
H	1.6018431	-1.4914009	2.3115920

H	2.1950102	-0.2020078	3.3718262
F	-2.5687950	1.6783385	2.1277117

Ac-(4S)Flp-OMe (2S-OMe)Ψ =-34.8° exo trans

H	-0.2643435	2.5263024	0.2018485
C	-0.2801909	1.5764831	0.7323649
C	-1.6757915	1.0489546	1.0020996
C	-1.4615432	-0.4370086	1.2426641
N	-0.3729237	-0.7361059	0.3359175
C	0.3686272	0.4453492	-0.0724398
H	0.2246502	1.6985537	1.6945440
H	-2.3447882	1.2194869	0.1545258
H	-1.1810681	-0.5951587	2.2910429
H	-2.3477909	-1.0305121	1.0211386
C	-0.0263173	-1.9469557	-0.1509899
H	3.5581494	-0.9571194	2.5795060
H	0.2682183	0.6050750	-1.1483856
C	1.8568324	0.3719450	0.2010327
H	4.0680669	-0.8890577	0.8660577
O	0.9150939	-2.0547323	-0.9276608
C	-0.8417962	-3.1221021	0.3068140
H	-0.8975475	-3.1642743	1.3967778
H	-0.3833922	-4.0323546	-0.0722743
H	-1.8635266	-3.0501636	-0.0750556
O	2.6785366	0.9233328	-0.4852926
O	2.1331813	-0.2899322	1.3169064
C	3.5180661	-0.3807371	1.6586382
H	3.9387233	0.6138462	1.8110717
F	-2.2492401	1.6542075	2.1056931

Ac-(4S)Flp-NMe$_2$(2S-NMe$_2$)Ψ =170.5° endo trans

H	2.1528812	1.1286180	0.3898376
C	1.8200323	0.4520227	-0.3962554
C	1.4811385	-0.9601720	0.0912756
N	0.5115971	-1.4030093	-0.8814346
C	-0.1245042	-0.3066261	-1.5926230
C	0.5337357	0.9417508	-1.0303666
H	2.5991410	0.3986091	-1.1596559
C	0.8731950	-0.9849622	1.5043859
H	2.3414245	-1.6291142	0.0602721
H	-1.2021418	-0.2664057	-1.4175991
H	0.0513167	-0.3812685	-2.6695171
H	0.6864534	1.7148385	-1.7844997
C	0.2130574	-2.7124803	-1.0102658
O	0.7980748	-3.5663420	-0.3537390
C	-0.8621877	-3.0538717	-2.0033336
H	-0.9528529	-4.1357556	-2.0638976
H	-0.6334114	-2.6499009	-2.9923826
H	-1.8177828	-2.6281432	-1.6863535
O	-0.3248136	-1.1665802	1.6537776
N	1.7197642	-0.7857935	2.5386248
C	1.2021539	-0.7380482	3.8876455
H	1.6705633	-1.5154658	4.4985121
H	1.4181471	0.2346894	4.3403095
H	0.1273834	-0.8951746	3.8658802
C	3.1426888	-0.5661956	2.4314407
H	3.6719513	-1.2617704	3.0891429
H	3.5057345	-0.7212998	1.4202653
H	3.3999577	0.4534683	2.7379949
F	-0.2819439	1.4998588	-0.0465554

Ac-(4S)Flp-NMe$_2$(2S-NMe$_2$)Ψ =-7.9° endo cis

H	1.8777834	1.2200203	0.4997430
C	1.7356001	0.5288535	-0.3290583
C	1.3570150	-0.8862603	0.1269943
N	0.5841188	-1.3895482	-0.9919445
C	0.1989184	-0.3472345	-1.9334127
C	0.6046527	0.9364184	-1.2440993
H	2.6593441	0.4900011	-0.9097409
C	0.7771998	-0.8925078	1.5510494
H	2.2636812	-1.4815941	0.2525224
H	-0.8714235	-0.3687337	-2.1487492
H	0.7303755	-0.4716705	-2.8810695
H	0.8592568	1.7362601	-1.9393789
C	0.3558802	-2.6886570	-1.2840601
O	-0.2444056	-3.0038290	-2.3046823
C	0.8713313	-3.7073392	-0.3057191
H	0.5102361	-4.6884493	-0.6044718
H	0.5458155	-3.4916330	0.7141132
H	1.9646873	-3.7139422	-0.3064523
O	1.5995982	-0.6486680	2.4301181
N	-0.5002265	-1.2056546	1.8354247
C	-0.8672202	-1.3681140	3.2296183
H	-1.3779955	-0.4759640	3.6050469
H	-1.5427487	-2.2212389	3.3204284
H	0.0234613	-1.5411998	3.8272546
C	-1.6097162	-1.3081827	0.9055682
H	-1.4805068	-0.6596450	0.0482307
H	-1.7681891	-2.3374368	0.5700768
H	-2.5110818	-0.9805958	1.4256407
F	-0.4649365	1.4006791	-0.4671841

Ac-(4S)Flp-OMe (2S-OMe)Ψ =-3.0° endo trans

H	2.0402761	1.1912243	0.4512320
C	1.7590201	0.4825991	-0.3260220
C	1.3352580	-0.8818219	0.2221882
N	0.4530994	-1.3852124	-0.8058861
C	-0.0068442	-0.3432562	-1.7127425
C	0.5470582	0.9333500	-1.1111775
H	2.6021913	0.3563971	-1.0077697
C	0.7051027	-0.7561801	1.6015564
H	2.1877798	-1.5502771	0.3552505
H	-0.7276135	-1.6362779	3.6270868
H	-1.0959186	-0.2940567	-1.7717022
H	0.3909485	-0.4899743	-2.7216129
H	0.7548746	1.7023831	-1.8555781
C	0.1519078	-2.6999597	-0.8828075
O	0.6098025	-3.5028576	-0.0795030
C	-0.7696947	-3.1056681	-1.9983205
H	-0.8755800	-4.1877880	-1.9887771
H	-0.3838868	-2.7842390	-2.9686481
H	-1.7523216	-2.6454256	-1.8659910
O	1.3440146	-0.3961116	2.5593002
O	-0.5781972	-1.0707578	1.6342674
C	-1.2103671	-0.9692279	2.9122112
H	-2.2444543	-1.2644238	2.7538314
H	-1.1568582	0.0558843	3.2804618
F	-0.3924189	1.4654631	-0.2238378

Ac-(4S)Flp-OMe (2S-OMe)Ψ =160.1° exo cis

H	-0.2531517	2.5100188	0.0071127
C	-0.2204700	1.5986895	0.6006001
C	-1.5859380	1.0572555	0.9787393
C	-1.3239739	-0.4076077	1.2751982
N	-0.3014978	-0.7367651	0.3029228
C	0.4256366	0.4298741	-0.1607416
H	0.3245144	1.7998721	1.5270468
H	-2.3095580	1.1749978	0.1678415
H	-0.9601517	-0.5157632	2.3034840
H	-2.1932405	-1.0491153	1.1431841
C	-0.1386525	-2.0045605	-0.1458438
H	4.3598574	2.0395883	-1.0922030
H	0.3096222	0.5664695	-1.2389383
C	1.9074556	0.3791346	0.1607249
H	4.1537006	1.6763229	0.6482196
O	-0.8092760	-2.9234821	0.3050586
C	0.9019331	-2.2279119	-1.2079644
H	0.8865575	-1.4588208	-1.9824364
H	0.7246580	-3.2013246	-1.6594510
H	1.8945722	-2.2299424	-0.7510880
O	2.4040269	-0.2877221	1.0292380
O	2.5776870	1.2129394	-0.6260064
C	3.9824562	1.3218986	-0.3685946
H	4.4661813	0.3538886	-0.5012513
F	-2.0985965	1.7124685	2.0841017

Ac-(4S)Flp-NMe$_2$(2S-NMe$_2$)Ψ =-39.7° exo trans

H	-0.5004242	2.6873963	0.3191151
C	-0.5183494	1.7336137	0.8427188
C	-1.9051119	1.1388083	0.9779583
C	-1.6498004	-0.3417441	1.2231758
N	-0.4329851	-0.5842237	0.4733939
C	0.2474857	0.6475806	0.0866905

H	-0.1071150	1.8813960	1.8451506
H	-2.5002448	1.2927794	0.0742684
H	-1.5158636	-0.5076437	2.3000730
H	-2.4703272	-0.9700560	0.8786103
C	-0.0099244	-1.7778464	0.0010448
H	0.1405176	0.7881410	-0.9917854
C	1.7592865	0.7406526	0.2940308
O	0.9871137	-1.8522231	-0.7053118
C	-0.8126862	-2.9854145	0.3975078
H	-1.0057675	-3.0068324	1.4721417
H	-0.2643294	-3.8775153	0.1041483
H	-1.7798010	-2.9800795	-0.1125120
O	2.3928358	1.3483488	-0.5616301
N	2.3393570	0.2314281	1.3990159
C	3.7830799	0.2445202	1.5039080
H	4.1480876	-0.7721005	1.6755598
H	4.2087102	0.6311178	0.5823864
H	4.1008912	0.8746054	2.3399547
C	1.6608250	-0.4290337	2.4901799
H	0.6511638	-0.0556287	2.6299186
H	1.6240986	-1.5143302	2.3484464
H	2.2125817	-0.2247361	3.4097142
F	-2.6086672	1.6975938	2.0309186

Ac-(4S)Flp-OMe (2S-OMe)Ψ =-176.2° endo cis

H	2.2244757	1.2071430	0.4201607
C	1.8779391	0.4906666	-0.3218843
C	1.4441579	-0.8517495	0.2757175
N	0.5268021	-1.3589723	-0.7158924
C	0.0148713	-0.3118239	-1.5895502
C	0.6336686	0.9576243	-1.0444269
H	2.6745228	0.3212483	-1.0488019
C	0.7923818	-0.7081374	1.6477419
H	2.3044047	-1.5123583	0.4077458
H	0.2870545	0.6596054	3.8506101
H	-1.0750010	-0.2633054	-1.5634260
H	0.3219335	-0.4917765	-2.6233095
H	0.8182542	1.7116070	-1.8097630
C	0.1452843	-2.6439165	-0.8869016
O	-0.6393723	-2.9460647	-1.7775181
C	0.7384016	-3.6650658	0.0438342
H	0.3287204	-4.6400283	-0.2090352
H	0.4998951	-3.4296438	1.0826612
H	1.8264046	-3.6976537	-0.0557722
O	-0.3191313	-1.0685427	1.9278925
O	1.6444785	-0.1622004	2.5091723
C	1.1596771	0.0058679	3.8453777
H	1.9773671	0.4567326	4.4017692
H	0.8910105	-0.9598559	4.2746379
F	-0.2340895	1.5286382	-0.1075196

Ac-(4S)Flp-NMe_2(2S-NMe_2)Ψ =-10.0° endo trans

H	1.8752755	1.1155509	0.4528961
C	1.6896419	0.4326923	-0.3741774
C	1.2460321	-0.9611826	0.0849681
N	0.4057790	-1.4144537	-1.0085029
C	0.0962241	-0.3598279	-1.9634185
C	0.5697910	0.9012403	-1.2731805
H	2.6035597	0.3531666	-0.9663184
C	0.7232618	-0.9484794	1.5283629
H	2.1039804	-1.6310484	0.1678338
H	-0.9709991	-0.3003785	-2.1891211
H	0.6391592	-0.4997399	-2.9034961
H	0.8522821	1.6903843	-1.9702110
C	0.1391312	-2.7283050	-1.1782976
O	0.5052741	-3.5593785	-0.3566388
C	-0.6439529	-3.0959879	-2.4072712
H	-0.7679886	-4.1758382	-2.4331022
H	-0.1329645	-2.7652432	-3.3147277
H	-1.6277541	-2.6192108	-2.3932989
O	1.5960476	-0.7562163	2.3724237
N	-0.5615205	-1.1564676	1.8656148
C	-0.8888568	-1.2989657	3.2709058
H	-1.4273930	-0.4180411	3.6338347
H	-1.5263201	-2.1764985	3.4037930
H	0.0227928	-1.4203237	3.8486506
C	-1.6985065	-1.2548665	0.9725545
H	-2.5820833	-0.9272867	1.5220395
H	-1.5944946	-0.6013294	0.1140408
H	-1.8649355	-2.2845991	0.6430637
F	-0.4710478	1.4076981	-0.4859328

Ac-(4S)Flp-NMe_2(2S-NMe_2)Ψ =163.1° exo cis

H	-0.4491076	2.4458353	-0.3122734
C	-0.2972751	1.6221759	0.3834302
C	-1.5832425	1.1103082	1.0050187
C	-1.2412542	-0.3062377	1.4242664
N	-0.3748600	-0.7314769	0.3475899
C	0.3098086	0.3797961	-0.2898517
H	0.3488240	1.9682422	1.1948312
H	-2.4137040	1.1223832	0.2942520
H	-0.7179006	-0.2903410	2.3879020
H	-2.1024867	-0.9666890	1.5030289
C	-0.2475001	-2.0363996	0.0158061
H	0.0919926	0.3714429	-1.3594495
C	1.8233744	0.3346405	-0.0448045
O	-0.8407147	-2.9090226	0.6379598
C	0.6476088	-2.3619418	-1.1478150
H	0.4763842	-1.7039708	-2.0021255
H	0.4648582	-3.3929421	-1.4417245
H	1.6918893	-2.2583141	-0.8445686
O	2.2663620	-0.3471671	0.8681795
N	2.6068434	1.0839522	-0.8476755
C	4.0324180	1.1324838	-0.6020157
H	4.3214216	2.1145689	-0.2142396
H	4.3005832	0.3691334	0.1228407
H	4.5711935	0.9567208	-1.5364879
C	2.1413966	1.9993395	-1.8647085
H	2.7124022	1.8436684	-2.7835841
H	1.0901085	1.8562399	-2.0942571
H	2.2863404	3.0363044	-1.5441315
F	-1.9615668	1.8846899	2.0885677

Ac-(4S)Flp-OMe (2S-OMe)Ψ =145.6° exo trans

H	-0.3270535	2.5474913	0.1475727
C	-0.3012540	1.6045706	0.6902718
C	-1.6747696	1.0504526	1.0169443
C	-1.4176909	-0.4268279	1.2678020
N	-0.3634878	-0.7123606	0.3179217
C	0.3460120	0.4767843	-0.1194596
H	0.2342637	1.7487579	1.6325527
H	-2.3779649	1.1945079	0.1924142
H	-1.0878072	-0.5644639	2.3044687
H	-2.2987220	-1.0428230	1.0917352
C	-0.0134616	-1.9175543	-0.1800767
H	4.3874285	1.5169001	-1.3931649
H	0.2197676	0.6210372	-1.1946936
C	1.8294919	0.3781492	0.1824210
H	4.2012792	1.5086314	0.3871175
O	0.8932263	-2.0070383	-0.9996801
C	-0.7829072	-3.1077565	0.3160907
H	-0.7872984	-3.1473702	1.4074791
H	-0.3233942	-4.0095342	-0.0814316
H	-1.8222322	-3.0578247	-0.0189514
O	2.2862974	-0.1204481	1.1775898
O	2.5493042	0.9766457	-0.7580192
C	3.9633946	0.9899209	-0.5422591
H	4.3453946	-0.0302867	-0.4955244
F	-2.2211955	1.6584499	2.1326466

Ac-(4S)Flp-OMe (2S-OMe)Ψ =-30.6° exo cis

H	-0.2669299	2.5459401	0.2110715
C	-0.2676823	1.5935744	0.7371780
C	-1.6515665	1.0319904	1.0007832
C	-1.4055894	-0.4505028	1.2121741
N	-0.3228644	-0.7160628	0.2865971
C	0.4174918	0.4823541	-0.0728011
H	0.2277407	1.7231086	1.7032036
H	-2.3290125	1.2069990	0.1609061
H	-1.1075840	-0.6293272	2.2518024
H	-2.2639459	-1.0794318	0.9829471
C	-0.1133621	-1.9559797	-0.2153744
H	3.5730477	-0.9381920	2.6148202

H	0.3439444	0.6863397	-1.1436537
C	1.8970320	0.4620276	0.2585847
H	4.1705352	-0.6796094	0.9478142
O	-0.7932675	-2.9067299	0.1469998
C	0.9951434	-2.1065805	-1.2204466
H	1.0042248	-1.3053960	-1.9614170
H	0.8749641	-3.0647127	-1.7207021
H	1.9613207	-2.1037866	-0.7088072
O	2.7134684	1.0906851	-0.3653051
O	2.1688348	-0.2702906	1.3289861
C	3.5385062	-0.2873955	1.7451870
H	3.8666704	0.7193640	2.0049712
F	-2.2255033	1.6113283	2.1182124

Ac-(4*R*)Flp-OMe (2R-OMe)and Ac-(4*R*)Flp-NMe$_2$ (2R-NMe$_2$)

Ac-(4*R*)Flp-NMe$_2$(2R-NMe$_2$)Ψ =165.4° endo cis

H	2.1180491	1.1740931	0.4298208
C	1.7716645	0.5102708	-0.3613483
C	1.5211462	-0.9243729	0.1094681
N	0.5737787	-1.4156771	-0.8682519
C	-0.1074125	-0.3539682	-1.5859649
C	0.4241302	0.9131680	-0.9325448
H	2.5060919	0.5096980	-1.1703487
C	0.9188518	-0.9368095	1.5252326
H	2.4329181	-1.5201004	0.0775599
H	-1.1893349	-0.4600408	-1.5012541
H	0.1568287	-0.3615083	-2.6481460
H	-0.2516499	1.2759153	-0.1554314
C	0.2129298	-2.7049573	-1.0409309
O	-0.6418203	-3.0128940	-1.8626188
C	0.9186472	-3.7285477	-0.1938272
H	0.5403510	-4.7133030	-0.4572915
H	0.7357403	-3.5457765	0.8675453
H	1.9982979	-3.7026584	-0.3605023
O	-0.2908873	-1.0528549	1.6653804
N	1.7677853	-0.7960564	2.5638303
C	1.2423324	-0.7401441	3.9110931
H	1.7785895	-1.4515659	4.5444276
H	1.3711067	0.2627813	4.3302095
H	0.1851621	-0.9897237	3.8973090
C	3.1915033	-0.5685187	2.4644211
H	3.7243391	-1.2900749	3.0897165
H	3.5540121	-0.6698561	1.4465096
H	3.4410547	0.4376508	2.8170522
F	0.5576759	1.9347257	-1.8606414

Ac-(4*R*)Flp-OMe(2R-OMe)Ψ =152.6° endo trans

H	1.9929546	1.3856461	0.3827807
C	1.7367812	0.6535174	-0.3820940
C	1.4375036	-0.7354349	0.1860075
N	0.5269561	-1.3042424	-0.7860364
C	0.0327974	-0.3366753	-1.7472863
C	0.4686142	0.9913772	-1.1430785
H	2.5627930	0.5861393	-1.0936378
C	0.7789543	-0.6298212	1.5519937
H	2.3363374	-1.3439341	0.2858036
H	0.6999425	0.4391077	3.9632652
H	-1.0485397	-0.4022586	-1.8682393
H	0.5063984	-0.4562235	-2.7284052
H	-0.3023438	1.3982557	-0.4853723
C	0.1457474	-2.5947550	-0.6681964
O	0.5819458	-3.2907555	0.2409806
C	-0.8241978	-3.1078591	-1.6929428
H	-0.9111872	-4.1862517	-1.5831501
H	-0.5046850	-2.8629489	-2.7079041
H	-1.8063964	-2.6538300	-1.5355298
O	-0.3992047	-0.4588831	1.7313580
O	1.6853959	-0.6866391	2.5194594
C	1.1868720	-0.5303243	3.8516911
H	2.0552432	-0.5985711	4.5018651
H	0.4738988	-1.3224876	4.0816098
F	0.7020915	1.9311751	-2.1327604

Ac-(4*R*)Flp-OMe(2R-OMe)Ψ =-22.2° endo cis

H	1.9078855	1.2503046	0.5662067
C	1.6685013	0.5508769	-0.2334546
C	1.3968136	-0.8691921	0.2699121
N	0.5631477	-1.4278062	-0.7736452
C	-0.0195374	-0.4192037	-1.6416159
C	0.3993253	0.8923716	-0.9924628
H	2.4992414	0.5264149	-0.9421311
C	0.7539706	-0.8230247	1.6495485
H	2.3279470	-1.4238427	0.3901179
H	-0.9545836	-1.6055128	3.5018963
H	-1.1008137	-0.5419875	-1.7088675
H	0.3958668	-0.4801417	-2.6523248
H	-0.3787839	1.2790141	-0.3305302
C	0.2634416	-2.7362766	-0.9335974
O	-0.4740127	-3.1036737	-1.8386854
C	0.8827590	-3.6980896	0.0430726
H	0.5534047	-4.7034389	-0.2076016
H	0.5818673	-3.4704868	1.0688038
H	1.9736336	-3.6552326	0.0000278
O	1.3997883	-0.7312607	2.6621215
O	-0.5704192	-0.8532769	1.6034162
C	-1.2438081	-0.7695035	2.8646592
H	-2.3049281	-0.8138120	2.6345161
H	-0.9965431	0.1682889	3.3627015
F	0.6330467	1.8661517	-1.9494011

Ac-(4*R*)Flp-NMe$_2$(2R-NMe$_2$)Ψ =131.1° exo trans

H	-0.5713423	2.6450991	0.4357937
C	-0.4113017	1.6722123	0.8985430
C	-1.7086287	1.0175921	1.3143510
C	-1.3580623	-0.4501381	1.3968471
N	-0.3746694	-0.6055763	0.3412463
C	0.2389523	0.6564528	-0.0474576
H	0.2134447	1.7970322	1.7855836
H	-2.1345925	1.4152181	2.2361863
H	-0.9351116	-0.6783661	2.3815385
H	-2.2363451	-1.0750669	1.2353028
C	-0.0323021	-1.7444737	-0.2880708
H	-0.0120732	0.8662790	-1.0859756
C	1.7500687	0.6063109	0.1849083
O	0.7910813	-1.7336439	-1.1992647
C	-0.7050180	-3.0059282	0.1758478
H	-0.6083223	-3.1321975	1.2563518
H	-0.2487219	-3.8513586	-0.3336701
H	-1.7717819	-2.9791784	-0.0615839
O	2.1595617	0.2640085	1.2891775
N	2.5571773	1.0027454	-0.8139631
C	3.9895942	0.9924930	-0.6072511
H	4.4261332	1.9093793	-1.0087959
H	4.1978250	0.9270787	0.4574361
H	4.4486467	0.1354102	-1.1106616
C	2.0940983	1.2260194	-2.1726351
H	1.5757504	0.3452371	-2.5569864
H	1.4401096	2.0980047	-2.2442036
H	2.9639292	1.4134279	-2.7998994
F	-2.6615513	1.1983846	0.3070753

Ac-(4*R*)Flp-NMe$_2$(2R-NMe$_2$)Ψ =-38.5° exo cis

H	-0.1681363	2.4655384	0.2951466
C	-0.2333898	1.4970083	0.7876963
C	-1.6609208	1.0867283	1.0718274
C	-1.5885274	-0.4158475	1.2090567
N	-0.5067998	-0.7864816	0.3096668
C	0.2998399	0.3583464	-0.0925786
H	0.3146867	1.5577361	1.7308953
H	-2.1030298	1.5821272	1.9368070
H	-1.3719471	-0.6995689	2.2453483
H	-2.5180186	-0.9035381	0.9166443
C	-0.4432475	-2.0334976	-0.2117918
H	0.1110041	0.5967855	-1.1422785
C	1.8228088	0.2406155	-0.0205686
O	-1.2088396	-2.9114403	0.1680129
C	0.6114133	-2.2909890	-1.2514282
H	0.6800939	-1.4850524	-1.9841517
H	0.3753241	-3.2266116	-1.7530258
H	1.5906446	-2.3883943	-0.7752279
O	2.4619051	0.7798144	-0.9176754
N	2.4153635	-0.4137074	0.9975254

C	3.8525272	-0.5935732	0.9747504
H	4.0898510	-1.6388563	1.1882199
H	4.2368674	-0.3247296	-0.0052932
H	4.3327473	0.0339085	1.7315509
C	1.7503405	-0.9795411	2.1519269
H	2.4072514	-0.8630247	3.0158418
H	0.8208003	-0.4687810	2.3795729
H	1.5428945	-2.0460797	2.0199599
F	-2.4497858	1.4032677	-0.0363471

Ac-(4*R*)Flp-OMe(2R-OMe)Ψ =-35.8° exo trans

H	-0.2846283	2.5309103	0.2947118
C	-0.2709079	1.5701016	0.8057390
C	-1.6610707	1.0513407	1.0987936
C	-1.4618155	-0.4366832	1.2778697
N	-0.3669740	-0.7310024	0.3693152
C	0.3630266	0.4547642	-0.0374753
H	0.2639997	1.6840045	1.7517720
H	-2.1451241	1.5303890	1.9504641
H	-1.1977405	-0.6586654	2.3177994
H	-2.3679380	-0.9857938	1.0214100
C	-0.0324015	-1.9376538	-0.1334403
H	3.6109115	-0.9603973	2.5356851
H	0.2341573	0.6371856	-1.1066639
C	1.8562175	0.3771207	0.2001553
H	4.0760154	-0.8950800	0.8095995
O	0.9034844	-2.0481881	-0.9168434
C	-0.8580230	-3.1097580	0.3156445
H	-0.9180872	-3.1591463	1.4050888
H	-0.4063324	-4.0213870	-0.0681807
H	-1.8778253	-3.0265953	-0.0693764
O	2.6646917	0.9258985	-0.5037233
O	2.1571686	-0.2850300	1.3106796
C	3.5497895	-0.3835834	1.6161788
H	3.9805472	0.6083775	1.7573691
F	-2.4732552	1.2661939	-0.0160273

Ac-(4*R*)Flp-NMe_2(2R-NMe_2)Ψ =130.6° endo trans

H	1.7426668	1.6753161	0.1733839
C	1.5265516	0.8905331	-0.5505476
C	1.5039260	-0.5070025	0.0674174
N	0.5947416	-1.2348052	-0.8053759
C	-0.1802164	-0.3744104	-1.6789994
C	0.1560103	1.0286600	-1.1873970
H	2.2777428	0.9255844	-1.3434160
C	0.9331324	-0.4483573	1.4862519
H	2.4849761	-0.9735155	0.0378343
H	-1.2476634	-0.5884115	-1.6134989
H	0.1344228	-0.4700064	-2.7242169
H	-0.5864951	1.3917863	-0.4780191
C	0.4817785	-2.5721462	-0.6919634
O	1.1563560	-3.1924354	0.1255684
C	-0.5024467	-3.2448133	-1.6056489
H	-0.3901305	-4.3222607	-1.5105608
H	-0.3515547	-2.9475221	-2.6456063
H	-1.5223330	-2.9656700	-1.3273817
O	-0.2143005	-0.0362575	1.6366007
N	1.7340596	-0.7933140	2.5078470
C	1.2602223	-0.6376411	3.8666296
H	1.0588168	-1.6132446	4.3195760
H	2.0173036	-0.1247475	4.4644962
H	0.3437164	-0.0535651	3.8626724
C	3.0298361	-1.4283042	2.3388481
H	2.9691965	-2.2435054	1.6174744
H	3.8002721	-0.7152247	2.0326538
H	3.3264550	-1.8507175	3.2979326
F	0.1836872	1.9214959	-2.2516788

Ac-(4*R*)Flp-NMe_2(2R-NMe_2)Ψ =-16.9° endo cis

H	1.7353165	1.2339081	0.5263212
C	1.6252263	0.5565103	-0.3184958
C	1.3468290	-0.8865171	0.1129522
N	0.5892510	-1.4271242	-1.0017915
C	0.1499682	-0.4142653	-1.9449503
C	0.4627054	0.8929952	-1.2316113
H	2.5404948	0.5883217	-0.9138310
C	0.7764116	-0.9623511	1.5387097
H	2.2925329	-1.4206882	0.2199771
H	-0.9060239	-0.5366504	-2.1916680
H	0.7206766	-0.4695776	-2.8773608
H	-0.3984770	1.2699769	-0.6731448
C	0.3855615	-2.7384183	-1.2619077
O	-0.2210526	-3.0897129	-2.2660543
C	0.9340150	-3.7215817	-0.2654294
H	0.6441270	-4.7232160	-0.5730910
H	0.5556182	-3.5267401	0.7406108
H	2.0244448	-3.6600251	-0.2232758
O	1.6059844	-0.8189822	2.4316852
N	-0.5224713	-1.2028323	1.8007578
C	-0.9150033	-1.4229024	3.1800729
H	-1.4042779	-0.5350063	3.5918168
H	-1.6166261	-2.2588804	3.2209504
H	-0.0377343	-1.6519125	3.7785878
C	-1.6224960	-1.2107936	0.8560001
H	-2.5124438	-0.8521240	1.3750224
H	-1.4517692	-0.5452301	0.0177934
H	-1.8312378	-2.2154795	0.4767876
F	0.8010167	1.8797604	-2.1425493

Ac-(4*R*)Flp-OMe(2R-OMe)Ψ =-28.5° endo trans

H	1.8575131	1.2275007	0.5891867
C	1.6453092	0.5602130	-0.2450417
C	1.3839816	-0.8836924	0.1907627
N	0.5064293	-1.3912226	-0.8460226
C	0.0119083	-0.3598982	-1.7383201
C	0.3878260	0.9239451	-1.0111361
H	2.4909510	0.5839863	-0.9360407
C	0.7814738	-0.9385616	1.5849246
H	2.3038999	-1.4662931	0.2452396
H	-0.9464954	-1.8021722	3.3758961
H	-1.0625885	-0.4460986	-1.9009543
H	0.5186258	-0.3814740	-2.7098184
H	-0.4134830	1.2511091	-0.3449903
C	0.1583174	-2.6962513	-0.8494783
O	0.5849320	-3.4550732	0.0125523
C	-0.7677717	-3.1450517	-1.9429701
H	-0.8279771	-4.2307123	-1.9263028
H	-0.4263617	-2.8077506	-2.9237130
H	-1.7666462	-2.7311305	-1.7792446
O	1.4517833	-0.9939106	2.5837227
O	-0.5427394	-0.8499981	1.5763504
C	-1.1761297	-0.8703703	2.8584325
H	-2.2427739	-0.7966716	2.6622232
H	-0.8382053	-0.0277837	3.4626297
F	0.6153996	1.9511477	-1.9108774

Ac-(4*R*)Flp-OMe(2R-OMe)Ψ =155.8° exo cis

H	-0.2454167	2.5446157	0.1823391
C	-0.2064547	1.6000168	0.7216251
C	-1.5780017	1.0715312	1.0742337
C	-1.3512738	-0.4053566	1.2832710
N	-0.2992925	-0.7146737	0.3282594
C	0.4172493	0.4646558	-0.1117218
H	0.3571390	1.7458708	1.6463554
H	-2.0366980	1.5696486	1.9288926
H	-1.0321734	-0.6036535	2.3117350
H	-2.2432797	-0.9963868	1.0790859
C	-0.1386651	-1.9708313	-0.1482977
H	4.3501274	1.9712004	-1.2119285
H	0.2660406	0.6495516	-1.1783213
C	1.9058356	0.4017171	0.1674033
H	4.1860023	1.6751911	0.5457443
O	-0.8179144	-2.8961314	0.2779476
C	0.9094807	-2.1763219	-1.2065367
H	0.9062612	-1.3866568	-1.9603169
H	0.7286095	-3.1361500	-1.6849946
H	1.8982658	-2.1987861	-0.7416579
O	2.4163422	-0.2376425	1.0491197
O	2.5681580	1.1936023	-0.6679627
C	3.9807333	1.2872482	-0.4523889
H	4.4444986	0.3066392	-0.5621490
F	-2.4275289	1.2530084	-0.0194697

Ac-(4*R*)Flp-NMe$_2$(2R-NMe$_2$)Ψ =-39.9° exo trans

H	-0.5464389	2.6898688	0.3820947
C	-0.5226260	1.7328014	0.9000721
C	-1.9024332	1.1380507	1.0719072
C	-1.6412925	-0.3389313	1.2727571
N	-0.4322915	-0.5764881	0.5005201
C	0.2366829	0.6587240	0.1147895
H	-0.0782515	1.8902932	1.8860894
H	-2.4878510	1.5862501	1.8754469
H	-1.4956249	-0.5543546	2.3383651
H	-2.4820950	-0.9334961	0.9154157
C	-0.0210133	-1.7668087	0.0120236
H	0.1066385	0.8148997	-0.9586057
C	1.7507211	0.7513110	0.2979682
O	0.9668429	-1.8434440	-0.7072810
C	-0.8282402	-2.9726208	0.4066322
H	-1.0074299	-3.0044920	1.4834873
H	-0.2912727	-3.8660322	0.0967077
H	-1.8018968	-2.9537823	-0.0905211
O	2.3721380	1.3653575	-0.5618486
N	2.3485979	0.2341830	1.3908132
C	3.7938631	0.2416346	1.4707287
H	4.1594926	-0.7786377	1.6183981
H	4.2041161	0.6428242	0.5484703
H	4.1282174	0.8565675	2.3116055
C	1.6874153	-0.4368780	2.4859172
H	0.6808500	-0.0632457	2.6468284
H	1.6454397	-1.5205586	2.3332757
H	2.2552488	-0.2437974	3.3981442
F	-2.6228706	1.3093762	-0.1114106

Ac-(4*R*)Flp-OMe(2R-OMe)Ψ =164.7° endo cis

H	2.0698686	1.2928798	0.4319715
C	1.7637359	0.5710691	-0.3236829
C	1.4363304	-0.8077991	0.2557839
N	0.5365668	-1.3684729	-0.7269554
C	-0.0224587	-0.3712126	-1.6231812
C	0.4818341	0.9433743	-1.0459203
H	2.5623783	0.4712964	-1.0620023
C	0.7831325	-0.6660173	1.6245001
H	2.3409906	-1.4061444	0.3728916
H	0.5783980	0.6302449	3.9162608
H	-1.1098770	-0.4413667	-1.6525702
H	0.3578266	-0.4974894	-2.6418831
H	-0.2498236	1.3904373	-0.3689436
C	0.1673793	-2.6660324	-0.8190902
O	-0.6144245	-3.0334798	-1.6859106
C	0.7682957	-3.6164661	0.1798849
H	0.3950896	-4.6160779	-0.0289135
H	0.4909937	-3.3382827	1.1994726
H	1.8590374	-3.6193288	0.1164149
O	-0.4012234	-0.7006828	1.8319934
O	1.7057779	-0.4681410	2.5583722
C	1.2166151	-0.2533234	3.8872512
H	2.0981676	-0.1084982	4.5061463
H	0.6496971	-1.1211140	4.2253086
F	0.7203971	1.8678253	-2.0493446

Ac-(4*R*)Flp-NMe$_2$(2R-NMe$_2$)Ψ =-19.6° endo trans

H	1.7736818	1.1423491	0.5198654
C	1.6113949	0.4846376	-0.3320155
C	1.2633887	-0.9477580	0.0830370
N	0.4227834	-1.4199790	-1.0044232
C	0.0622256	-0.3770638	-1.9466460
C	0.4484967	0.8988937	-1.2103116
H	2.5146113	0.4806747	-0.9464655
C	0.7579522	-1.0294706	1.5298409
H	2.1636265	-1.5624104	0.1284227
H	-0.9982118	-0.4079538	-2.2046172
H	0.6457707	-0.4413748	-2.8717276
H	-0.3849107	1.2948455	-0.6236186
C	0.1688460	-2.7398655	-1.1492958
O	0.5593712	-3.5481784	-0.3166731
C	-0.6240288	-3.1399398	-2.3611024
H	-0.7002176	-4.2242329	-2.3869862
H	-0.1514193	-2.7826364	-3.2789285
H	-1.6284541	-2.7096203	-2.3208673
O	1.6390388	-0.9642792	2.3821709
N	-0.5428970	-1.1545115	1.8559795
C	-0.8876929	-1.3824739	3.2458815
H	-1.4007258	-0.5109392	3.6634764
H	-1.5540531	-2.2461097	3.3158425
H	0.0152597	-1.5725066	3.8185249
C	-1.6790791	-1.1317320	0.9593210
H	-2.5365108	-0.7479669	1.5141578
H	-1.5248322	-0.4705551	0.1134727
H	-1.9294016	-2.1328773	0.5953248
F	0.8054030	1.8900983	-2.1083520

Ac-(4*R*)Flp-NMe$_2$(2R-NMe$_2$)Ψ =160.0° exo cis

H	-0.3981827	2.5182788	-0.1027714
C	-0.2632421	1.6406133	0.5279253
C	-1.5710860	1.1428551	1.0994911
C	-1.2936709	-0.3042187	1.4208952
N	-0.3793854	-0.7008665	0.3660783
C	0.3042141	0.4284211	-0.2342292
H	0.3990797	1.9083559	1.3548578
H	-1.9299674	1.7210406	1.9516058
H	-0.8318212	-0.3948264	2.4101705
H	-2.1941601	-0.9167835	1.4021336
C	-0.2615497	-1.9938467	-0.0055321
H	0.0493091	0.4841020	-1.2941988
C	1.8213528	0.3564551	-0.0336014
O	-0.8779100	-2.8775141	0.5794240
C	0.6532922	-2.2973399	-1.1595151
H	0.5350046	-1.5906160	-1.9833230
H	0.4393879	-3.3042542	-1.5107593
H	1.6908524	-2.2520168	-0.8208547
O	2.2775988	-0.3238545	0.8747550
N	2.5974358	1.0838318	-0.8630799
C	4.0327329	1.0774517	-0.6782626
H	4.3749674	2.0429072	-0.2922119
H	4.3037691	0.2950553	0.0249166
H	4.5232422	0.8948800	-1.6378107
C	2.1290352	2.0015239	-1.8767202
H	2.5742348	1.7482280	-2.8429639
H	1.0493089	1.9797877	-1.9827765
H	2.4246654	3.0242858	-1.6222377
F	-2.5541796	1.2108805	0.1081022

Ac-(4*R*)Flp-OMe(2R-OMe)Ψ =143.0° exo trans

H	-0.3375513	2.5605971	0.2734162
C	-0.2864214	1.6000395	0.7829650
C	-1.6555151	1.0528646	1.1203069
C	-1.4181580	-0.4299012	1.2951317
N	-0.3497062	-0.7011822	0.3494117
C	0.3461762	0.4974445	-0.0760902
H	0.2762506	1.7219191	1.7117630
H	-2.1222083	1.5233060	1.9863317
H	-1.1126269	-0.6429552	2.3254111
H	-2.3199568	-0.9998141	1.0707681
C	-0.0020355	-1.8991292	-0.1643219
H	4.3653275	1.4758927	-1.4688910
H	0.1904972	0.6728494	-1.1427166
C	1.8354616	0.3949980	0.1898112
H	4.2245686	1.5003077	0.3154031
O	0.9069320	-1.9846730	-0.9823622
C	-0.7801354	-3.0917733	0.3137615
H	-0.7950629	-3.1431831	1.4046489
H	-0.3211948	-3.9915610	-0.0888860
H	-1.8161320	-3.0336034	-0.0304066
O	2.3123701	-0.0849159	1.1854994
O	2.5381284	0.9682435	-0.7785286
C	3.9569217	0.9687114	-0.5985122
H	4.3283244	-0.0550457	-0.5436970
F	-2.5076249	1.2485710	0.0315574

Ac-(4*R*)Flp-OMe(2R-OMe)Ψ =-31.8° exo cis

H	-0.1411823	2.5097686	0.2576783
C	-0.1553382	1.5485281	0.7677984
C	-1.5551508	1.0729076	1.0828184
C	-1.3984422	-0.4182901	1.2519560

N	-0.3442358	-0.7469415	0.3058596
C	0.4301152	0.4135442	-0.0925978
H	0.3990921	1.6407334	1.7047699
H	-2.0057191	1.5664347	1.9444419
H	-1.1072556	-0.6605103	2.2793884
H	-2.3121826	-0.9623465	1.0156349
C	-0.2229769	-1.9966272	-0.1971668
H	3.6656388	-1.1364776	2.4233093
H	0.3002946	0.6346142	-1.1547502
C	1.9226831	0.3199863	0.1503266
H	4.1697493	-0.9338387	0.7183528
O	-0.9417110	-2.9062455	0.1970236
C	0.8356970	-2.2138196	-1.2427677
H	0.8609525	-1.4153298	-1.9864155
H	0.6417296	-3.1643767	-1.7342955
H	1.8195059	-2.2650619	-0.7687353
O	2.7330547	0.8968786	-0.5290744
O	2.2209402	-0.4108259	1.2159351
C	3.6109794	-0.4966749	1.5465077
H	4.0090817	0.4939542	1.7678608
F	-2.3770074	1.3230585	-0.0180990

Ac-(4S)Azp-OMe (3S-OMe)and Ac-(4S)Azp-NMe_2 (3S-NMe_2)

Ac-(4S)Azp-OMe(3S-OMe) endo trans Ψ =-177.4° C^{δ}-C^{γ}-N-N torsion = -172.6°

H	1.9256267	1.2394665	0.4794059
C	1.6171420	0.5383563	-0.2954669
C	1.3144760	-0.8608257	0.2399512
N	0.4236371	-1.4028169	-0.7555553
C	-0.1505460	-0.3773997	-1.6168612
C	0.3223417	0.9305824	-1.0000375
H	2.4189066	0.4770567	-1.0337059
C	0.6743415	-0.8545222	1.6226577
H	2.2179653	-1.4696433	0.3209610
H	0.1064221	0.3528277	3.9019957
H	-1.2399928	-0.4273179	-1.6438652
H	0.2295442	-0.4680408	-2.6387033
H	0.4742773	1.7011618	-1.7595855
C	0.2476978	-2.7374152	-0.8807081
O	0.8356794	-3.5263955	-0.1524567
C	-0.6997542	-3.1815541	-1.9592585
H	-0.7136176	-4.2684127	-1.9870103
H	-0.3965298	-2.7952033	-2.9354267
H	-1.7078960	-2.8114135	-1.7566184
O	-0.4087180	-1.2943396	1.8952804
O	1.5017460	-0.2969342	2.5054826
C	1.0246252	-0.2337951	3.8525028
H	1.8143213	0.2472177	4.4240373
H	0.8325208	-1.2371311	4.2336842
N	-0.7164507	1.3835542	-0.0516072
N	-0.4275366	2.3491456	0.6327206
N	-0.2573077	3.2293168	1.3146195

Ac-(4S)Azp-OMe(3S-OMe) endo trans Ψ =11.0° C^{δ}-C^{γ}-N-N torsion = 176.5°

H	1.8708461	1.1783131	0.5019656
C	1.5782557	0.4809549	-0.2823864
C	1.2318038	-0.9034249	0.2578332
N	0.3869359	-1.4519673	-0.7783978
C	-0.1456244	-0.4231043	-1.6632498
C	0.3052202	0.8802611	-1.0216643
H	2.3992200	0.4015345	-0.9974023
C	0.5813909	-0.8126657	1.6321598
H	2.1176707	-1.5279201	0.3944272
H	-0.5664877	-1.8578771	3.7586810
H	-1.2330453	-0.4696141	-1.7395633
H	0.2800708	-0.5119688	-2.6669553
H	0.4720847	1.6610749	-1.7672816
C	0.2509243	-2.7865453	-0.9439520
O	0.8070080	-3.5814950	-0.1971678
C	-0.6178568	-3.2252085	-2.0894147
H	-0.6220146	-4.3117955	-2.1287507
H	-0.2486652	-2.8281578	-3.0382668
H	-1.6402958	-2.8627831	-1.9567109
O	1.1011342	-0.2181894	2.5449261
O	-0.5689372	-1.4566052	1.7201638
C	-1.2084842	-1.4134723	2.9975415
H	-2.1245363	-1.9881687	2.8880023
H	-1.4342599	-0.3823793	3.2725288
N	-0.7586777	1.3098641	-0.0888045
N	-0.5175037	2.3145115	0.5595881
N	-0.3928158	3.2286803	1.2040040

Ac-(4S)Azp-OMe(3S-OMe) endo trans Ψ =178.0° C^{δ}-C^{γ}-N-N torsion = -51.9°

H	1.9112327	1.2758249	0.4380422
C	1.6336432	0.5585447	-0.3317820
C	1.3147441	-0.8316803	0.2214561
N	0.4379628	-1.3858618	-0.7804613
C	-0.1098359	-0.3741605	-1.6700436
C	0.3573632	0.9462635	-1.0611176
H	2.4507987	0.4788991	-1.0502969
C	0.6444716	-0.8002633	1.5893728
H	2.2136685	-1.4425299	0.3281405
H	0.1183256	0.4191443	3.8702429
H	-1.1984833	-0.4386898	-1.7415701
H	0.2961748	-0.4754618	-2.6815620
H	0.5263694	1.7036964	-1.8303100
C	0.2263035	-2.7187497	-0.8619828
O	0.7795889	-3.4959841	-0.0952455
C	-0.7158966	-3.1758631	-1.9397618
H	-0.7402430	-4.2628788	-1.9452263
H	-0.4038656	-2.8117299	-2.9215046
H	-1.7225993	-2.7942078	-1.7503125
O	-0.4754539	-1.1615669	1.8278953
O	1.4911225	-0.3260281	2.5004969
C	0.9847382	-0.2423830	3.8351635
H	1.7964206	0.1610069	4.4354330
H	0.6987491	-1.2314629	4.1943744
N	-0.5705227	1.4904426	-0.0491571
N	-1.7370134	1.5955056	-0.3798488
N	-2.8363046	1.7263941	-0.5873105

Ac-(4S)Azp-OMe(3S-OMe) endo trans Ψ =-0.1° C^{δ}-C^{γ}-N-N torsion = -52.3°

H	1.9360647	1.1825675	0.5571918
C	1.6706148	0.5059225	-0.2528968
C	1.2826864	-0.8891318	0.2394426
N	0.4347968	-1.3825713	-0.8217692
C	-0.0206370	-0.3258375	-1.7116145
C	0.4393040	0.9557077	-1.0220056
H	2.5152544	0.4252784	-0.9386635
C	0.6243952	-0.8374384	1.6110424
H	2.1551843	-1.5337905	0.3628057
H	-0.7627325	-1.8509445	3.6045239
H	-1.1026576	-0.3558214	-1.8625062
H	0.4542484	-0.4030451	-2.6949300
H	0.6560387	1.7463676	-1.7440808
C	0.1945232	-2.7046848	-0.9682655
O	0.6691157	-3.5242477	-0.1927172
C	-0.6835433	-3.0959906	-2.1234692
H	-0.7373973	-4.1809741	-2.1698646
H	-0.2945161	-2.7081943	-3.0679061
H	-1.6903152	-2.6911667	-1.9904154
O	1.2218769	-0.4539226	2.5860089
O	-0.6307745	-1.2536469	1.6192707
C	-1.2873017	-1.2182677	2.8879092
H	-2.2916347	-1.5946821	2.7110075
H	-1.3225613	-0.1958066	3.2661940
N	-0.5294348	1.4636568	-0.0282068
N	-1.6761636	1.6081141	-0.4092662
N	-2.7620722	1.7692482	-0.6616565

Ac-(4S)Azp-OMe(3S-OMe) endo cis Ψ =15.1° C^{δ}-C^{γ}-N-N torsion = 176.7°

H	2.0823540	1.1445957	0.5614323
C	1.7409382	0.4665968	-0.2199879
C	1.3065615	-0.8919205	0.3264691
N	0.4565962	-1.3966513	-0.7274126
C	-0.0428159	-0.3328295	-1.5907475
C	0.4902622	0.9400350	-0.9536007

H	2.5502929	0.3280485	-0.9391070
C	0.6321608	-0.7353172	1.6871501
H	2.1691969	-1.5408338	0.4944640
H	-0.5802398	-1.6694296	3.8344098
H	-1.1320644	-0.3394427	-1.6418508
H	0.3466608	-0.4585382	-2.6041023
H	0.7007072	1.7111073	-1.6978964
C	0.1930586	-2.6901446	-1.0125822
O	-0.5107315	-2.9848355	-1.9711966
C	0.8129970	-3.7309874	-0.1214609
H	0.4462581	-4.7079321	-0.4272726
H	0.5622650	-3.5585166	0.9262950
H	1.9022182	-3.7156733	-0.2150225
O	1.1409036	-0.1156098	2.5877498
O	-0.5230437	-1.3732269	1.7773238
C	-1.1990199	-1.2639811	3.0334407
H	-2.1139920	-1.8405078	2.9260271
H	-1.4276801	-0.2192161	3.2462009
N	-0.5369720	1.4336126	-0.0096114
N	-0.2306586	2.4238846	0.6333289
N	-0.0453172	3.3299769	1.2746024

Ac-(4S)Azp-OMe(3S-OMe) endo cis Ψ =5.3° C^{δ}-C^{γ}-N-N torsion = -56.4°

H	2.0268720	1.1727749	0.5739342
C	1.7251211	0.4913816	-0.2190991
C	1.3265810	-0.8902375	0.3057066
N	0.4757488	-1.3939675	-0.7480920
C	-0.0217814	-0.3362800	-1.6157308
C	0.4769837	0.9454990	-0.9571773
H	2.5459256	0.3821869	-0.9292035
C	0.6744326	-0.7828375	1.6805158
H	2.2086263	-1.5186317	0.4472654
H	-0.6527473	-1.7288489	3.7526944
H	-1.1092313	-0.3809509	-1.7082254
H	0.3948205	-0.4410776	-2.6215808
H	0.6827762	1.7269522	-1.6921592
C	0.1861859	-2.6871193	-1.0102660
O	-0.5267754	-2.9851483	-1.9608526
C	0.7884219	-3.7219037	-0.1006866
H	0.4178164	-4.6998731	-0.3982965
H	0.5249643	-3.5353720	0.9418743
H	1.8787594	-3.7150311	-0.1786139
O	1.2570444	-0.3143909	2.6257049
O	-0.5527559	-1.2763431	1.7258892
C	-1.2134598	-1.1858232	2.9913684
H	-2.1928652	-1.6335296	2.8445536
H	-1.3098306	-0.1412489	3.2893270
N	-0.4555747	1.4718083	0.0623099
N	-1.5936644	1.6950931	-0.3095166
N	-2.6674890	1.9320719	-0.5513974

Ac-(4S)Azp-OMe(3S-OMe) endo cis Ψ =-164.8° C^{δ}-C^{γ}-N-N torsion = 174.7°

H	2.1251199	1.1707251	0.5005348
C	1.7443979	0.4791671	-0.2492924
C	1.3778411	-0.8914384	0.3184259
N	0.4756840	-1.4113098	-0.6810256
C	-0.1047638	-0.3572495	-1.5046223
C	0.4409242	0.9266276	-0.9011963
H	2.5061485	0.3554624	-1.0214857
C	0.7276171	-0.8319758	1.6994846
H	2.2668312	-1.5184303	0.4267029
H	-0.0674816	0.4442640	3.8672388
H	-1.1944805	-0.3892886	-1.4818390
H	0.2177845	-0.4710121	-2.5426385
H	0.5887202	1.7013565	-1.6567160
C	0.2056588	-2.7096247	-0.9405885
O	-0.5572604	-3.0142824	-1.8493082
C	0.8948029	-3.7394845	-0.0891604
H	0.5362244	-4.7232565	-0.3824951
H	0.6880285	-3.5749945	0.9693168
H	1.9775010	-3.6977506	-0.2359439
O	-0.2615291	-1.4360202	2.0174820
O	1.4337485	-0.0643884	2.5238552
C	0.9412657	0.0303604	3.8648162
H	1.6269718	0.6943732	4.3845523
H	0.9294063	-0.9537168	4.3340914
N	-0.5335722	1.4035593	0.1052562
N	-0.1918715	2.3766322	0.7546812
N	0.0303674	3.2654236	1.4094802

Ac-(4S)Azp-OMe(3S-OMe) endo cis Ψ =-173.1° C^{δ}-C^{γ}-N-N torsion = -56.0°

H	2.1349340	1.1539901	0.5362252
C	1.7858043	0.4751427	-0.2386301
C	1.3970279	-0.9053354	0.2964356
N	0.4944713	-1.3934684	-0.7183585
C	-0.0385701	-0.3241042	-1.5498083
C	0.5083712	0.9481035	-0.9112384
H	2.5684024	0.3585567	-0.9897559
C	0.7422676	-0.8585509	1.6747493
H	2.2789260	-1.5435160	0.3952586
H	0.1258306	0.3926771	3.9145639
H	-1.1298016	-0.3576131	-1.5824212
H	0.3209174	-0.4249979	-2.5779050
H	0.6872709	1.7302025	-1.6527076
C	0.1647682	-2.6806183	-0.9655209
O	-0.6023028	-2.9600622	-1.8788017
C	0.7929909	-3.7304033	-0.0920045
H	0.4119560	-4.7027977	-0.3946987
H	0.5552309	-3.5567916	0.9587805
H	1.8807706	-3.7238818	-0.2002139
O	-0.3280760	-1.3325639	1.9474178
O	1.5466459	-0.2678108	2.5516472
C	1.0520008	-0.1824076	3.8916016
H	1.8281218	0.3217415	4.4616844
H	0.8688109	-1.1797690	4.2925615
N	-0.3611948	1.4824731	0.1578732
N	-1.5160899	1.7167665	-0.1507285
N	-2.5984643	1.9656085	-0.3344406

Ac-(4S)Azp-NMe_2(3S-NMe_2) endo trans Ψ =171.3°, C^{δ}-C^{γ}-N-N torsion = 149.1°

H	1.8778106	1.1838455	0.4437236
C	1.5756015	0.5114333	-0.3596659
C	1.3669717	-0.9371342	0.0921219
N	0.4362322	-1.4420698	-0.8878304
C	-0.2070066	-0.3957291	-1.6676197
C	0.2473035	0.8962168	-1.0019710
H	2.3530908	0.5472358	-1.1252072
C	0.7807032	-1.0464344	1.5108415
H	2.2877510	-1.5196937	0.0425624
H	-1.2943448	-0.4814007	-1.6513105
H	0.1307256	-0.4213221	-2.7084568
H	0.3644161	1.6987177	-1.7339035
C	0.2451232	-2.7700576	-1.0305738
O	0.8733776	-3.5777154	-0.3560358
C	-0.7687800	-3.1900316	-2.0574225
H	-0.7634140	-4.2748631	-2.1316789
H	-0.5500964	-2.7548243	-3.0354312
H	-1.7659268	-2.8535999	-1.7617948
O	-0.3936863	-1.3371530	1.6726976
N	1.6166343	-0.7768703	2.5381081
C	1.1043613	-0.7657847	3.8904125
H	1.6584530	-1.4783779	4.5079604
H	1.2148759	0.2318348	4.3266605
H	0.0529390	-1.0385611	3.8782895
C	3.0083930	-0.4110522	2.4184309
H	3.6043141	-1.0186594	3.1048663
H	3.3895846	-0.5731255	1.4148372
H	3.1573635	0.6421349	2.6812172
N	-0.7968249	1.3107194	-0.0418882
N	-0.4377851	1.9151881	0.9480352
N	-0.2053062	2.4677175	1.9032640

Ac-(4S)Azp-NMe_2(3S-NMe_2) endo trans Ψ =170.6°, C^{δ}-C^{γ}-N-N torsion = -20.2°

H	2.1856185	0.9239551	0.3891760
C	1.8104878	0.2995127	-0.4200061
C	1.4034074	-1.1094201	0.0208611
N	0.4363601	-1.4819129	-0.9836489
C	-0.0947013	-0.3421090	-1.7116436
C	0.5312063	0.8667641	-1.0241196

H	2.5782868	0.2301095	-1.1934233
C	0.7575013	-1.1476815	1.4178998
H	2.2366426	-1.8118241	-0.0097682
H	-1.1871454	-0.3203043	-1.6969358
H	0.2188183	-0.3634069	-2.7599423
H	0.7375845	1.6650501	-1.7396183
C	0.0668759	-2.7701186	-1.1434771
O	0.5829477	-3.6647994	-0.4844548
C	-0.9996775	-3.0341562	-2.1688533
H	-1.1217495	-4.1090391	-2.2788022
H	-0.7464946	-2.5925199	-3.1353732
H	-1.9476404	-2.5972457	-1.8432733
O	-0.4513685	-1.2820579	1.5306252
N	1.5824215	-1.0138589	2.4786155
C	1.0287572	-0.9728742	3.8134425
H	1.4300818	-1.7943957	4.4142881
H	1.2905526	-0.0281928	4.2999375
H	-0.0524348	-1.0622342	3.7562468
C	3.0161189	-0.8559874	2.4152502
H	3.4943066	-1.5844930	3.0762757
H	3.4009277	-1.0125243	1.4123461
H	3.3093164	0.1464019	2.7454766
N	-0.2630946	1.4615278	0.0714802
N	-1.4543217	1.2306592	0.1145298
N	-2.5619814	1.0751951	0.2475710

Ac-(4S)Azp-NMe$_2$(3S-NMe$_2$) endo trans
Ψ =-3.1°, C^{δ}-C^{γ}-N-N torsion = 170.7°

H	1.7139130	1.0779347	0.5061077
C	1.4952486	0.4311343	-0.3427345
C	1.1623724	-1.0013329	0.0808455
N	0.3577908	-1.4801203	-1.0268004
C	-0.0830313	-0.4087460	-1.9118148
C	0.2813802	0.8579479	-1.1569227
H	2.3696418	0.4277450	-0.9967607
C	0.6428265	-1.0465639	1.5266697
H	2.0676755	-1.6082931	0.1454557
H	-1.1549261	-0.4574994	-2.1146092
H	0.4515882	-0.4422816	-2.8655118
H	0.4955940	1.6851601	-1.8378364
C	0.2609441	-2.8010777	-1.2948293
O	0.7384066	-3.6379452	-0.5382765
C	-0.4828358	-3.1743597	-2.5459153
H	-0.4725050	-4.2566349	-2.6492941
H	-0.0260631	-2.7190403	-3.4279641
H	-1.5177993	-2.8253715	-2.4961974
O	1.4743573	-0.6932909	2.3607094
N	-0.5873127	-1.4622266	1.8770399
C	-0.8865477	-1.6089514	3.2888633
H	-1.6147624	-0.8565221	3.6063183
H	-1.3095586	-2.6005465	3.4683423
H	0.0233618	-1.4910307	3.8695904
C	-1.6833604	-1.8060018	0.9910934
H	-2.6089924	-1.7105858	1.5590965
H	-1.7488510	-1.1223031	0.1507102
H	-1.6066588	-2.8365718	0.6348092
N	-0.8740498	1.2157033	-0.3070000
N	-0.6876974	2.1097693	0.5006279
N	-0.6192579	2.9198489	1.2790241

Ac-(4S)Azp-NMe$_2$(3S-NMe$_2$) endo trans
Ψ =-4.7°, C^{δ}-C^{γ}-N-N torsion = -67.6°

H	1.7107313	1.0773296	0.5663732
C	1.5189387	0.4435453	-0.2966584
C	1.1742905	-0.9965484	0.0938445
N	0.3588321	-1.4452653	-1.0194794
C	-0.0525478	-0.3579819	-1.8952814
C	0.3267709	0.8991155	-1.1177658
H	2.4045615	0.4449869	-0.9342374
C	0.6665469	-1.0885033	1.5409969
H	2.0759416	-1.6111303	0.1363075
H	-1.1210282	-0.3976378	-2.1257516
H	0.4933729	-0.3846677	-2.8437428
H	0.5660302	1.7289753	-1.7867169
C	0.2200294	-2.7621847	-1.2929310
O	0.6766964	-3.6154817	-0.5428744
C	-0.5422082	-3.1071034	-2.5412301
H	-0.5581413	-4.1883869	-2.6538018
H	-0.0828053	-2.6546879	-3.4233519
H	-1.5687578	-2.7355158	-2.4793906
O	1.5157454	-0.8015248	2.3821268
N	-0.5692599	-1.4902183	1.8870553
C	-0.8516639	-1.7079913	3.2925230
H	-1.4910775	-0.9117123	3.6863209
H	-1.3707306	-2.6622153	3.4101883
H	0.0774962	-1.7270438	3.8545350
C	-1.6969616	-1.7267948	1.0098855
H	-2.6084422	-1.5165861	1.5711185
H	-1.6843668	-1.0632567	0.1529891
H	-1.7339553	-2.7664368	0.6719889
N	-0.7337675	1.3180606	-0.1775742
N	-1.7662328	1.7186480	-0.6857364
N	-2.7589024	2.1007567	-1.0558673

Ac-(4S)Azp-NMe$_2$(3S-NMe$_2$) endo cis
Ψ =178.5°, C^{δ}-C^{γ}-N-N torsion = 161.4°

H	2.0490620	1.1739449	0.4803385
C	1.7025348	0.5060478	-0.3078089
C	1.4528743	-0.9332759	0.1583661
N	0.5207498	-1.4142594	-0.8332383
C	-0.1442103	-0.3438890	-1.5624591
C	0.3671028	0.9289367	-0.9081936
H	2.4577951	0.5048781	-1.0962305
C	0.8621792	-1.0211501	1.5803834
H	2.3750615	-1.5169252	0.1241427
H	-1.2287738	-0.4318883	-1.4908582
H	0.1309948	-0.3798582	-2.6198477
H	0.4810912	1.7374323	-1.6340659
C	0.2219562	-2.7038592	-1.0957268
O	-0.5968378	-2.9907820	-1.9617348
C	0.9491014	-3.7521105	-0.2997640
H	0.6055801	-4.7313165	-0.6247194
H	0.7500581	-3.6321964	0.7667747
H	2.0289161	-3.6825968	-0.4550511
O	-0.2771746	-1.4264844	1.7480709
N	1.6553153	-0.6266809	2.6001450
C	1.1224087	-0.5930635	3.9449889
H	1.5955912	-1.3601694	4.5657159
H	1.3183237	0.3852543	4.3920251
H	0.0508957	-0.7695932	3.9129323
C	3.0461807	-0.2503051	2.4925166
H	3.4580474	-0.4789244	1.5139553
H	3.1785828	0.8193048	2.6866328
H	3.6271829	-0.8034350	3.2355504
N	-0.6170389	1.3519976	0.1112963
N	-0.2226954	2.1304198	0.9573933
N	0.0493300	2.8385226	1.7919116

Ac-(4S)Azp-NMe$_2$(3S-NMe$_2$) endo cis
Ψ =174.3°, C^{δ}-C^{γ}-N-N torsion = -25.1°

H	2.2205864	0.9478133	0.4370678
C	1.8441727	0.3319065	-0.3774493
C	1.4587989	-1.0919192	0.0443674
N	0.5078445	-1.4618254	-0.9777043
C	-0.0515127	-0.3139158	-1.6724347
C	0.5501661	0.8869103	-0.9563803
H	2.6032133	0.2825077	-1.1607152
C	0.8208820	-1.1585111	1.4466050
H	2.3232278	-1.7574185	0.0125753
H	-1.1433209	-0.3410712	-1.6543138
H	0.2503132	-0.3167421	-2.7235475
H	0.7324637	1.7140323	-1.6453442
C	0.0733169	-2.7080003	-1.2611390
O	-0.7653676	-2.8926947	-2.1357237
C	0.6767930	-3.8383717	-0.4748810
H	0.2488664	-4.7728021	-0.8300549
H	0.4612538	-3.7216667	0.5890501
H	1.7619263	-3.8698330	-0.6009344
O	-0.3705590	-1.4026167	1.5644073
N	1.6348296	-0.9504980	2.5030750
C	1.0772353	-0.9154369	3.8373014
H	1.5262591	-1.6981115	4.4557777
H	1.2816105	0.0536256	4.3023091
H	0.0034125	-1.0701738	3.7819572

C	3.0551023	-0.6963803	2.4352645
H	3.5705329	-1.3377428	3.1552290
H	3.4626517	-0.9077602	1.4511617
H	3.2789936	0.3455719	2.6878384
N	-0.2436834	1.4173291	0.1727951
N	-1.4429622	1.2220383	0.1756392
N	-2.5567531	1.0919995	0.2795875

Ac-(4S)Azp-NMe_2(3S-NMe_2) endo cis

Ψ =-1.9°, C^{δ}-C^{γ}-N-N torsion = **174.5°**

H	1.6617580	1.1897727	0.5622272
C	1.5172784	0.5230296	-0.2872309
C	1.2483708	-0.9224109	0.1400736
N	0.5380366	-1.4604163	-1.0014228
C	0.0402025	-0.4263840	-1.9000177
C	0.3189829	0.8709860	-1.1609386
H	2.4193115	0.5577502	-0.9014299
C	0.6441998	-0.9918844	1.5534629
H	2.1982517	-1.4448650	0.2725024
H	-1.0214477	-0.5593728	-2.1159258
H	0.5804565	-0.4619420	-2.8494217
H	0.5259026	1.6921683	-1.8510256
C	0.4688041	-2.7615062	-1.3572099
O	-0.0868225	-3.0970615	-2.3963480
C	1.1036991	-3.7574246	-0.4261947
H	0.8252557	-4.7582449	-0.7474960
H	0.7961731	-3.6050983	0.6103428
H	2.1926121	-3.6632999	-0.4629535
O	1.3995926	-0.6018924	2.4401439
N	-0.5689081	-1.5078813	1.8263294
C	-0.9261274	-1.6994265	3.2198451
H	-1.5833182	-0.8963179	3.5676515
H	-1.4530067	-2.6501850	3.3224714
H	-0.0291243	-1.7099280	3.8326218
C	-1.6338348	-1.8096372	0.8868244
H	-2.5833430	-1.6485583	1.3990363
H	-1.6156522	-1.1476866	0.0294024
H	-1.6029103	-2.8508874	0.5530356
N	-0.8789858	1.2016555	-0.3591348
N	-0.7757593	2.1665542	0.3786426
N	-0.7823280	3.0382116	1.0910030

Ac-(4S)Azp-NMe_2(3S-NMe_2) endo cis

Ψ =-3.5°, C^{δ}-C^{γ}-N-N torsion = -66.5°

H	1.6687143	1.1923381	0.6272728
C	1.5404346	0.5465213	-0.2387179
C	1.2595460	-0.9095814	0.1476681
N	0.5378487	-1.4087475	-1.0057876
C	0.0657015	-0.3476453	-1.8837472
C	0.3552072	0.9313180	-1.1049849
H	2.4468426	0.5881008	-0.8447311
C	0.6681414	-1.0198126	1.5639268
H	2.2053635	-1.4447175	0.2570622
H	-0.9923665	-0.4736556	-2.1289054
H	0.6176709	-0.3619785	-2.8278990
H	0.5766914	1.7683913	-1.7708732
C	0.4258358	-2.7017834	-1.3824495
O	-0.1438210	-3.0009548	-2.4249982
C	1.0299532	-3.7322367	-0.4695916
H	0.7367327	-4.7192125	-0.8191601
H	0.7093016	-3.5985472	0.5655324
H	2.1207294	-3.6577048	-0.4877706
O	1.4468929	-0.6958515	2.4566311
N	-0.5565622	-1.5083347	1.8351228
C	-0.9004221	-1.7382301	3.2256456
H	-1.4765993	-0.8996546	3.6294128
H	-1.5076946	-2.6430643	3.2921930
H	0.0038020	-1.8594366	3.8157816
C	-1.6538465	-1.7245945	0.9136307
H	-2.5751456	-1.3943062	1.3977646
H	-1.5391363	-1.1508154	0.0042686
H	-1.7630150	-2.7840260	0.6616370
N	-0.7523988	1.3073115	-0.2005756
N	-1.7943264	1.6341344	-0.7415592
N	-2.7985116	1.9491892	-1.1419071

Ac-(4R)Azp-OMe (3R-OMe)and Ac-(4R)Azp-NMe_2 (3R-NMe_2)

Ac-(4R)Azp-NMe_2(3R-NMe_2) exo cis

Ψ =159.8°, C^{δ}-C^{γ}-N-N torsion = 65.6°

H	-0.3103196	2.6036384	-0.0576197
C	-0.2461592	1.7033017	0.5515776
C	-1.6013457	1.2258625	1.0381750
C	-1.3312301	-0.2322420	1.3738743
N	-0.3872149	-0.6301310	0.3479457
C	0.3227559	0.4993356	-0.2200639
H	0.3811712	1.9147012	1.4201920
H	-1.9578391	1.7919839	1.9022205
H	-0.8933178	-0.3126962	2.3748712
H	-2.2164413	-0.8681749	1.3429929
C	-0.2802022	-1.9191913	-0.0416040
H	4.4110532	2.0705987	-0.1840997
H	0.0869757	0.5763869	-1.2832771
C	1.8341494	0.4073476	0.0093531
H	4.3131703	0.3220360	0.1222313
O	-0.9295384	-2.8013280	0.5088594
C	0.6651806	-2.2189906	-1.1713316
H	0.5844511	-1.4965160	-1.9859740
H	0.4459346	-3.2161017	-1.5463930
H	1.6920933	-2.1964215	-0.7996698
O	2.2643113	-0.2886681	0.9185875
C	4.0657220	1.1111598	-0.5821066
H	4.5735740	0.9277818	-1.5324492
N	-2.5532569	1.3757275	-0.0823670
N	-3.6881113	0.9866184	0.1333704
N	-4.7602730	0.6588306	0.2350034
N	2.6345915	1.1355640	-0.7957404
C	2.1972782	2.0681792	-1.8097846
H	2.6618499	1.8205819	-2.7683398
H	1.1200709	2.0575725	-1.9404329
H	2.4965889	3.0853856	-1.5380245

Ac-(4R)Azp-NMe_2(3R-NMe_2) exo cis

Ψ =160.4°, C^{δ}-C^{γ}-N-N torsion = -178.2°

H	-0.2452074	2.6365949	0.0040869
C	-0.1947356	1.7234626	0.5981424
C	-1.5496343	1.2562933	1.1154945
C	-1.2963386	-0.2049736	1.4192389
N	-0.3843382	-0.6027592	0.3630415
C	0.3387764	0.5204136	-0.1989624
H	0.4539080	1.9186623	1.4556522
H	-1.8746388	1.8128941	1.9977803
H	-0.8335329	-0.3111383	2.4057748
H	-2.1994809	-0.8120165	1.3913206
C	-0.3005880	-1.8888421	-0.0419831
H	4.4407441	2.0369761	-0.1661113
H	0.0917775	0.6165332	-1.2581151
C	1.8518220	0.3994202	0.0070435
H	4.3313414	0.2808285	0.0874923
O	-0.9457336	-2.7699186	0.5146723
C	0.6138359	-2.1871200	-1.1975907
H	0.5262877	-1.4524064	-2.0004560
H	0.3699526	-3.1753308	-1.5809786
H	1.6491735	-2.1865825	-0.8493984
O	2.2835513	-0.3248306	0.8929188
C	4.0848007	1.0928178	-0.5906569
H	4.5838179	0.9346845	-1.5500245
N	-2.5939722	1.3260974	0.0718793
N	-2.8839319	2.4472475	-0.3051904
N	-3.2202127	3.4400545	-0.7183152
N	2.6521967	1.1344753	-0.7923899
C	2.2140107	2.1057430	-1.7691618
H	2.7035897	1.9118959	-2.7272721
H	1.1405670	2.0750111	-1.9261024
H	2.4838127	3.1164846	-1.4463102

Ac-(4R)Azp-NMe_2(3R-NMe_2) exo cis

Ψ =165.6°, C^{δ}-C^{γ}-N-N torsion =-74.2°

H	-0.2962282	2.6507052	-0.2339481
C	-0.1487062	1.7988494	0.4296849
C	-1.4471505	1.3364605	1.0885303
C	-1.1713665	-0.1305160	1.3789391

N	-0.4024532	-0.5348146	0.2201089
C	0.3938677	0.5520888	-0.3078423
H	0.5492529	2.1057111	1.2110501
H	-1.6702490	1.8962595	1.9937579
H	-0.5845382	-0.2313555	2.2991081
H	-2.0730643	-0.7325508	1.4699854
C	-0.3998846	-1.8128144	-0.2279045
H	4.5124315	1.9735198	0.1072172
H	0.2364566	0.6285077	-1.3855128
C	1.8854850	0.3643539	-0.0008095
H	4.3449882	0.2134243	0.2984242
O	-1.0466229	-2.6837022	0.3384331
C	0.4122383	-2.0935840	-1.4614995
H	0.1435237	-1.4203892	-2.2792189
H	0.2301853	-3.1211628	-1.7666927
H	1.4766690	-1.9655456	-1.2529053
O	2.2292678	-0.4433812	0.8503164
C	4.1709410	1.0504561	-0.3718096
H	4.7409065	0.9069390	-1.2932628
N	-2.6383683	1.5141224	0.2371256
N	-2.5367609	1.2361381	-0.9437756
N	-2.5585427	1.0299867	-2.0524672
N	2.7594486	1.1339826	-0.6808429
C	2.4099831	2.1726198	-1.6233670
H	3.0188882	2.0659424	-2.5246293
H	1.3661313	2.1247972	-1.9177239
H	2.6030138	3.1618654	-1.1955367

Ac-(4R)Azp-NMe$_2$(3R-NMe$_2$) exo cis
Ψ =-38.2°, C^δ-C^γ-N-N torsion = 66.4°

H	-0.4015716	2.7525156	0.3813887
C	-0.4771827	1.7867750	0.8774243
C	-1.9035213	1.2739002	0.9647059
C	-1.7092508	-0.2205727	1.1831520
N	-0.5021201	-0.5166692	0.4300461
C	0.2533879	0.6843722	0.1004828
H	-0.0723945	1.8875715	1.8864145
H	-2.4673440	1.7387671	1.7771254
H	-1.5837987	-0.4348459	2.2508487
H	-2.5384184	-0.8321902	0.8255933
C	-0.2802206	-1.7604110	-0.0558352
H	4.0084434	-0.9719703	1.8758116
H	0.1759822	0.8897668	-0.9702699
C	1.7599586	0.6970170	0.3614926
H	4.1919982	0.3362812	0.6808863
O	-1.0162499	-2.6924085	0.2450698
C	0.9099965	-1.9426152	-0.9553746
H	1.0001029	-1.1437039	-1.6935123
H	0.8141628	-2.9015705	-1.4592711
H	1.8290785	-1.9498348	-0.3634419
O	2.4579846	1.2790009	-0.4618851
C	3.7139066	0.0470835	1.6127081
H	4.0444697	0.7213752	2.4083917
N	-2.5579538	1.5659889	-0.3266343
N	-3.7123367	1.1880639	-0.4298611
N	-4.7782350	0.8812495	-0.6216718
N	2.2748087	0.1053440	1.4573218
C	1.5222090	-0.5026040	2.5339476
H	0.5351262	-0.0654053	2.6403189
H	1.4163648	-1.5833713	2.3973106
H	2.0594523	-0.3268777	3.4676846

Ac-(4R)Azp-NMe$_2$(3R-NMe$_2$) exo cis
Ψ =-37.9°, C^δ-C^γ-N-N torsion = -179.7°

H	-0.3082557	2.7357612	0.5068955
C	-0.4324407	1.7395769	0.9309211
C	-1.8796949	1.2603083	0.9643014
C	-1.7324940	-0.2439702	1.0817565
N	-0.5191555	-0.5260655	0.3304698
C	0.2794949	0.6684785	0.0960044
H	-0.0438701	1.7645992	1.9516812
H	-2.4427322	1.6841072	1.7993086
H	-1.6321970	-0.5369187	2.1325189
H	-2.5757612	-0.7886862	0.6601919
C	-0.3189585	-1.7437964	-0.2250505
H	3.9510323	-1.2096824	1.8276651
H	0.2331770	0.9458480	-0.9602292
C	1.7801492	0.6199647	0.3865086
H	4.1943142	0.1685924	0.7257667
O	-1.0824481	-2.6732670	0.0072202
C	0.8827485	-1.9030398	-1.1140118
H	1.0100675	-1.0618640	-1.7976921
H	0.7689996	-2.8245542	-1.6802508
H	1.7897239	-1.9761893	-0.5080352
O	2.5102267	1.2374250	-0.3815055
C	3.6893103	-0.1677451	1.6270700
H	4.0209405	0.4434068	2.4718174
N	-2.5826456	1.5423667	-0.3036898
N	-2.7736705	2.7216622	-0.5455329
N	-2.9997553	3.7799173	-0.8565825
N	2.2558169	-0.0593688	1.4483446
C	1.4644588	-0.7164462	2.4667595
H	0.4876145	-0.2593742	2.5835134
H	1.3320805	-1.7820974	2.2551921
H	1.9865775	-0.6192378	3.4203091

Ac-(4R)Azp-NMe$_2$(3R-NMe$_2$) exo cis
Ψ =-37.9°, C^δ-C^γ-N-N torsion = -29.4°

H	-0.2898331	2.7107260	0.3301934
C	-0.4059593	1.7462106	0.8205034
C	-1.8595961	1.2861243	0.8955957
C	-1.7246805	-0.2146122	1.1005116
N	-0.5244047	-0.5505494	0.3491425
C	0.2907971	0.6189213	0.0479053
H	-0.0056400	1.8287943	1.8339095
H	-2.3937434	1.7651483	1.7134499
H	-1.6128521	-0.4429405	2.1659241
H	-2.5751905	-0.7897186	0.7333921
C	-0.3465073	-1.8007903	-0.1448264
H	3.9357903	-1.2226922	1.8719452
H	0.2531853	0.8352977	-1.0237863
C	1.7921294	0.5627876	0.3364912
H	4.1990427	0.0846332	0.6910864
O	-1.1207401	-2.7031969	0.1449301
C	0.8390420	-2.0197295	-1.0413905
H	0.9568647	-1.2218564	-1.7769071
H	0.7124189	-2.9730214	-1.5490127
H	1.7562462	-2.0585395	-0.4478125
O	2.5283874	1.1246640	-0.4669352
C	3.6919716	-0.1896349	1.6120525
H	4.0378455	0.4636237	2.4186613
N	-2.6076114	1.6622165	-0.3192452
N	-2.5698012	0.9057903	-1.2732236
N	-2.6174289	0.2808951	-2.2099884
N	2.2597405	-0.0663141	1.4318997
C	1.4600570	-0.6501176	2.4878179
H	0.4961209	-0.1622531	2.5868807
H	1.3000335	-1.7216960	2.3334150
H	1.9910927	-0.5159974	3.4318039

Ac-(4R)Azp-NMe$_2$(3R-NMe$_2$) exo trans
Ψ =131.2°, C^δ-C^γ-N-N torsion = -73.5°

H	-0.5055439	2.8159007	0.3689086
C	-0.3555571	1.8497833	0.8503650
C	-1.6663561	1.1999402	1.2914913
C	-1.3039747	-0.2787158	1.3369922
N	-0.4026286	-0.4143179	0.2102378
C	0.3083730	0.8214023	-0.0807135
H	0.2676000	1.9957797	1.7337832
H	-1.9991144	1.5614303	2.2617391
H	-0.7951398	-0.5062943	2.2803105
H	-2.1784575	-0.9202896	1.2398986
C	-0.1257926	-1.5306333	-0.4943708
H	4.6119712	1.8790170	-0.6281629
H	0.1601284	1.0695293	-1.1300463
C	1.7914375	0.6705729	0.2638950
H	4.2249455	0.7919027	0.7267583
O	0.6969908	-1.5035060	-1.4038637
C	-0.8721952	-2.7784890	-0.1163797
H	-0.7277938	-3.0160631	0.9399292
H	-0.5069833	-3.5999860	-0.7280231
H	-1.9447376	-2.6538618	-0.2858168
O	2.0897339	0.2525582	1.3781231
C	4.1049002	0.9553219	-0.3409569

H	4.5627941	0.1210110	-0.8815889
N	-2.8042524	1.4604376	0.3911005
N	-2.6102524	1.4000993	-0.8100269
N	-2.5554563	1.3879283	-1.9361537
N	2.6948474	1.0539113	-0.6529147
C	2.3493850	1.3906003	-2.0236597
H	1.7943681	0.5779318	-2.4963911
H	1.7722540	2.3161703	-2.0847117
H	3.2740349	1.5411502	-2.5780910

Ac-(4R)Azp-NMe$_2$(3R-NMe$_2$) exo trans

Ψ =131.3°, C$^{\delta}$-C$^{\gamma}$-N-N torsion = -176.4°

H	-0.4327984	2.7830304	0.4993439
C	-0.3601887	1.7817186	0.9251737
C	-1.7119394	1.1718559	1.2786702
C	-1.3949950	-0.3092672	1.3431712
N	-0.4109144	-0.4725908	0.2898092
C	0.2667068	0.7716747	-0.0411807
H	0.2415150	1.8360413	1.8347390
H	-2.1098650	1.5489227	2.2240156
H	-0.9745964	-0.5556140	2.3238343
H	-2.2790117	-0.9217743	1.1709910
C	-0.1170901	-1.5994577	-0.3844151
H	4.5199886	1.8873094	-0.8585500
H	0.0494722	1.0261450	-1.0774392
C	1.7685582	0.6485796	0.2217018
H	4.2202546	0.8518772	0.5574610
O	0.7175132	-1.5891912	-1.2853324
C	-0.8558988	-2.8455493	0.0158340
H	-0.7774194	-3.0250699	1.0902057
H	-0.4354752	-3.6884613	-0.5275838
H	-1.9172650	-2.7557163	-0.2301618
O	2.1378995	0.2473481	1.3202352
C	4.0395807	0.9715921	-0.5075320
H	4.4769662	0.1199550	-1.0385801
N	-2.7147056	1.3640467	0.2093035
N	-3.0274031	2.5194473	-0.0160148
N	-3.3812243	3.5513295	-0.2969311
N	2.6137076	1.0467062	-0.7443613
C	2.1907450	1.3447903	-2.1018270
H	1.6374407	0.5066625	-2.5301439
H	1.5828811	2.2511483	-2.1518863
H	3.0817333	1.5113864	-2.7047900

Ac-(4R)Azp-NMe$_2$(3R-NMe$_2$) exo trans

Ψ =131.4°, C$^{\delta}$-C$^{\gamma}$-N-N torsion = 65.2°

H	-0.4822136	2.7584196	0.4951280
C	-0.3914862	1.7621502	0.9251590
C	-1.7366327	1.1484255	1.2692336
C	-1.4067115	-0.3367598	1.3418806
N	-0.4174992	-0.4949512	0.2946750
C	0.2451741	0.7570158	-0.0404913
H	0.2080508	1.8172384	1.8353844
H	-2.1442604	1.5264596	2.2100121
H	-0.9907053	-0.5723859	2.3278780
H	-2.2778021	-0.9728349	1.1781731
C	-0.1116966	-1.6215321	-0.3753363
H	4.4903194	1.9032421	-0.8709448
H	0.0203858	1.0075380	-1.0760270
C	1.7486232	0.6484349	0.2183890
H	4.2005218	0.8648078	0.5449213
O	0.7241418	-1.6065497	-1.2745488
C	-0.8404226	-2.8731453	0.0270032
H	-0.7680779	-3.0466397	1.1027535
H	-0.4076755	-3.7144791	-0.5090970
H	-1.9009203	-2.7969718	-0.2279786
O	2.1248725	0.2491992	1.3153109
C	4.0147491	0.9853447	-0.5190987
H	4.4529918	0.1359826	-1.0531776
N	-2.6707252	1.4562701	0.1652287
N	-3.7865678	0.9785751	0.2682463
N	-4.8404341	0.5810504	0.2726157
N	2.5875921	1.0554039	-0.7495164
C	2.1577223	1.3507772	-2.1053604
H	1.6256969	0.5017105	-2.5394644
H	1.5266609	2.2411679	-2.1497382
H	3.0443800	1.5444904	-2.7066030

Ac-(4R)Azp-NMe$_2$(3R-NMe$_2$) exo trans

Ψ =-39.0°, C$^{\delta}$-C$^{\gamma}$-N-N torsion = -178.1°

H	-0.3326021	2.7033098	0.5912955
C	-0.4513160	1.7013904	1.0030641
C	-1.8977054	1.2183412	1.0296027
C	-1.7465626	-0.2884647	1.1345471
N	-0.5250052	-0.5586397	0.3934858
C	0.2634920	0.6423709	0.1604505
H	-0.0652444	1.7166287	2.0253131
H	-2.4654212	1.6303457	1.8675560
H	-1.6538880	-0.5818802	2.1867495
H	-2.6037453	-0.8073147	0.7075070
C	-0.1896555	-1.7281119	-0.1935082
H	4.0324079	0.3092358	2.5436543
H	0.2095057	0.9037982	-0.8990289
C	1.7670529	0.5934543	0.4274587
H	3.9738592	-1.2538383	1.7017391
O	0.8235700	-1.8199957	-0.8748175
C	-1.1106668	-2.8925781	0.0447710
H	-1.3455635	-3.0121346	1.1046443
H	-0.6325963	-3.7946893	-0.3297163
H	-2.0532823	-2.7431978	-0.4886316
O	2.4799044	1.2397470	-0.3321852
C	3.6973318	-0.1978327	1.6338808
H	4.1870359	0.2498469	0.7738541
N	-2.6038505	1.5027874	-0.2356113
N	-2.7724874	2.6835810	-0.4880336
N	-2.9792271	3.7427840	-0.8082307
N	2.2622317	-0.0796725	1.4856868
C	1.4915928	-0.7973044	2.4746390
H	0.5129778	-0.3538761	2.6309827
H	1.3673215	-1.8519964	2.2073360
H	2.0257410	-0.7481800	3.4254223

Ac-(4R)Azp-NMe$_2$(3R-NMe$_2$) exo trans

Ψ =-39.3°, C$^{\delta}$-C$^{\gamma}$-N-N torsion = 66.0°

H	-0.4185226	2.7265590	0.4889114
C	-0.4957664	1.7477697	0.9581690
C	-1.9235031	1.2335226	1.0166376
C	-1.7304076	-0.2678019	1.2038302
N	-0.5086334	-0.5436761	0.4686459
C	0.2421617	0.6665709	0.1642247
H	-0.1031660	1.8227319	1.9745711
H	-2.4996303	1.6760226	1.8329240
H	-1.6241015	-0.4965765	2.2714304
H	-2.5727463	-0.8483876	0.8250387
C	-0.1452775	-1.7336054	-0.0585102
H	4.0398166	0.5582483	2.5252653
H	0.1680642	0.8710546	-0.9064818
C	1.7489216	0.6712887	0.4165656
H	4.0150900	-1.0457614	1.7624421
O	0.8645202	-1.8339403	-0.7430603
C	-1.0320620	-2.9085932	0.2490819
H	-1.2573922	-2.9776478	1.3154497
H	-0.5310670	-3.8159106	-0.0800608
H	-1.9815287	-2.8158736	-0.2854026
O	2.4380468	1.2909749	-0.3856880
C	3.7101710	-0.0019097	1.6451606
H	4.1802018	0.4148245	0.7590240
N	-2.5651470	1.5519393	-0.2745584
N	-3.7150417	1.1688128	-0.4004263
N	-4.7758854	0.8561546	-0.6120578
N	2.2711900	0.0719811	1.5061398
C	1.5291829	-0.6107432	2.5405950
H	0.5401111	-0.1860220	2.6824577
H	1.4317424	-1.6815779	2.3328024
H	2.0698721	-0.4950852	3.4818802

Ac-(4R)Azp-NMe$_2$(3R-NMe$_2$) exo trans

Ψ =-39.0°, C$^{\delta}$-C$^{\gamma}$-N-N torsion = -26.1°

H	-0.3070885	2.6832533	0.4250687
C	-0.4212577	1.7090063	0.8960159
C	-1.8744571	1.2449517	0.9510727
C	-1.7363217	-0.2599855	1.1373851
N	-0.5255578	-0.5795679	0.3972250
C	0.2820240	0.5987299	0.1110670
H	-0.0301174	1.7740315	1.9146037

H	-2.4203930	1.7086642	1.7703002
H	-1.6339918	-0.4916537	2.2038194
H	-2.6001422	-0.8097340	0.7618696
C	-0.2097790	-1.7789073	-0.1450411
H	4.0393791	0.3229257	2.5163030
H	0.2420870	0.8060966	-0.9618762
C	1.7845467	0.5396516	0.3849325
H	3.9559822	-1.2813712	1.7580002
O	0.8004583	-1.9105566	-0.8217761
C	-1.1530331	-2.9159352	0.1317054
H	-1.3917709	-2.9954006	1.1943225
H	-0.6927490	-3.8391667	-0.2120436
H	-2.0915714	-2.7667220	-0.4097032
O	2.5087224	1.1373040	-0.4026980
C	3.6979114	-0.2259459	1.6336014
H	4.1971865	0.1672943	0.7527057
N	-2.6096694	1.6330233	-0.2679744
N	-2.6074528	0.8608815	-1.2094397
N	-2.6868804	0.2194070	-2.1328857
N	2.2652417	-0.0917840	1.4746472
C	1.4788644	-0.7451957	2.4952279
H	0.5121798	-0.2701074	2.6325468
H	1.3281977	-1.8075907	2.2766902
H	2.0152813	-0.6658659	3.4425617

Ac-(4R)Azp-OMe (3R-OMe) exo cis

Ψ =156.9°, C^{δ}-C^{γ}-N-N torsion = 66.1°

H	-0.1619409	2.6352688	0.3029086
C	-0.1728142	1.6644809	0.7943472
C	-1.5715044	1.1690798	1.1111277
C	-1.3576641	-0.3268447	1.2845442
N	-0.3304957	-0.6254557	0.3019731
C	0.4134709	0.5518338	-0.0939872
H	0.3903809	1.7340381	1.7274410
H	-1.9840988	1.6354114	2.0090279
H	-1.0141488	-0.5438062	2.3015101
H	-2.2477986	-0.9279410	1.0967801
C	-0.2172957	-1.8617816	-0.2371677
H	4.3700187	2.0333514	-1.1393678
H	0.2554079	0.7876367	-1.1495958
C	1.9021975	0.4379021	0.1662664
H	4.2142974	1.6343509	0.5985882
O	-0.9199876	-2.7851048	0.1537189
C	0.8087769	-2.0466915	-1.3201812
H	0.8136973	-1.2242243	-2.0380325
H	0.5959164	-2.9794320	-1.8374027
H	1.8037589	-2.1137510	-0.8732391
O	2.4053823	-0.2660476	1.0017674
O	2.5753355	1.2642950	-0.6258397
C	3.9915595	1.3127745	-0.4191820
H	4.4316501	0.3305196	-0.5930613
N	-2.4266756	1.4805893	-0.0521005
N	-3.5848898	1.1088559	0.0299183
N	-4.6697189	0.8094002	0.0105199

Ac-(4R)Azp-OMe (3R-OMe) exo cis

Ψ =-31.3°, C^{δ}-C^{γ}-N-N torsion = 66.1°

H	-0.3083498	2.7066254	0.4161874
C	-0.3065972	1.7266112	0.8889106
C	-1.6983426	1.1663084	1.1151422
C	-1.4294404	-0.3218318	1.2795356
N	-0.3326241	-0.5586890	0.3570496
C	0.3765481	0.6595389	0.0150443
H	0.1991331	1.8009463	1.8538821
H	-2.1831252	1.5991204	1.9935425
H	-1.1386598	-0.5414951	2.3123359
H	-2.2792732	-0.9575963	1.0293174
C	-0.1239900	-1.7816366	-0.1827739
H	3.6358749	-0.7667572	2.5733240
H	0.2608692	0.9041046	-1.0436725
C	1.8641614	0.6554289	0.3009047
H	4.1735527	-0.4665775	0.8931715
O	-0.7979879	-2.7447523	0.1597711
C	0.9739827	-1.8992634	-1.2031887
H	0.9673066	-1.0780581	-1.9220925
H	0.8548776	-2.8446030	-1.7275123
H	1.9459446	-1.9019380	-0.7027267
O	2.6528839	1.3092706	-0.3327037
O	2.1804593	-0.0932465	1.3487312
C	3.5631919	-0.0992090	1.7187979
H	3.8870277	0.9065466	1.9878722
N	-2.4964993	1.4596267	-0.0921974
N	-3.6401525	1.0372653	-0.0842299
N	-4.7077629	0.6918496	-0.1722738

Ac-(4R)Azp-OMe (3R-OMe) exo cis

Ψ =156.7°, C^{δ}-C^{γ}-N-N torsion = -179.9°

H	-0.1211804	2.6642671	0.3320310
C	-0.1521179	1.6846724	0.8080097
C	-1.5593030	1.1936055	1.1268840
C	-1.3597767	-0.3001358	1.2760120
N	-0.3374202	-0.5954983	0.2855641
C	0.4212283	0.5771579	-0.0938990
H	0.4131101	1.7405608	1.7416317
H	-1.9632739	1.6490545	2.0341108
H	-1.0140021	-0.5377836	2.2867997
H	-2.2619217	-0.8747734	1.0737640
C	-0.2284078	-1.8287276	-0.2610308
H	4.3952900	2.0247678	-1.1203567
H	0.2699404	0.8278723	-1.1470447
C	1.9084512	0.4442959	0.1676447
H	4.2335668	1.6109338	0.6135770
O	-0.9320491	-2.7529792	0.1256776
C	0.7948133	-2.0101151	-1.3476904
H	0.8074631	-1.1795751	-2.0560480
H	0.5723339	-2.9346130	-1.8756089
H	1.7894516	-2.0929772	-0.9025800
O	2.4024934	-0.2734811	0.9967138
O	2.5916414	1.2709944	-0.6156163
C	4.0081576	1.3016158	-0.4073961
H	4.4373170	0.3161894	-0.5903706
N	-2.4891127	1.4138777	0.0006505
N	-2.7478234	2.5790942	-0.2448299
N	-3.0503611	3.6207160	-0.5478255

Ac-(4R)Azp-OMe (3R-OMe) exo cis

Ψ =-31.1°, C^{δ}-C^{γ}-N-N torsion = -179.8°

H	-0.2343393	2.7118629	0.4677090
C	-0.2663917	1.7153259	0.9068794
C	-1.6752724	1.1743852	1.1199998
C	-1.4403429	-0.3174213	1.2336912
N	-0.3485567	-0.5497092	0.3021434
C	0.3939622	0.6592356	0.0035576
H	0.2388068	1.7534076	1.8750623
H	-2.1486230	1.5872163	2.0140050
H	-1.1524582	-0.5796995	2.2565425
H	-2.3099876	-0.9102131	0.9554609
C	-0.1597802	-1.7618768	-0.2680353
H	3.6005128	-0.9303356	2.5326167
H	0.2943981	0.9395562	-1.0479264
C	1.8791430	0.6100108	0.2991163
H	4.1572961	-0.5850988	0.8673874
O	-0.8494559	-2.7223311	0.0495807
C	0.9369029	-1.8720833	-1.2910615
H	0.9484690	-1.0303860	-1.9857893
H	0.7985393	-2.7989499	-1.8429576
H	1.9080929	-1.9104663	-0.7905212
O	2.6872724	1.2663744	-0.3069245
O	2.1700806	-0.1811744	1.3225492
C	3.5496147	-0.2322433	1.7012689
H	3.8945601	0.7558557	2.0070531
N	-2.5393184	1.4051771	-0.0550185
N	-2.8167756	2.5703684	-0.2805213
N	-3.1311668	3.6121845	-0.5699015

Ac-(4R)Azp-OMe (3R-OMe) exo cis

Ψ =166.0°, C^{δ}-C^{γ}-N-N torsion = -68.2°

H	-0.1457545	2.6917832	0.0773621
C	-0.0837149	1.7676180	0.6497124
C	-1.4448929	1.2709641	1.1346952
C	-1.2211133	-0.2258394	1.2897379
N	-0.3436133	-0.5302067	0.1748804
C	0.4857924	0.5968244	-0.1864063
H	0.5482595	1.9542524	1.5200775

H	-1.7361460	1.7366130	2.0730887
H	-0.7353170	-0.4396544	2.2481887
H	-2.1361044	-0.8114674	1.2253600
C	-0.3445081	-1.7460079	-0.4268675
H	4.5409034	2.0726258	-0.7359222
H	0.4191910	0.8149553	-1.2550012
C	1.9492466	0.3920780	0.1600686
H	4.2614249	1.4267842	0.9097663
O	-1.0573900	-2.6512245	-0.0181793
C	0.5548833	-1.9088891	-1.6201903
H	0.3669792	-1.1376053	-2.3709789
H	0.3728223	-2.8880170	-2.0562177
H	1.6039887	-1.8424671	-1.3227576
O	2.3727240	-0.4488193	0.9079710
O	2.6958740	1.3030025	-0.4526079
C	4.0968347	1.2677132	-0.1562622
H	4.5198763	0.3064819	-0.4490035
N	-2.5508693	1.5796840	0.2117671
N	-2.3975677	1.3262729	-0.9700854
N	-2.3739365	1.1520620	-2.0838097

Ac-(4R)Azp-OMe (3R-OMe) exo cis
Ψ =-30.5°, C^{δ}-C^{γ}-N-N torsion = -36.5°

H	-0.1270637	2.8035176	0.4414983
C	-0.1795248	1.8071010	0.8752622
C	-1.6095143	1.3213305	1.0978379
C	-1.4381638	-0.1870863	1.1664814
N	-0.3738366	-0.4380065	0.2077573
C	0.4409725	0.7356645	-0.0415690
H	0.3358102	1.8190222	1.8387209
H	-2.0313021	1.7282886	2.0140223
H	-1.1442754	-0.4891827	2.1766805
H	-2.3338959	-0.7449519	0.8941467
C	-0.2500720	-1.6403520	-0.4058945
H	3.4888987	-1.1258185	2.4957495
H	0.3876730	1.0446396	-1.0889715
C	1.9142145	0.5921041	0.2838935
H	4.1068642	-0.7512422	0.8586449
O	-0.9875947	-2.5718952	-0.1160413
C	0.8292165	-1.7657654	-1.4443576
H	0.8518375	-0.9136658	-2.1262682
H	0.6580903	-2.6796226	-2.0083242
H	1.8069882	-1.8352530	-0.9601682
O	2.7713552	1.2233486	-0.2793773
O	2.1342295	-0.2521384	1.2818362
C	3.4988003	-0.3956029	1.6909857
H	3.8900198	0.5594083	2.0426525
N	-2.5224474	1.7738367	0.0330792
N	-2.5211471	1.1696721	-1.0252158
N	-2.6189453	0.6908813	-2.0407931

Ac-(4R)Azp-OMe (3R-OMe) exo trans
Ψ =143.8°, C^{δ}-C^{γ}-N-N torsion = -178.0°

H	-0.2090792	2.6810219	0.4048950
C	-0.2287961	1.6895736	0.8567612
C	-1.6351184	1.1817817	1.1590880
C	-1.4259417	-0.3152663	1.2818861
N	-0.3853402	-0.5785782	0.3037267
C	0.3578224	0.6101893	-0.0607020
H	0.3320240	1.7287031	1.7937933
H	-2.0493612	1.6145197	2.0729627
H	-1.0945834	-0.5630877	2.2956858
H	-2.3376258	-0.8696760	1.0626123
C	-0.0938643	-1.7587566	-0.2812814
H	4.4070871	1.5332554	-1.4002380
H	0.2083619	0.8467873	-1.1163942
C	1.8424639	0.4370413	0.1942485
H	4.2695382	1.4477237	0.3824241
O	0.8014319	-1.8335794	-1.1153596
C	-0.9136916	-2.9450018	0.1392417
H	-0.9144553	-3.0601755	1.2253685
H	-0.4977236	-3.8370300	-0.3229669
H	-1.9512493	-2.8251103	-0.1833947
O	2.3027514	-0.1242941	1.1544298
O	2.5639911	1.0466162	-0.7374338
C	3.9822625	0.9857123	-0.5628110
H	4.3174354	-0.0518304	-0.5752322
N	-2.5658245	1.4092884	0.0351679
N	-2.8071773	2.5767777	-0.2179526
N	-3.0942762	3.6206166	-0.5275060

Ac-(4R)Azp-OMe (3R-OMe) exo trans
Ψ =43.5°, C^{δ}-C^{γ}-N-N torsion = 65.9°

H	-0.2478888	2.6550038	0.3928686
C	-0.2495300	1.6684381	0.8522046
C	-1.6484769	1.1572239	1.1488292
C	-1.4270608	-0.3441301	1.2857179
N	-0.3818978	-0.6055366	0.3141891
C	0.3465110	0.5899791	-0.0609622
H	0.3100100	1.7112142	1.7890781
H	-2.0712252	1.5947449	2.0564608
H	-1.0991698	-0.5777575	2.3047971
H	-2.3281852	-0.9232590	1.0781856
C	-0.0781778	-1.7881578	-0.2604257
H	4.3831893	1.5311322	-1.4271401
H	0.1881851	0.8189309	-1.1170259
C	1.8335515	0.4330714	0.1894960
H	4.2529893	1.4646615	0.3568879
O	0.8179249	-1.8614787	-1.0932379
C	-0.8869892	-2.9788084	0.1695728
H	-0.8925450	-3.0823703	1.2568115
H	-0.4588494	-3.8711410	-0.2807400
H	-1.9241184	-2.8754012	-0.1606051
O	2.3023372	-0.1129355	1.1544968
O	2.5464655	1.0376172	-0.7518783
C	3.9657716	0.9898090	-0.5819818
H	4.3090506	-0.0451691	-0.5839557
N	-2.5024519	1.4903932	-0.0092540
N	-3.6569825	1.1063653	0.0585597
N	-4.7384269	0.7943215	0.0271676

Ac-(4R)Azp-OMe (3R-OMe) exo trans
Ψ =145.1°, C^{δ}-C^{γ}-N-N torsion = -31.6°

H	-0.0697263	2.7747076	0.4095075
C	-0.1110536	1.7812184	0.8515428
C	-1.5387118	1.3182414	1.1361522
C	-1.3836794	-0.1932083	1.2209094
N	-0.3557811	-0.4664879	0.2313807
C	0.4546909	0.6954473	-0.0740455
H	0.4427675	1.7916694	1.7935394
H	-1.9224389	1.7421401	2.0616881
H	-1.0580250	-0.4755838	2.2275330
H	-2.3111911	-0.7224959	0.9998846
C	-0.1115383	-1.6389614	-0.3959753
H	4.5872161	1.5013858	-1.2183809
H	0.3582415	0.9617746	-1.1295314
C	1.9197537	0.4430695	0.2253614
H	4.3775057	1.3373157	0.5517737
O	0.7931892	-1.7231800	-1.2166925
C	-1.0009698	-2.7955541	-0.0418573
H	-1.0250452	-2.9607383	1.0374243
H	-0.6278124	-3.6868210	-0.5404290
H	-2.0253203	-2.6038058	-0.3730146
O	2.3166696	-0.1858402	1.1715722
O	2.7012961	1.0639456	-0.6478903
C	4.1083892	0.9334754	-0.4248706
H	4.4003760	-0.1158172	-0.4760925
N	-2.4827609	1.7666155	0.0961835
N	-2.5906280	1.0958571	-0.9149111
N	-2.7877536	0.5544565	-1.8836442

Ac-(4R)Azp-OMe (3R-OMe) exo trans
Ψ =-34.9°, C^{δ}-C^{γ}-N-N torsion = -178.3°

H	-0.2450592	2.6922417	0.4404624
C	-0.2798757	1.7034452	0.8966857
C	-1.6948039	1.1869576	1.1370106
C	-1.4819850	-0.3083246	1.2760854
N	-0.3935675	-0.5702982	0.3503505
C	0.3525276	0.6243531	0.0083273
H	0.2372869	1.7542474	1.8579984
H	-2.1535739	1.6206277	2.0289358
H	-1.1988533	-0.5502317	2.3059701
H	-2.3790113	-0.8690597	1.0162963
C	-0.0727158	-1.7528390	-0.2148449

H	3.5658222	-0.9619026	2.5256899
H	0.2374935	0.8579075	-1.0525220
C	1.8423423	0.5242932	0.2581677
H	4.0504365	-0.7924847	0.8120693
O	0.8623949	-1.8317754	-1.0030847
C	-0.9101740	-2.9385470	0.1720642
H	-0.9684219	-3.0462312	1.2574142
H	-0.4685039	-3.8326219	-0.2614975
H	-1.9296487	-2.8241523	-0.2055589
O	2.6606684	1.1105304	-0.4029451
O	2.1282264	-0.2062153	1.3287687
C	3.5170924	-0.3293885	1.6428228
H	3.9496902	0.6502188	1.8494504
N	-2.5735792	1.4024032	-0.0297362
N	-2.8159023	2.5671093	-0.2959165
N	-3.1005716	3.6069125	-0.6202565

Ac-(4R)Azp-OMe (3R-OMe) exo trans
Ψ =-35.2°, C^{δ}-C^{γ}-N-N torsion = 65.8°

H	-0.3161716	2.6885906	0.3954886
C	-0.3219314	1.7120794	0.8753386
C	-1.7230825	1.1788185	1.1169830
C	-1.4806805	-0.3151409	1.2946709
N	-0.3838048	-0.5762649	0.3811445
C	0.3336651	0.6280013	0.0093795
H	0.1918541	1.7879393	1.8360307
H	-2.1968041	1.6254838	1.9944982
H	-1.1998922	-0.5230057	2.3333854
H	-2.3600090	-0.9152177	1.0556834
C	-0.0357954	-1.7657620	-0.1535060
H	3.5849365	-0.8225214	2.5588801
H	0.2080374	0.8355463	-1.0556430
C	1.8258181	0.5654829	0.2564295
H	4.0611639	-0.6918236	0.8395485
O	0.9021823	-1.8442420	-0.9377995
C	-0.8486377	-2.9593754	0.2613396
H	-0.9118146	-3.0391158	1.3487817
H	-0.3840407	-3.8545482	-0.1451062
H	-1.8679157	-2.8800500	-0.1261268
O	2.6305145	1.1493215	-0.4231424
O	2.1293596	-0.1288939	1.3462917
C	3.5212160	-0.2160745	1.6588243
H	3.9351849	0.7771589	1.8365103
N	-2.5265055	1.4719845	-0.0868029
N	-3.6749557	1.0648134	-0.0668565
N	-4.7469979	0.7293353	-0.1439344

Ac-(4R)Azp-OMe (3R-OMe) exo trans
Ψ =-34.3°, C^{δ}-C^{γ}-N-N torsion = -26.3°

H	-0.0865723	2.7884298	0.4246045
C	-0.1580498	1.8000143	0.8735770
C	-1.6003908	1.3454244	1.0839832
C	-1.4552392	-0.1644750	1.2004546
N	-0.3742924	-0.4566639	0.2742061
C	0.4468606	0.7036662	-0.0145559
H	0.3450396	1.8188681	1.8435847
H	-2.0358764	1.7853414	1.9789637
H	-1.1879093	-0.4310465	2.2284353
H	-2.3703351	-0.6967387	0.9381488
C	-0.1044331	-1.6408879	-0.3201659
H	3.4729021	-1.1266953	2.5664103
H	0.3872131	0.9605138	-1.0752964
C	1.9192865	0.5169243	0.2849764
H	4.0327068	-0.9319377	0.8785358
O	0.8438679	-1.7458917	-1.0870464
C	-1.0200241	-2.7862193	0.0037903
H	-1.1116600	-2.9296097	1.0825634
H	-0.6225695	-3.6890792	-0.4534694
H	-2.0211034	-2.5968545	-0.3934708
O	2.7909382	1.0774605	-0.3279418
O	2.1246162	-0.2600335	1.3409204
C	3.4920813	-0.4663076	1.7030895
H	3.9661851	0.4824259	1.9570903
N	-2.4731935	1.7828946	-0.0218011
N	-2.5923970	1.0503875	-0.9872335
N	-2.7906801	0.4516291	-1.9213564

Ac-(4R)Azp-NMe$_2$ (3R-NMe$_2$) endo cis
Ψ =165.5°, C^{δ}-C^{γ}-N-N torsion = 88.9°

H	1.6725535	1.4319309	0.2362574
C	1.4291756	0.6496752	-0.4819469
C	1.3606873	-0.7480751	0.1381372
N	0.4987714	-1.4586420	-0.7814795
C	-0.2675481	-0.5763398	-1.6422540
C	0.0417215	0.8127813	-1.0838521
H	2.1739911	0.6642542	-1.2811357
C	0.7470025	-0.6880369	1.5473896
H	2.3428204	-1.2194256	0.1699957
H	1.0003806	0.8523551	4.2061475
H	-1.3291907	-0.8238824	-1.6055115
H	0.0675033	-0.6550833	-2.6815180
H	-0.6773187	1.0635215	-0.2984983
C	0.3087812	-2.7944972	-0.8187314
O	-0.4715698	-3.2958681	-1.6192448
C	1.1060830	-3.6227185	0.1515009
H	0.8634381	-4.6703669	-0.0092888
H	0.8643576	-3.3515428	1.1816270
H	2.1792515	-3.4767057	0.0059917
O	-0.4389023	-0.9455684	1.7026761
C	1.0130988	-0.1978957	3.8979153
H	1.6326735	-0.7580920	4.6029642
H	-0.0005720	-0.5881597	3.9139752
N	0.0535279	1.8594685	-2.1081109
N	-1.0150715	2.4008683	-2.3359306
N	-1.9430626	2.9713451	-2.6192929
C	2.9397784	0.0763152	2.4374043
H	3.0494494	1.1384851	2.6799811
H	3.5557660	-0.4973423	3.1352687
H	3.3250804	-0.0833068	1.4354649
N	1.5575943	-0.3266153	2.5633065

Ac-(4R)Azp-NMe$_2$ (3R-NMe$_2$) endo cis
Ψ =165.7°, C^{δ}-C^{γ}-N-N torsion = 159.8°

H	1.8213337	1.4328484	0.3710838
C	1.5511901	0.6889148	-0.3783083
C	1.4090754	-0.7247847	0.1925455
N	0.5272769	-1.3609102	-0.7621887
C	-0.1848570	-0.4147960	-1.6017699
C	0.1733787	0.9321800	-0.9918824
H	2.3057221	0.6948730	-1.1682747
C	0.7817034	-0.6849397	1.5963977
H	2.3677885	-1.2426656	0.2186680
H	1.0778934	0.7506555	4.3107572
H	-1.2564980	-0.6147763	-1.5911261
H	0.1633594	-0.4703646	-2.6381436
H	-0.5446070	1.1837424	-0.2056385
C	0.2760670	-2.6842360	-0.8488640
O	-0.5145647	-3.1208068	-1.6767812
C	1.0190814	-3.5819953	0.1024548
H	0.7309723	-4.6109159	-0.0990120
H	0.7737263	-3.3359751	1.1380136
H	2.1000427	-3.4813297	-0.0224269
O	-0.4162651	-0.8934962	1.7305686
C	1.0422017	-0.2873184	3.9650649
H	1.6245447	-0.9024652	4.6559336
H	0.0103348	-0.6267941	3.9564122
N	0.1654748	1.9677357	-2.0276227
N	0.0552445	3.1152999	-1.6320080
N	-0.0461545	4.2070094	-1.3758344
C	2.9969384	-0.0545945	2.5350572
H	3.1537384	0.9920124	2.8162871
H	3.5767406	-0.6808980	3.2185254
H	3.3863726	-0.1974231	1.5321720
N	1.5958490	-0.3953913	2.6323659

Ac-(4R)Azp-NMe$_2$ (3R-NMe$_2$) endo cis
Ψ =165.7°, C^{δ}-C^{γ}-N-N torsion = -60.5°

H	1.7108302	1.4912439	0.3377474
C	1.4665880	0.7372588	-0.4095256
C	1.3765431	-0.6811538	0.1587970
N	0.5141357	-1.3489193	-0.7922702
C	-0.2515142	-0.4313321	-1.6149408
C	0.0779955	0.9349577	-1.0137559
H	2.2282897	0.7643395	-1.1938366

C	0.7524398	-0.6656026	1.5648376
H	2.3526559	-1.1654309	0.1811002
H	1.0036158	0.7781071	4.2797342
H	-1.3159006	-0.6636877	-1.5744796
H	0.0675075	-0.4965659	-2.6613778
H	-0.6348064	1.1639264	-0.2228396
C	0.3149993	-2.6816042	-0.8810518
O	-0.4678463	-3.1453082	-1.7010864
C	1.1062493	-3.5521156	0.0562893
H	0.8530235	-4.5907727	-0.1420331
H	0.8700111	-3.3165468	1.0964049
H	2.1804044	-3.4113031	-0.0871793
O	-0.4362803	-0.9200103	1.7013714
C	1.0045874	-0.2600739	3.9329322
H	1.6107573	-0.8545832	4.6213577
H	-0.0143165	-0.6367216	3.9266394
N	-0.0347293	2.0578077	-1.9479229
N	0.6894745	2.0202240	-2.9289850
N	1.3115427	2.0873377	-3.8652758
C	2.9448732	0.0473963	2.4969414
H	3.3373910	-0.0840336	1.4936811
H	3.0624382	1.1001088	2.7744541
H	3.5494631	-0.5543142	3.1809731
N	1.5580383	-0.3463607	2.5985276

Ac-(4R)Azp-NMe$_2$ (3R-NMe$_2$) endo cis
Ψ =-14.1°, C^δ-C^γ-N-N torsion = 162.9°

H	1.5268802	1.2581279	0.7859491
C	1.5026619	0.6014191	-0.0824539
C	1.2710722	-0.8637646	0.2965046
N	0.6463575	-1.4134425	-0.8922593
C	0.2218913	-0.3964036	-1.8390827
C	0.3601512	0.8975326	-1.0496757
H	2.4502063	0.6938706	-0.6174843
C	0.5824762	-0.9967055	1.6647363
H	2.2323105	-1.3485801	0.4772068
H	-0.3549553	-1.7513009	3.8318957
H	-0.7950862	-0.5818240	-2.1892706
H	0.8781978	-0.3805033	-2.7147112
H	-0.5600883	1.1018120	-0.4912270
C	0.5585656	-2.7244303	-1.2103220
O	0.0650308	-3.0786969	-2.2737193
C	1.0900952	-3.7046776	-0.2017199
H	0.8735712	-4.7113965	-0.5505370
H	0.6402581	-3.5554799	0.7824114
H	2.1712653	-3.5893662	-0.0889356
O	1.3144163	-0.7822250	2.6264693
C	-1.1947460	-1.6146106	3.1565627
H	-1.8078189	-2.5184652	3.1438280
H	-1.8074867	-0.7817150	3.5144133
N	0.6260032	2.0046682	-1.9693035
N	0.4387378	3.1239263	-1.5222557
N	0.2932783	4.1965596	-1.2142312
C	-1.7185248	-1.4736222	0.7817148
H	-1.5344381	-0.8113142	-0.0558411
H	-1.8068089	-2.4993585	0.4114947
H	-2.6768919	-1.1866333	1.2169705
N	-0.7072180	-1.3554655	1.8145854

Ac-(4R)Azp-NMe$_2$ (3R-NMe$_2$) endo cis
Ψ =-14.2°, C^δ-C^γ-N-N torsion = -61.6°

H	1.3422963	1.3699426	0.6342111
C	1.3363699	0.6870325	-0.2132005
C	1.1987743	-0.7776398	0.2096496
N	0.5696885	-1.3969664	-0.9425698
C	0.0547532	-0.4366538	-1.9023275
C	0.1543066	0.8936748	-1.1547230
H	2.2727150	0.8117917	-0.7634955
C	0.5615145	-0.9105964	1.6027760
H	2.1903967	-1.2041216	0.3721229
H	-0.2623092	-1.6672659	3.8153498
H	-0.9647546	-0.6830274	-2.2040592
H	0.6722966	-0.4330871	-2.8073849
H	-0.7529460	1.0641288	-0.5744689
C	0.5505688	-2.7201762	-1.2223436
O	0.0465624	-3.1319273	-2.2594899
C	1.1705760	-3.6381699	-0.2060716
H	1.0027002	-4.6653913	-0.5201504
H	0.7442350	-3.4867178	0.7881095
H	2.2461297	-3.4571483	-0.1328706
O	1.3097132	-0.6297358	2.5342475
C	-1.1295414	-1.5905881	3.1656725
H	-1.6936756	-2.5254024	3.1928666
H	-1.7737389	-0.7828211	3.5257888
N	0.2428927	2.0688596	-2.0235720
N	1.2127552	2.1226938	-2.7628225
N	2.0647091	2.2745943	-3.4826820
C	-1.7351444	-1.5351780	0.8062656
H	-1.6171320	-0.8803209	-0.0488548
H	-1.7759702	-2.5715984	0.4582906
H	-2.6940615	-1.2944716	1.2674702
N	-0.6999594	-1.3379049	1.8027801

Ac-(4R)Azp-NMe$_2$ (3R-NMe$_2$) endo cis
Ψ =-14.5°, C^δ-C^γ-N-N torsion = 89.2°

H	1.3465999	1.3248882	0.6087357
C	1.3329459	0.6296752	-0.2286879
C	1.2012132	-0.8283353	0.2176419
N	0.5665331	-1.4648522	-0.9215756
C	0.0556603	-0.5154083	-1.8952988
C	0.1508200	0.8242478	-1.1636272
H	2.2558112	0.7547911	-0.7991246
C	0.5703769	-0.9421158	1.6152728
H	2.1946907	-1.2500422	0.3812525
H	-0.2478065	-1.6614554	3.8423606
H	-0.9612314	-0.7713940	-2.1999081
H	0.6809819	-0.5045736	-2.7934523
H	-0.7600396	1.0074160	-0.5837025
C	0.5425089	-2.7916994	-1.1803573
O	0.0336565	-3.2193726	-2.2090063
C	1.1628798	-3.6953303	-0.1512502
H	0.9946611	-4.7269573	-0.4503612
H	0.7365941	-3.5294999	0.8406801
H	2.2384085	-3.5133162	-0.0804797
O	1.3233777	-0.6522019	2.5401219
C	-1.1172987	-1.5929589	3.1948053
H	-1.6828916	-2.5263361	3.2379879
H	-1.7589744	-0.7789728	3.5453767
N	0.3903850	1.9515754	-2.0660699
N	-0.6018572	2.4942651	-2.5225555
N	-1.4455110	3.0723581	-2.9923576
C	-1.7322620	-1.5703281	0.8379279
H	-1.6125575	-0.9327876	-0.0299391
H	-1.7805855	-2.6128636	0.5095899
H	-2.6882096	-1.3150305	1.2976429
N	-0.6923692	-1.3610090	1.8268520

Ac-(4R)Azp-NMe$_2$ (3R-NMe$_2$) endo trans
Ψ =131.5°, C^δ-C^γ-N-N torsion = 157.8°

H	1.1254553	1.8704580	0.0129557
C	1.0740548	1.0217356	-0.6690665
C	1.2715466	-0.3221833	0.0344354
N	0.5136253	-1.2413753	-0.8002643
C	-0.3161583	-0.5761639	-1.7862600
C	-0.2935001	0.8782034	-1.3353457
H	1.8374600	1.1461818	-1.4407135
C	0.6812844	-0.2784424	1.4458312
H	2.3177728	-0.6174556	0.0402538
H	0.0103216	0.1572755	3.7945201
H	-1.3283672	-0.9813569	-1.8034402
H	0.1111312	-0.6585387	-2.7922186
H	-1.0820711	1.0470590	-0.5994723
C	0.6063120	-2.5682874	-0.5911892
O	1.3301886	-3.0143019	0.2950546
C	-0.2158533	-3.4522033	-1.4852665
H	0.0572020	-4.4885183	-1.3007249
H	-0.0604326	-3.2111915	-2.5390574
H	-1.2792796	-3.3188780	-1.2690586
O	-0.5226244	-0.0720587	1.5745948
C	1.0133369	-0.2593496	3.8328119
H	1.6640195	0.4075729	4.4031121
H	0.9763254	-1.2291070	4.3388075
N	-0.4766927	1.7529300	-2.4977314
N	-0.8927591	2.8735846	-2.2608346

N	-1.2757991	3.9270152	-2.1543490
C	2.9103978	-0.8147928	2.3544244
H	3.5428189	-0.0103858	1.9690885
H	3.2779205	-1.0839147	3.3435801
H	2.9948570	-1.6929308	1.7132661
N	1.5228692	-0.4041868	2.4857559

Ac-(4R)Azp-NMe$_2$ (3R-NMe$_2$) endo trans
Ψ =132.6°, C^{δ}-C^{γ}-N-N torsion =-62.3°

H	1.3548242	1.9384745	0.2022917
C	1.2605537	1.1318495	-0.5239010
C	1.3526965	-0.2583432	0.1072654
N	0.5479271	-1.0796880	-0.7837565
C	-0.2274502	-0.3121328	-1.7378272
C	-0.1022271	1.1198631	-1.2126390
H	2.0566032	1.2401128	-1.2661929
C	0.7426552	-0.2492434	1.5110650
H	2.3755586	-0.6262780	0.1113908
H	0.0547190	0.0983200	3.8692825
H	-1.2670142	-0.6388361	-1.7748435
H	0.1964657	-0.3934970	-2.7465804
H	-0.8814420	1.3015017	-0.4767895
C	0.5426401	-2.4187756	-0.6346211
O	1.2204703	-2.9536415	0.2381493
C	-0.3273450	-3.1999562	-1.5773527
H	-0.1297398	-4.2604126	-1.4397214
H	-0.1421179	-2.9216890	-2.6170759
H	-1.3814915	-3.0023474	-1.3648312
O	-0.4514832	0.0097337	1.6326067
C	1.0375255	-0.3645194	3.9002257
H	1.7075859	0.2426562	4.5131637
H	0.9492289	-1.3561116	4.3549639
N	-0.2931450	2.1517948	-2.2364486
N	0.5090352	2.1613814	-3.1549881
N	1.1923558	2.2609825	-4.0449947
C	2.9289796	-0.9364031	2.4196254
H	2.9761400	-1.8036988	1.7595460
H	3.6002584	-0.1549411	2.0538166
H	3.2791482	-1.2427073	3.4041092
N	1.5616567	-0.4636233	2.5545811

Ac-(4R)Azp-NMe$_2$ (3R-NMe$_2$) endo trans
Ψ =-16.8°, C^{δ}-C^{γ}-N-N torsion =-63.3°

H	1.2841115	1.3725833	0.5830950
C	1.2556919	0.6977253	-0.2702476
C	1.1454487	-0.7719753	0.1414533
N	0.4516438	-1.3832305	-0.9789652
C	-0.0667549	-0.4168048	-1.9292480
C	0.0377436	0.9041360	-1.1636478
H	2.1700451	0.8406938	-0.8524242
C	0.5954842	-0.9273209	1.5661940
H	2.1351845	-1.2220115	0.2336182
H	-1.4622623	-2.5764449	3.3019899
H	-1.0939421	-0.6397542	-2.2248891
H	0.5478732	-0.3831945	-2.8368378
H	-0.8512721	1.0443679	-0.5481773
C	0.4650086	-2.7254534	-1.1444709
O	0.9708574	-3.4561104	-0.3025190
C	-0.1869020	-3.2518844	-2.3913368
H	-0.0542256	-4.3304422	-2.4268760
H	0.2465588	-2.7990428	-3.2861068
H	-1.2556045	-3.0204823	-2.3916647
O	1.4023845	-0.6576979	2.4514193
C	-1.0164258	-1.5813675	3.2245513
H	-1.7425455	-0.8448992	3.5810775
H	-0.1255096	-1.5330081	3.8434951
N	0.0688394	2.0908101	-2.0197688
N	1.0231726	2.1887709	-2.7747484
N	1.8556405	2.3805776	-3.5077234
C	-1.7325988	-1.5771589	0.9002509
H	-1.6985407	-0.9130210	0.0433067
H	-1.7269495	-2.6153591	0.5548761
H	-2.6790417	-1.3944266	1.4107866
N	-0.6632816	-1.3192094	1.8423434

Ac-(4R)Azp-NMe$_2$ (3R-NMe$_2$) endo trans
Ψ =-17.1°, C^{δ}-C^{γ}-N-N torsion =-92.8°

H	1.2886851	1.3320568	0.5490382
C	1.2547019	0.6428363	-0.2927011
C	1.1470426	-0.8192930	0.1449290
N	0.4507273	-1.4483977	-0.9634034
C	-0.0606399	-0.4940584	-1.9307595
C	0.0399322	0.8380402	-1.1844492
H	2.1570227	0.7835269	-0.8918315
C	0.5991002	-0.9521331	1.5725444
H	2.1374258	-1.2667574	0.2425035
H	-1.4616807	-2.5652661	3.3371379
H	-1.0853007	-0.7254494	-2.2307927
H	0.5623705	-0.4572768	-2.8309968
H	-0.8540423	0.9956881	-0.5720587
C	0.4612528	-2.7924819	-1.1077593
O	0.9647819	-3.5118191	-0.2543065
C	-0.1916271	-3.3370908	-2.3465061
H	-0.0579204	-4.4159343	-2.3670872
H	0.2397608	-2.8959816	-3.2480562
H	-1.2606845	-3.1071486	-2.3482478
O	1.4079742	-0.6733303	2.4531621
C	-1.0145034	-1.5721928	3.2428444
H	-1.7395528	-0.8290079	3.5874822
H	-0.1232311	-1.5147706	3.8604946
N	0.2280438	1.9754467	-2.0857799
N	-0.7859146	2.5489163	-2.4467556
N	-1.6527869	3.1539113	-2.8329809
C	-1.7325635	-1.6032706	0.9197454
H	-1.6952209	-0.9552446	0.0506348
H	-1.7319012	-2.6475847	0.5931865
H	-2.6780161	-1.4067561	1.4271121
N	-0.6616140	-1.3332433	1.8564430

Ac-(4R)Azp-OMe (3R-OMe) endo cis
Ψ =163.8°, C^{δ}-C^{γ}-N-N torsion = 88.9°

H	1.6579514	1.3748821	0.4561623
C	1.4429824	0.6296553	-0.3084769
C	1.3177986	-0.7897948	0.2520445
N	0.4848233	-1.4529871	-0.7251944
C	-0.1834595	-0.5283283	-1.6247063
C	0.1028823	0.8363284	-0.9977460
H	2.2399125	0.6547419	-1.0546756
C	0.6760590	-0.7548835	1.6323592
H	2.2979291	-1.2603292	0.3448196
H	0.3567418	0.4656490	3.9525107
H	-1.2481262	-0.7540504	-1.6918164
H	0.2435245	-0.5839020	-2.6312140
H	-0.6669810	1.0729656	-0.2568257
C	0.2889838	-2.7867003	-0.8314710
O	-0.4360175	-3.2450723	-1.7045518
C	1.0052133	-3.6601181	0.1616932
H	0.7681175	-4.6980579	-0.0586874
H	0.6888179	-3.4310453	1.1821064
H	2.0870511	-3.5182994	0.1046812
O	-0.4922617	-0.9336226	1.8568747
O	1.5840610	-0.4657368	2.5569557
C	1.0948600	-0.3349382	3.8966010
H	1.9630197	-0.0974763	4.5057560
H	0.6406654	-1.2700318	4.2252988
N	0.2008415	1.9148945	-1.9822176
N	-0.8419984	2.4802647	-2.2658835
N	-1.7395475	3.0744186	-2.5942199

Ac-(4R)Azp-OMe (3R-OMe) endo cis
Ψ =164.3°, C^{δ}-C^{γ}-N-N torsion = 159.9°

H	1.8420802	1.3363631	0.6134006
C	1.5981389	0.6361192	-0.1849957
C	1.3739224	-0.7931266	0.3184067
N	0.5248818	-1.3654653	-0.7014497
C	-0.0677529	-0.3683240	-1.5764657
C	0.2807516	0.9459537	-0.8935082
H	2.4102026	0.6385406	-0.9149746
C	0.7062706	-0.7720894	1.6865939
H	2.3215715	-1.3261506	0.4119177
H	0.4050961	0.3806359	4.0442832
H	-1.1415717	-0.5265632	-1.6750041
H	0.3774379	-0.4092246	-2.5756972
H	-0.4900882	1.1969663	-0.1581251

C	0.2534594	-2.6797511	-0.8661993
O	-0.4764190	-3.0591945	-1.7724499
C	0.8931368	-3.6332578	0.1053556
H	0.5989193	-4.6453463	-0.1615012
H	0.5681624	-3.4252089	1.1275554
H	1.9825993	-3.5559348	0.0758422
O	-0.4739058	-0.8972335	1.8832647
O	1.6094776	-0.5635741	2.6373910
C	1.1012482	-0.4554476	3.9719658
H	1.9683755	-0.2864892	4.6049751
H	0.5922834	-1.3763954	4.2575583
N	0.3856517	2.0116806	-1.8913082
N	0.2802712	3.1501664	-1.4677467
N	0.1924964	4.2363360	-1.1856896

Ac-(4R)Azp-OMe (3R-OMe) endo cis
Ψ =164.5°, C^{δ}-C^{γ}-N-N torsion = -60.5°

H	1.7060745	1.4176639	0.5500131
C	1.4834436	0.7023212	-0.2402888
C	1.3294822	-0.7330845	0.2702360
N	0.4909455	-1.3485810	-0.7333538
C	-0.1743818	-0.3853906	-1.5927583
C	0.1422521	0.9532609	-0.9258413
H	2.2938227	0.7305282	-0.9733812
C	0.6813161	-0.7355823	1.6482586
H	2.3003403	-1.2245309	0.3517011
H	0.3576006	0.4137659	4.0040413
H	-1.2439485	-0.5864162	-1.6513680
H	0.2331807	-0.4325479	-2.6089669
H	-0.6173392	1.1746657	-0.1767817
C	0.2813758	-2.6755766	-0.8915015
O	-0.4486195	-3.0906980	-1.7814726
C	0.9897925	-3.5946587	0.0650862
H	0.7357809	-4.6202722	-0.1911786
H	0.6835103	-3.3979110	1.0952555
H	2.0732979	-3.4670549	0.0055233
O	-0.4885550	-0.9167596	1.8616223
O	1.5866358	-0.4784197	2.5846163
C	1.0928107	-0.3876194	3.9260091
H	1.9595997	-0.1727392	4.5453872
H	0.6338580	-1.3307928	4.2235151
N	0.1139656	2.1062495	-1.8275867
N	0.9013207	2.0839222	-2.7598488
N	1.5844916	2.1681156	-3.6507404

Ac-(4R)Azp-OMe (3R-OMe) endo cis
Ψ =-19.8°, C^{δ}-C^{γ}-N-N torsion = 160.1°

H	1.7667374	1.2061478	0.7589179
C	1.5606467	0.5523932	-0.0880163
C	1.3521955	-0.9084084	0.3222662
N	0.5471255	-1.4337417	-0.7594481
C	-0.0434417	-0.3967878	-1.5884891
C	0.2617676	0.8818859	-0.8212755
H	2.3951457	0.6124513	-0.7897908
C	0.7097203	-0.9795586	1.7006647
H	2.3084344	-1.4261276	0.4100692
H	-0.9178093	-1.9897270	3.5146296
H	-1.1112718	-0.5682849	-1.7248518
H	0.4269180	-0.3704895	-2.5764080
H	-0.5364720	1.0812559	-0.0994244
C	0.3096884	-2.7404727	-1.0106015
O	-0.3908568	-3.0800370	-1.9552595
C	0.9487119	-3.7368167	-0.0824698
H	0.6681845	-4.7367423	-0.4041935
H	0.6163376	-3.5879091	0.9478508
H	2.0374540	-3.6462287	-0.0969134
O	1.3507844	-0.8847861	2.7162466
O	-0.6078632	-1.1157274	1.6550734
C	-1.2780148	-1.1496844	2.9202758
H	-2.3334037	-1.2690327	2.6903951
H	-1.1049051	-0.2207348	3.4640270
N	0.3798712	2.0029554	-1.7549167
N	0.2475980	3.1145969	-1.2713971
N	0.1369741	4.1819203	-0.9316556

Ac-(4R)Azp-OMe (3R-OMe) endo cis
Ψ =-20.0°, C^{δ}-C^{γ}-N-N torsion = -60.7°

H	1.6193148	1.3033503	0.6746262
C	1.4333802	0.6281089	-0.1591992
C	1.3021212	-0.8355974	0.2703763
N	0.5044433	-1.4144884	-0.7896313
C	-0.1648788	-0.4216900	-1.6114889
C	0.1071493	0.8878776	-0.8700416
H	2.2626236	0.7124009	-0.8665260
C	0.6867632	-0.9198278	1.6606899
H	2.2828348	-1.3069254	0.3474763
H	-0.8639713	-1.9833725	3.5110886
H	-1.2283524	-0.6421964	-1.7045362
H	0.2641352	-0.4073540	-2.6196050
H	-0.6774578	1.0592307	-0.1338762
C	0.3314308	-2.7352493	-1.0228326
O	-0.3717861	-3.1201638	-1.9475306
C	1.0449803	-3.6861480	-0.1017198
H	0.8063990	-4.7029325	-0.4038059
H	0.7349738	-3.5406574	0.9360352
H	2.1269305	-3.5412431	-0.1487770
O	1.3395278	-0.7845802	2.6640084
O	-0.6235522	-1.1165922	1.6379708
C	-1.2716077	-1.1671161	2.9142426
H	-2.3238825	-1.3368926	2.7026389
H	-1.1325248	-0.2252770	3.4453845
N	0.0803263	2.0831165	-1.7151068
N	0.8948255	2.1218779	-2.6234003
N	1.6014673	2.2625235	-3.4883535

Ac-(4R)Azp-OMe (3R-OMe) endo cis
Ψ =-20.7°, C^{δ}-C^{γ}-N-N torsion = 89.1°

H	1.5712573	1.2782502	0.5854129
C	1.3970214	0.5717194	-0.2249014
C	1.2918509	-0.8781444	0.2557046
N	0.5042382	-1.5064893	-0.7831861
C	-0.1627451	-0.5505162	-1.6503942
C	0.0761240	0.7867279	-0.9478852
H	2.2163064	0.6539033	-0.9423231
C	0.6775020	-0.9252054	1.6479733
H	2.2809959	-1.3285309	0.3487673
H	-0.8660349	-1.9395874	3.5313633
H	-1.2200972	-0.7952875	-1.7575257
H	0.2900822	-0.5450091	-2.6468715
H	-0.7224070	0.9741317	-0.2235223
C	0.3458811	-2.8364648	-0.9660271
O	-0.3469239	-3.2652368	-1.8796193
C	1.0612716	-3.7434812	-0.0027607
H	0.8412297	-4.7738486	-0.2710343
H	0.7349931	-3.5657325	1.0249556
H	2.1415349	-3.5842914	-0.0405366
O	1.3289169	-0.7559758	2.6470852
O	-0.6320475	-1.1299122	1.6320125
C	-1.2791835	-1.1450643	2.9094798
H	-2.3304310	-1.3283976	2.7039851
H	-1.1463264	-0.1863272	3.4112237
N	0.1837964	1.9134033	-1.8760276
N	-0.8603409	2.4741625	-2.1636351
N	-1.7590606	3.0679547	-2.4896262

Ac-(4R)Azp-OMe (3R-OMe) endo trans
Ψ =152.0°, C^{δ}-C^{γ}-N-N torsion = 93.3°

H	1.5760002	1.4739995	0.4152445
C	1.4118053	0.7265985	-0.3603622
C	1.3174169	-0.6995759	0.1858028
N	0.4775894	-1.3716793	-0.7828673
C	-0.1353763	-0.4654635	-1.7356639
C	0.0881317	0.9031090	-1.0894754
H	2.2311150	0.7889676	-1.0798586
C	0.6739303	-0.7074404	1.5625644
H	2.2935963	-1.1791430	0.2612265
H	0.4993375	0.2941843	3.9984298
H	-1.1941538	-0.6842131	-1.8778636
H	0.3662095	-0.5018182	-2.7092672
H	-0.7111283	1.1024211	-0.3699264
C	0.2730524	-2.7029009	-0.6821201
O	0.8046785	-3.3474485	0.2142088
C	-0.6280281	-3.3268165	-1.7085514
H	-0.5743040	-4.4084374	-1.6097735

H	-0.3472260	-3.0328907	-2.7220078
H	-1.6600102	-3.0053902	-1.5432440
O	-0.5133461	-0.6919490	1.7627960
O	1.5959749	-0.6668469	2.5162095
C	1.1032975	-0.6029927	3.8581513
H	1.9836800	-0.5739226	4.4949832
H	0.5002133	-1.4838894	4.0796248
N	0.1774701	1.9873237	-2.0681842
N	-0.8623152	2.5733588	-2.3184933
N	-1.7586557	3.1848391	-2.6171147

Ac-(4R)Azp-OMe (3R-OMe) endo trans
Ψ =152.4°, C^{δ}-C^{γ}-N-N torsion = 159.7°

H	1.7753924	1.4096251	0.5747973
C	1.5764520	0.7035409	-0.2314512
C	1.3719268	-0.7307577	0.2623445
N	0.5101989	-1.3082079	-0.7470151
C	-0.0222397	-0.3293284	-1.6762851
C	0.2772898	0.9918476	-0.9816521
H	2.4128460	0.7319304	-0.9330613
C	0.7004701	-0.7420890	1.6255531
H	2.3112255	-1.2790128	0.3390501
H	0.5397908	0.1884074	4.0903477
H	-1.0898930	-0.4698721	-1.8450637
H	0.4940518	-0.3653825	-2.6422689
H	-0.5221321	1.2201306	-0.2704857
C	0.2166454	-2.6255400	-0.7023251
O	0.6852504	-3.3375296	0.1781072
C	-0.7009214	-3.1491780	-1.7694401
H	-0.7201245	-4.2349360	-1.7120111
H	-0.3798405	-2.8363617	-2.7651138
H	-1.7129605	-2.7665646	-1.6113959
O	-0.4872186	-0.6577316	1.8044799
O	1.6038491	-0.7912941	2.5966336
C	1.0892220	-0.7400705	3.9308757
H	1.9567259	-0.7873315	4.5840759
H	0.4279741	-1.5878885	4.1116576
N	0.3898786	2.0579542	-1.9779958
N	0.2446297	3.1945224	-1.5607057
N	0.1233189	4.2786601	-1.2839658

Ac-(4R)Azp-OMe (3R-OMe) endo trans
Ψ =152.5°, C^{δ}-C^{γ}-N-N torsion = -62.4°

H	1.6098137	1.5100112	0.4965679
C	1.4399335	0.7888455	-0.3020898
C	1.3265839	-0.6526939	0.1987692
N	0.4797008	-1.2854323	-0.7904638
C	-0.1352079	-0.3462472	-1.7083607
C	0.1123095	1.0003264	-1.0256629
H	2.2712725	0.8558393	-1.0089628
C	0.6826015	-0.6952282	1.5748686
H	2.2965947	-1.1466303	0.2597104
H	0.5121796	0.2391847	4.0374493
H	-1.1986850	-0.5438726	-1.8420364
H	0.3498382	-0.3764951	-2.6919176
H	-0.6770819	1.1822578	-0.2976377
C	0.2639782	-2.6180678	-0.7309854
O	0.7939246	-3.2943875	0.1421723
C	-0.6461874	-3.2011657	-1.7729523
H	-0.6009071	-4.2857960	-1.7094465
H	-0.3672241	-2.8770497	-2.7777015
H	-1.6747235	-2.8767341	-1.5927837
O	-0.5045116	-0.6818422	1.7750434
O	1.6051979	-0.6837793	2.5285940
C	1.1134634	-0.6555120	3.8722426
H	1.9943581	-0.6465143	4.5089422
H	0.5080769	-1.5405271	4.0696174
N	0.0638118	2.1531539	-1.9258232
N	0.8981756	2.1820601	-2.8161381
N	1.6198786	2.3117116	-3.6705270

Ac-(4R)Azp-OMe (3R-OMe) endo trans
Ψ =-27.9°, C^{δ}-C^{γ}-N-N torsion =160.6°

H	1.7166888	1.1613494	0.7606814
C	1.5367742	0.5358527	-0.1133733
C	1.3400114	-0.9411387	0.2386869
N	0.4900038	-1.4252607	-0.8302579
C	-0.0279400	-0.3668954	-1.6769557
C	0.2506325	0.8864111	-0.8589203
H	2.3849102	0.6373176	-0.7938163
C	0.7393512	-1.1035089	1.6247988
H	2.2861167	-1.4829123	0.2638111
H	-0.9352728	-2.1570721	3.3627404
H	-1.0909364	-0.4950491	-1.8816583
H	0.5085386	-0.3121038	-2.6309709
H	-0.5642361	1.0482567	-0.1465878
C	0.2040436	-2.7420426	-0.9174156
O	0.6568799	-3.5322826	-0.0978322
C	-0.6893987	-3.1657872	-2.0477293
H	-0.7046210	-4.2522030	-2.0924724
H	-0.3494020	-2.7605619	-3.0029870
H	-1.7065659	-2.8026366	-1.8765829
O	1.4118396	-1.1758849	2.6212918
O	-0.5877117	-1.0876275	1.6180919
C	-1.2176154	-1.2130492	2.8959278
H	-2.2869217	-1.1891323	2.7018144
H	-0.9271340	-0.3867765	3.5456367
N	0.3742128	2.0384247	-1.7528156
N	0.2310854	3.1327661	-1.2338523
N	0.1120349	4.1874140	-0.8596572

Ac-(4R)Azp-OMe (3R-OMe) endo trans
Ψ =-28.0°, C^{δ}-C^{γ}-N-N torsion =-62.6°

H	1.5607393	1.2748484	0.6707032
C	1.4036746	0.6293173	-0.1923048
C	1.2980836	-0.8526105	0.1763845
N	0.4553091	-1.3984769	-0.8690697
C	-0.1461572	-0.3843629	-1.7131691
C	0.0852632	0.8987117	-0.9128728
H	2.2426103	0.7649384	-0.8800780
C	0.7351129	-1.0320718	1.5764437
H	2.2733492	-1.3402651	0.1877926
H	-0.8441682	-2.1572434	3.3574921
H	-1.2063081	-0.5717356	-1.8836640
H	0.3563632	-0.3279327	-2.6867634
H	-0.7157095	1.0160504	-0.1839918
C	0.2418843	-2.7312345	-0.9314273
O	0.7567415	-3.4814740	-0.1114745
C	-0.6506593	-3.2210674	-2.0351401
H	-0.6041390	-4.3070764	-2.0659853
H	-0.3561883	-2.8104020	-3.0031282
H	-1.6829426	-2.9145745	-1.8447783
O	1.4302529	-1.0562777	2.5595008
O	-0.5905027	-1.0893468	1.5958498
C	-1.1878501	-1.2355624	2.8871704
H	-2.2603105	-1.2732987	2.7139571
H	-0.9314768	-0.3872463	3.5226245
N	0.0422571	2.1245105	-1.7107913
N	0.8906837	2.2351411	-2.5814980
N	1.6248118	2.4424813	-3.4093839

Ac-(4R)Azp-OMe (3R-OMe) endo trans
Ψ =-28.3°, C^{δ}-C^{γ}-N-N torsion =92.6°

H	1.5172302	1.2621476	0.6002615
C	1.3742256	0.5866018	-0.2421550
C	1.2859554	-0.8833386	0.1751381
N	0.4595851	-1.4734873	-0.8587458
C	-0.1336427	-0.4920735	-1.7473867
C	0.0661327	0.8180914	-0.9832866
H	2.2070572	0.7189396	-0.9362014
C	0.7126264	-1.0252188	1.5751067
H	2.2677649	-1.3565321	0.2108835
H	-0.8713880	-2.1097545	3.3774049
H	-1.1870217	-0.7018560	-1.9362646
H	0.3929501	-0.4439305	-2.7071910
H	-0.7513044	0.9548163	-0.2692215
C	0.2601747	-2.8090567	-0.8796847
O	0.7715769	-3.5274576	-0.0291507
C	-0.6137073	-3.3442029	-1.9775958
H	-0.5578351	-4.4302022	-1.9705523
H	-0.3095447	-2.9642170	-2.9550636
H	-1.6510318	-3.0405356	-1.8115905
O	1.3987288	-1.0119036	2.5647715
O	-0.6128665	-1.0945847	1.5855461

C	-1.2195097	-1.2064124	2.8757895
H	-2.2901477	-1.2597718	2.6953081
H	-0.9764575	-0.3365199	3.4868860
N	0.1698981	1.9812458	-1.8649927
N	-0.8703796	2.5732490	-2.0986250
N	-1.7665545	3.1962721	-2.3730099

Ac-(4S)Azp-NMe$_2$ (3S-NMe$_2$) exo cis
Ψ =162.3°, C$^{\delta}$-C$^{\gamma}$-N-N torsion = 61.2°

H	0.4172442	1.2514163	1.4085316
C	1.1289938	1.1798127	0.5802091
C	0.9939322	-0.1933814	-0.0987243
N	0.1350773	0.0644451	-1.2407093
C	-0.3546563	1.4238320	-1.2735058
C	0.7359710	2.1695212	-0.5134553
H	2.1274297	1.3573769	0.9766777
C	0.3632247	-1.1956665	0.8757890
H	1.9641960	-0.5492629	-0.4501897
H	-0.4255970	-2.8980166	2.5012600
H	-1.3214428	1.5107914	-0.7608213
H	-0.4790947	1.7610439	-2.3006303
H	1.5812736	2.3456006	-1.1785887
C	-0.2058796	-0.8310626	-2.1955977
O	-0.9690041	-0.5271611	-3.1041551
C	0.3945896	-2.2054422	-2.0905203
H	0.2395494	-2.7197051	-3.0361630
H	-0.1086178	-2.7620134	-1.2966016
H	1.4616019	-2.1784465	-1.8608436
O	-0.8421780	-1.3963014	0.8352346
C	0.6106756	-2.6917655	2.7534882
H	0.6560546	-2.2420601	3.7504418
H	1.1778609	-3.6258513	2.7685093
N	0.3569079	3.5001818	-0.0363029
N	-0.5728081	3.5510808	0.7528305
N	-1.4116218	3.7162885	1.4855312
C	2.5844610	-1.5192586	1.9479205
H	3.1419118	-2.4591881	1.9754245
H	2.9886530	-0.9111546	1.1448375
H	2.7525804	-0.9948665	2.8942544
N	1.1774743	-1.7961626	1.7676962

Ac-(4S)Azp-NMe$_2$ (3S-NMe$_2$) exo cis
Ψ =162.5°, C$^{\delta}$-C$^{\gamma}$-N-N torsion = -162.3°

H	0.4435462	1.2411014	1.4555103
C	1.1619187	1.1582301	0.6356652
C	1.0085088	-0.2012427	-0.0660131
N	0.1341698	0.0872291	-1.1883380
C	-0.3279602	1.4580680	-1.1946665
C	0.7815576	2.1700432	-0.4427750
H	2.1627301	1.3112315	1.0381196
C	0.3884273	-1.2186912	0.8991339
H	1.9707163	-0.5571062	-0.4398259
H	-0.3805984	-2.9489431	2.5046413
H	-1.2782771	1.5676301	-0.6591734
H	-0.4615129	1.8095789	-2.2159376
H	1.6276105	2.3455761	-1.1156986
C	-0.2321555	-0.7848654	-2.1543802
O	-1.0003802	-0.4527641	-3.0492321
C	0.3452798	-2.1712854	-2.0811429
H	0.1799159	-2.6615680	-3.0377450
H	-0.1665675	-2.7362272	-1.2988250
H	1.4128530	-2.1684464	-1.8525769
O	-0.8177433	-1.4166940	0.8733162
C	0.6595469	-2.7490561	2.7458203
H	0.7196176	-2.3161237	3.7494668
H	1.2246791	-3.6844705	2.7376411
N	0.2929217	3.4420414	0.0916599
N	1.1472576	4.2481397	0.4164559
N	1.8518247	5.0641113	0.7413575
C	2.6241675	-1.5662580	1.9331790
H	3.1785295	-2.5081955	1.9502481
H	3.0213813	-0.9567639	1.1276258
H	2.8052995	-1.0465520	2.8798025
N	1.2145845	-1.8385532	1.7671073

Ac-(4S)Azp-NMe$_2$ (3S-NMe$_2$) exo cis
Ψ =163.2°, C$^{\delta}$-C$^{\gamma}$-N-N torsion = -86.5°

H	0.4908024	1.2683949	1.3756681
C	1.1946562	1.1549483	0.5468703
C	1.0179928	-0.2214353	-0.1155399
N	0.1482924	0.0471062	-1.2461726
C	-0.3140468	1.4171544	-1.2728282
C	0.8127798	2.1416015	-0.5445852
H	2.2017815	1.3104597	0.9307761
C	0.3818237	-1.2031405	0.8762288
H	1.9741122	-0.6038878	-0.4784448
H	-0.4128772	-2.8720494	2.5340163
H	-1.2565306	1.5370840	-0.7259727
H	-0.4605531	1.7478040	-2.2998528
H	1.6530548	2.2947690	-1.2299833
C	-0.2202067	-0.8430861	-2.1949671
O	-0.9876517	-0.5270656	-3.0962299
C	0.3555845	-2.2284158	-2.0938357
H	0.1735674	-2.7442556	-3.0337876
H	-0.1425781	-2.7714035	-1.2874891
H	1.4270558	-2.2201276	-1.8842102
O	-0.8251907	-1.3953047	0.8449979
C	0.6268857	-2.6715547	2.7763787
H	0.6825497	-2.2045609	3.7648957
H	1.1848766	-3.6108214	2.8049880
N	0.4072911	3.4167356	0.0492004
N	0.4642575	4.3905259	-0.6826220
N	0.4869602	5.3528510	-1.2659067
C	2.6061781	-1.5298746	1.9396747
H	3.1519354	-2.4744295	2.0075648
H	3.0167107	-0.9636443	1.1095092
H	2.7824486	-0.9667277	2.8620648
N	1.1962938	-1.7995024	1.7712464

Ac-(4S)Azp-NMe$_2$ (3S-NMe$_2$) exo cis
Ψ =-38.4°, C$^{\delta}$-C$^{\gamma}$-N-N torsion = -90.6°

H	-0.0898738	1.7158683	1.1493353
C	0.8385014	1.4836612	0.6212703
C	0.8252762	0.0517833	0.0810053
N	0.2231514	0.1882384	-1.2409834
C	0.0161273	1.5737251	-1.6175195
C	0.9139179	2.3293035	-0.6393508
H	1.6679691	1.6509536	1.3059623
C	0.2019342	-0.9313577	1.0742215
H	1.8533210	-0.3074130	-0.0165475
H	-0.8826018	-2.7459649	2.3701058
H	-1.0280935	1.8824159	-1.4874210
H	0.2821809	1.7255984	-2.6629987
H	1.9408079	2.3463782	-1.0184980
C	0.0339123	-0.8089316	-2.1375597
O	-0.4361259	-0.5774675	-3.2445870
C	0.4053898	-2.2000410	-1.7054605
H	0.4556650	-2.8298794	-2.5906324
H	-0.3613778	-2.6001810	-1.0367825
H	1.3571375	-2.2338287	-1.1725848
O	0.9777421	-1.5590637	1.7873434
C	-1.6796064	-2.0872773	2.0364172
H	-2.4336724	-2.6665270	1.4976868
H	-2.1521409	-1.6269406	2.9092264
N	0.4513782	3.6914810	-0.3700266
N	0.8911739	4.5628807	-1.1014179
N	1.2406093	5.4432054	-1.7090560
N	-1.1346513	-1.0739842	1.1561424
C	-2.1223968	-0.2970037	0.4376672
H	-1.7556555	0.6902637	0.1783251
H	-2.4522571	-0.8026500	-0.4752223
H	-2.9893137	-0.1626942	1.0869355

Ac-(4S)Azp-NMe$_2$ (3S-NMe$_2$) exo cis
Ψ =-38.4°, C$^{\delta}$-C$^{\gamma}$-N-N torsion = -162.0°

H	-0.0371964	1.6750531	1.2165381
C	0.8947182	1.4331690	0.6994030
C	0.8554960	0.0149537	0.1242878
N	0.2751812	0.1990498	-1.2015048
C	0.1138312	1.5977986	-1.5509064
C	1.0112608	2.3051915	-0.5479374
H	1.7171706	1.5613099	1.4015973
C	0.1938500	-0.9741331	1.0860237
H	1.8757862	-0.3670187	0.0321851

H	-0.9536968	-2.7858804	2.3299439
H	-0.9234819	1.9334340	-1.4319164
H	0.4014834	1.7657567	-2.5876190
H	2.0442395	2.3031610	-0.9116129
C	0.0689211	-0.7718862	-2.1225468
O	-0.3801664	-0.5024032	-3.2296548
C	0.3950895	-2.1825616	-1.7180027
H	0.4405422	-2.7928577	-2.6170220
H	-0.3922023	-2.5764164	-1.0697712
H	1.3377479	-2.2557438	-1.1730519
O	0.9436642	-1.6369439	1.7952218
C	-1.7295019	-2.1014907	1.9980709
H	-2.4886619	-2.6512445	1.4360807
H	-2.2045760	-1.6478143	2.8729871
N	0.5489310	3.6803258	-0.3563715
N	1.3534720	4.4488192	0.1431144
N	2.0169197	5.2400549	0.5907253
C	-2.1052691	-0.2677104	0.4323114
H	-1.7101571	0.7142890	0.1956901
H	-2.4366232	-0.7474732	-0.4939035
H	-2.9765274	-0.1232322	1.0735652
N	-1.1469926	-1.0837067	1.1475220

Ac-(4S)Azp-NMe$_2$ (3S-NMe$_2$) exo cis

Ψ =-38.8°, C^δ-C^γ-N-N torsion = 61.9°

H	-0.0355165	1.6382449	1.2558174
C	0.8856539	1.4250319	0.7049920
C	0.8490358	0.0113290	0.1191385
N	0.2486172	0.1965049	-1.1980091
C	0.0675138	1.5935244	-1.5403103
C	0.9683790	2.3090831	-0.5355498
H	1.7221879	1.5599700	1.3879527
C	0.2087702	-0.9905772	1.0826259
H	1.8713816	-0.3603495	0.0107430
H	-0.9086534	-2.8141268	2.3358584
H	-0.9793385	1.9040350	-1.4238357
H	0.3481203	1.7722575	-2.5770858
H	1.9912177	2.3215748	-0.9114565
C	0.0404431	-0.7726923	-2.1218008
O	-0.4273436	-0.5013951	-3.2202392
C	0.3885094	-2.1812688	-1.7293784
H	0.4266854	-2.7867309	-2.6319893
H	-0.3841447	-2.5869748	-1.0709046
H	1.3403278	-2.2462999	-1.1995593
O	0.9734500	-1.6545171	1.7741733
C	-1.6941552	-2.1344284	2.0175392
H	-2.4560550	-2.6875336	1.4626065
H	-2.1613645	-1.6884720	2.9005296
N	0.6362021	3.7186610	-0.3196314
N	-0.4723067	3.9470739	0.1360696
N	-1.4671103	4.2680223	0.5550728
C	-2.1049936	-0.2947495	0.4684811
H	-1.7217718	0.6924249	0.2337994
H	-2.4443628	-0.7691326	-0.4575002
H	-2.9687115	-0.1614764	1.1220141
N	-1.1305014	-1.1077138	1.1646898

Ac-(4S)Azp-NMe$_2$ (3S-NMe$_2$) exo trans

Ψ =132.8°, C^δ-C^γ-N-N torsion = 63.5°

H	0.0848596	1.5627979	1.3823994
C	0.9222402	1.5063431	0.6814715
C	1.0096292	0.0849182	0.1207496
N	0.2424957	0.1542223	-1.1171320
C	-0.3202672	1.4635449	-1.3569638
C	0.6148576	2.3592010	-0.5464013
H	1.8330898	1.8113393	1.1945499
C	0.3617303	-0.9088369	1.0874657
H	2.0370212	-0.1848214	-0.1187652
H	-0.4129437	-2.3818699	2.9298382
H	-1.3468723	1.5339141	-0.9748219
H	-0.3225362	1.7186477	-2.4158499
H	1.5258003	2.5354744	-1.1186226
C	0.1187760	-0.9436670	-1.8877599
O	0.6705953	-1.9926194	-1.5681306
C	-0.7105065	-0.8131500	-3.1337259
H	-0.8026066	-1.7938535	-3.5944610
H	-0.2354280	-0.1286609	-3.8412953
H	-1.7035740	-0.4195457	-2.9063501
O	-0.8387814	-0.8033393	1.3120315
C	0.5701260	-2.7399636	2.6356074
H	1.2084308	-2.8200041	3.5179821
H	0.4651909	-3.7329862	2.1867391
N	0.0878097	3.6959857	-0.2701599
N	-0.9192111	3.7534658	0.4177050
N	-1.8394198	3.9271557	1.0424187
C	2.5282227	-2.0584874	1.2931989
H	2.5867916	-2.3098107	0.2320020
H	3.1772543	-1.2058416	1.5051745
H	2.8996709	-2.9058039	1.8669847
N	1.1543582	-1.8095182	1.6932084

Ac-(4S)Azp-NMe$_2$ (3S-NMe$_2$) exo trans

Ψ =133.1°, C^δ-C^γ-N-N torsion = -164.5°

H	0.1234852	1.5600152	1.3975904
C	0.9723794	1.4768222	0.7152716
C	1.0331673	0.0592462	0.1421330
N	0.2617857	0.1576422	-1.0903621
C	-0.2593417	1.4870179	-1.3216519
C	0.6972372	2.3457225	-0.5100174
H	1.8846719	1.7524697	1.2435870
C	0.3738574	-0.9337842	1.1018066
H	2.0543423	-0.2270248	-0.1054121
H	-0.4141646	-2.4146197	2.9333235
H	-1.2780193	1.5986858	-0.9318540
H	-0.2554241	1.7453942	-2.3799013
H	1.6191519	2.5054027	-1.0797326
C	0.1034610	-0.9300778	-1.8676615
O	0.6261606	-1.9972919	-1.5582978
C	-0.7268560	-0.7671032	-3.1093366
H	-0.8536943	-1.7424169	-3.5732757
H	-0.2318424	-0.0964838	-3.8165781
H	-1.7050355	-0.3409280	-2.8764068
O	-0.8243002	-0.8161899	1.3314279
C	0.5606551	-2.7854974	2.6277099
H	1.2021510	-2.8915278	3.5049525
H	0.4349145	-3.7690790	2.1634681
N	0.0696891	3.6323860	-0.2045444
N	0.8198790	4.5130887	0.1788924
N	1.4225759	5.3945423	0.5357555
C	2.5243681	-2.1169698	1.2879639
H	2.5726520	-2.3723928	0.2270565
H	3.1849782	-1.2714923	1.4921736
H	2.8903049	-2.9664079	1.8621796
N	1.1567001	-1.8503677	1.6974207

Ac-(4S)Azp-NMe$_2$ (3S-NMe$_2$) exo trans

Ψ =133.2°, C^δ-C^γ-N-N torsion = -89.9°

H	0.1069972	1.5946736	1.3340745
C	0.9535165	1.5005052	0.6501509
C	1.0209399	0.0718607	0.1067108
N	0.2545773	0.1403275	-1.1310741
C	-0.2842941	1.4587927	-1.3813805
C	0.6722123	2.3437380	-0.5835914
H	1.8644646	1.7942730	1.1699054
C	0.3618962	-0.9051504	1.0827911
H	2.0440114	-0.2155121	-0.1312959
H	-0.4309594	-2.3583060	2.9336426
H	-1.3017345	1.5645558	-0.9871229
H	-0.2883334	1.6987962	-2.4444305
H	1.5915453	2.4990398	-1.1579726
C	0.1115459	-0.9625272	-1.8903276
O	0.6449929	-2.0181529	-1.5606943
C	-0.7156178	-0.8300624	-3.1377298
H	-0.8266090	-1.8139009	-3.5875250
H	-0.2275746	-0.1629645	-3.8530941
H	-1.7008422	-0.4148773	-2.9147667
O	-0.8387261	-0.7914992	1.3014558
C	0.5512509	-2.7247562	2.6470046
H	1.1841487	-2.8038055	3.5333410
H	0.4419070	-3.7198339	2.2036216
N	0.0887847	3.6323989	-0.2070709
N	0.2442992	4.5440930	-1.0012205
N	0.3369852	5.4537715	-1.6579692
C	2.5201195	-2.0670682	1.3097695

H	2.5793018	-2.3480828	0.2558643
H	3.1708192	-1.2103133	1.4978735
H	2.8898627	-2.8983003	1.9077052
N	1.1468095	-1.8039591	1.7022678

Ac-(4S)Azp-NMe$_2$ (3S-NMe$_2$) exo trans

Ψ =-39.6°, C^δ-C^γ-N-N torsion = -92.7°

H	-0.1846731	1.7408143	1.1255986
C	0.7508273	1.5133418	0.6075046
C	0.7517868	0.0824548	0.0710162
N	0.1374315	0.2109785	-1.2455044
C	-0.0638333	1.5930385	-1.6349314
C	0.8333524	2.3532733	-0.6563048
H	1.5734739	1.6922091	1.2973376
C	0.1550348	-0.9192550	1.0597167
H	1.7772841	-0.2759574	-0.0496497
H	-2.3569413	-2.7741284	1.4635971
H	-1.1057269	1.9114884	-1.5028478
H	0.2173460	1.7657027	-2.6736261
H	1.8613701	2.3628910	-1.0323102
C	-0.0465933	-0.8760568	-2.0273085
O	0.2436806	-1.9941595	-1.6220880
C	-0.6292166	-0.6365842	-3.3924434
H	-0.8858090	-1.5971402	-3.8330131
H	0.1022195	-0.1369043	-4.0333724
H	-1.5170244	-0.0022184	-3.3466379
O	0.9477840	-1.5170642	1.7788988
C	-1.6970757	-2.1139101	2.0334977
H	-2.2693838	-1.6747651	2.8561011
H	-0.8712584	-2.6905699	2.4400927
N	0.3710695	3.7190290	-0.4069693
N	0.8479387	4.5867657	-1.1191418
N	1.2292602	5.4636739	-1.7124815
N	-1.1792919	-1.0775958	1.1655138
C	-2.1843495	-0.3585831	0.4171362
H	-1.8584121	0.6422854	0.1520815
H	-2.4734508	-0.8936993	-0.4936037
H	-3.0703498	-0.2551976	1.0463977

Ac-(4S)Azp-NMe$_2$ (3S-NMe$_2$) exo trans

Ψ =-39.6°, C^δ-C^γ-N-N torsion = -163.0°

H	-0.1219634	1.6959938	1.1862075
C	0.8133050	1.4527604	0.6751352
C	0.7787376	0.0338289	0.1076093
N	0.1799890	0.2098127	-1.2104197
C	0.0295994	1.6051474	-1.5754176
C	0.9348745	2.3146371	-0.5782421
H	1.6333848	1.5902392	1.3782270
C	0.1437227	-0.9697729	1.0700262
H	1.7945100	-0.3515764	-0.0119314
H	-2.4209732	-2.7584551	1.4164652
H	-1.0028940	1.9556106	-1.4496613
H	0.3282407	1.7889858	-2.6068226
H	1.9671436	2.2984152	-0.9438400
C	-0.0344148	-0.8542539	-2.0152572
O	0.2138718	-1.9896304	-1.6303034
C	-0.5968292	-0.5680090	-3.3798253
H	-0.8827387	-1.5102894	-3.8412917
H	0.1569704	-0.0815935	-4.0048365
H	-1.4624089	0.0959656	-3.3285622
O	0.9121474	-1.6063258	1.7823571
C	-1.7519397	-2.1254236	2.0062413
H	-2.3237695	-1.6853164	2.8286225
H	-0.9469598	-2.7303557	2.4135023
N	0.4835145	3.6959573	-0.4076978
N	1.2875630	4.4602852	0.0995771
N	1.9512590	5.2479980	0.5525318
C	-2.1724464	-0.3265603	0.4196511
H	-1.8148908	0.6686230	0.1746321
H	-2.4690817	-0.8369901	-0.5027390
H	-3.0605149	-0.2080607	1.0432931
N	-1.1957161	-1.0881917	1.1634753

Ac-(4S)Azp-NMe$_2$ (3S-NMe$_2$) exo trans

Ψ =-40.0°, C^δ-C^γ-N-N torsion = -63.4°

H	-0.1236589	1.6562953	1.2243325
C	0.8029627	1.4434748	0.6818840
C	0.7743727	0.0299185	0.1013791
N	0.1584409	0.2077072	-1.2090117
C	-0.0130370	1.6016516	-1.5652991
C	0.8929422	2.3195647	-0.5630023
H	1.6348969	1.5874717	1.3684823
C	0.1600707	-0.9879903	1.0626273
H	1.7927293	-0.3433032	-0.0327540
H	-2.3853817	-2.7987936	1.4301037
H	-1.0559218	1.9258440	-1.4430735
H	0.2804473	1.7986377	-2.5956529
H	1.9157886	2.3195571	-0.9391283
C	-0.0488890	-0.8551564	-2.0189260
O	0.2191621	-1.9883494	-1.6423577
C	-0.6281544	-0.5683163	-3.3760125
H	-0.9050164	-1.5115169	-3.8410604
H	0.1126398	-0.0669766	-4.0047550
H	-1.5027265	0.0826996	-3.3121473
O	0.9422281	-1.6245837	1.7592941
C	-1.7154528	-2.1630240	2.0158584
H	-2.2817932	-1.7319896	2.8467074
H	-0.9007099	-2.7629684	2.4108279
N	0.5693643	3.7337077	-0.3676956
N	-0.5287799	3.9747705	0.1069163
N	-1.5135654	4.3078838	0.5394865
C	-2.1694148	-0.3582258	0.4458065
H	-1.8261876	0.6437618	0.2075316
H	-2.4690254	-0.8612091	-0.4796047
H	-3.0527229	-0.2553517	1.0786634
N	-1.1774017	-1.1162858	1.1726930

Ac-(4S)Azp-OMe (3S-OMe) exo cis

Ψ =158.2°, C^δ-C^γ-N-N torsion =60.8°

H	0.0805319	1.3198021	1.4096826
C	0.9308106	1.2262164	0.7266837
C	0.9253186	-0.1659484	0.0754219
N	0.1919224	0.0276339	-1.1607990
C	-0.2412514	1.3972918	-1.3459910
C	0.7328155	2.1658753	-0.4593406
H	1.8442240	1.4173836	1.2861245
C	0.2621491	-1.1542322	1.0159743
H	1.9482690	-0.4990703	-0.1175815
H	-0.1115565	-2.0138997	3.4826301
H	-1.2756259	1.5360927	-1.0072286
H	-0.1863179	1.6761474	-2.3966319
H	1.6760247	2.2982592	-0.9893220
C	-0.0250967	-0.9122748	-2.1124066
O	-0.6521293	-0.6455257	-3.1286564
C	0.5276439	-2.2870585	-1.8575252
H	0.5374629	-2.8307539	-2.7994377
H	-0.1220821	-2.8161630	-1.1559308
H	1.5351142	-2.2666194	-1.4378819
O	-0.9097451	-1.4236328	1.0178152
O	1.1503465	-1.6448284	1.8721137
C	0.6315302	-2.5286230	2.8729704
H	1.4853512	-2.8224439	3.4777274
H	0.1751156	-3.4011704	2.4050583
N	0.3097623	3.5219899	-0.1106158
N	-0.7349418	3.6225907	0.5130461
N	-1.6784970	3.8337346	1.0896091

Ac-(4S)Azp-OMe (3S-OMe) exo cis

Ψ =159.1°, C^δ-C^γ-N-N torsion =-161.5°

H	0.1144653	1.3194672	1.4209662
C	0.9679080	1.2053034	0.7472581
C	0.9429944	-0.1813025	0.0844269
N	0.2026013	0.0344942	-1.1432496
C	-0.2097280	1.4140534	-1.3104255
C	0.7808777	2.1571845	-0.4316303
H	1.8840585	1.3749714	1.3104310
C	0.2775881	-1.1707362	1.0217628
H	1.9604350	-0.5250774	-0.1198923
H	-0.0967633	-2.0401673	3.4850527
H	-1.2313836	1.5776576	-0.9495441
H	-0.1636577	1.6991206	-2.3598940
H	1.7265996	2.2855715	-0.9687555
C	-0.0341936	-0.8911913	-2.1029332
O	-0.6638807	-0.6050800	-3.1127309

C	0.5001453	-2.2768540	-1.8673592
H	0.4986074	-2.8087566	-2.8160456
H	-0.1540461	-2.8053077	-1.1695539
H	1.5094807	-2.2759495	-1.4516601
O	-0.8940921	-1.4407410	1.0231601
O	1.1658687	-1.6647956	1.8765591
C	0.6467833	-2.5522709	2.8737071
H	1.5002884	-2.8482736	3.4778997
H	0.1907299	-3.4232587	2.4024912
N	0.2350946	3.4603567	-0.0528044
N	1.0427546	4.2821929	0.3457116
N	1.7006994	5.1147121	0.7211533

Ac-(4S)Azp-OMe (3S-OMe) exo cis

Ψ =160.4°, C^{δ}-C^{γ}-N-N torsion =-87.6°

H	0.1229603	1.3453723	1.3610418
C	0.9640029	1.2178885	0.6740741
C	0.9339678	-0.1836985	0.0432792
N	0.1938534	0.0028371	-1.1891279
C	-0.2431632	1.3724668	-1.3703780
C	0.7559628	2.1435598	-0.5133435
H	1.8876663	1.4053188	1.2179531
C	0.2672080	-1.1513189	1.0026093
H	1.9502421	-0.5349622	-0.1534291
H	-0.1303478	-1.9458300	3.4884165
H	-1.2608352	1.5257052	-0.9940399
H	-0.2181140	1.6406215	-2.4255150
H	1.6937636	2.2693798	-1.0645850
C	-0.0282130	-0.9402687	-2.1355683
O	-0.6617660	-0.6788008	-3.1496078
C	0.5287792	-2.3131637	-1.8789932
H	0.5184763	-2.8662686	-2.8153666
H	-0.1045504	-2.8348715	-1.1572944
H	1.5452569	-2.2877233	-1.4817828
O	-0.9002498	-1.4387827	0.9950936
O	1.1493102	-1.6044941	1.8859078
C	0.6281251	-2.4670067	2.9036533
H	1.4769779	-2.7321827	3.5283772
H	0.1899922	-3.3585002	2.4543078
N	0.2576533	3.4417190	-0.0584198
N	0.4424470	4.3813088	-0.8139266
N	0.5672364	5.3159912	-1.4280023

Ac-(4S)Azp-OMe (3S-OMe) exo cis

Ψ =-30.4°, C^{δ}-C^{γ}-N-N torsion =-89.3°

H	-0.0049568	1.5105799	1.3000649
C	0.8786399	1.3545215	0.6754250
C	0.8924878	-0.0755731	0.1146344
N	0.2054486	0.0465067	-1.1594404
C	-0.2053252	1.4074072	-1.4418785
C	0.7455005	2.2190882	-0.5674798
H	1.7653922	1.5719366	1.2674396
C	0.2507104	-1.0126640	1.1188865
H	1.9191833	-0.4242554	-0.0210594
H	-1.4010668	-2.9181158	1.8862090
H	-1.2429630	1.5876556	-1.1392965
H	-0.1124319	1.6175748	-2.5064358
H	1.7149033	2.3154992	-1.0674497
C	0.0103686	-0.9502415	-2.0544894
O	-0.5774006	-0.7464087	-3.1085816
C	0.5353974	-2.3103846	-1.6855628
H	0.5463918	-2.9274229	-2.5810314
H	-0.1289306	-2.7769052	-0.9532267
H	1.5374016	-2.2725054	-1.2546931
O	0.8821199	-1.5914079	1.9659761
O	-1.0669021	-1.0724482	0.9908951
C	-1.7527278	-1.8877350	1.9472041
H	-2.8052644	-1.8272812	1.6836126
H	-1.5863904	-1.5077976	2.9555354
N	0.2248723	3.5389706	-0.2110177
N	0.4852081	4.4451322	-0.9848821
N	0.6733540	5.3527420	-1.6228990

Ac-(4S)Azp-OMe (3S-OMe) exo cis

Ψ =-30.7°, C^{δ}-C^{γ}-N-N torsion =-161.4°

H	0.0199847	1.4658648	1.3662806
C	0.9099737	1.3148702	0.7497208
C	0.9089118	-0.0988154	0.1484770
N	0.2241677	0.0696643	-1.1215390
C	-0.1494115	1.4468295	-1.3774625
C	0.8036608	2.2148856	-0.4786463
H	1.7934069	1.4998102	1.3584957
C	0.2553782	-1.0553296	1.1261114
H	1.9317896	-0.4552338	0.0035875
H	-1.4242585	-2.9562145	1.8413952
H	-1.1866447	1.6455976	-1.0851390
H	-0.0377580	1.6802125	-2.4348376
H	1.7795607	2.3058452	-0.9672484
C	0.0066160	-0.9006277	-2.0399343
O	-0.5748880	-0.6578299	-3.0892386
C	0.4982334	-2.2816702	-1.7039420
H	0.5054501	-2.8733156	-2.6164640
H	-0.1847934	-2.7536863	-0.9926629
H	1.4959569	-2.2787909	-1.2616947
O	0.8799169	-1.6688372	1.9536982
O	-1.0634125	-1.0898732	1.0010486
C	-1.7596077	-1.9227821	1.9343621
H	-2.8116830	-1.8380503	1.6756526
H	-1.5850014	-1.5760605	2.9532096
N	0.2585118	3.5409433	-0.1893405
N	1.0565225	4.3698428	0.2145724
N	1.7060300	5.2102468	0.5867647

Ac-(4S)Azp-OMe (3S-OMe) exo cis

Ψ =-31.7°, C^{δ}-C^{γ}-N-N torsion =61.0°

H	0.0021979	1.4583813	1.3668471
C	0.8822042	1.3330494	0.7281827
C	0.8977798	-0.0842844	0.1359401
N	0.2078813	0.0625773	-1.1346038
C	-0.1959016	1.4280735	-1.4001460
C	0.7459097	2.2242432	-0.5028467
H	1.7701259	1.5411753	1.3215242
C	0.2570228	-1.0430880	1.1203716
H	1.9250492	-0.4274111	-0.0082524
H	-1.4176495	-2.9426649	1.8535127
H	-1.2431330	1.5944388	-1.1178799
H	-0.0879434	1.6594744	-2.4582588
H	1.7127977	2.3282181	-0.9950768
C	0.0036130	-0.9201398	-2.0440123
O	-0.5873719	-0.6962031	-3.0917541
C	0.5222505	-2.2885068	-1.6979863
H	0.5316806	-2.8897483	-2.6041757
H	-0.1457424	-2.7650384	-0.9754171
H	1.5236445	-2.2630966	-1.2648923
O	0.8900371	-1.6558695	1.9416985
O	-1.0629800	-1.0776001	1.0077668
C	-1.7511040	-1.9088545	1.9487488
H	-2.8055064	-1.8232190	1.7001274
H	-1.5661866	-1.5610038	2.9653659
N	0.3191138	3.5965122	-0.2289336
N	-0.7493723	3.7278824	0.3464076
N	-1.7148900	3.9656477	0.8743756

Ac-(4S)Azp-OMe (3S-OMe) exo trans

Ψ =144.8°, C^{δ}-C^{γ}-N-N torsion =62.7°

H	0.0270572	1.3971772	1.3670936
C	0.8952192	1.3060276	0.7068023
C	0.9162918	-0.0890694	0.0756021
N	0.1720020	0.0796729	-1.1592679
C	-0.2479817	1.4454294	-1.3870036
C	0.7175066	2.2271747	-0.4973319
H	1.7941976	1.5191207	1.2818719
C	0.2505492	-1.1021707	0.9874714
H	1.9339976	-0.4246556	-0.1355801
H	0.0201348	-2.2514640	3.3475928
H	-1.2851077	1.6057586	-1.0651193
H	-0.1627543	1.7279978	-2.4355850
H	1.6682951	2.3452590	-1.0170914
C	-0.0457153	-0.9875900	-1.9578883
O	0.3885623	-2.0902176	-1.6465723
C	-0.8287756	-0.7460764	-3.2161060
H	-1.0179004	-1.7026638	-3.6972892
H	-0.2654553	-0.1061237	-3.9002624
H	-1.7771183	-0.2488893	-3.0008822

O	-0.9402011	-1.2034556	1.1284008
O	1.1512891	-1.8059601	1.6606859
C	0.6260711	-2.7550176	2.5935113
H	1.4904983	-3.2286087	3.0517470
H	0.0162531	-3.4941884	2.0732455
N	0.2916851	3.5917119	-0.1885824
N	-0.7472096	3.7077423	0.4425512
N	-1.6862012	3.9334582	1.0207541

Ac-(4S)Azp-OMe (3S-OMe) exo trans
Ψ =145.2°, C^{δ}-C^{γ}-N-N torsion =-162.9°

H	0.0593799	1.4015245	1.3697215
C	0.9327838	1.2843597	0.7228546
C	0.9325361	-0.1077199	0.0844349
N	0.1857862	0.0820952	-1.1450989
C	-0.2060712	1.4591542	-1.3625727
C	0.7744875	2.2139443	-0.4786142
H	1.8329723	1.4751244	1.3051202
C	0.2572044	-1.1168811	0.9933690
H	1.9441557	-0.4576287	-0.1334892
H	0.0209149	-2.2744910	3.3490845
H	-1.2315088	1.6505231	-1.0253261
H	-0.1225289	1.7425928	-2.4111770
H	1.7311155	2.3223394	-1.0008198
C	-0.0551541	-0.9741467	-1.9501832
O	0.3596899	-2.0877841	-1.6501368
C	-0.8391846	-0.7087444	-3.2032812
H	-1.0512571	-1.6584327	-3.6885814
H	-0.2651302	-0.0777732	-3.8868996
H	-1.7753131	-0.1919404	-2.9810945
O	-0.9339192	-1.2080451	1.1370466
O	1.1527286	-1.8329201	1.6615912
C	0.6201232	-2.7806279	2.5913351
H	1.4807028	-3.2649732	3.0456170
H	0.0016821	-3.5114205	2.0693759
N	0.2295678	3.5303817	-0.1478071
N	1.0302114	4.3483925	0.2728229
N	1.6823006	5.1780269	0.6640698

Ac-(4S)Azp-OMe (3S-OMe) exo trans
Ψ =145.7°, C^{δ}-C^{γ}-N-N torsion =-90.9°

H	0.0401988	1.4321921	1.3115374
C	0.9075250	1.3100010	0.6573016
C	0.9171575	-0.0947979	0.0477782
N	0.1691295	0.0614153	-1.1857309
C	-0.2516362	1.4267822	-1.4199725
C	0.7293727	2.2143953	-0.5523576
H	1.8099937	1.5245955	1.2265681
C	0.2511382	-1.0915257	0.9771955
H	1.9314460	-0.4409208	-0.1633977
H	0.0182467	-2.1934343	3.3600249
H	-1.2766501	1.6037872	-1.0740339
H	-0.1838156	1.6945265	-2.4742713
H	1.6801599	2.3266963	-1.0835947
C	-0.0513240	-1.0121089	-1.9742596
O	0.3836305	-2.1127867	-1.6559652
C	-0.8379420	-0.7811945	-3.2325513
H	-1.0297523	-1.7420476	-3.7041307
H	-0.2755333	-0.1488768	-3.9245616
H	-1.7848152	-0.2804870	-3.0193812
O	-0.9393360	-1.1988987	1.1149618
O	1.1533444	-1.7765316	1.6685199
C	0.6299353	-2.7089519	2.6187635
H	1.4952164	-3.1679974	3.0900688
H	0.0262854	-3.4623660	2.1118825
N	0.2172351	3.5229885	-0.1459458
N	0.4780187	4.4553562	-0.8875570
N	0.6674537	5.3840921	-1.4942975

Ac-(4S)Azp-OMe (3S-OMe) exo trans
Ψ =-34.6°, C^{δ}-C^{γ}-N-N torsion =-92.7°

H	-0.0224054	1.5042038	1.2777656
C	0.8591999	1.3553931	0.6484923
C	0.8639718	-0.0588971	0.0600861
N	0.1391093	0.0881788	-1.1898050
C	-0.2405202	1.4590875	-1.4622173
C	0.7310645	2.2421485	-0.5800072
H	1.7489052	1.5621325	1.2399555
C	0.2471064	-1.0492648	1.0259714
H	1.8797536	-0.4144627	-0.1278853
H	-1.4234335	-2.9739407	1.6772532
H	-1.2712990	1.6671760	-1.1522027
H	-0.1344914	1.7051596	-2.5186391
H	1.6983638	2.3274909	-1.0856698
C	-0.0936989	-0.9932354	-1.9640670
O	0.2980177	-2.1005818	-1.6153538
C	-0.8437563	-0.7627534	-3.2448541
H	-1.0537783	-1.7262991	-3.7030282
H	-0.2461898	-0.1613610	-3.9349244
H	-1.7795409	-0.2291551	-3.0646108
O	0.8968526	-1.6962585	1.8067935
O	-1.0781754	-1.0548197	0.9673278
C	-1.7360747	-1.9471219	1.8695394
H	-2.7997118	-1.8309870	1.6771942
H	-1.5030238	-1.6849175	2.9021739
N	0.2306521	3.5664151	-0.2117639
N	0.5579908	4.4866887	-0.9420716
N	0.8061726	5.4060558	-1.5417340

Ac-(4S)Azp-OMe (3S-OMe) exo trans
Ψ =-34.9°, C^{δ}-C^{γ}-N-N torsion =-162.6°

H	0.0086244	1.4561578	1.3405530
C	0.8950071	1.3076215	0.7181442
C	0.8786627	-0.0906944	0.0924433
N	0.1582114	0.1033993	-1.1534549
C	-0.1806931	1.4900275	-1.4006224
C	0.7957416	2.2267869	-0.4969183
H	1.7830878	1.4792159	1.3241433
C	0.2434149	-1.0952945	1.0311056
H	1.8889567	-0.4576796	-0.1033055
H	-1.4666755	-3.0022734	1.6301382
H	-1.2094002	1.7194389	-1.0986091
H	-0.0561527	1.7562954	-2.4496676
H	1.7695177	2.3017753	-0.9926515
C	-0.0995473	-0.9532561	-1.9530497
O	0.2635462	-2.0780241	-1.6296108
C	-0.8404406	-0.6743632	-3.2295290
H	-1.0783301	-1.6216674	-3.7075159
H	-0.2234383	-0.0774908	-3.9063256
H	-1.7599482	-0.1162989	-3.0400247
O	0.8811415	-1.7773857	1.7917788
O	-1.0817748	-1.0709286	0.9761218
C	-1.7567261	-1.9751103	1.8534959
H	-2.8180372	-1.8305940	1.6675927
H	-1.5159395	-1.7485740	2.8927863
N	0.2788325	3.5615820	-0.1964978
N	1.0912441	4.3636227	0.2329382
N	1.7557687	5.1808134	0.6290281

Ac-(4S)Azp-OMe (3S-OMe) exo trans
Ψ =-35.4°, C^{δ}-C^{γ}-N-N torsion =62.8°

H	-0.0127449	1.4477355	1.3433416
C	0.8666753	1.3264015	0.7029479
C	0.8689546	-0.0744644	0.0829704
N	0.1460452	0.1001016	-1.1651051
C	-0.2222584	1.4764871	-1.4198827
C	0.7422677	2.2397185	-0.5131029
H	1.7568319	1.5196401	1.2980386
C	0.2464120	-1.0832998	1.0261116
H	1.8845844	-0.4272451	-0.1102744
H	-1.4499771	-3.0001022	1.6372526
H	-1.2624395	1.6741516	-1.1298182
H	-0.0994615	1.7435705	-2.4688072
H	1.7084633	2.3275793	-1.0098978
C	-0.0932896	-0.9674277	-1.9574954
O	0.2897867	-2.0823908	-1.6249471
C	-0.8390139	-0.7106510	-3.2354852
H	-1.0559605	-1.6649965	-3.7093635
H	-0.2352228	-0.1030230	-3.9145687
H	-1.7708276	-0.1724090	-3.0484596
O	0.8917219	-1.7575862	1.7869360
O	-1.0792146	-1.0689817	0.9743838
C	-1.7456660	-1.9739467	1.8577320
H	-2.8084448	-1.8365523	1.6750034

H	-1.5025134	-1.7416685	2.8951671
N	0.3447167	3.6180095	-0.2274129
N	-0.7053962	3.7656113	0.3775195
N	-1.6524612	4.0182900	0.9311097

5. Torsion Potential of Methylesters and Dimethylamides of Proline Derivatives

The absolute and local energy minima of the *endo* and *exo* puckered *cis* and *trans* conformers of Ac-Pro-OMe (**1-OMe**) and Ac-Pro-NMe$_2$ (**1-NMe$_2$**) were confirmed by determining the torsion potential of the ψ-angle in the gas phase by constrained geometry optimization (relaxed scan) at the PBE0-D3/def2-TZVP level of theory.

5.1. Ac-Pro-OMe (1-OMe) - *trans-endo*

Table 14 Torsion potential of the ψ-angle in the *trans-endo* conformation of Ac-Pro-OMe (**1-OMe**). Calculated in the gas phase by constrained geometry optimization (relaxed scan) at the PBE0-D3/def2-TZVP level of theory.

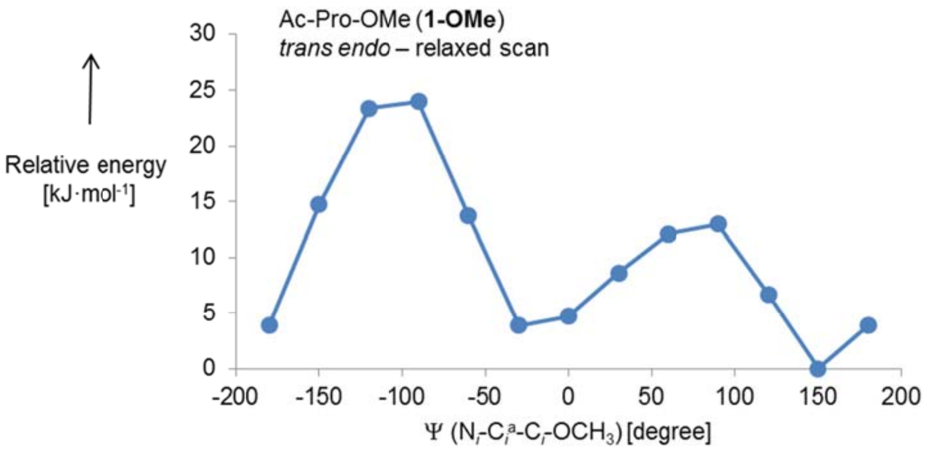

Ac-Pro-OMe *trans endo* – relaxed scan	
Ψ (N$_i$-C$_i^{\alpha}$-C$_i$-OCH$_3$) [degree]	relative energy E$_{rel}$ [kJ·mol^{-1}]
-180	3.9
-150	14.7
-120	23.4
-90	24
-60	13.7
-30	3.9
0	4.7
30	8.6
60	12.1
90	13
120	6.7
150	0
180	3.9

5.2. Ac-Pro-OMe (1-OMe) - *cis-endo*

Table 15 Torsion potential of the ψ-angle in the *cis-endo* conformation of Ac-Pro-OMe (**1-OMe**). Calculated in the gas phase by constrained geometry optimization (relaxed scan) at the PBE0-D3/def2-TZVP level of theory.

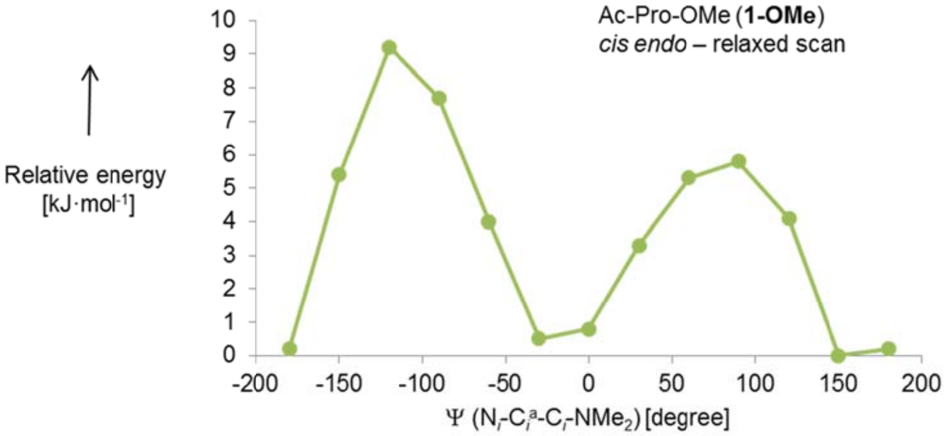

**Ac-Pro-OMe *cis endo* – relaxed scan**	
Ψ (N_i-C_i^a-C_i-OCH_3) [degree]	relative energy E_{rel} [kJ·mol^{-1}]
-180	0.2
-150	5.4
-120	9.2
-90	7.7
-60	4
-30	0.5
0	0.8
30	3.3
60	5.3
90	5.8
120	4.1
150	0
180	0.2

5.3. Ac-Pro-NMe$_2$ (1-NMe$_2$)- *trans-endo*

Table 16 Torsion potential of the ψ-angle in the *trans-endo* conformation of Ac-Pro-NMe$_2$ (**1-NMe$_2$**). Calculated in the gas phase by constrained geometry optimization (relaxed scan) at the PBE0-D3/def2-TZVP level of theory. **Note:** The structure at Ψ = -30° was confirmed to be a minimum by vibrational frequency calculations.

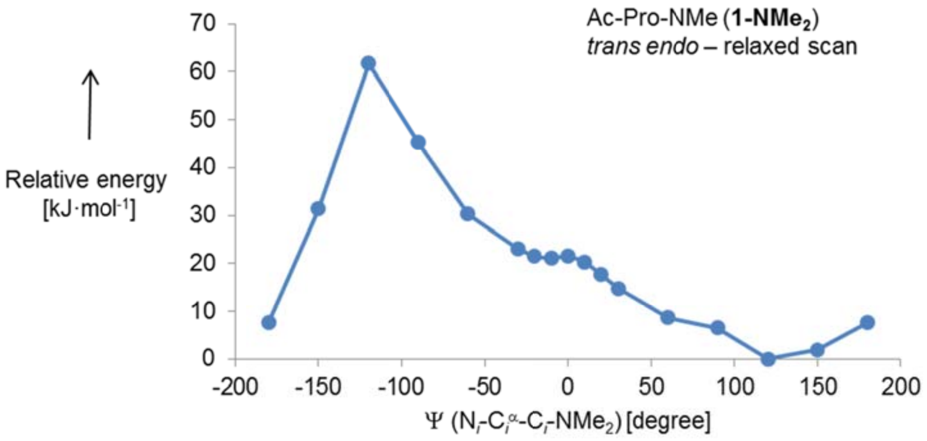

Ac-Pro-NMe$_2$ (**1-NMe$_2$**) *trans endo*- relaxed scan	
Ψ (N$_i$-C$_i^a$-C$_i$-OCH$_3$) [degree]	relative energy E$_{rel}$ [kJ·mol^{-1}]
-180	7.6
-150	31.3
-120	61.8
-90	45.4
-60	30.3
-30	23
-20	21.5
-10	21.1
0	21.6
10	20.3
20	17.7
30	14.8
60	8.7
90	6.5
120	0
150	1.9
180	7.6

5.4. Ac-Pro-NMe_2 (1-NMe_2)- *cis-endo*

Table 17 Torsion potential of the ψ-angle in the *cis-endo* conformation of Ac-Pro-NMe_2 (**1-NMe_2**). Calculated in the gas phase by constrained geometry optimization (relaxed scan) at the PBE0-D3/def2-TZVP level of theory.

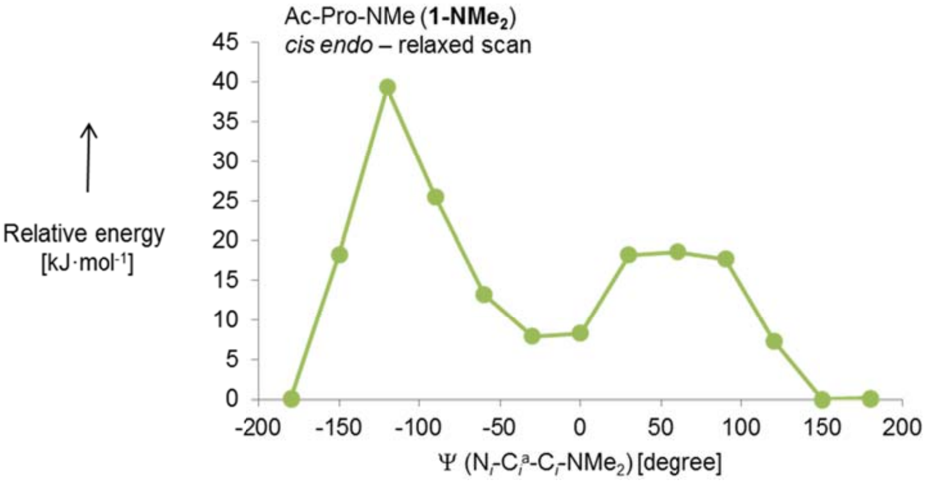

**Ac-Pro-NMe_2 *cis endo* - relaxed scan**	
Ψ (N_i-C_i^a-C_i-OCH_3) [degree]	relative energy E_{rel} [kJ·mol^{-1}]
-180	0.1
-150	18.3
-120	39.4
-90	25.5
-60	13.2
-30	7.9
0	8.3
30	18.2
60	18.6
90	17.7
120	7.3
150	0.0
180	0.1

5.5. Torsion Potential of the Azide Substituent in Ac-(4*R*)Azp-OMe (3R-OMe) and Ac-(4*S*)Azp-OMe (3S-OMe)

The torsion potential of the C^{δ}-C^{γ}-N-N torsion angle was determined by constrained geometry optimization (relaxed scan) at the PBE0-D3/def2-TZVP level of theory.

5.5.1. Ac-(4*R*)Azp-OMe (3R-OMe) - *trans-exo*

Table 18 Potential of the the C^{δ}-C^{γ}-N-N torsion angle in the *trans-exo* conformation of Ac-(4*R*)Azp-OMe (**3R-OMe**). Calculated in the gas phase by constrained geometry optimization (relaxed scan) at the PBE0-D3/def2-TZVP level of theory.

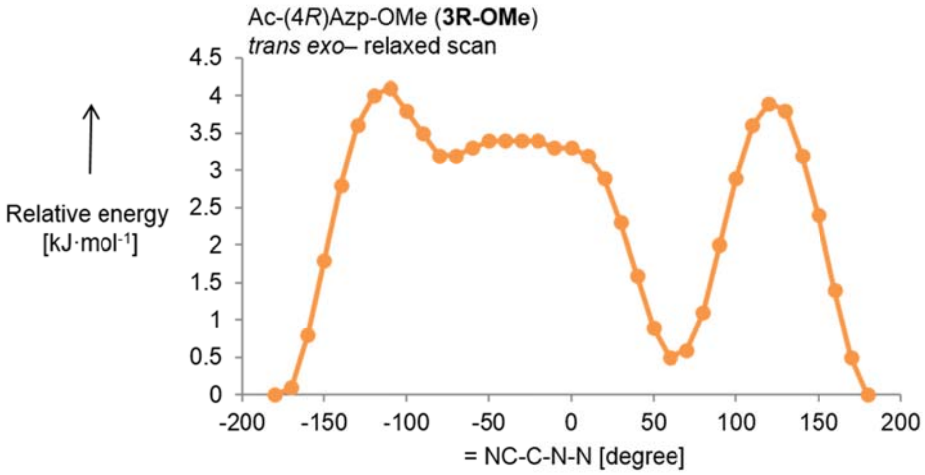

Ac-(4*R*)Azp-OMe (3R-OMe) *trans exo*- relaxed scan	
C^{δ}-C^{γ}-N-N torsion [degree]	relative energy E_{rel} [kJ·mol^{-1}]
-180	0
-170	0.1
-160	0.8
-150	1.8
-140	2.8
-130	3.6
-120	4
-110	4.1
-100	3.8
-90	3.5
-80	3.2
-70	3.2
-60	3.3
-50	3.4
-40	3.4
-30	3.4
-20	3.4
-10	3.3
0	3.3
10	3.2
20	2.9
30	2.3
40	1.6

50	0.9
60	0.5
70	0.6
80	1.1
90	2
100	2.9
110	3.6
120	3.9
130	3.8
140	3.2
150	2.4
160	1.4
170	0.5
180	0
-180	0
-170	0.1

5.5.2. Ac-(4*S*)Azp-OMe (3S-OMe) - *trans-endo*

Table 19 Potential of the the C^{δ}-C^{γ}-N-N torsion angle in the *trans-endo* conformation of Ac-(4*S*)Azp-OMe (**3S-OMe**). Calculated in the gas phase by constrained geometry optimization (relaxed scan) at the PBE0-D3/def2-TZVP level of theory. **Note:** Data points between 50°-70° were excluded since the system prefers to convert from the *endo* to *exo* conformation, which are considered separately.

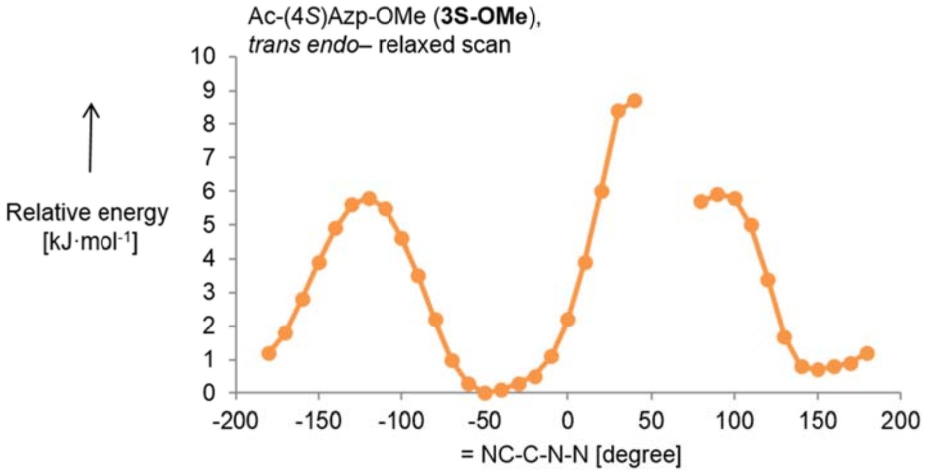

Ac-(4*S*)Azp-OMe (3S-OMe) *trans endo*- relaxed scan	
C^{δ}-C^{γ}-N-N torsion [degree]	relative energy E_{rel} [kJ·mol^{-1}]
-180	1.2
-170	1.8
-160	2.8
-150	3.9
-140	4.9
-130	5.6
-120	5.8
-110	5.5
-100	4.6
-90	3.5
-80	2.2
-70	1
-60	0.3
-50	0
-40	0.1
-30	0.3
-20	0.5
-10	1.1
0	2.2
10	3.9
20	6
30	8.4
40	8.7
50	
60	
70	
80	5.7
90	5.9
100	5.8

110	5
120	3.4
130	1.7
140	0.8
150	0.7
160	0.8
170	0.9
180	1.2
-180	1.2
-170	1.8

6. NMR Spectra

6.1. Ac-Pro-OMe (1-OMe) 80mM in D_2O

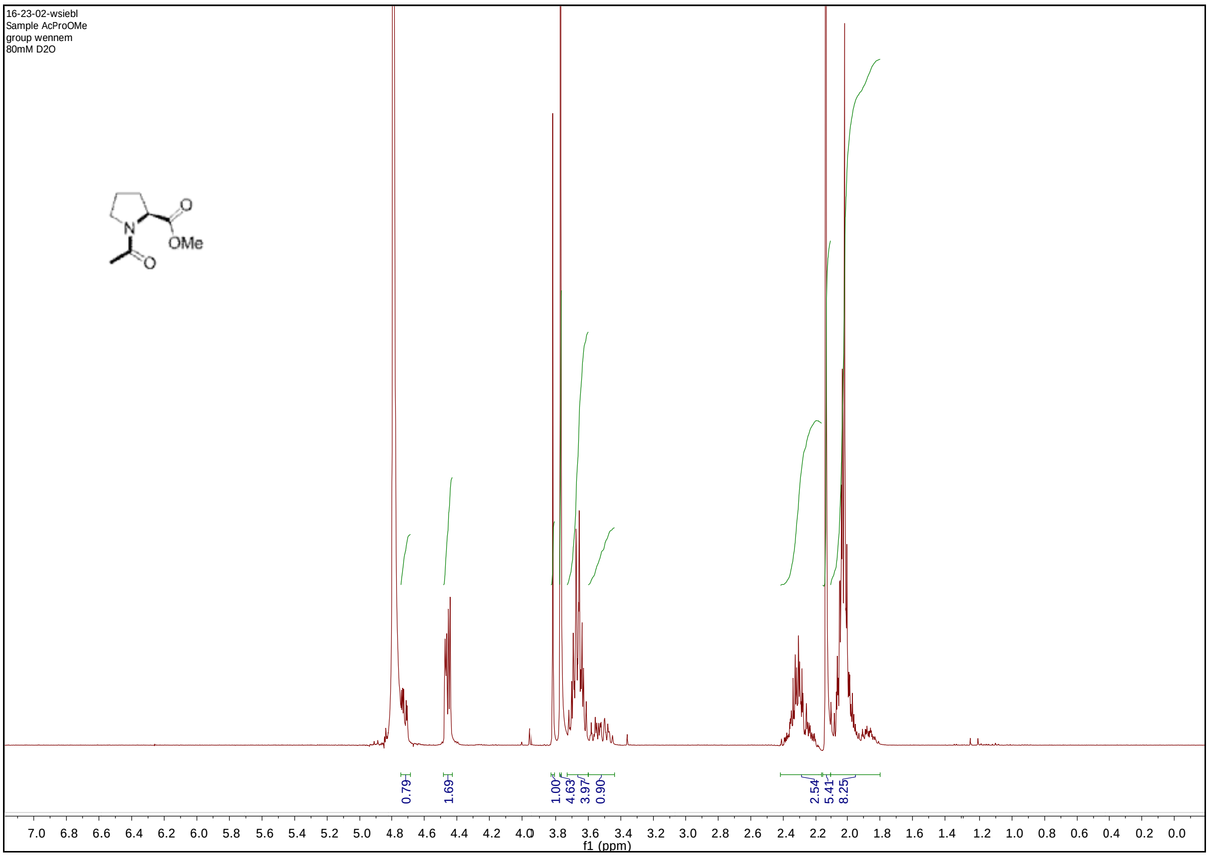

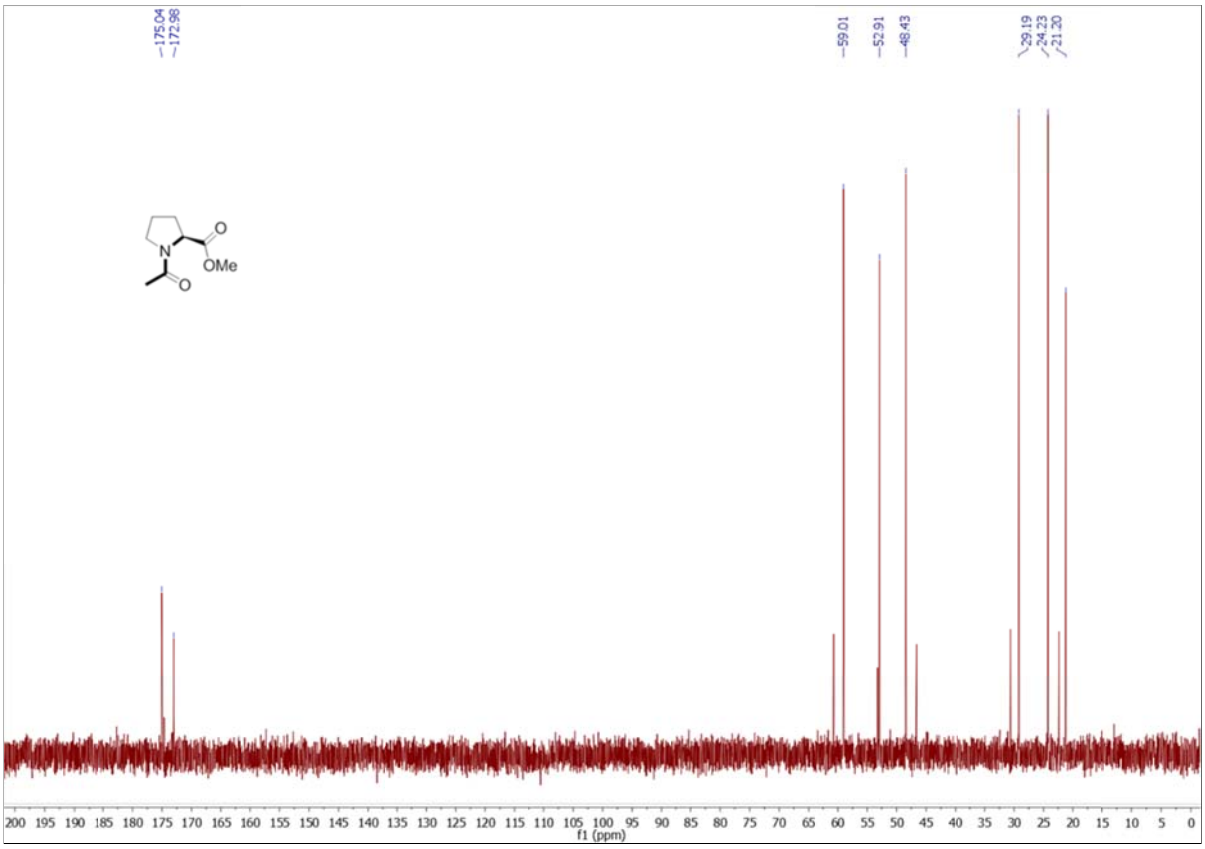

6.2. Ac-Pro-OMe (1-OMe) 80mM in $CDCl_3$

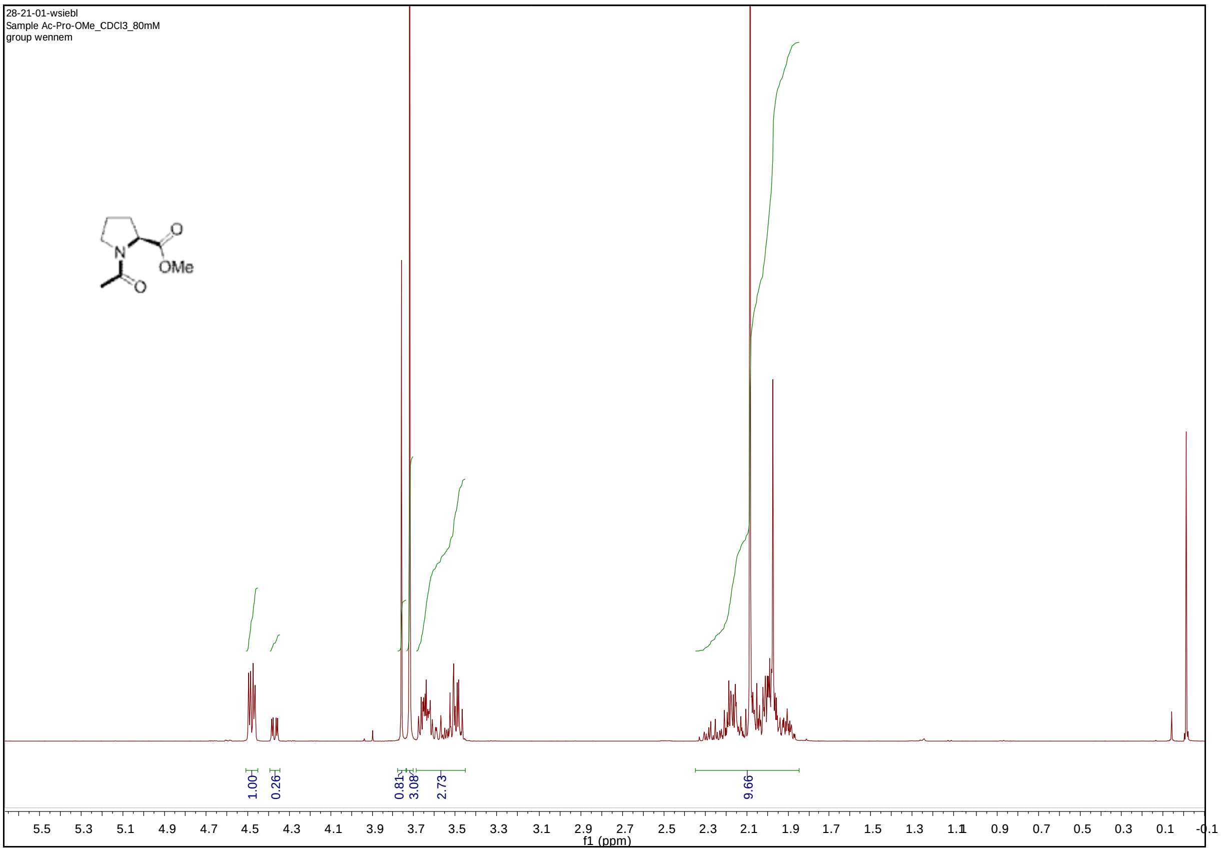

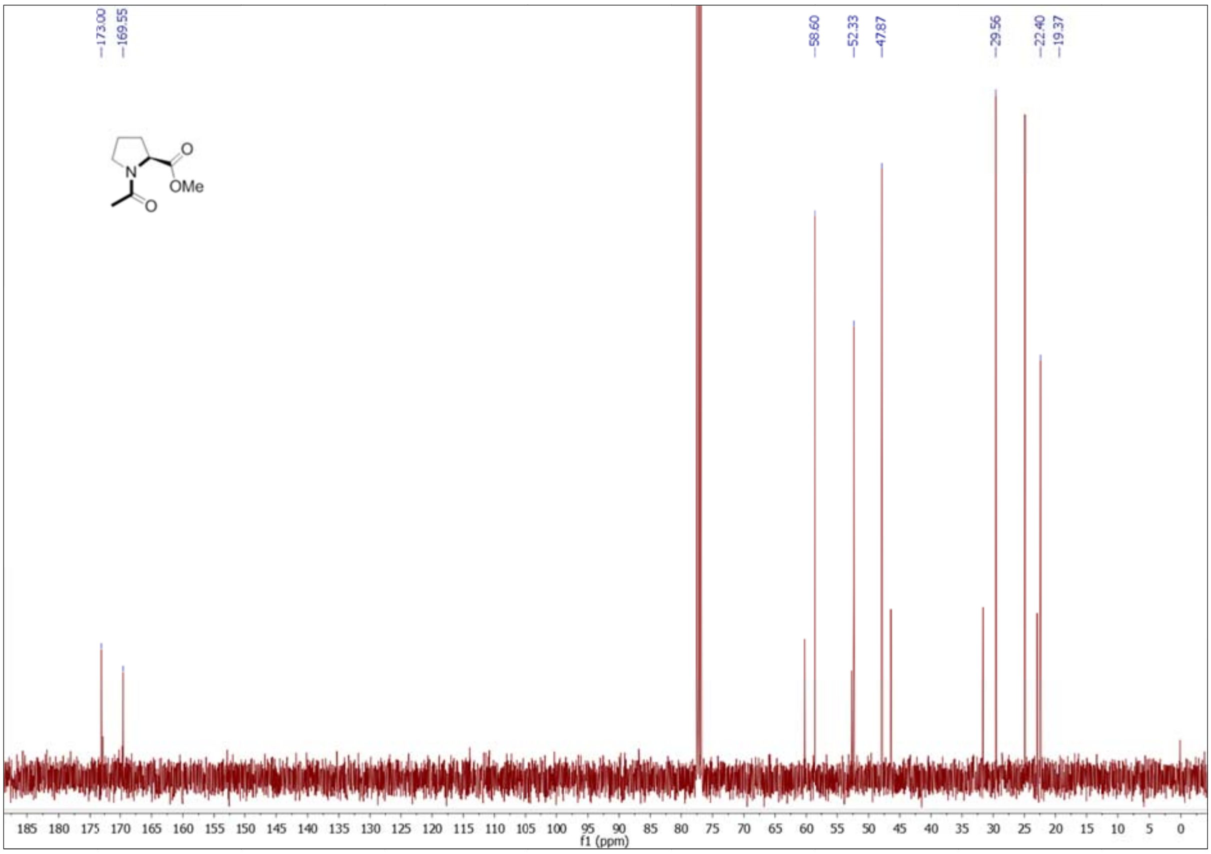

6.3. Ac-Pro-OMe (1-OMe) 80mM in DMSO-d6

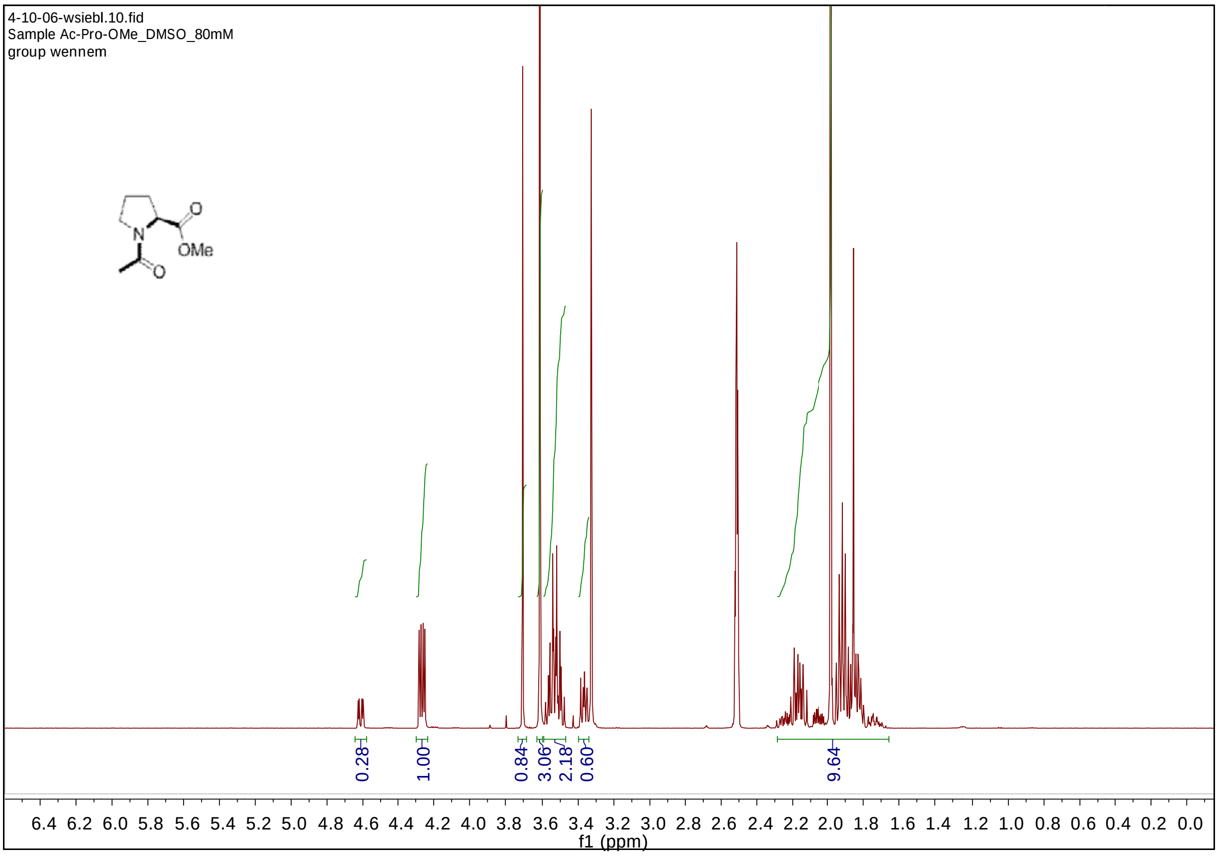

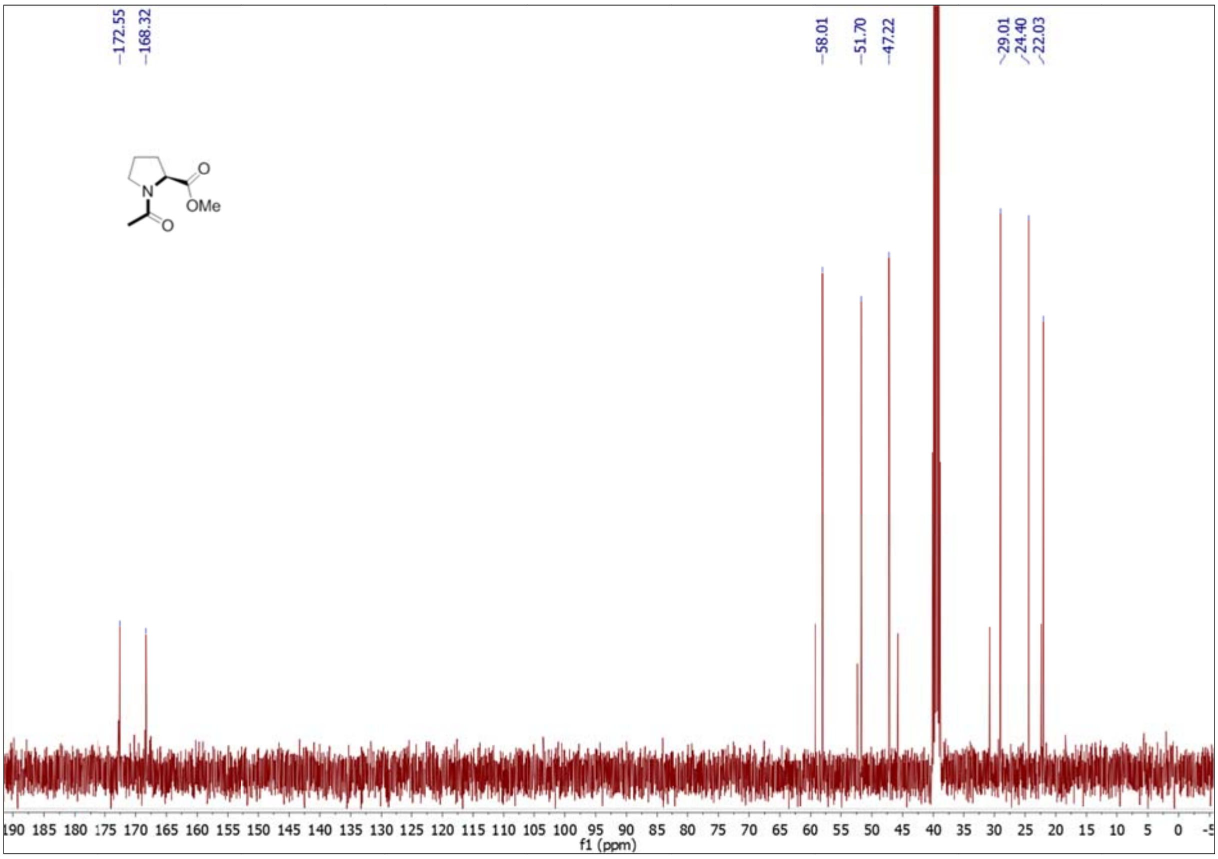

6.4. Ac-Pro-OMe (1-OMe) 80mM in dioxane-d8

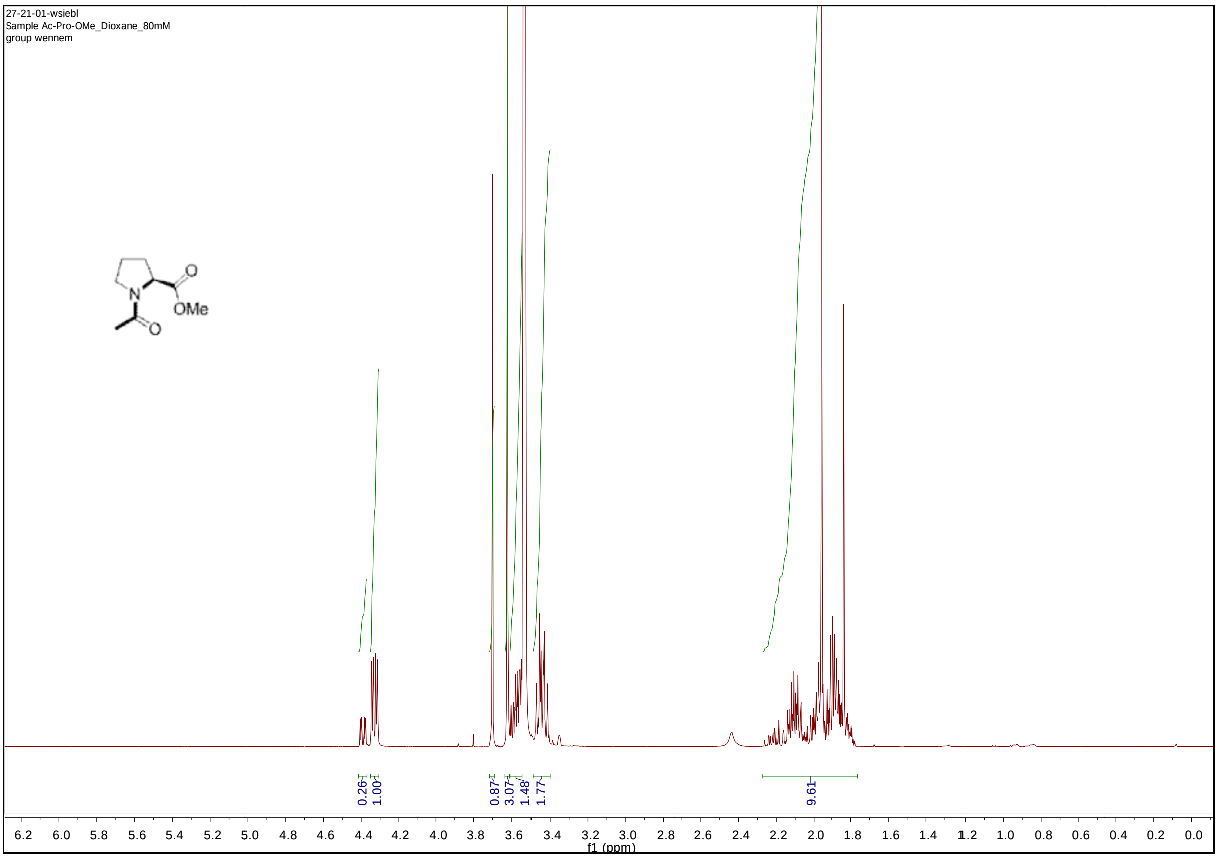

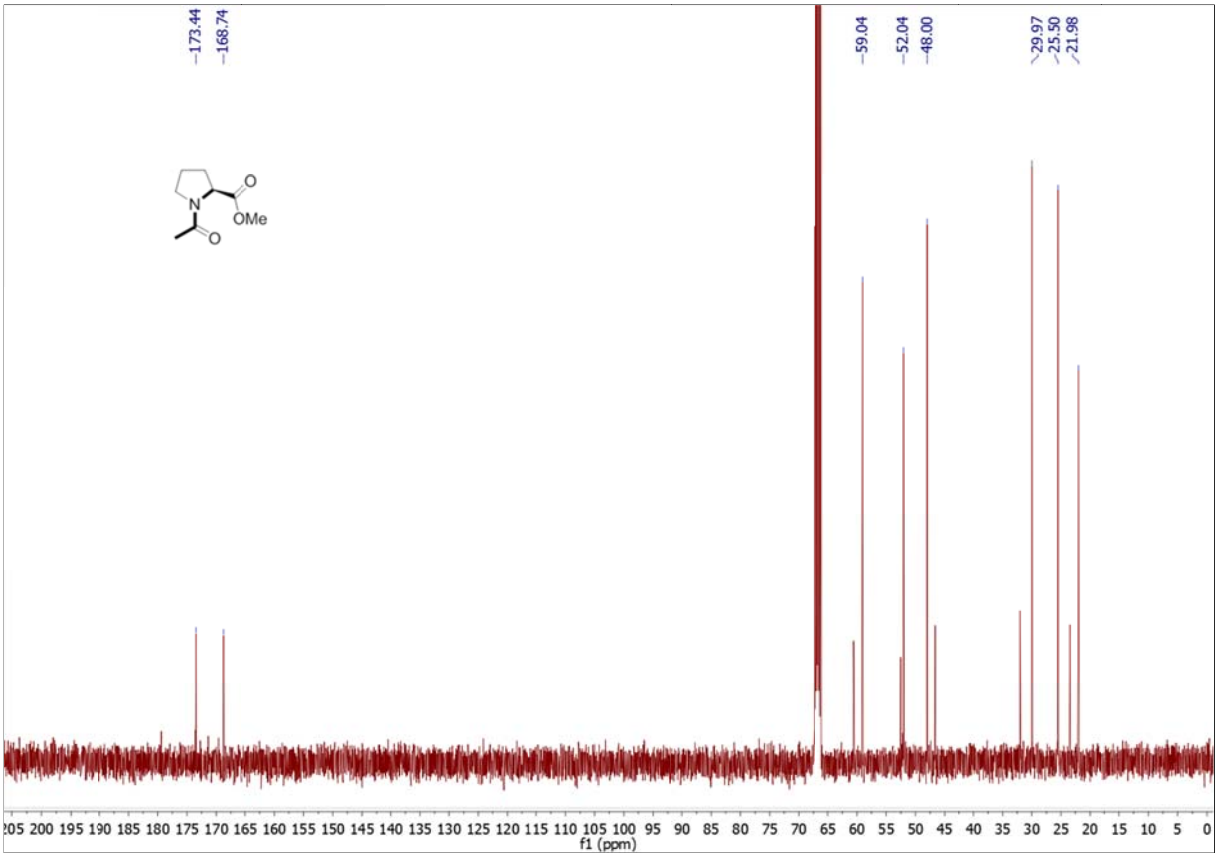

6.5. Ac-Pro-NMe$_2$ (1- NMe$_2$) 80mM in D_2O

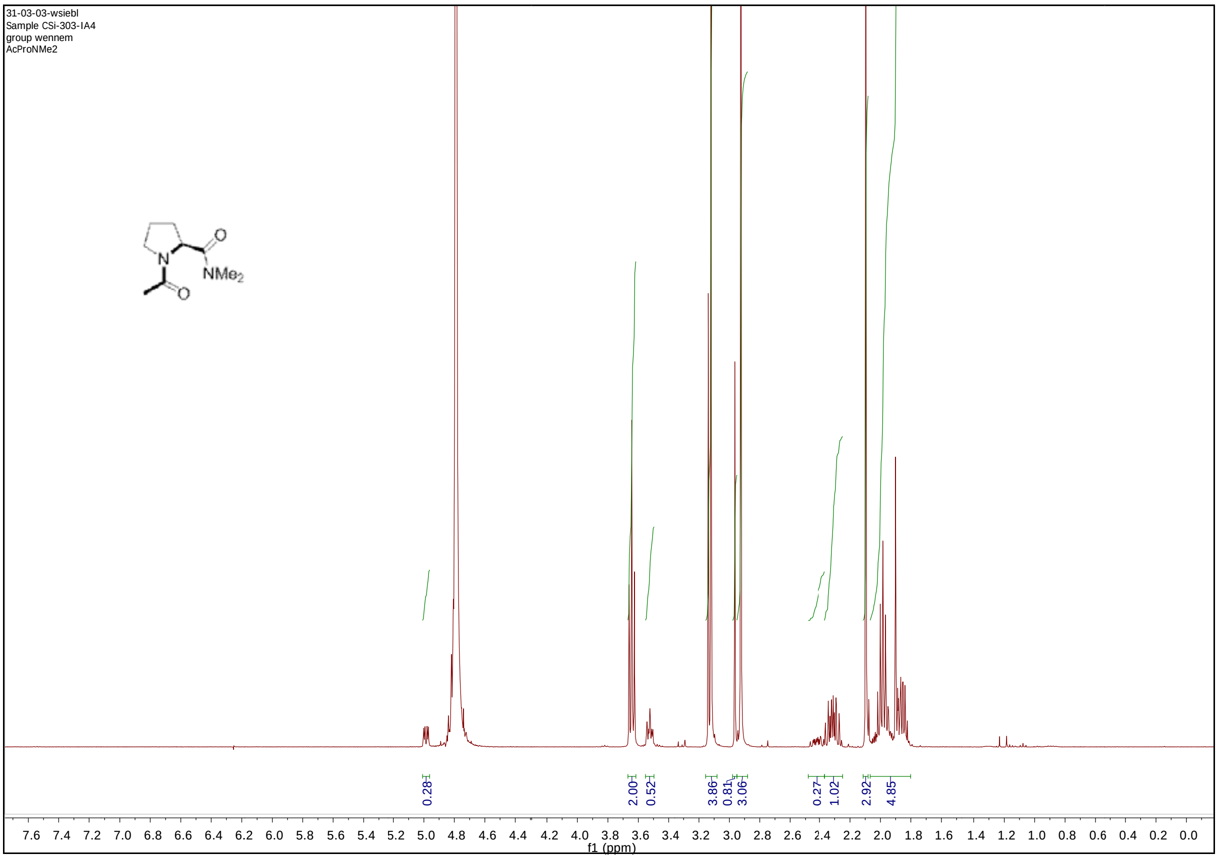

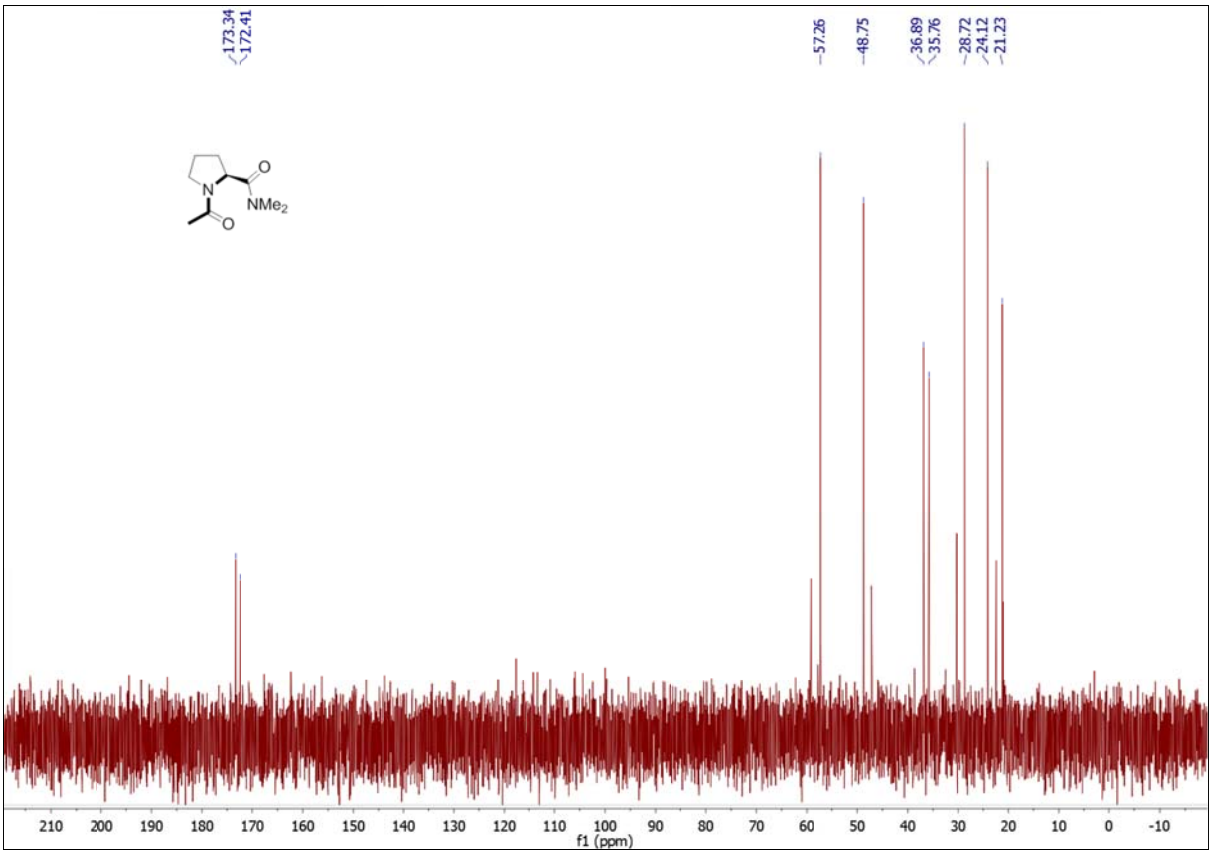

6.6. Ac-Pro-NMe$_2$ (1- NMe$_2$) 80mM in CDCl$_3$

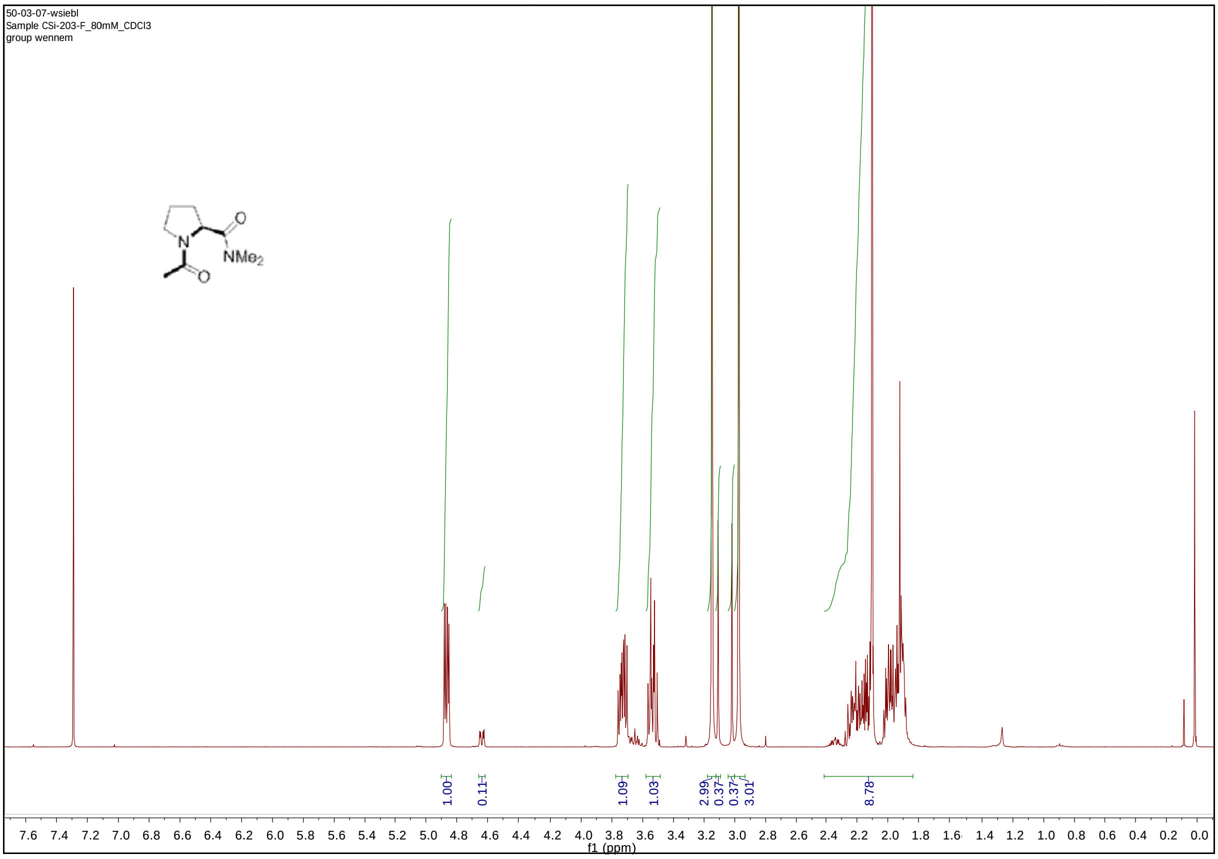

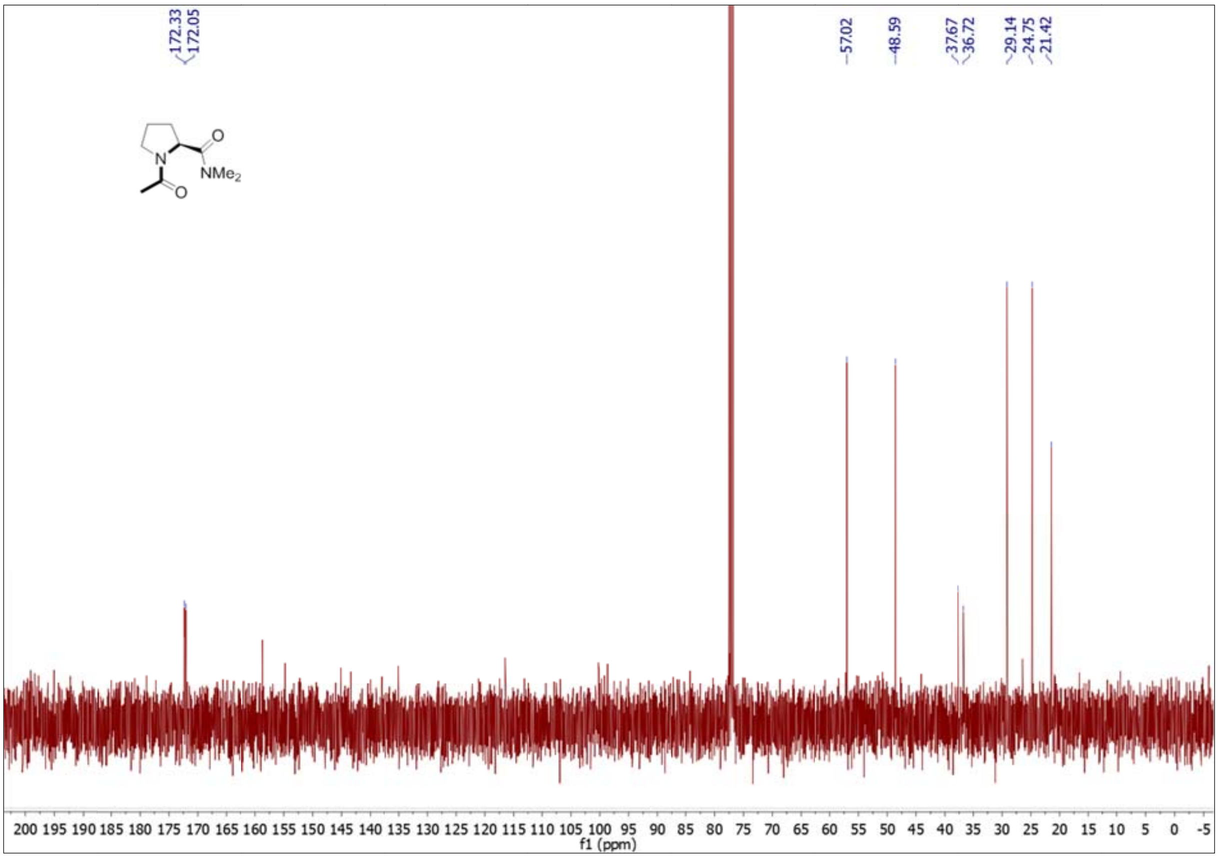

6.7. Ac-Pro-NMe$_2$ (1- NMe$_2$) 80mM in DMSO-d6

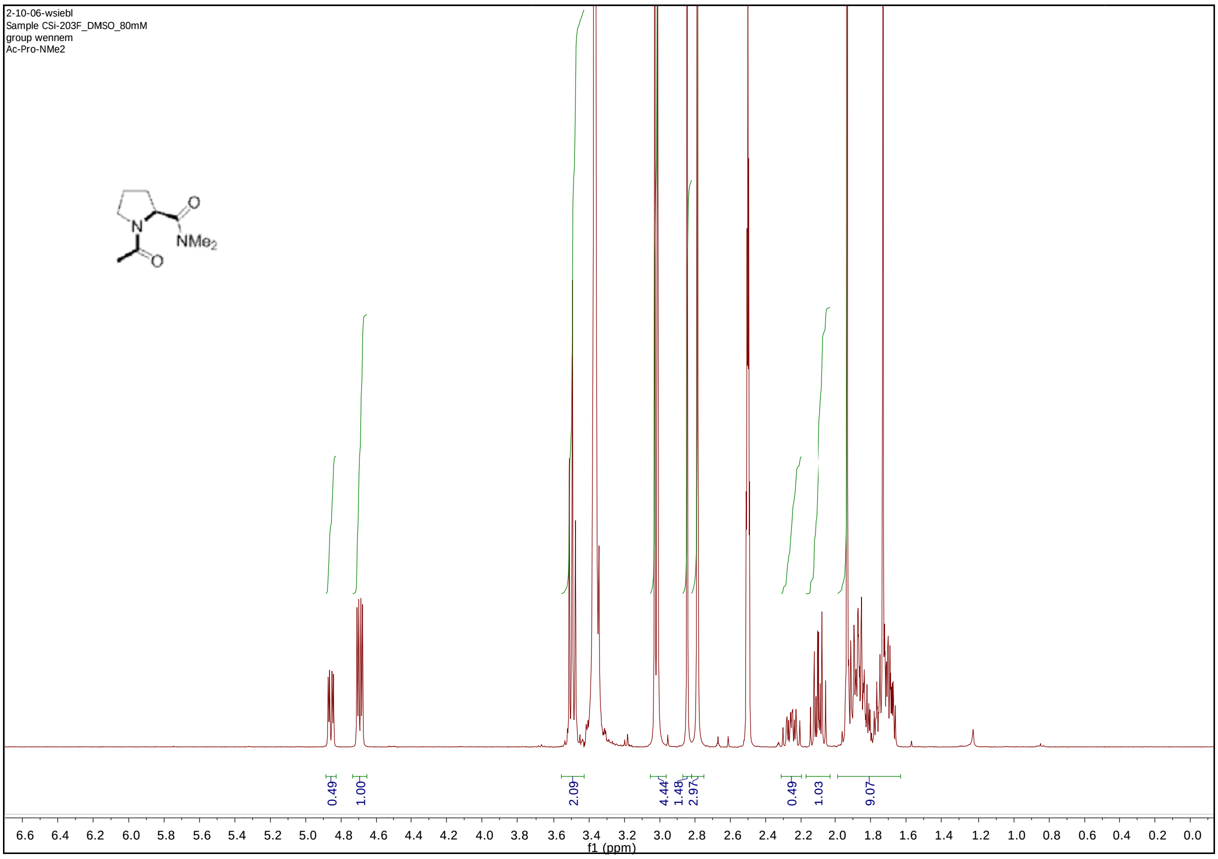

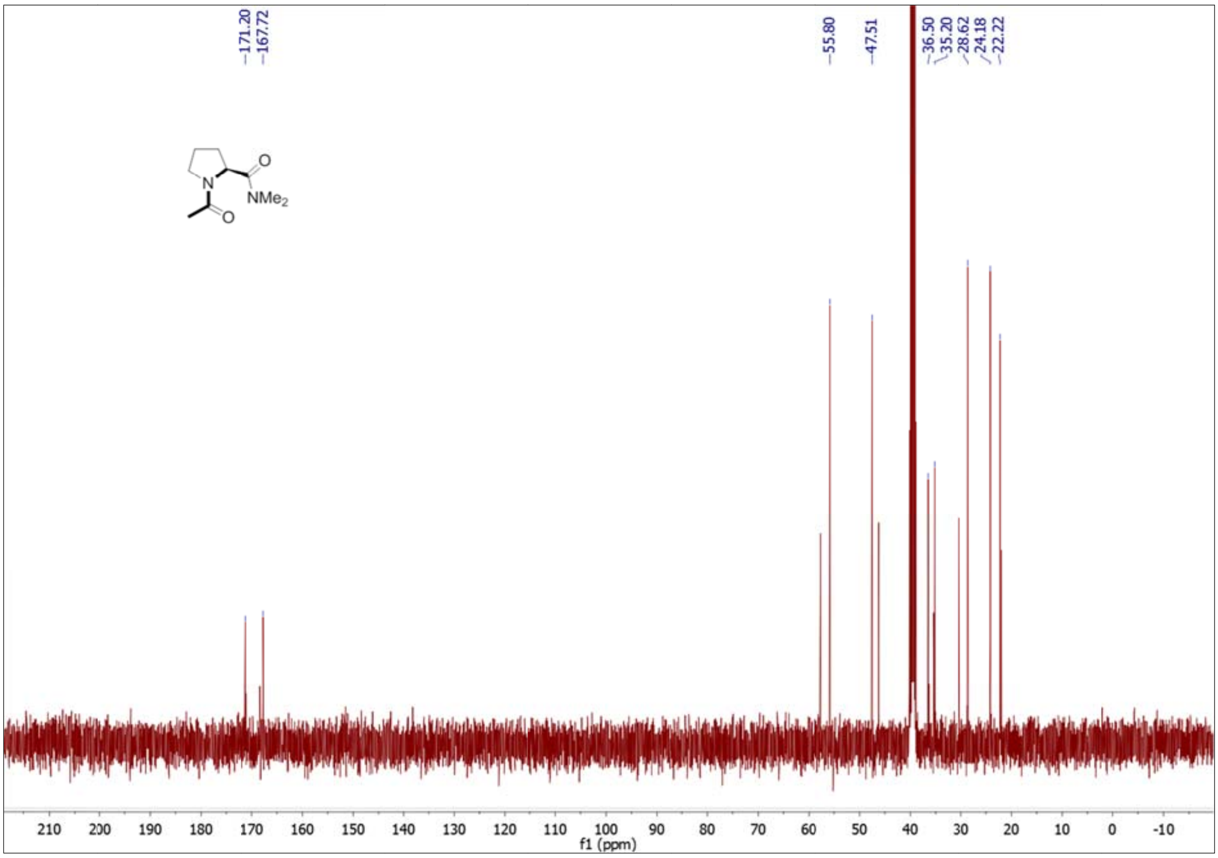

6.8. Ac-Pro-NMe$_2$ (1- NMe$_2$) 80mM in dioxane-d8

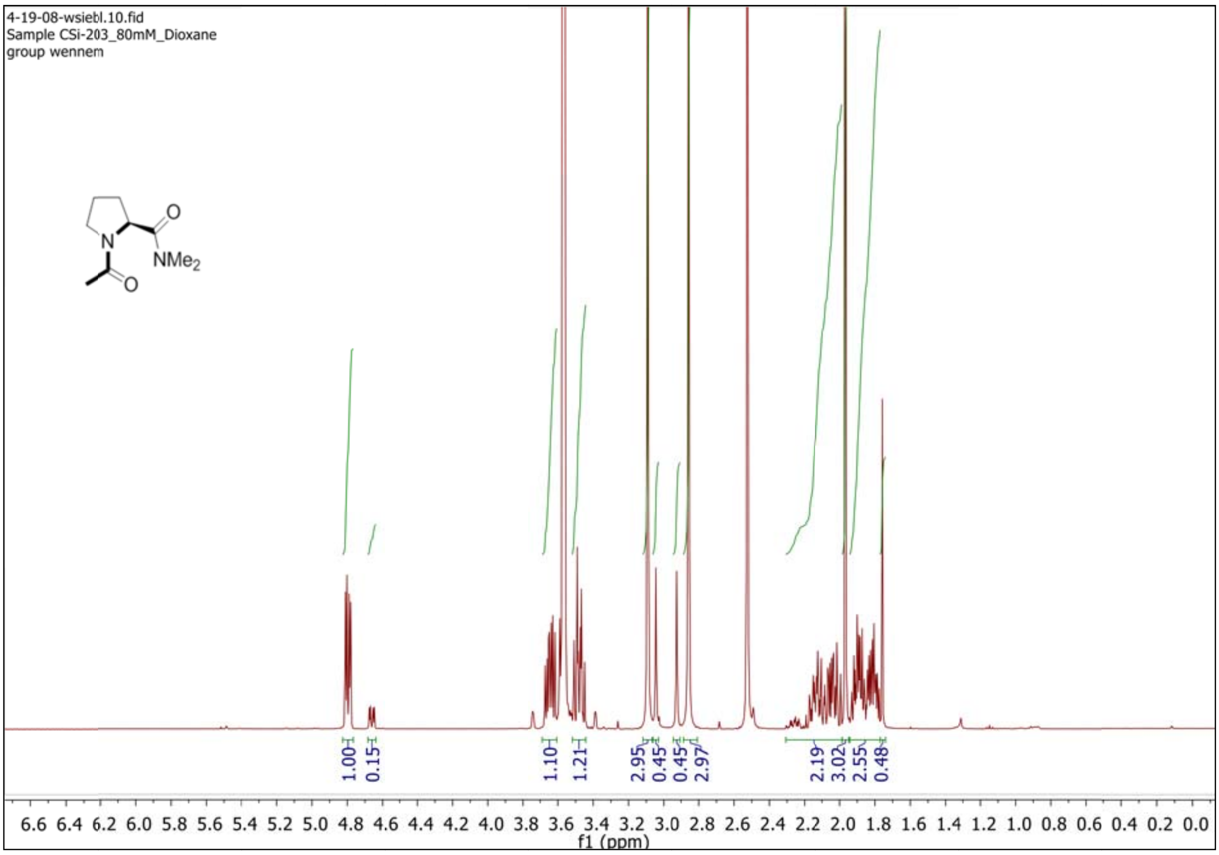

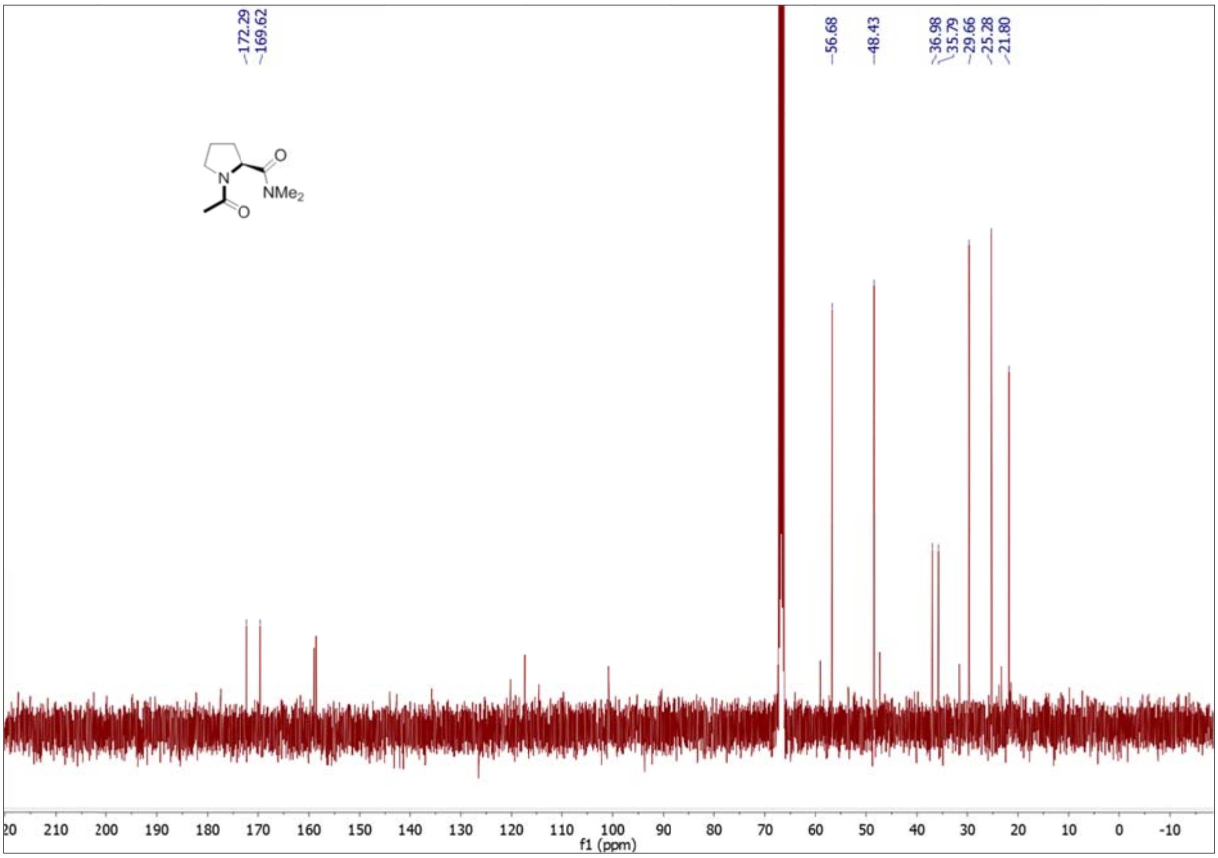

6.9. Ac-(4*R*)Flp-OMe (2R-OMe) 80mM in D_2O

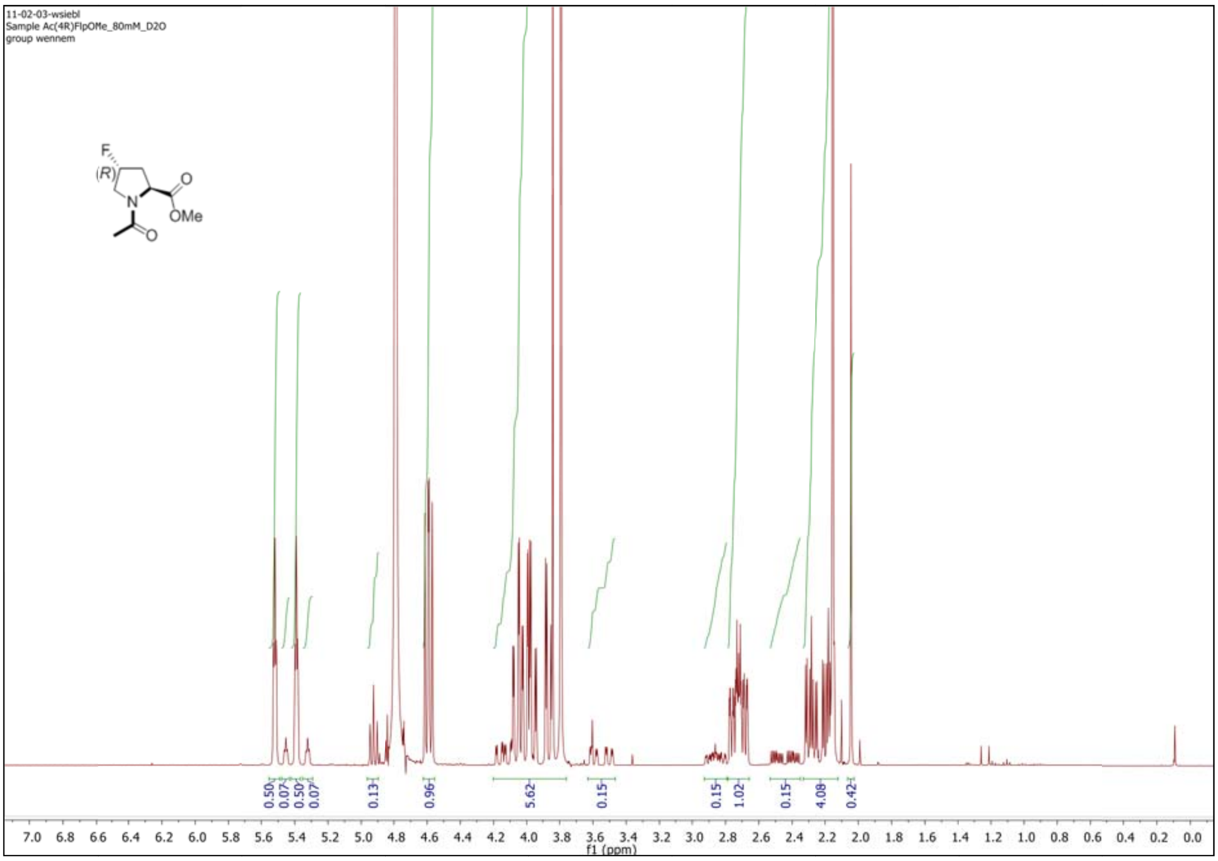

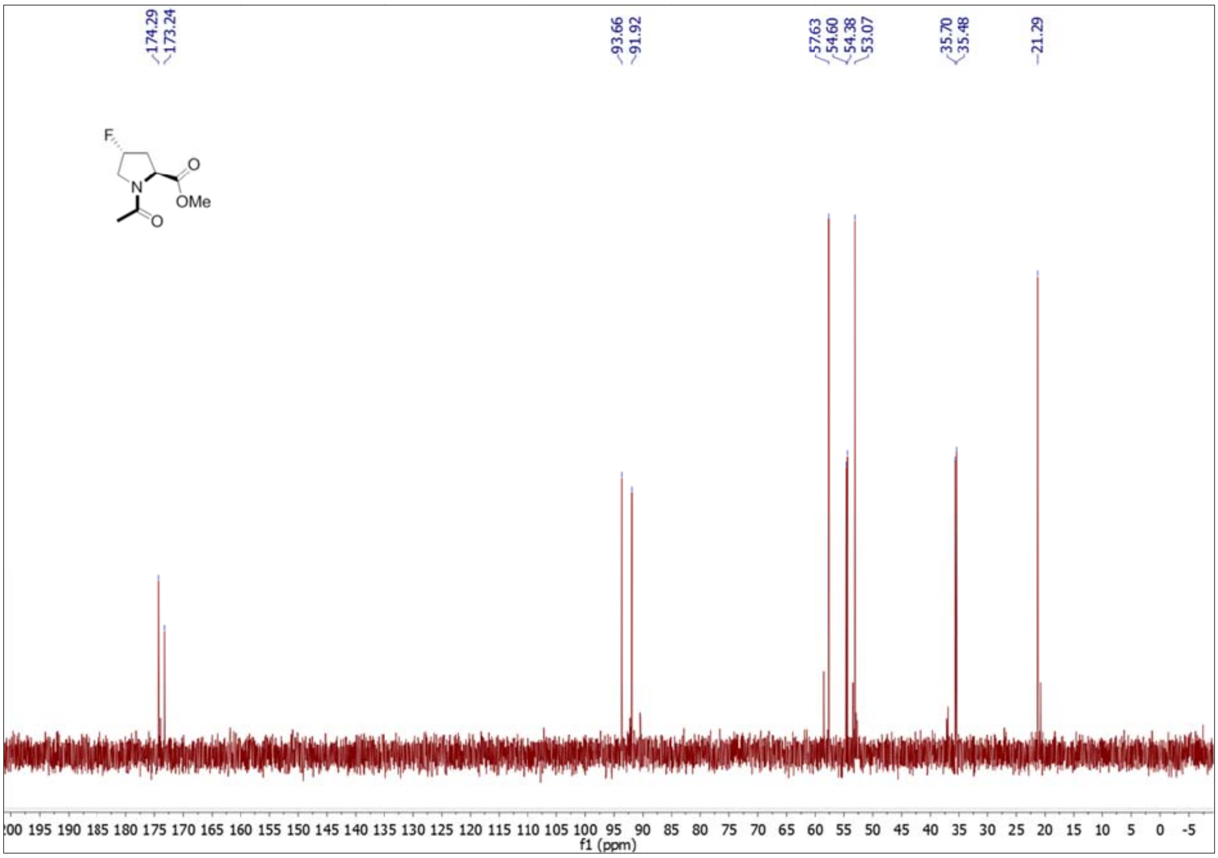

Ac-(4*R*)Flp-OMe (2R-OMe) 80mM in $CDCl_3$

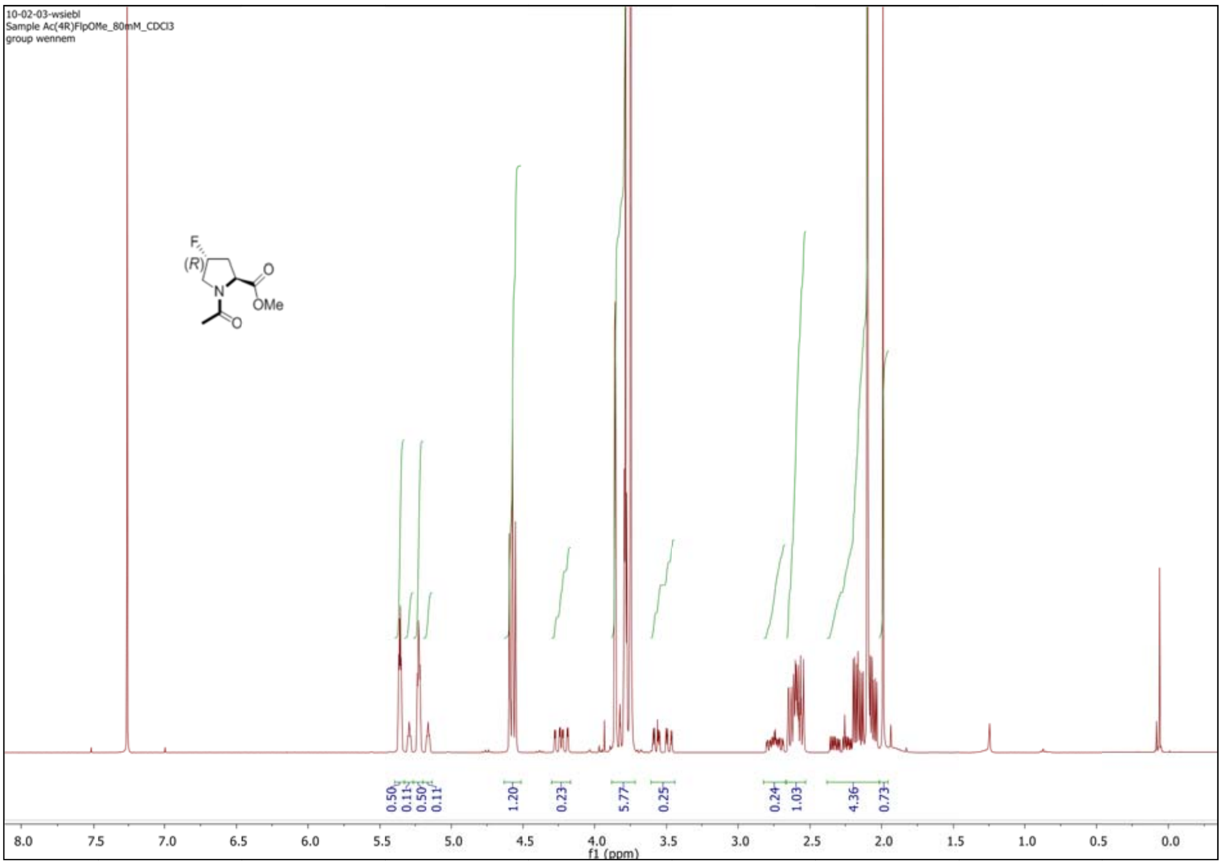

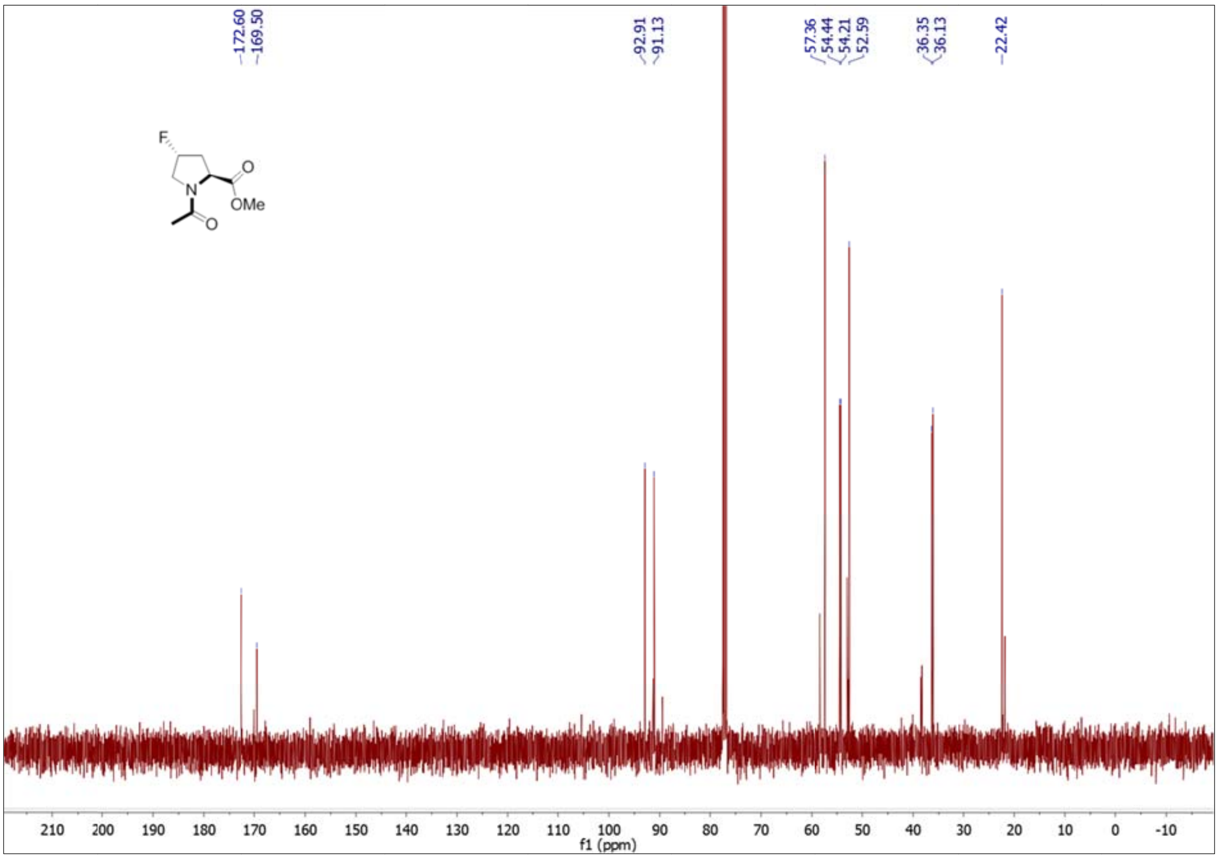

6.10. Ac-(4*R*)Flp-NMe$_2$ (2R-NMe$_2$) 80mM in D_2O

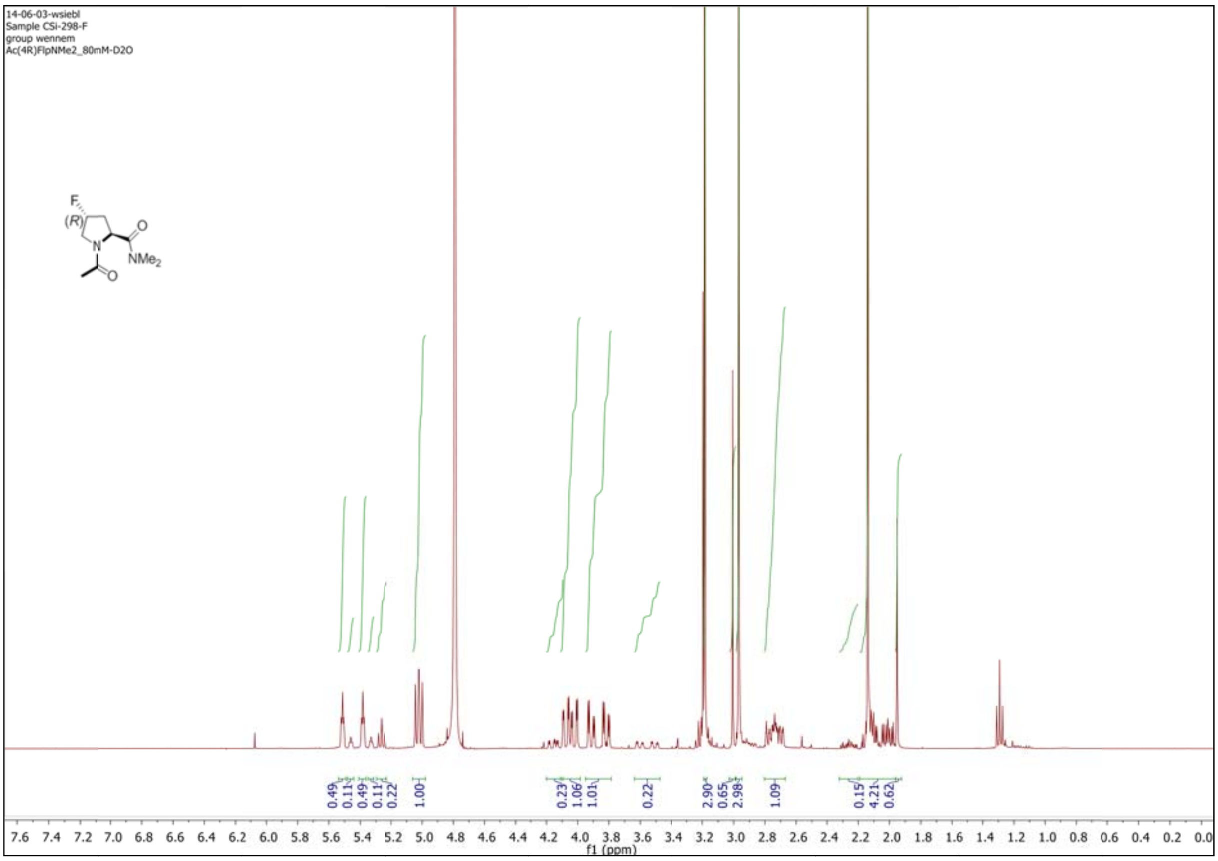

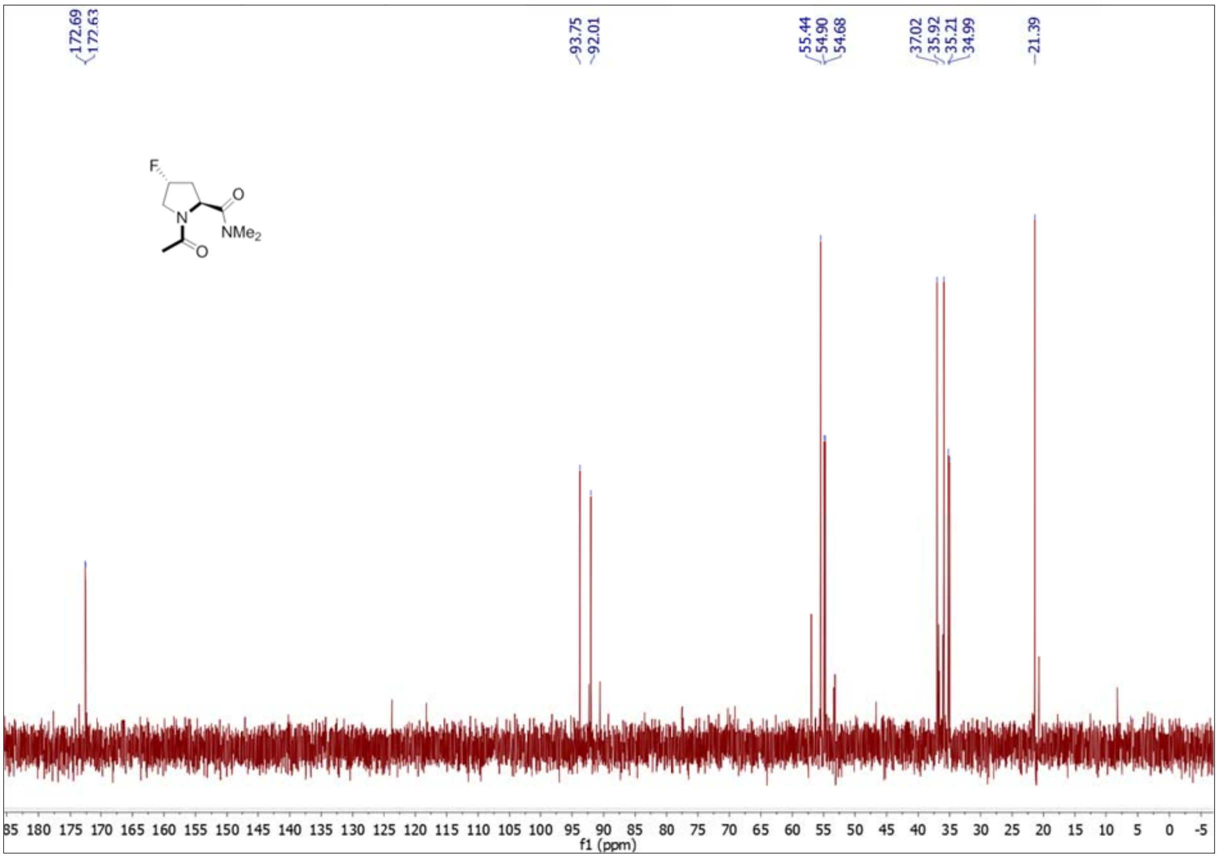

6.11. Ac-(4*R*)Flp-NMe_2 (2R-NMe_2) 80mM in $CDCl_3$

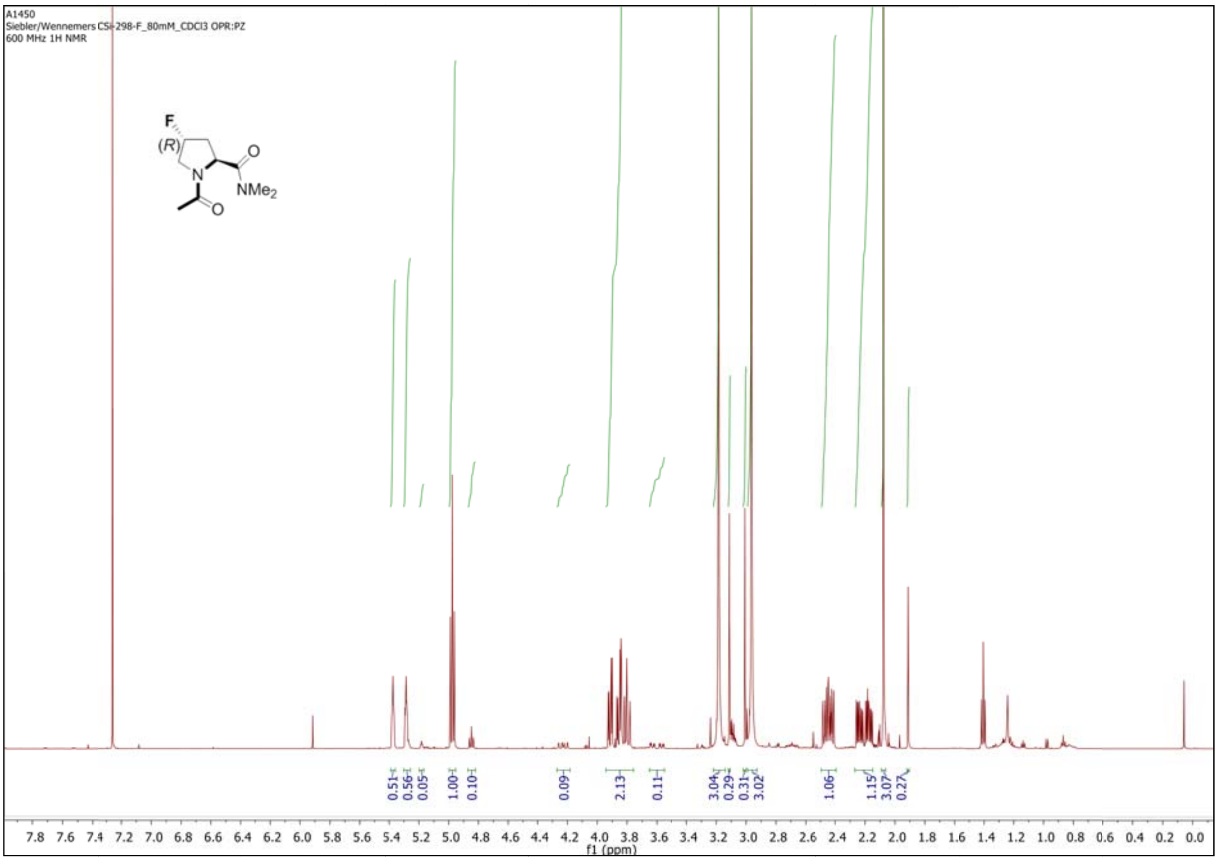

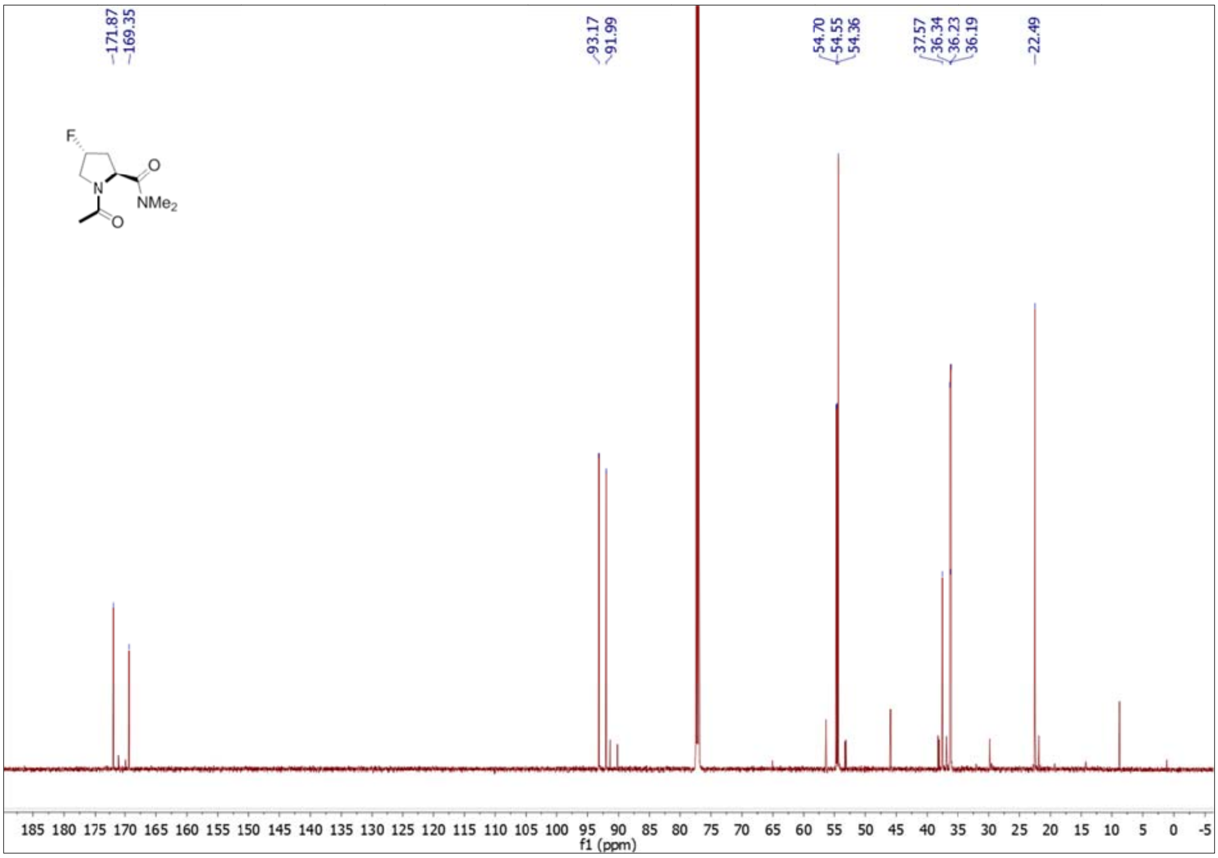

6.12. Ac-(4*R*)Azp-NMe$_2$ (3R-NMe$_2$) 80mM in D$_2$O

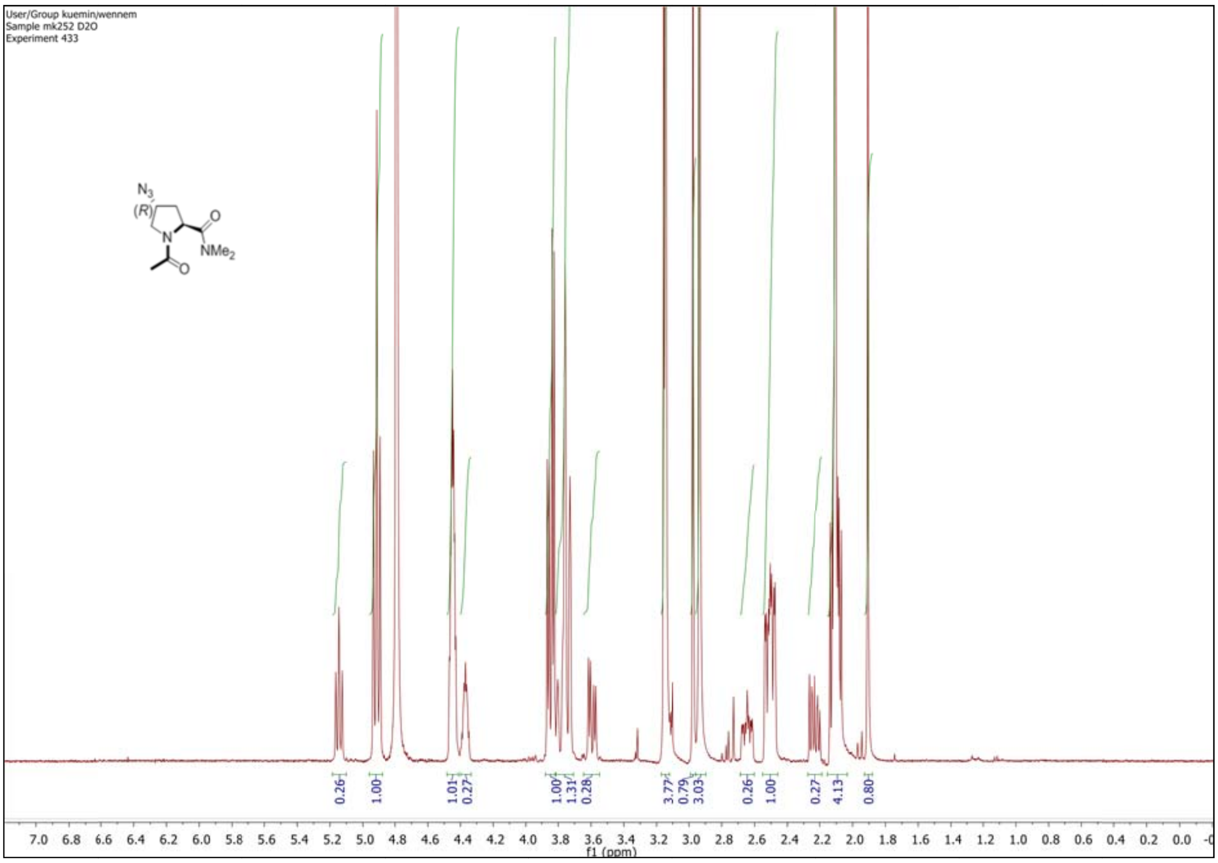

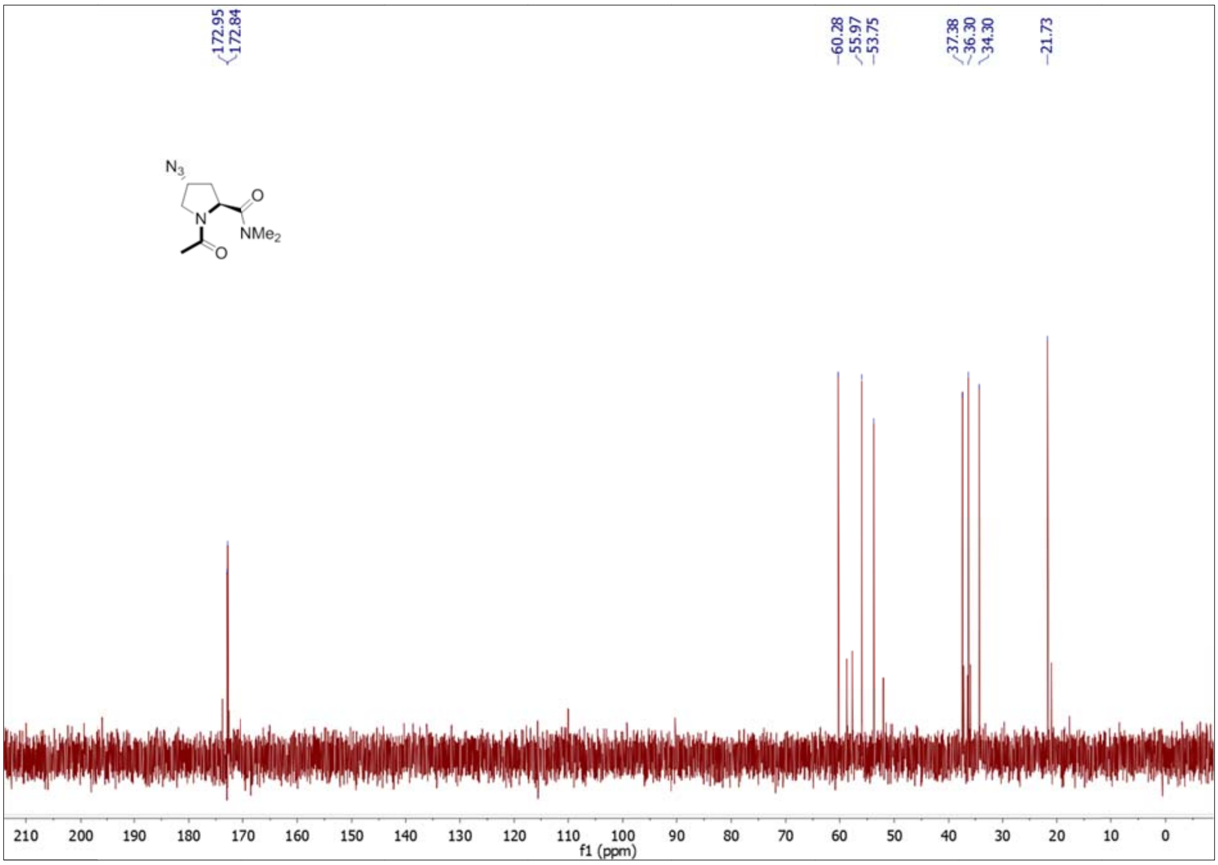

6.13. Ac-(4*R*)Azp-NMe$_2$ (3R-NMe$_2$) 80mM in CDCl$_3$

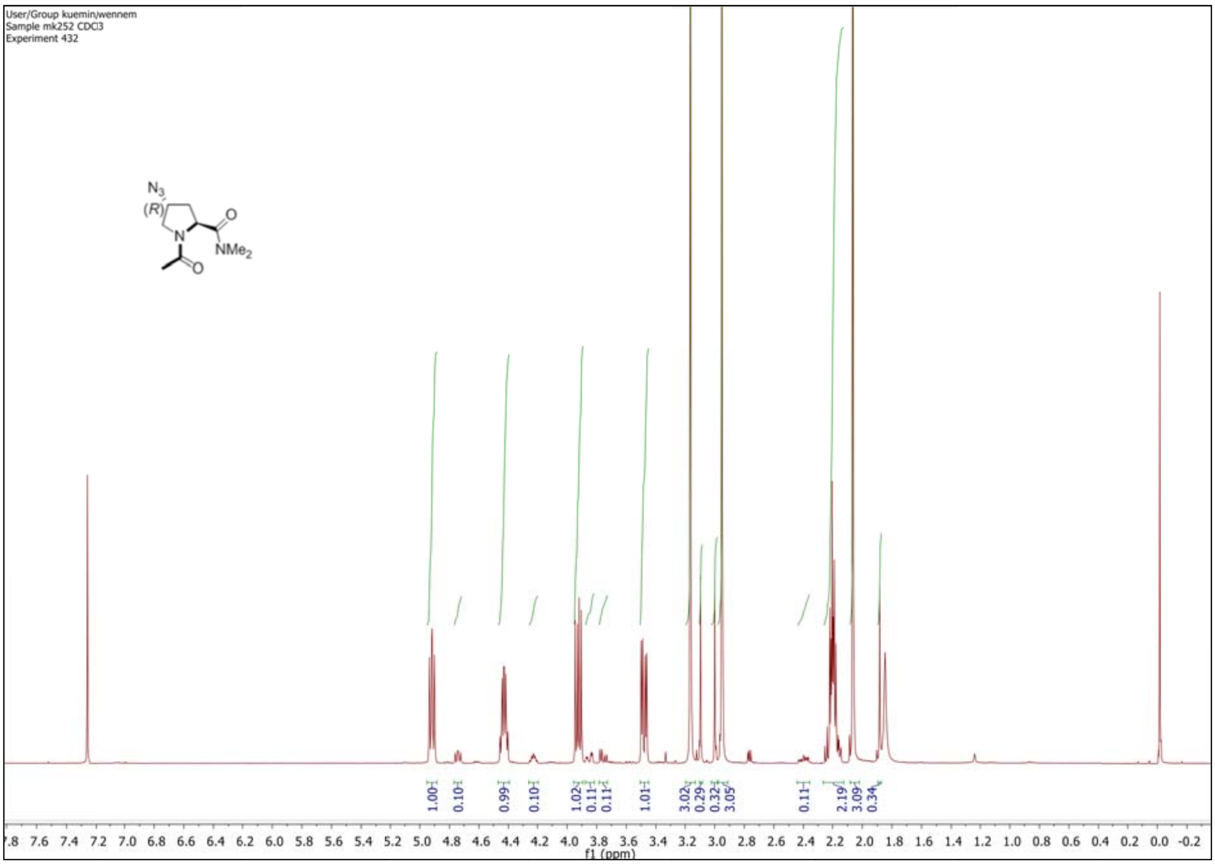

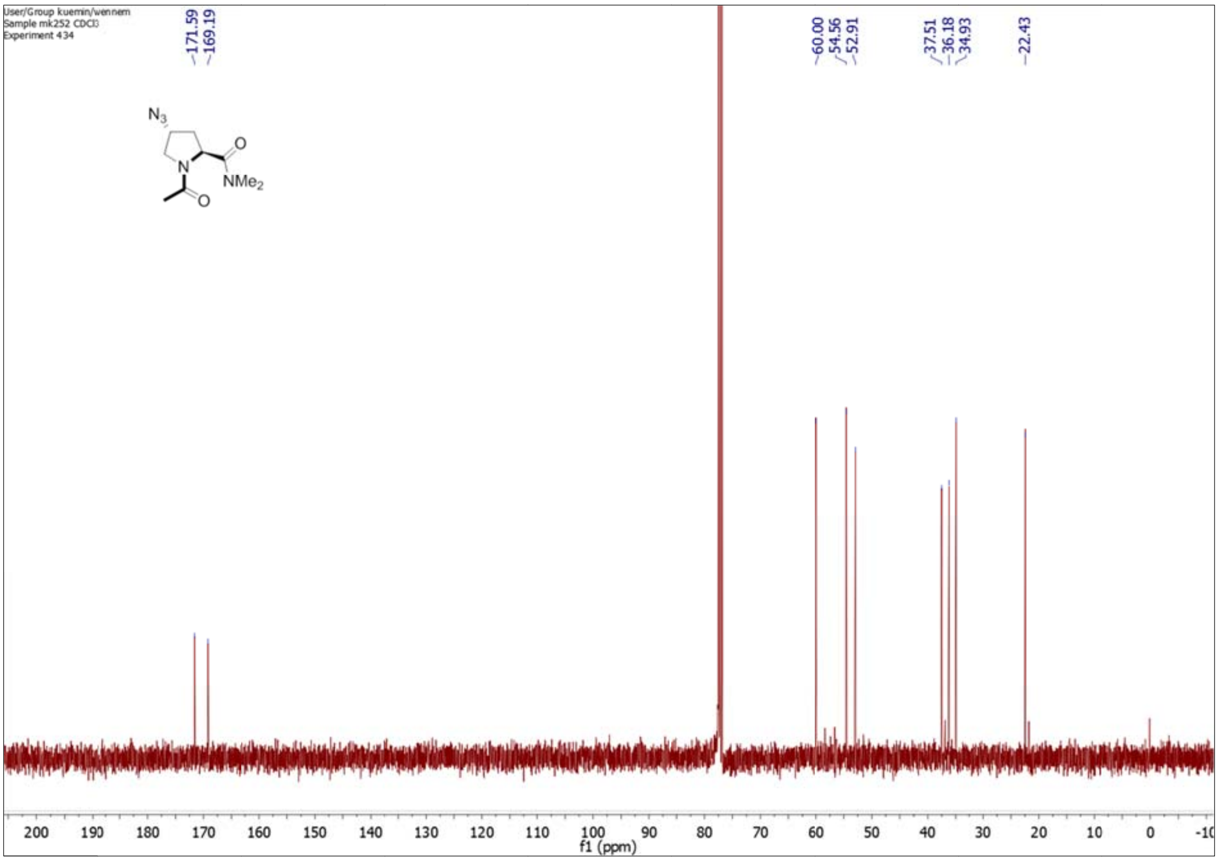

6.14. Ac-(4*S*)Flp-OMe (2S-OMe) 80mM in D_2O

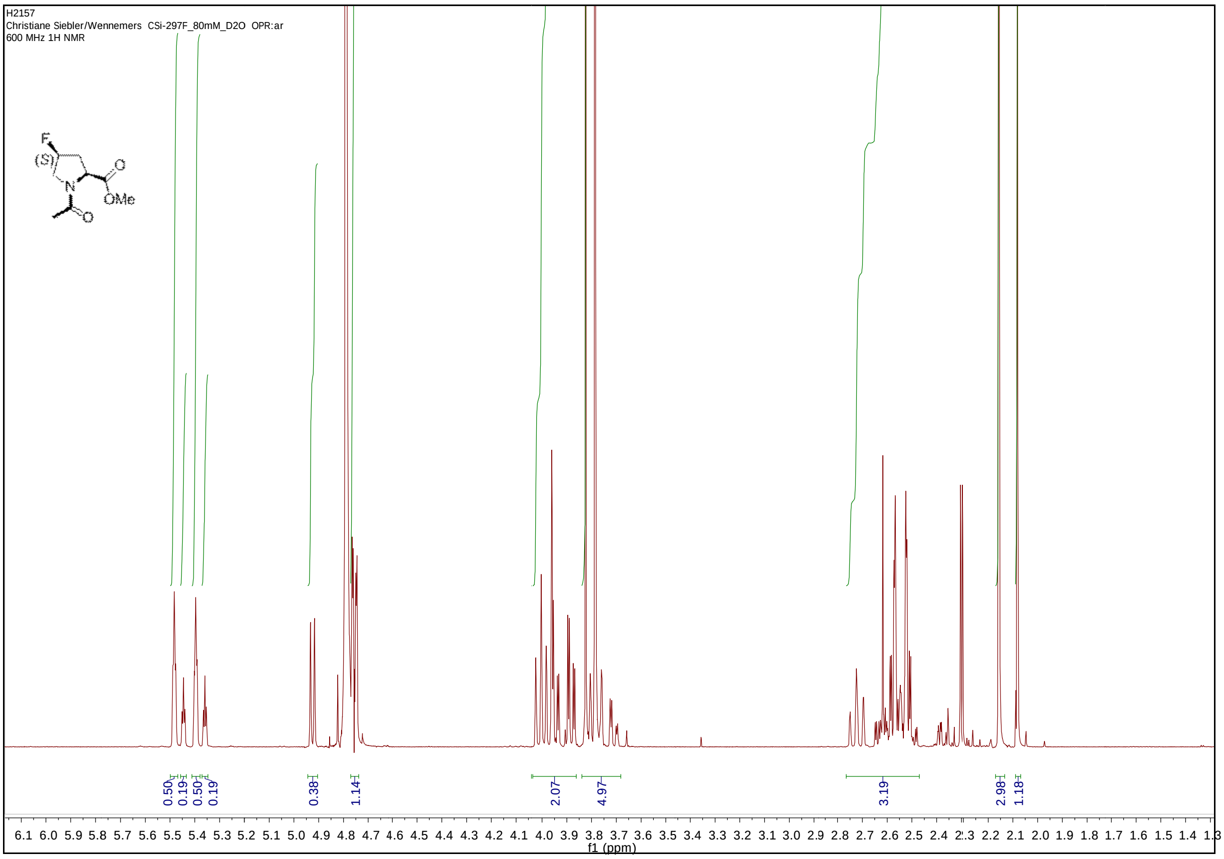

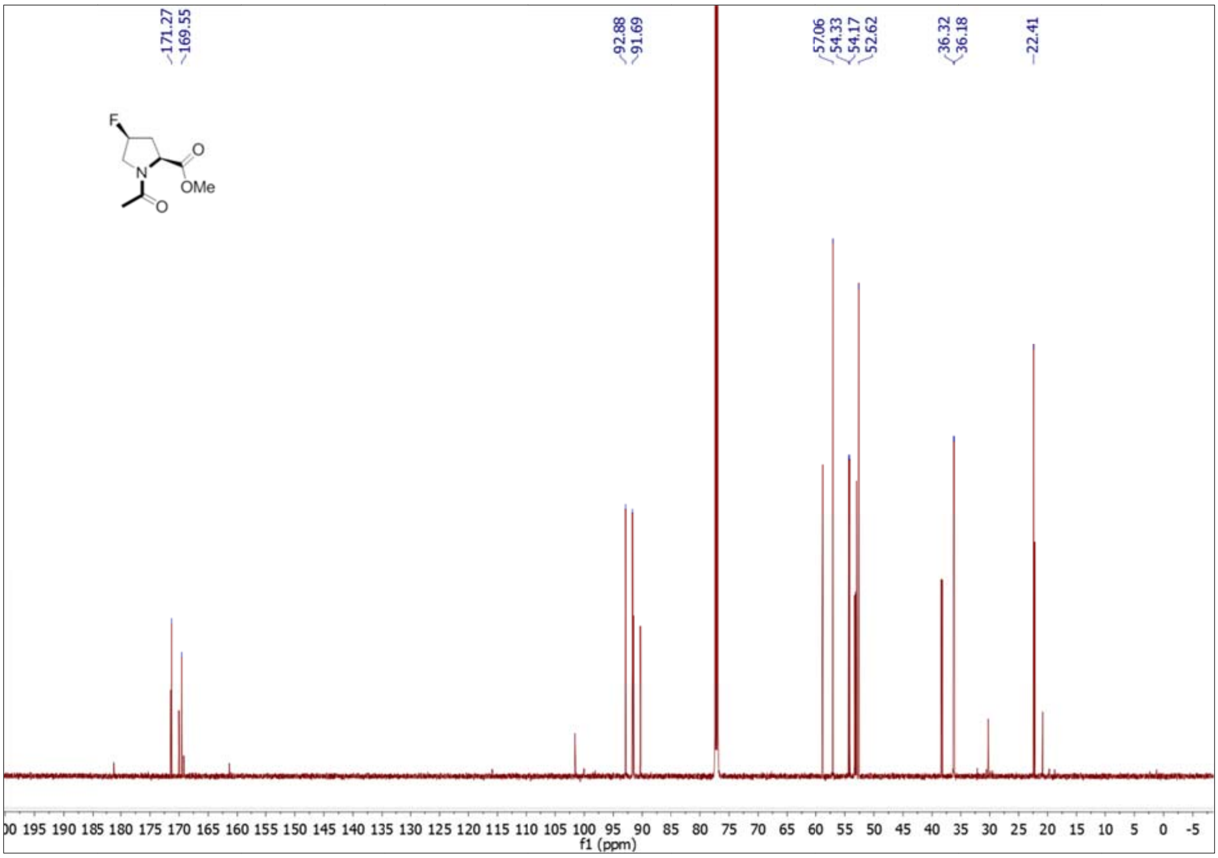

6.15. Ac-(4*S*)Flp-OMe (2S-OMe) 80mM in $CDCl_3$

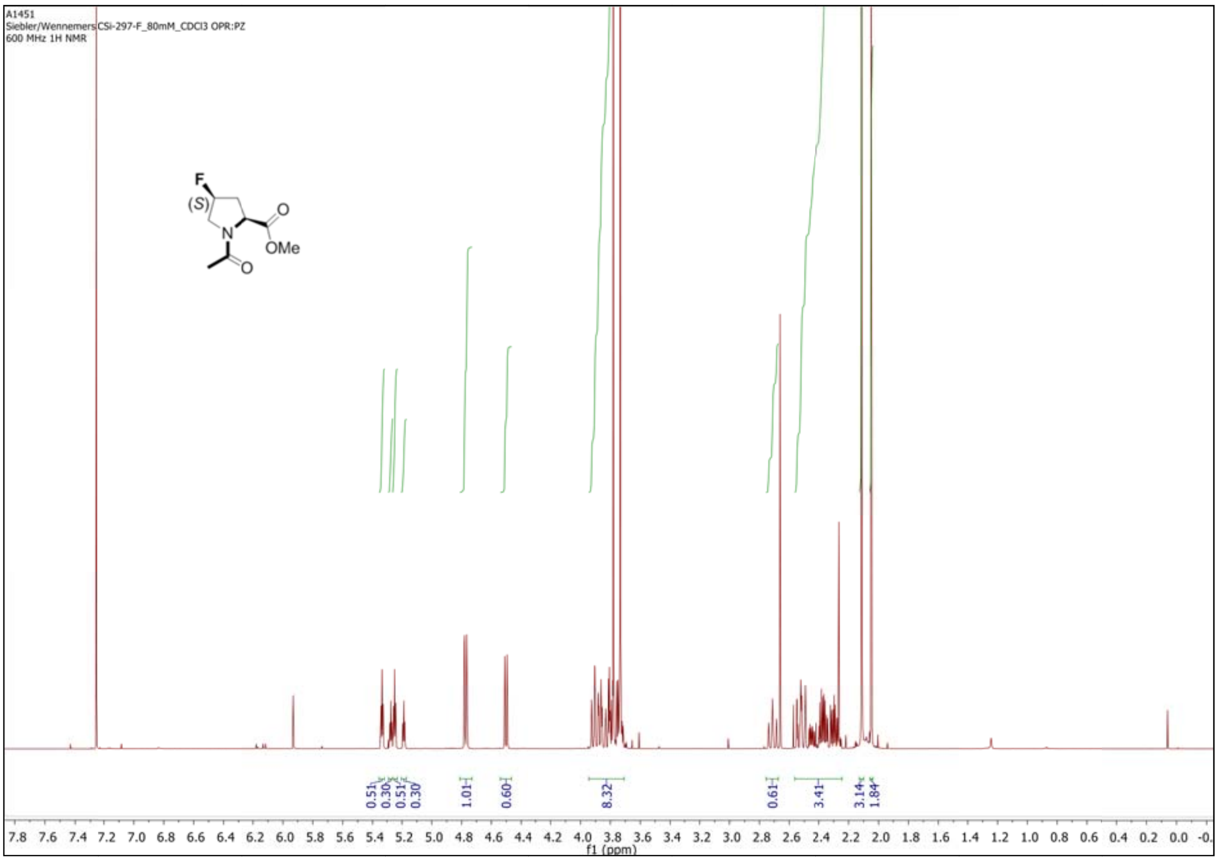

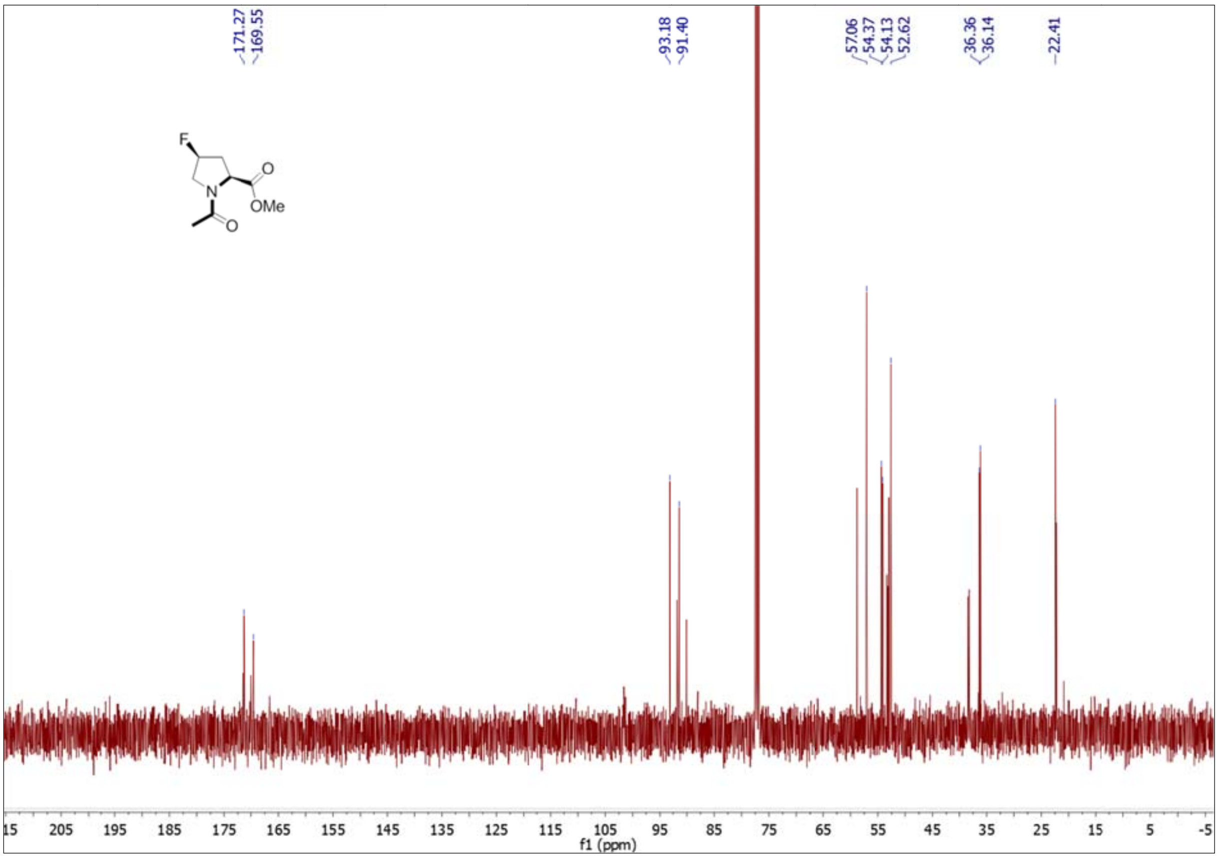

6.16. Ac-(4*S*)Flp-NMe_2 (2S-NMe_2) 80mM in D_2O

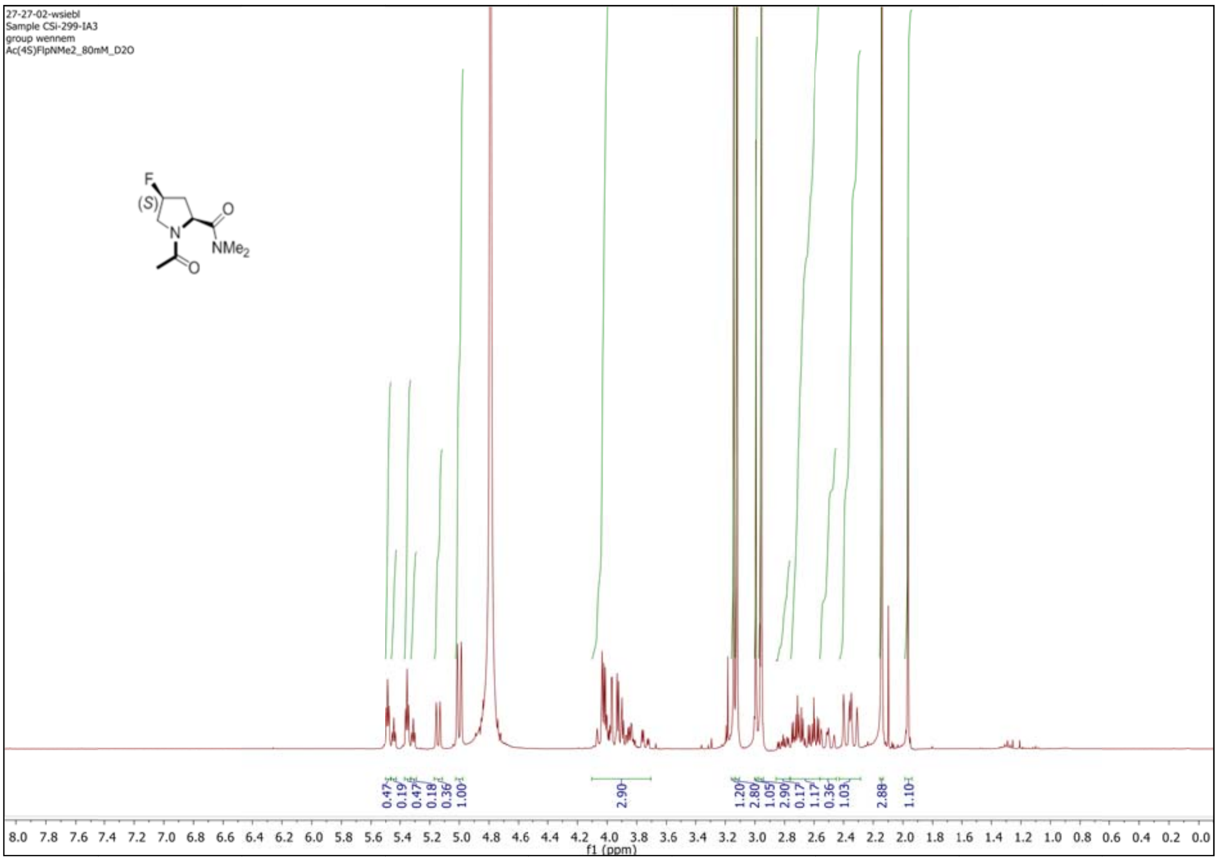

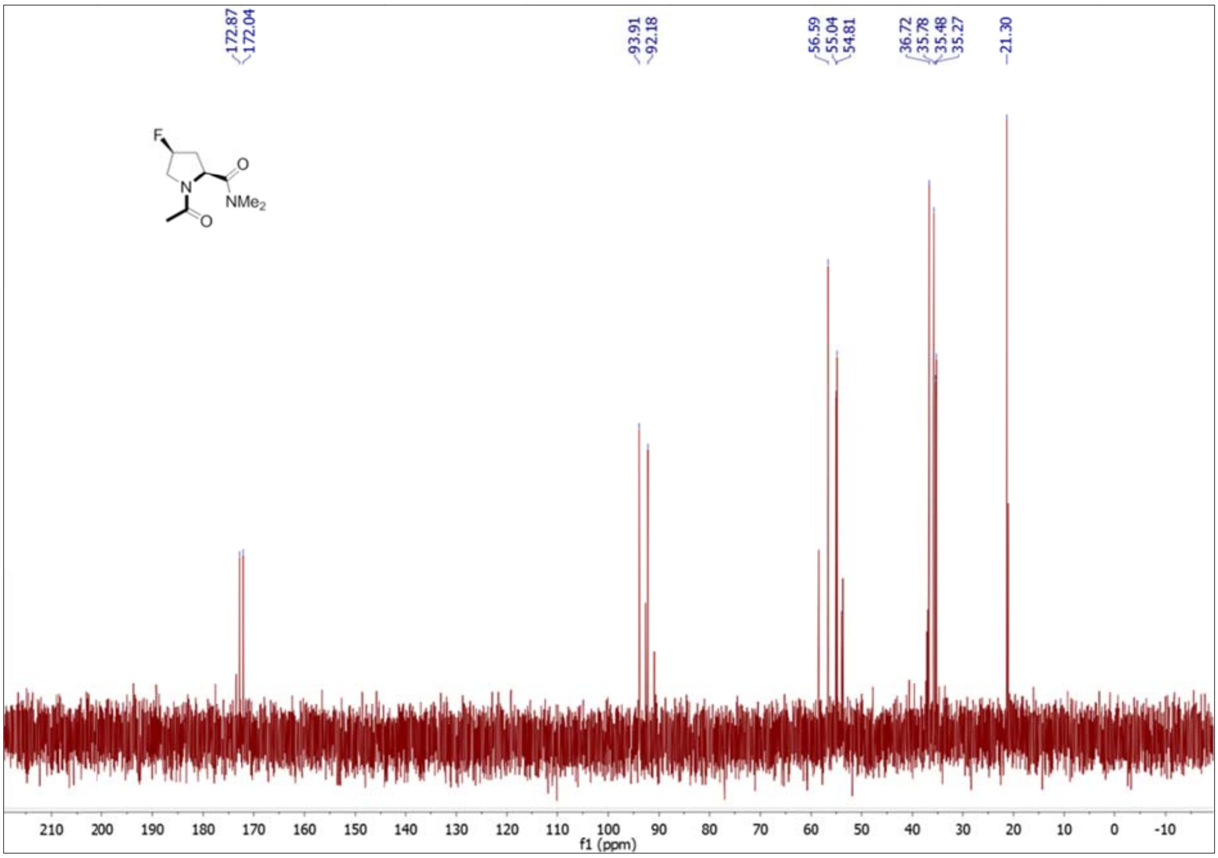

6.17. Ac-(4*S*)Flp-NMe$_2$ (2S-NMe$_2$) 80mM in CDCl$_3$

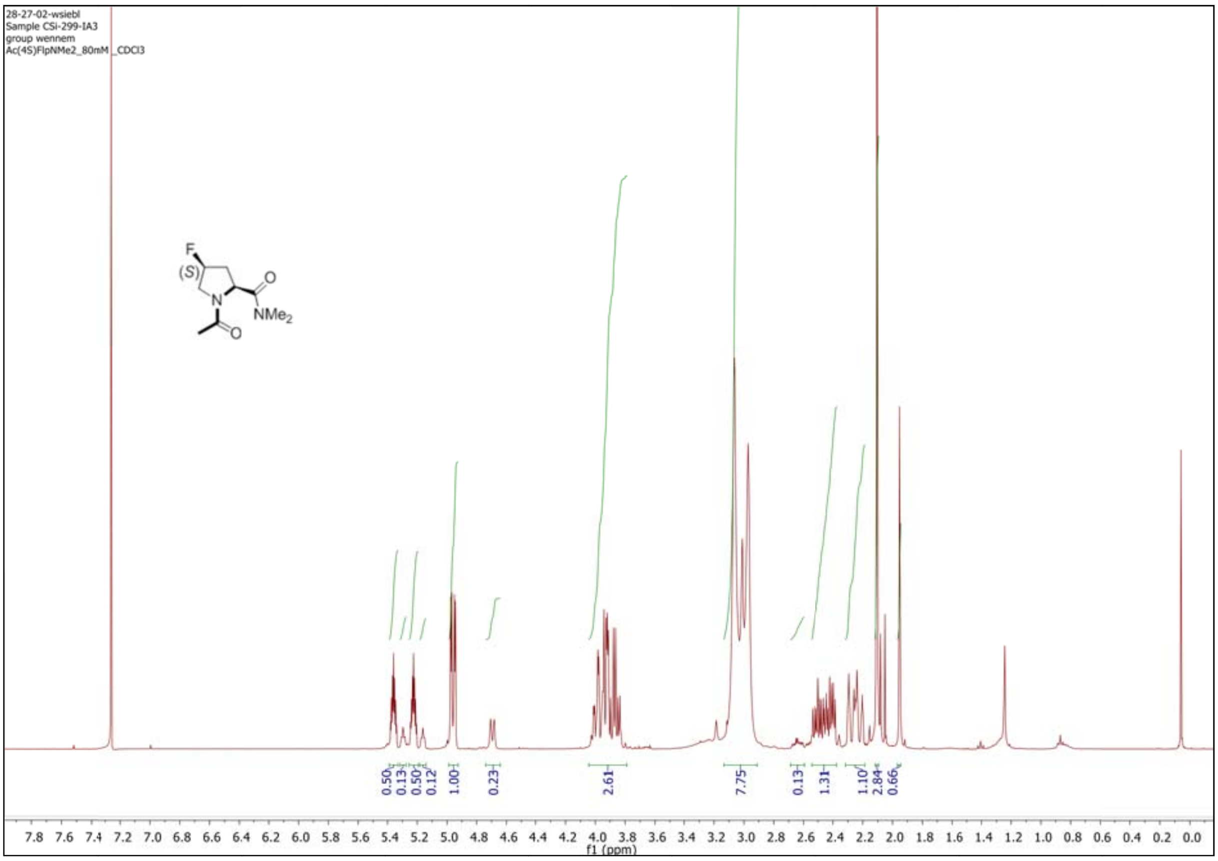

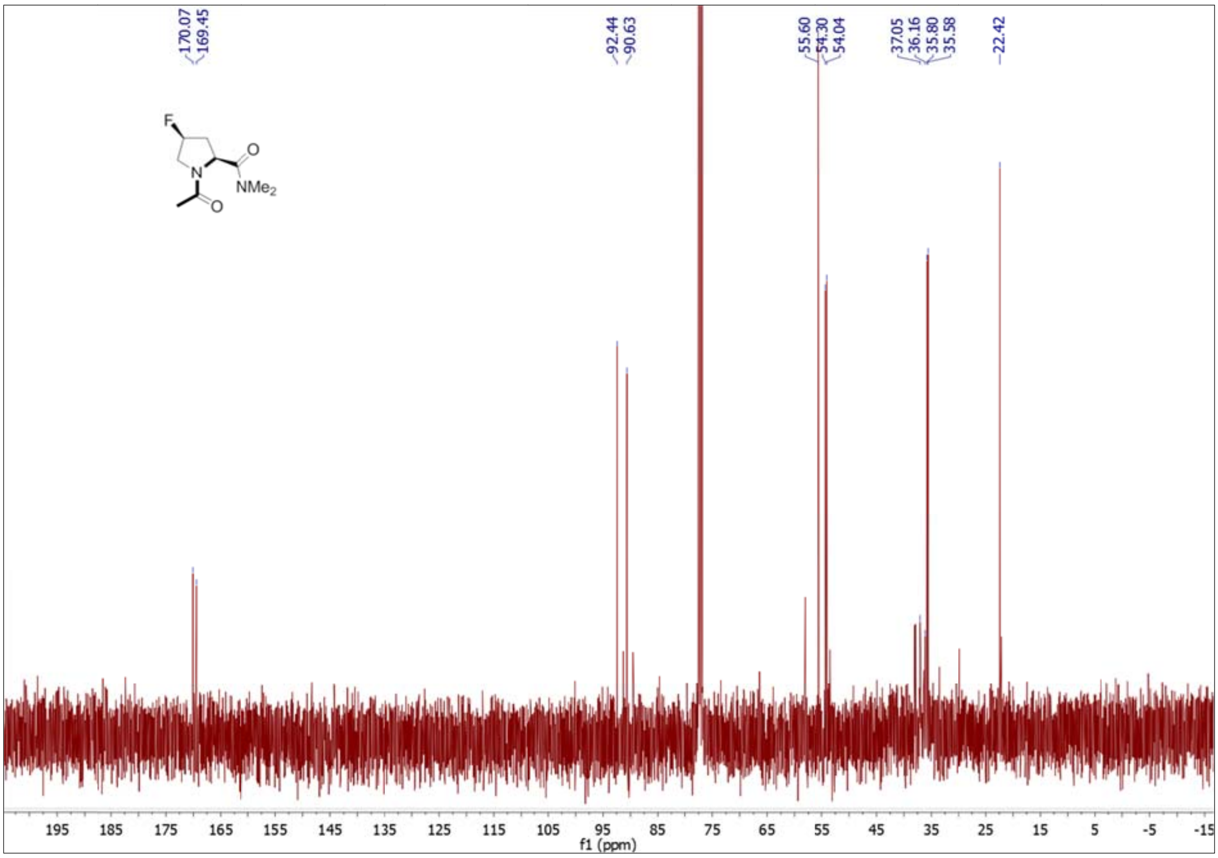

6.18. Ac-(4*S*)Azp-NMe$_2$ (3S-NMe$_2$) 80mM in D_2O

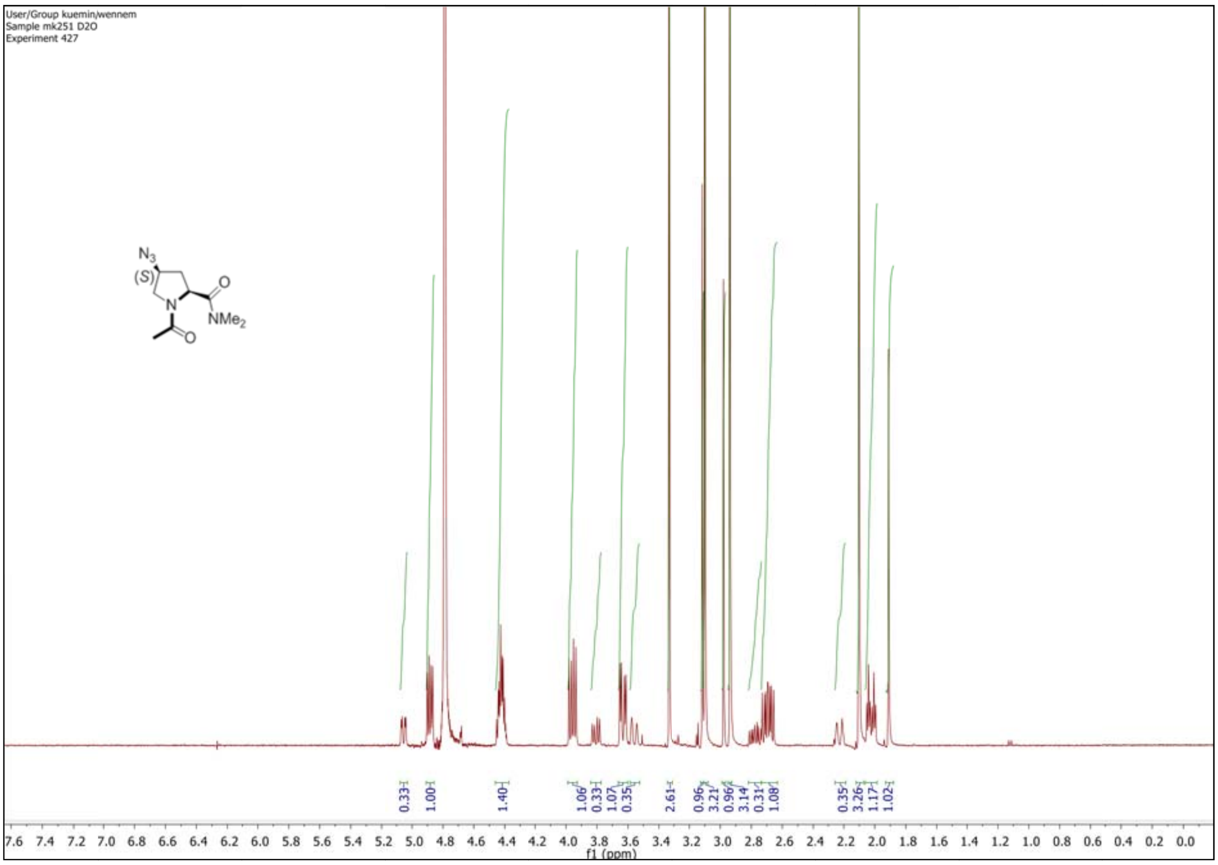

6.19. Ac-(4*S*)Azp-NMe$_2$ (3S-NMe$_2$) 80mM in $CDCl_3$

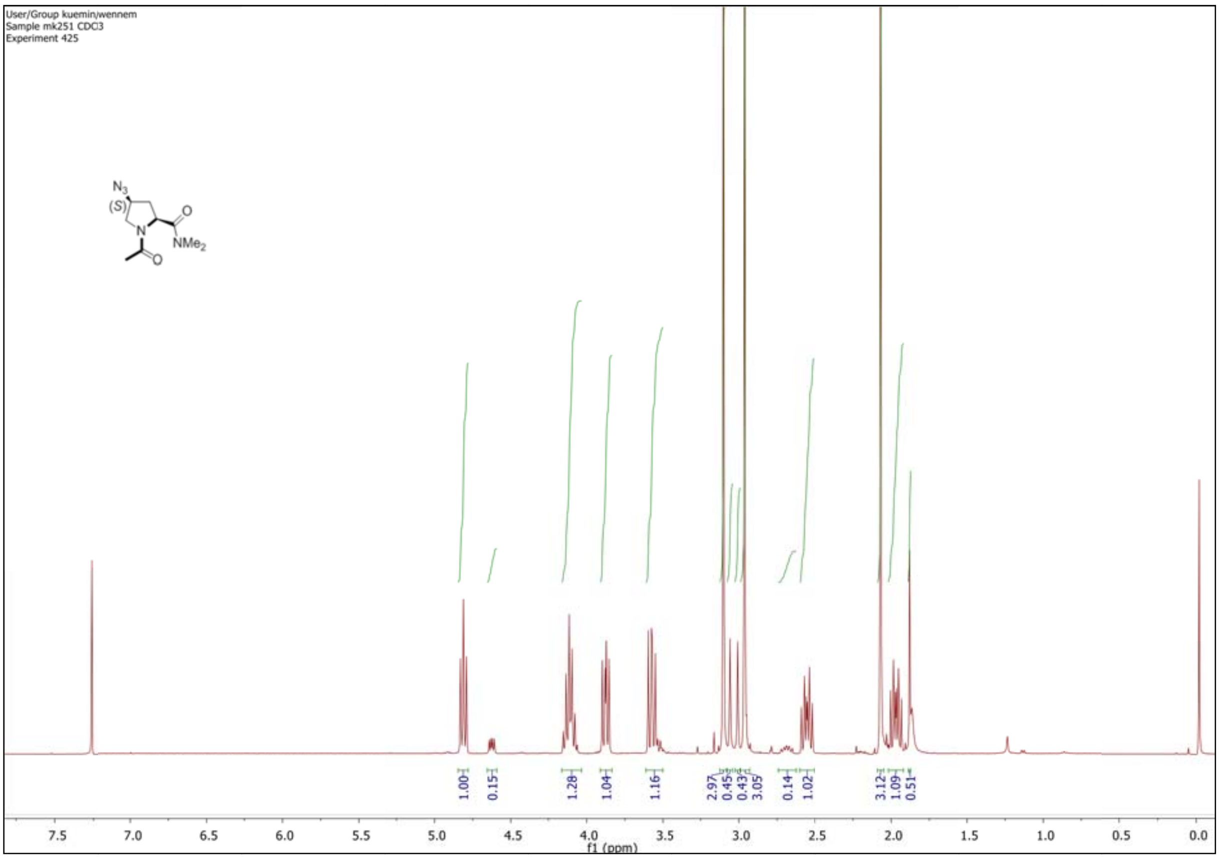

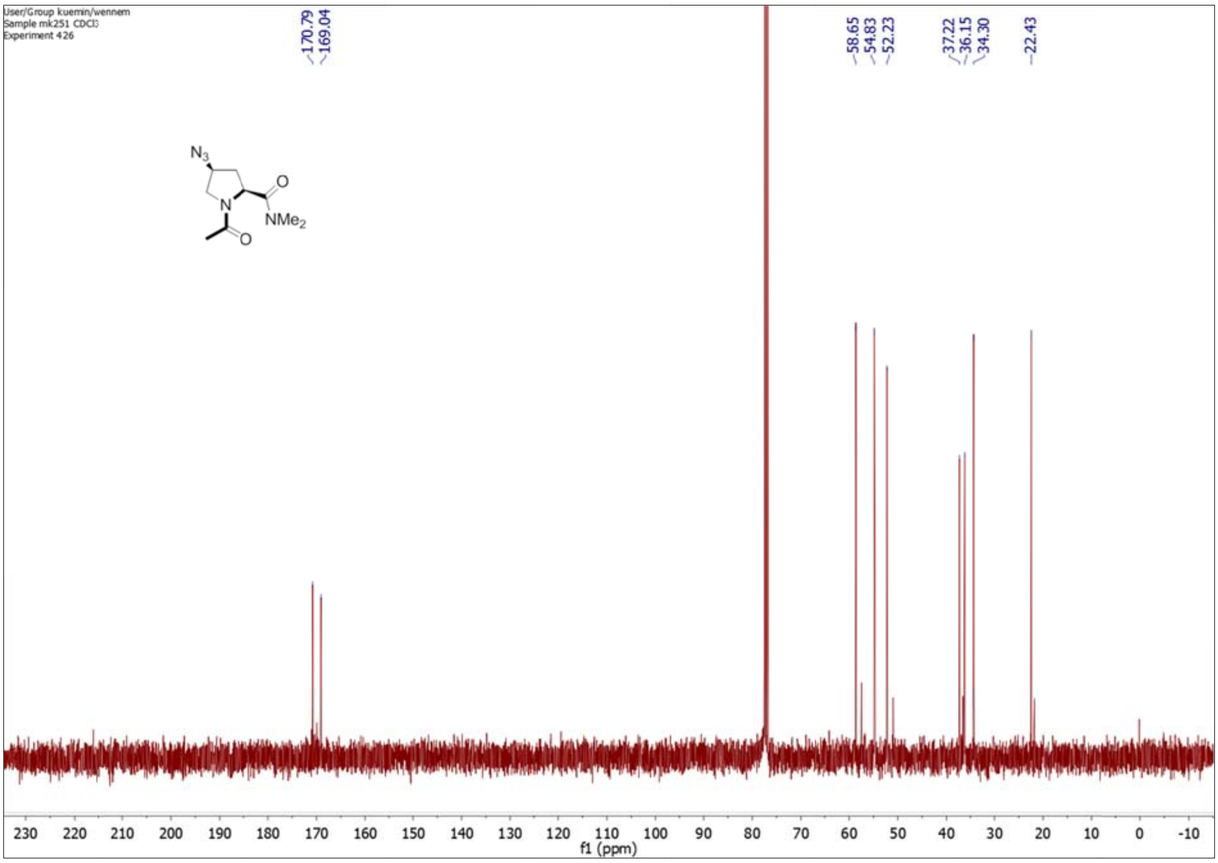

6.20. H-Pro-Pro-Asp-NH$_2$ · TFA 30mM in $CDCl_3$:CD_3OH 9:1

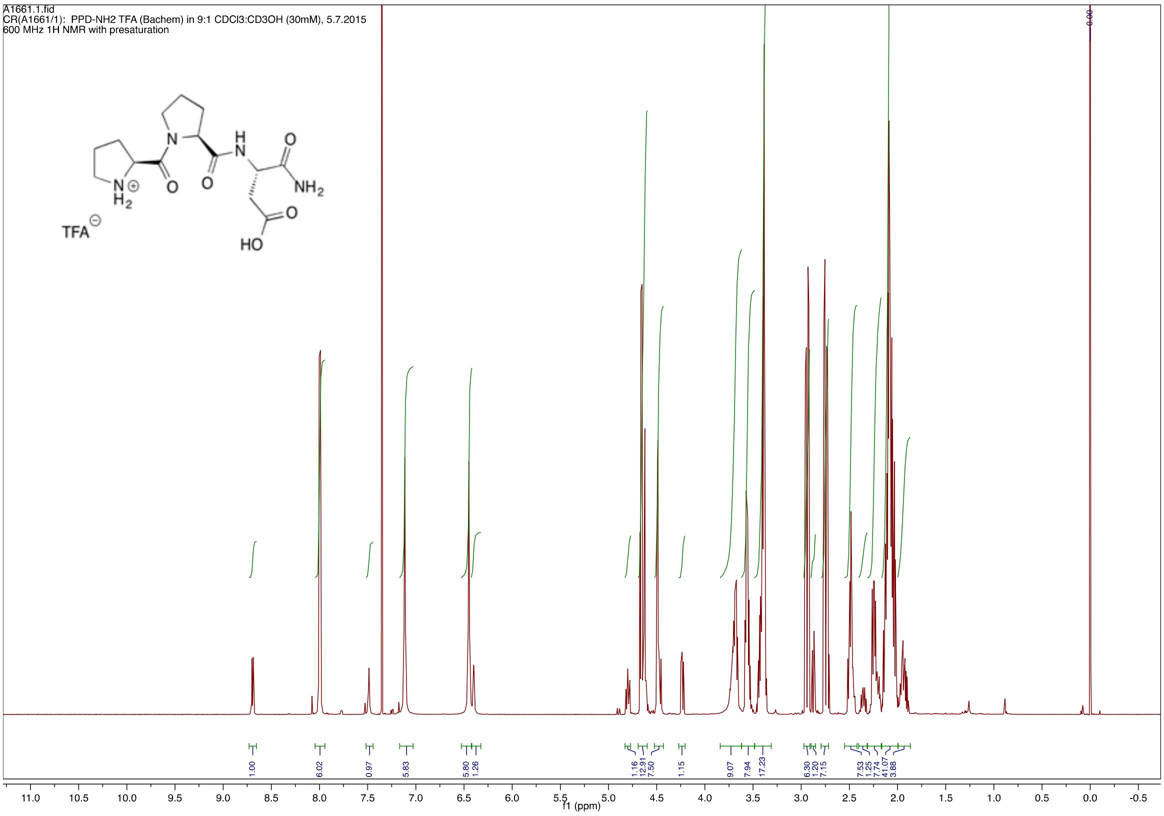

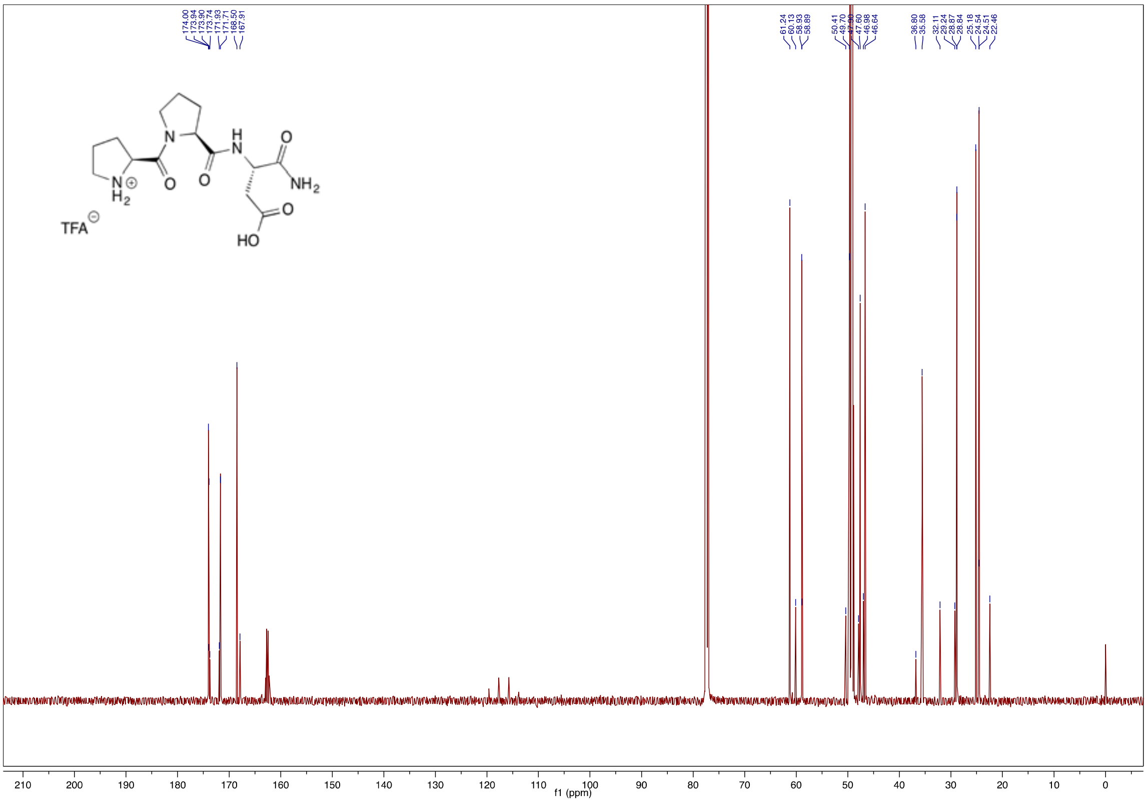

6.21. H-Pro-Pro-Asp-NH_2 · TFA 30mM in DMSO-d_6

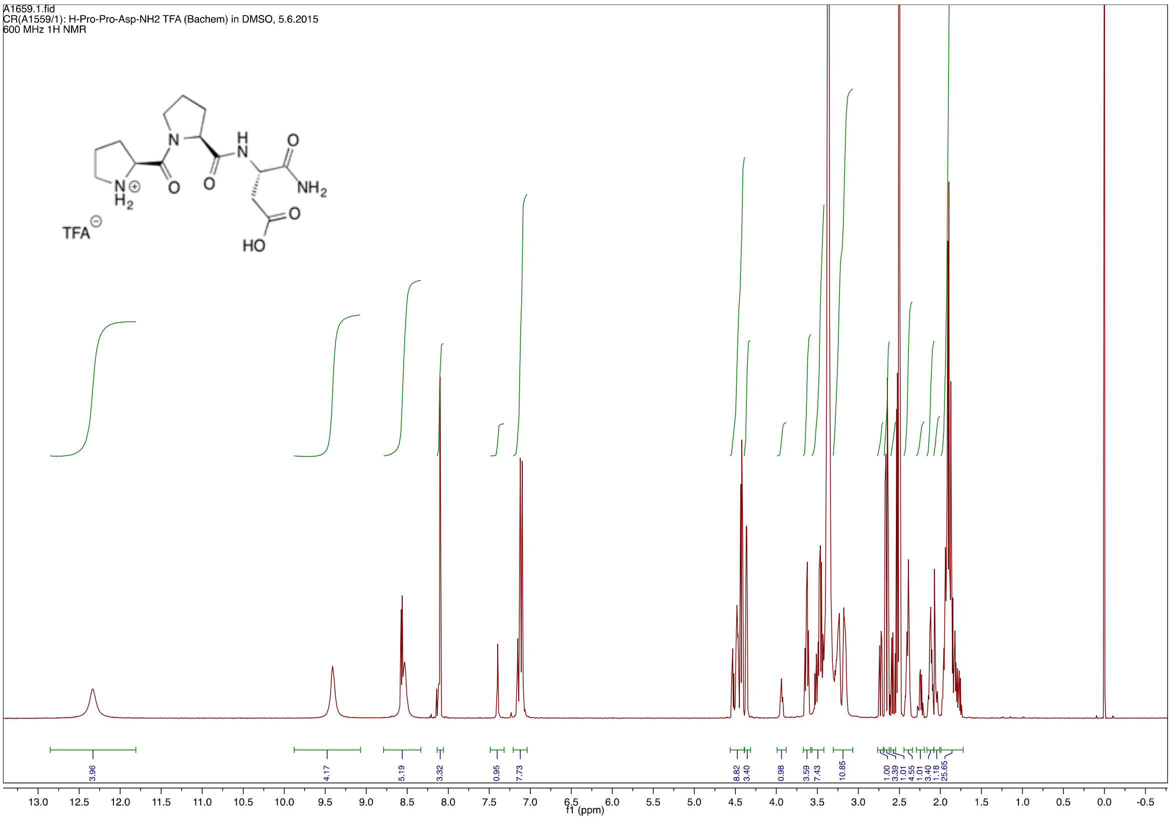

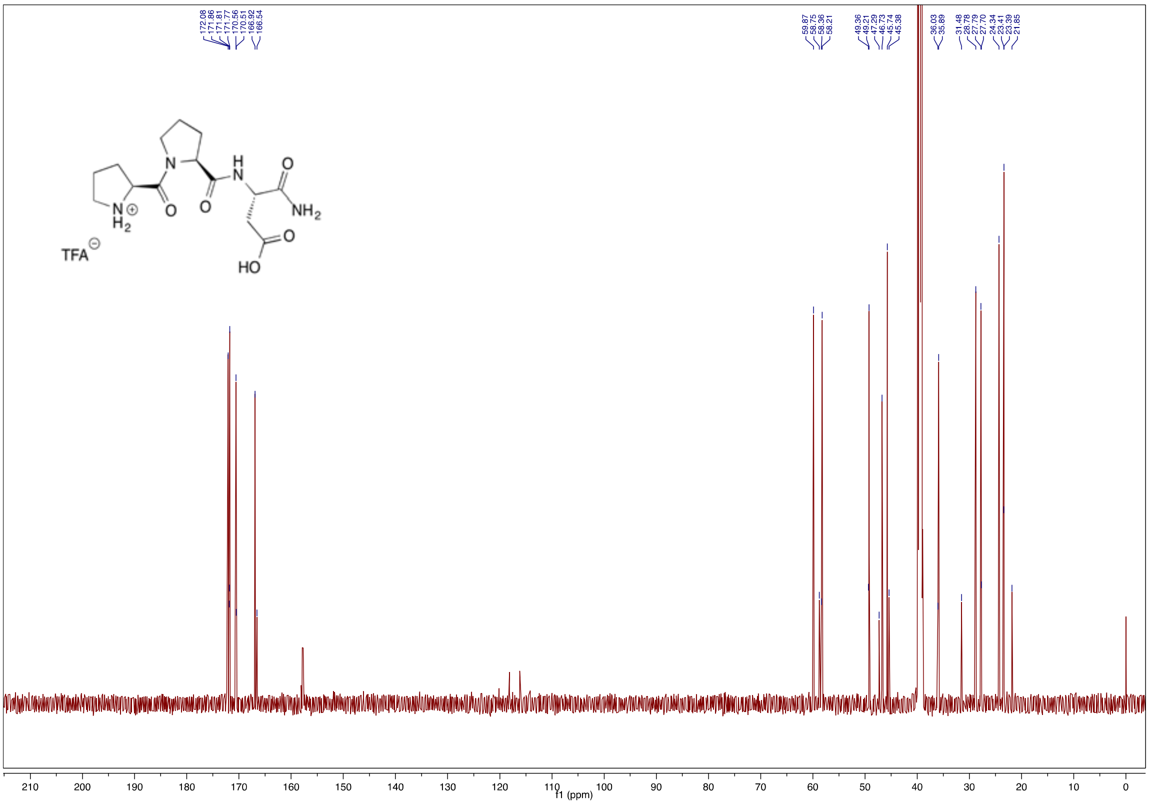

7. References

1. L. E. Bretscher, C. L. Jenkins, K. M. Taylor, M. L. DeRider and R. T. Raines, *J. Am. Chem. Soc.* 2001, **123**, 777.
2. R. W. Newberry, B. VanVeller, I. A. Guzei and R. T. Raines, *J. Am. Chem. Soc.* 2013, **135**, 7843.
3. (a) P. Krattiger, R. Kovàsy, J. D. Revell, S. Ivan and H. Wennemers, *Org. Lett.* 2005, **7**, 1101. (b) J. D. Revell, H. Wennemers, *Tetrahedron* 2007, **63**, 8420.
4. C. Adamo, G. E. Scuseria and V. Barone, *J. Chem. Phys.*, 1999, **111**, 2889.
5. S. Grimme, J. Antony, S. Ehrlich and H. Krieg, *J. Chem. Phys.*, 2010, **132,** 154104.
6. F. Weigend and R. Ahlrichs, *Phys. Chem. Chem. Phys.*, 2005, **7**, 3297.
7. A. Klamt and G. Schuurmann, *J. Chem. Soc., Perk. T. 2*, 1993, 799.
8. (a) TURBOMOLE V6.3 2011, a development of University of Karlsruhe and Forschungszentrum Karlsruhe GmbH, 1989-2007, TURBOMOLE GmbH, since 2007, available from http://www.turbomole.com; (b) TURBOMOLE V6.6 2014, a development of University of Karlsruhe and Forschungszentrum Karlsruhe GmbH, 1989-2007, TURBOMOLE GmbH, since 2007; available from http://www.turbomole.com
